# Electrochemical Oxidative Radical Polar Crossover Route to 1,4‐Keto Carboxylates Mediated by Anchimeric Assistance

**DOI:** 10.1002/chem.202501779

**Published:** 2025-07-02

**Authors:** Ian MacLean, Laura Blanco, Elena Echávarri, Alba Collado, Leyre Marzo, José Alemán

**Affiliations:** ^1^ Organic Chemistry Department (Módulo 1) Universidad Autónoma de Madrid C/ Francisco Tomás y Valiente 7 Madrid 28049 Spain; ^2^ Inorganic Chemistry Department (Módulo 7) Universidad Autónoma de Madrid Madrid 28049 Spain; ^3^ Institute for Advanced Research in Chemical Sciences (IAdChem) Universidad Autónoma de Madrid Madrid 28049 Spain

**Keywords:** 1,4‐keto carboxylate, anchimeric assistance, cross‐polar‐reaction, electrochemistry, radical chemistry

## Abstract

The 1,4‐keto carboxylate scaffold is present in the structure of many biologically relevant compounds. To date, there are only a branch of methods to prepare them, using either transition metal catalysts, or noncommercially available and sensitive starting materials. Herein, we describe a sustainable electrochemical methodology starting from commercially available and highly stable 1,3‐diketones and alkenes to prepare 1,4‐keto aryl and alkyl carboxylates in a highly efficient manner, without the need of an external catalyst or harsh reaction conditions. This electrochemical oxidative radical polar crossover transformation presents a broad scope, is compatible with many functional groups, and can even be applied in flow chemistry. In addition, the key stabilization of the intermediate carbocation by anchimeric assistance allows a high diastereo‐ and regio‐control of the reaction. Moreover, the utility of these 1,4‐keto carboxylates was demonstrated in several derivatizations and applications in the late‐stage functionalization of bioactive compounds.

## Graphical abstract

In this work we developed an electrochemical method for 1,4‐dioxygenated compounds, offering broad scope, functional group compatibility, and diastereoselectivity.

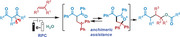

## 1 Introduction

**Scheme 1:**
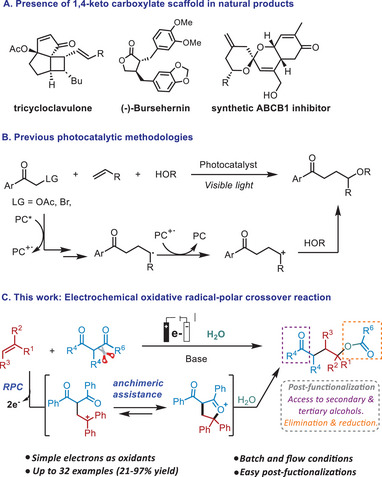
A) Biological relevance of the 1,4‐doxigenated moiety; B) Previous methodologies to prepare 1,4‐diketones and 1,4‐ketooxygenated structures; C) This work: electrochemical synthesis of 1,4‐keto benzoates.

The 1,4‐dioxygenated scaffold is a common structural feature found in thousands of biologically active compounds (Scheme 1A). Well‐established reactions such as the enolate (hetero)coupling described by Baran and coworkers,^[^ ^1^ ^]^ or the coupling of an enamine and an enolate under singly occupied molecular orbital (SOMO) catalysis described by MacMillan and coworkers,^[^ ^2^ ^]^ give access to 1,4‐diketones, with the same functionality in both positions 1 and 4. Visible light photocatalysis has provided several methods to access 1,4‐keto alcohols or 1,4‐keto ethers, starting from α‐bromo or α‐acetate ketones and alkenes, in which the different functionality allows the unsymmetric functionalization of positions 1 and 4. However, the presence of an ether group makes its transformation difficult, or in the case of a hydroxyl group, implies the beforehand activation of the hydroxy group for a subsequent transformation (Scheme 1B).^[^ ^3^, ^4^, ^5^ ^]^ More interesting is the direct preparation of 1,4‐keto carboxylates, in which this carboxylate is already a good leaving group and can be engaged in further transformations (see post‐functionalization, Scheme 1C). Moreover, 1,4‐ketocarboxylate moiety can be found in the structure of natural products such as the tricycloclavulone,^[^ ^6^ ^]^ the (‐)‐bursehernin^[^ ^7^ ^]^ or the synthetic ABCB1 inhibitor^[^ ^8^ ^]^ shown in Scheme 1A. There are scarce methodologies in the literature to prepare 1,4‐keto carboxylate derivatives, and they require either transition metals as catalysts of the reaction,^[^ ^9^, ^10^, ^11^, ^12^ ^]^ or the use of sensitive and/or pre‐synthesized starting materials.^[^ ^13^ ^]^

Electrochemistry has recently been reborn as an ecological and greener synthetic method,^[^ ^14^, ^15^, ^16^, ^17^, ^18^, ^19^, ^20^, ^21^, ^22^, ^23^ ^]^ alternative and, in many cases, complementary^[^ ^24^ ^]^ to photocatalysis.^[^ ^25^ ^]^ Electrochemistry exploits the generation of open‐shell radical intermediates for organic transformations using electrons as reagents under very mild conditions, overcoming the redox power limitation intrinsic to photocatalytic approaches and allowing two consecutive oxidation or reduction steps in one reaction pathway. The potential of these transformations, together with the development of simpler set‐ups accessible to a broader spectrum of the scientific community, has triggered its implementation as a fundamental tool in organic synthesis. Indeed, the electrochemical functionalization of ketones bearing a β‐EWG (electron‐withdrawing group) has opened the door to the preparation of β‐keto spirolactones,^[^ ^26^ ^]^ 3‐dihydrofurane derivatives,^[^ ^27^ ^]^ or 1,4‐dicarbonylic compounds,^[^ ^28^ ^]^ among others.^[^ ^29^ ^]^

Here, we present a sustainable electrochemical method to access 1,4‐keto aryl and alkyl carboxylates under very mild conditions, avoiding the use of expensive catalytic systems, and starting from simple, highly stable, and commercially available alkenes and 1,3‐diketones (Scheme 1C). Under oxidative electrochemical conditions, in the presence of a base, the 1,3‐diketone is oxidized and trapped by the alkene. The neighboring group participation or anchimeric assistance is particularly effective in the stabilization of carbocations,^[^ ^30^ ^]^ allowing the diastereocontrol outcome of a nucleophilic substitution reaction.^[^ ^31^ ^]^ In this case, the radical intermediate undergoes an anodic polar crossover reaction to form a carbocation that is stabilized by anchimeric assistance of the carbonylic oxygen, that allows the diastereoselective synthesis of 1,4‐keto aryl and alkyl carboxylates.

## 2 Results and Discussion

Initially, the reaction between diphenylethylene **1a** and diketone **2a** was studied. Using 2,6‐lutidine as a base, lithium perchlorate (0.1 M) as the electrolyte, a 30:1 mixture of acetone/water as the solvent, Ni, and graphite as electrodes, under a constant 5 mA current for 8 hours in an undivided cell, **3a** was obtained in 86% yield (entry 1, Table 1). Variations in the reaction time, the current intensity, the concentration, or the use of a graphite electrode in the cathode did not improve this result (entries 2–6, Table 1). However, while the use of THF or methanol as a solvent afforded the worst results, with acetonitrile, **3a** was isolated in 97% yield in 8 hours of reaction (entries 7–9, Table 1). Decreasing the reaction time to 4 or 6 hours or the use of other electrolytes afforded lower yields of **3a** (entries 10–12, Table 1). Without the base the reaction works with lower yield, suggesting that the base either favors the enolization of the diketone and the deprotonation of the hemiketal intermediate (see mechanistic discussion for further details) increasing the final efficiency of the process. Finally, the electrochemical nature of the process was corroborated when no conversion was observed with electricity.

**Table 1:** Screening and optimization in batch.^[^ ^a^ ^]^

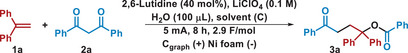		
Entry	Conditions	Yield [%]^[^ ^b^ ^]^
1	Acetone	86
2	Acetone, 3 hours, 1.1 F/mol	62
3	Acetone, 10 mA, 3 hours, 2.2 F/mol	78
4	Acetone, (0.08 M)	69
5	Acetone (0.04 M)	62
6	Acetone, *C*_graph_ instead of Ni foam (‐)	17
**7**	**Acetonitrile**	**97**
8	THF	68
9	MeOH	0
10	Acetonitrile, 4 hours, 1.5 F/mol	77
11	Acetonitrile, 6 hours, 2.2 F/mol	88
12	TBAPF_6_, NH_4_PF_6_, NaClO_4_	66–81^[^ ^c^ ^]^
13	No base	54^[^ ^c^ ^]^
14	No electricity	0

^[a]^ General conditions: **1a** (1.0 mmol), **2a** (0.5 mmol), 2,6‐lutidine (40 mol%) in a 0.1 M LiClO_4_ solvent mixture (3.0 mL) at room temperature.

^[b]^ Isolated yield after flash chromatography purification.

^[c]^ Yield determined by ^1^H NMR using 1,3,5‐trimethoxybenzene as internal standard.

With the optimized conditions in hand (entry 7, Table 1), the scope of the reaction was studied. Initially, we wondered whether the reaction might be susceptible to the alkene substitution. To our delight, the reaction with styrene or the α‐methyl styrene afforded the final products **3b** and **3c** with good to very good yields, and **3a** could be obtained on two‐times larger scale (1 mmol) with a slight decrease in yield. Additionally, we confirmed the structure of **3c** by X‐ray analysis.^[^ ^32^ ^]^ Moreover, di‐ and trisubstituted olefines were also tolerated, obtaining **3d**, **3e, 3f,** and **3** **h** in moderate to good yields, and with a complete diastereoselectivity for the *anti*adducts in compounds **3d, 3e,** and **3** **h** (for further explanation about the observed diastereoselectivity, see Scheme 5). However, with the highly hindered 1,1,2,2‐tetraphenylethene no reactivity was found (Table 2).^[^ ^33^ ^]^ Interestingly, when a conjugated diene is subjected to the reaction conditions, only the first double bond is activated, affording **3i** as the only regioisomer in very good yield. Then, the effect of different substituents in the aromatic ring of the alkene was examined. The electronic character of the substituents did not have a significant impact on the reaction. Thus, the final products were obtained with good to very good yields either with electron donating (**3j**, **3o**) or electron‐withdrawing substituents in the aromatic ring (**3k**‐**3 m**, **3p**, **3q**). Moreover, the reaction tolerated heteroarenes such as pyridine, that afforded **3n** in moderate yield. Next, the scope of 1,3‐diketones **2** was studied. The reaction with symmetric diketones afforded good results independently of the substituent present in the aromatic ring (**3r**–**3v**). This behavior was corroborated when studying unsymmetric diketones with one electron donating substituent and a neutral or electron withdrawing one (**3 w**, **3x**), obtaining in both cases an equimolecular mixture of the two possible regioisomers. The reaction also tolerated electron deficient or electron rich heteroaromatic substituents, or the increase of steric hindrance afforded by the substitution of the methylenic position, obtaining **3y**–**3aa** in moderate yields. In these cases, we corroborated the structure of **3aa** by X‐ray diffraction analysis.^[^ ^32^ ^]^ Delightfully, asymmetric 1,3‐diketones bearing one phenyl and a methyl substituent presented a complete regioselectivity toward the formation of compound **3ab** due to the lower steric hindrance of the methyl group. Finally, the di‐*tert*‐butyl 1,3‐diketone afforded **3ac** in 84% yield in the presence of DBU, instead of 2,6‐lutidine.

**Table 2:** Scope of the electrochemical synthesis of **3**.^[^ ^a^ ^]^

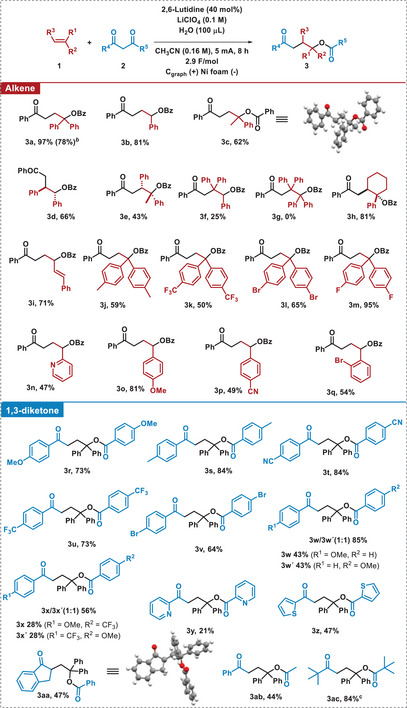

^[a]^ General conditions: **1** (1.0 mmol), **2** (0.5 mmol), 2,6‐lutidine (40 mol%) in a 0.1 M LiClO_4_, CH_3_CN/H_2_O 30:1 (3.0 mL) at room temperature.

^[b]^ This reaction was carried out at larger scale using 2.0 mmol of **1a** and 1.0 mmol of **2a**.

^[c]^ This reaction was carried out using 40 mol% of DBU instead of 2,6‐lutidine as base.

**Scheme 2:**
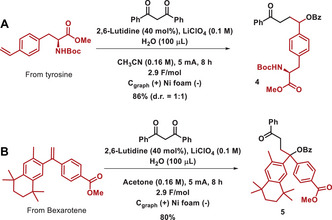
Late‐stage functionalization of natural products.

To prove the robustness and applicability of the methodology, it was applied to the late‐stage functionalization of two natural products. Thus, a derivative of the hormone tyrosine underwent the reaction smoothly, affording compound **4** in 86% yield (Scheme 2A). In addition, a derivative of bexarotene, that is an antineoplastic used to treat some types of cancer, could be functionalized, affording compound **5** in 80% yield (Scheme 2B).^[^ ^34^ ^]^

**Scheme 3:**
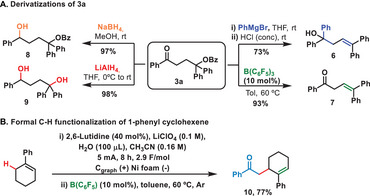
Derivatizations of the 1,4‐keto benzoates derivatives **3**.

The products obtained in this electrochemical transformation are highly versatile, since many further functionalizations and derivatizations can be performed. For example, **3a** in the presence of phenyl magnesium bromide followed by the treatment with acidic media afforded the alkene **6** in 73% yield. However, in the presence of the Lewis acid tris(pentafluorobencene)borane the β,γ‐unsaturated ketone **7**, challenging to prepare following other methodologies, is obtained in excellent 93% yield (Scheme 3A‐right). Moreover, adjusting the strength of the reducing agent, it is possible to isolate the 1,4‐hydroxybenzoate **8** (through the exclusive reduction of the ketone with NaBH_4_) or the 1,4‐diol **9** (upon reduction of the ketone and the benzoate with LiAlH_4_) (Scheme 3A‐left). The robustness of the method could be further proved through the formal α‐CH functionalization of the 1‐phenylcyclohexene. Thus, in a two‐step procedure, compound **10** bearing an acetophenone residue was prepared in a very good overall 77% yield (Scheme 3B), like the formal alkylation reaction of the double bond.

Designing efficient and sustainable methods for large‐scale production remains one of the industry's key challenges today. Flow chemistry has been attracting increasing interest among scientists due to its ability to enable the development of methodologies that are simple, safe, highly efficient, and generate minimal waste.^[^ ^35^, ^36^ ^]^ Given the importance of the structures obtained earlier, we opted to develop this electrochemical methodology under flow conditions (Table 3). The flow system consists of an electrochemical undivided‐cell reactor with a fixed reactor volume (see optimization of the reaction conditions in Table S1 in Supporting Information). Under the optimized reaction conditions, **3a** could be isolated in 63% yield in only 42 minutes of residence time. It is important to highlight the higher production of the flow process that allowed the preparation of 0.126 mmol of **3a** per hour compared to the 0.060 mmol per hour obtained under batch conditions. This flow synthesis could be applied to differently substituted alkenes such as 4‐vinylanisole, 1‐phenylcyclohexene or 2‐bromostyrene, producing compounds **3o**, **3** **g**, and **3q** with moderate to good yields. Similarly, diaryl substituted 1,3‐diketones bearing either electron‐donating or electron‐withdrawing substituents could be carried out in good yield (**3r**, **3t**, **3v**). In general, the relative reactivity under flow conditions is in accordance with the one observed under batch conditions.

**Table 3:** Substrate scope of the electrochemical synthesis of **3** under electro‐flow conditions.^a^

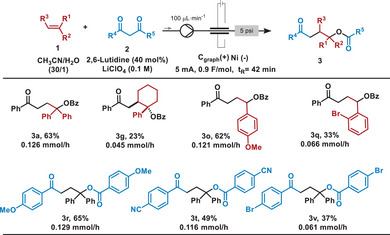

^[a]^ Reaction conditions: **1** (0.28 mmol), **2** (0.14 mmol), 2,6‐lutidine (40 mol%), LiClO_4_ (0.1 M) and H_2_O (1.75 mmol) at constant current (5.0 mA) in dry acetontrile (0.055 M) at room temperature for 42 minutes of residence time (196 min of reaction time), using *C*_graph_ as anode and Ni as cathode in a recirculating system.

**Scheme 4:**
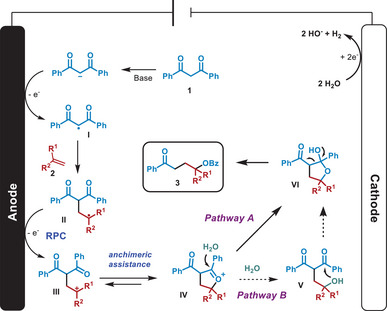
Mechanistic proposal.

Finally, a mechanism is proposed in Scheme 4. Initially, the enolate formed upon deprotonation of the 1,3‐diketone **1a** (*E*_2a_ = 1.43 V vs. SCE;^[^ ^37^ ^]^ *E*_1a_ = 2.41 V vs. SCE;^[^ ^38^ ^]^) is oxidized in the anode to form the intermediate radical **I**. This radical is consecutively trapped by the alkene **1a**, affording the radical intermediate **II**, that undergoes radical polar crossover in the anode to the carbocation **III**. This carbocation **III** is stabilized through anchimeric assistance^[^ ^30^, ^31^ ^]^ by the oxygen from one of the carbonyl groups to form intermediate **IV**. Then, a molecule of H_2_O can add to the carbonyl carbon, yielding intermediate **VI** (pathway A, Scheme 4) or to the anchimerically assisted position to afford intermediate **V** that evolves to **VI** through nucleophilic attack of the alcohol to the carbonyl (pathway B). Further ring opening of **VI** yields **3**.

**Scheme 5:**
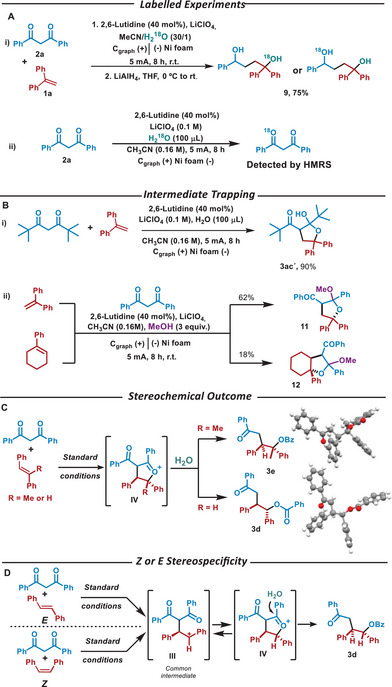
Mechanistic investigations.

To get further insight into the most probable mechanistic pathway, the reaction was performed in the presence of H_2_O^18^, followed by a reduction step in which the diol **9** was isolated. HMRS analysis revealed the presence of ^18^O in **9** (Scheme 5A‐i). Additionally, **1a** was submitted to the standard electrochemical reaction conditions in the presence of H_2_^18^O obtaining **1a^18^** determined by HMRS (Scheme 5A‐ii). This result indicated that the ^18^O could proceed either from the attack of water to intermediate **IV**, or from **1a^18^**. Therefore, from labelling experiments, it is not possible to elucidate between pathways A or B, and additional mechanistic probes need to be performed.

The reaction between the di‐*tert*‐butyl 1,3‐diketone and 1,1‐diphenylethylene in the presence of 2,6‐lutidine as a base was also performed, affording the hemiketal **3ac´** that corroborated the formation of intermediate **V** (Scheme 5B‐i). Moreover, to get further evidence on the attack of the nucleophile to the carbonyl group, the electrochemical reaction between **1a** and 1,1‐diphenylethene or 1‐phenyl‐cyclohexene in the presence of methanol, instead of water, was performed (Scheme 5B‐ii). In both cases, the methanol‐derived ketal intermediates **11** and **12** were isolated, corroborating the addition of the molecule of water to the carbonyl group. In addition, the excellent regioselectivity observed for allylic derivative **3i** can be easily explained according to this reaction mechanism (see Table 2). Starting from a diene, intermediate **III** is an allylic carbocation that can be stabilized through anchimeric assistance, forming a five‐ or seven‐membered ring in intermediate **IV**. According to Baldwin's rules, the formation of a five‐membered ring is more favorable than the seven‐membered one, explaining why only the functionalization of the terminal alkene is observed in a completely regioselective manner (see compound **3i,** Table 2). Notably, the formation of a single regioisomer also supports the radical polar crossover mechanism.

Finally, the observed diastereoselectivity can be explained by attending to the stereochemical outcome of the reaction (Scheme 5C). Stereochemically, **I** reacts with **2** to form an initial C─C bond through one of the prochiral faces (**II**, Scheme 4). After the oxidation of **II**, the carbonylic oxygen attacks the carbocation **III** through the same prochiral face to afford **IV** as a single diastereoisomer in compounds **3e** and **3d** (see X‐ray analyisis,^[^ ^32^ ^]^ see right Scheme 5C) as well as also observed in compounds **3ac’**, **11,** or **12**. The presence of a carbocation intermediate was indirectly confirmed through a test using two olefins with opposite *Z* or *E* configurations. Starting from both, the same compound **3d** with an *anti*configuration was obtained, indicating that they must share a common intermediate carbocation (Scheme 5D), and therefore, a nonstereospecific reaction.

## 3 Conclusion

In summary, a sustainable electrochemical method to prepare highly versatile 1,4‐keto aryl and alkyl carboxylates has been developed.^[^ ^39^ ^]^ The reaction presents high functional group tolerance and is not dependant on the electronic effect of the substituents in the diketone or in the alkene. Moreover, it presents a high diastereoselectivity, which is controlled by the anchimeric assistance of a carbonyl group that stabilizes the carbocationic intermediate generated in the reaction. The robustness of the method could be demonstrated through the late‐stage functionalization of two natural product derivatives, and the utility of the final products corroborated through different derivatization reactions. The mechanistic proposal based on the formation of a dihydrofuranium intermediate and an hemiketal intermediate could be corroborated through the isolation of different reaction intermediates.

## Conflict of Interest

The authors declare no conflict of interest.

## Supporting information



Supporting information

## Data Availability

The data that support the findings of this study are available in the supplementary material of this article.
