# Supplementary material for: Electrochemical Oxidative Radical Polar Crossover Route to 1,4‐Keto Carboxylates Mediated by Anchimeric Assistance
Source: Chemistry. 2025 Jul 2;31(41):e202501779. doi: 10.1002/chem.202501779 (PMC12284624; doi:10.1002/chem.202501779)
Supplement: Supplementary file 1 — Supporting information [file CHEM-31-e202501779-s001.pdf]

SUPPORTING INFORMATION

Electrochemical Oxidative Radical Polar Crossover Route to 1,4-Keto Carboxylates Mediated by Anchimeric Assistance

Ian MacLean,[a] Laura Blanco,[b] Elena Echávarri,[a] Alba Collado,[b,c] Leyre Marzo,*[a,c] José Alemán*[a,c]

[a] Organic Chemistry Department (Módulo 1) Universidad Autónoma de Madrid, 28049 Madrid (Spain)

[b] Inorganic Chemistry Department (Módulo 7) Universidad Autónoma de Madrid, 28049 Madrid (Spain)

[c] Institute for Advanced Research in Chemical Sciences (IAdChem) Universidad Autónoma de Madrid, 28049 Madrid (Spain)

*E-mail: leyre.marzo@uam.es, jose.aleman@uam.es

TABLE OF CONTENT

1. General methods and materials ... 2

2. Electrochemical setup ... 2

3. Electrochemical flow setup ... 3

4. Synthesis and characterization of reactants and products ... 3

4.1. General procedure A: Synthesis of alkenes ... 3

4.1.1. General procedure A1: Synthesis of aryl alkenes 1j and 1m ... 3

4.1.2. Synthesis of 4,4'-(ethene-1,1-diyl)bis((trifluoromethyl)benzene) 1k ... 4

4.1.3. Synthesis of 4,4'-(ethene-1,1-diyl)bis(bromobenzene) 1l ... 5

4.1.4. Synthesis of methyl (*S*)-2-((tert-butoxycarbonyl)amino)-3-(4-vinylphenyl)propanoate 1r 5

4.1.5. Synthesis of bexarotene methyl ester, methyl 4-(1-(3,5,5,8,8-pentamethyl-5,6,7,8-tetrahydronaphthalen-2-yl)vinyl)benzoate 1s. ... 6

4.2. General procedure B: Synthesis of 1,3-Diketones 2c, 2d, 2e, 2f, 2g and 2h ... 6

4.3. Synthesis of 2-benzoyl-2,3-dihydro-1H-inden-1-one 2k. ... 8

4.4. General procedure C: Synthesis of 4-oxobenzoates ... 9

4.5. Synthesis of 4-oxobenzoates under flow conditions ... 9

4.5.1. Optimization ... 9

4.5.2. General procedure D: Electro-flow synthesis of 4-oxobenzoates ... 10

4.6. Scale up of the reaction ... 21

4.7. Unsuccessful examples ... 22

5. Derivatizations ... 23

5.1. Synthesis of 4-hydroxy-1,1,4-triphenylbutyl benzoate 6. ... 23

5.2. General procedure E: Elimination reactions ... 23

5.3. Synthesis of 4-hydroxy-1,1,4-triphenylbutyl benzoate 8. ... 24

5.4. Synthesis of 1,1,4-triphenylbutane-1,4-diol 9. ... 24

6. Mechanism experiments ... 25

6.1. Isotopic labelling experiments ... 25

7. NMR spectroscopic data ... 29

8. Single Crystal X-Ray Diffraction of 3c, 3aa, 3d and 3e ... 82

9. References ... 84

1. General methods and materials

Commercial-grade reagents and solvents were purchased from Acros Organics, Alfa Aesar, Fluorochem, Sigma-Aldrich, BLD Pharm, and TCI Chemicals. All synthesis were carried out under air, using solvents with analytical standard grades. Analytical TLC was performed using pre-coated aluminum-backed plates (Merck TLC Silicagel 60 F254) and visualized by ultraviolet irradiation. Chromatographic purification of products was accomplished by flash chromatography using silica gel (Merck Geduran® Si 60) unless another stationary phase is specified. NMR spectra were acquired on a BRUKER AVANCE 300 or BRUKER AVANCE NEO 500 spectrometer operating at 300 or 500 MHz for ^{1}H and 75 or 125 MHz for ^{13}C. Chemical shifts were internally referenced to residual solvent signals ($CDCl_3$: δ 7.26 ppm for ^{1}H NMR and δ 77.0 ppm for ^{13}C-NMR). Data for ^{1}H NMR are reported as follows: chemical shift (δ ppm), multiplicity (*s* = singlet, *d* = doublet, *t* = triplet, *q* = quartet, *m* = multiplet, *br* = broad), coupling constant (Hz) and integration. The diastereomeric ratios were determined by ^{1}H NMR analysis of the crude reaction mixture through integration of diagnostic signals. High-Resolution Mass Spectra (HRMS) were obtained on an Agilent Technologies 6120 Quadrupole LC/MS coupled with an SFC Agilent Technologies 1260 Infinity Series instrument for the MS (ESI) (Electrospray Ionization). MassWorks software version 4.0.0.0 (Cerno Bioscience) was used for the formula identification. MassWorks is an MS calibration software which calibrates isotope profiles to achieve high mass accuracy and enables elemental composition determination on conventional mass spectrometers of unit mass resolution allowing highly accurate comparisons between calibrated and theoretical spectra.

Crystal of **3c**, **3aa**, **3d** and **3e** were obtained by the controlled diffusion of n-pentane in a saturated solution of the corresponding complex in dichloromethane. Suitable crystals were mounted and placed on a MiTeGen micromount (for **3aa**, **3d** and **3e**) or on a Hampton Research cryoloop (for **3c**) on an XtaLAB Synergy R, HyPix-Arc 100 diffractometer equipped with a graphite monochromated CuKα radiation source (λ = 1.5418 Å). Structures were solved with the ShelXT 2018/2 (Sheldrick, 2018) structure solution program using the Intrinsic Phasing solution method and by using **Olex2** (Dolomanov et al., 2009) as the graphical interface. The model was refined with version 2018/3 of ShelXL 2018/3 (Sheldrick, 2015) using Least Squares minimisation. Crystals of **3d** were kept at 149.97(10) K and crystals of **3c**, **3aa** and **3e** at 250.00(10) K during data collection.

2. Electrochemical setup

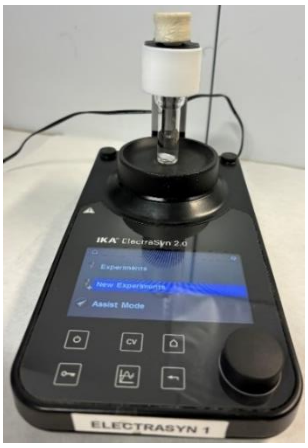

All electrodes and equipment (ElectraSyn 2.0) used for the batch electrochemical experiments were acquired from IKA. The electrodes used have dimensions of 8 mm width, 50 mm length, and 1 mm thickness. The vials employed have capacities of 10 mL and 5 mL.

3. Electrochemical flow setup

All flow experiments were carried out using a commercially available Vapored Ion electrochemical reactor fixed on an E-series Vapourtec equipment. The reactor has a channel volume of 0.6 mL and was equipped with two electrodes of 5 × 5 cm size with a 0.5 mm spacer. For the study of flow optimization, the electrodes used were graphite (working) and nickel (counter) and $LiClO_4$ as electrolyte.

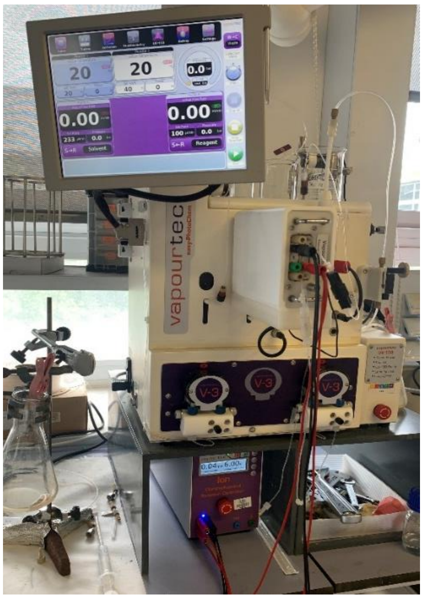

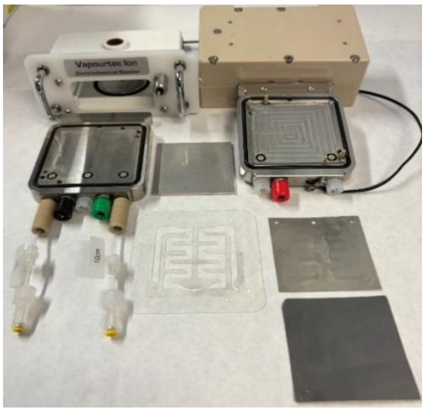

4. Synthesis and characterization of reactants and products

4.1. General procedure A: Synthesis of alkenes

4.1.1. General procedure A1: Synthesis of aryl alkenes 1j and 1m

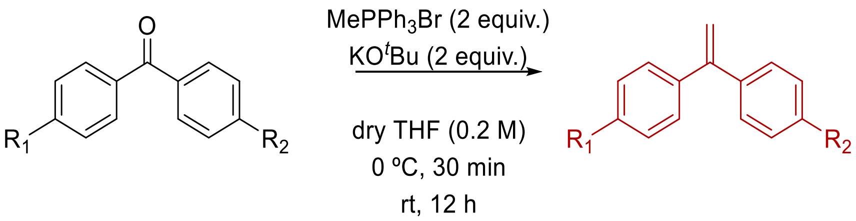

In a 100 mL round bottom flask with a stirring bar, methyltriphenylphosphonium bromide (2.8 g, 8.0 mmol) and potassium tert-butoxide (897.6 mg, 8.0 mmol) were dissolved in 20.0 mL of dry THF at 0 ºC under nitrogen. The reaction mixture was stirred at 0 ºC for 30 min. Then, the corresponding benzophenone (4.0 mmol) was added. The mixture was stirred at room temperature during 12 h. 50 mL of H_2O were added to quench the reaction, and the THF was removed under vacuum. 1 M aq. solution of HCl (20.0 mL) was added and the mixture was extracted with EtOAc (3 x 30 mL). The combined organic layers were dried with anhydrous $MgSO_4$, filtered, and concentrated until dryness. The residue was further purified by flash column chromatography on silica gel (Cyclohexane/EtOAc) to provide the aryl alkenes **1j** and **1m**.

***4,4'-(Ethene-1,1-diyl)bis(methoxybenzene)dione* (1j)**

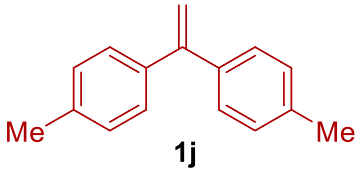

1j

Following the **general procedure A1**, methyltriphenylphosphonium bromide (2.86 g, 8.0 mmol), di-p-tolylmethanone (841.4 mg, 4.0 mmol), potassium tert-butoxide (897.7 mg, 8.0 mmol) gave product **1j** (735 mg, 88%) as a colourless oil after purification (99:1 Cyclohexane/EtOAc).

^{1}H NMR (300 MHz, $CDCl_3$) δ 7.15 (dt, *J* = 8.1 Hz, 4H), 7.05 (dt, *J* = 8.1 Hz, 4H), 5.29 (s, 2H), 2.28 (s, 6H).

Spectroscopic data are consistent with those reported in the literature.[1]

***4,4'-(Ethene-1,1-diyl)bis(methoxybenzene)* (1m)**

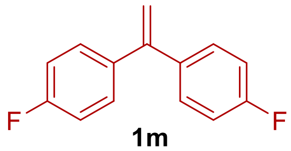

1m

Following the **general procedure A1**, methyltriphenylphosphonium bromide (2.86 g, 8.0 mmol), di-p-tolylmethanone (872.8 mg, 4.0 mmol), potassium tert-butoxide (897.7 mg, 8.0 mmol) gave product **1m** (784.9 mg, 91%) as a colourless oil after purification (99:1 Cyclohexane/EtOAc).

^{1}H NMR (300 MHz, $CDCl_3$) δ 7.36 – 7.23 (m, 4H), 7.10 – 6.96 (m, 4H), 5.40 (s, 2H).

Spectrocopic data are consistent with those reported in the literature.[1]

4.1.2. Synthesis of 4,4'-(ethene-1,1-diyl)bis((trifluoromethyl)benzene) 1k

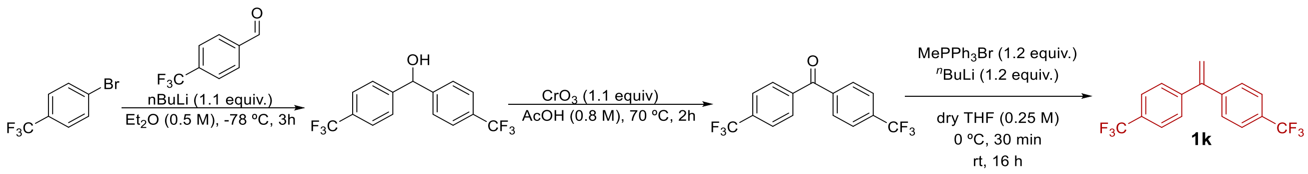

1-Bromo-4-(trifluoromethyl)benzene (1.5g, 6.7 mmol) was dissolved in Et_2O (14 mL, 0.5 M) under argon atmosphere. The solution was cooled to -78 °C and *n*-butyllithium (2.7 mL, 2.5 M in *n*-hexane, 1.1 equiv.) was added dropwise and the resulting mixture was stirred for 1 h. A solution of 4-(trifluoromethyl)benzaldehyde (6.7 mmol, 1.1 equiv.) in Et_2O (1.4 mL) was added dropwise at -78 °C and stirred for 3 h. After this time, the solution was allowed to warm to room temperature. The resulting mixture was quenched with 10 mL of an aqueous $NaHCO_3$ solution. The aqueous layer was separated and extracted (3 x 10 mL) with Et_2O. The combined organic layers were washed with brine, dried over anhydrous $MgSO_4$, filtered, and concentrated under vacuum until dryness. The product was used directly for next step without further purification.

Bis(4-(trifluoromethyl)phenyl)methanol was dissolved in glacial acetic acid (8.5 mL, 0.8 M) in a 25 mL round-bottom flask equipped with a condenser. CrO_3 (7.33 mmol, 1.1 equiv.) was added at room temperature and the solution was heated at 70 °C for 2 h. The reaction mixture was then diluted with 10 mL of water and extracted with *n*-hexane (3 x 10 mL). The combined organic layers were washed with a 2M NaOH aqueous solution until the washings remained basic, dried over anhydrous $MgSO_4$, filtered, and concentrated under vacuum until dryness. The residue was purified by flash column chromatography on silica gel (98:2 Cyclohexane/EtOAc) to afford a white solid (1.32 g, 63%).

Methyltriphenylphosphonium bromide (1.9 mmol, 1.2 equiv.) was added to a flame-dried round-bottom flask and dissolved in THF (6.3 mL, 0.25 M) under argon atmosphere. The suspension was vigorously stirred and *n*-butyllithium (0.75 mL of 2.5M solution in *n*-hexane, 1.2 equiv.) was added dropwise at 0 °C. The reaction was stirred for 30 min until a bright yellow heterogeneous mixture was achieved. Then, the corresponding 1,1-diarylketone (1.6 mmol) was added slowly. Upon complete addition, the ice bath was removed, and the reaction was stirred overnight. Then, the reaction mixture was washed with brine and extracted with EtOAc (3 × 10 mL). The combined organic layers were dried over anhydrous $MgSO_4$ and concentrated under vacuum. The residue was purified by silica gel flash chromatography (99:1 Cyclohexane/EtOAc) to afford the alkene **1k** (383.4 mg, 77%) as a white solid.

^{1}**H NMR** (300 MHz, $CDCl_3$) δ 7.52 (d, *J* = 8.0 Hz, 4H), 7.34 (d, *J* = 8.0 Hz, 4H), 5.60 (s, 2H).

Spectroscopic data are consistent with those reported in the literature.[2]

4.1.3. Synthesis of 4,4'-(ethene-1,1-diyl)bis(bromobenzene) 1l

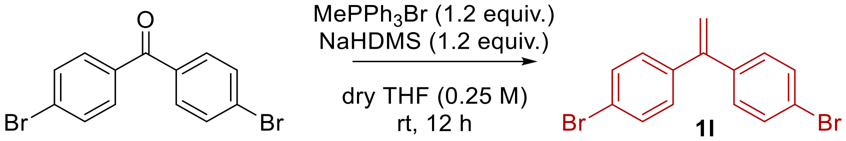

1l

Under argon atmosphere, a 100 mL round bottom flask was charged with methyltriphenylphosphonium bromide (3.5 mmol, 1.2 equiv.) and dry THF (14.0 mL, 0.25 M). Then, sodium hexamethyldisilazide (5.88 mL of a 0.6 M THF solution, 3.5 mmol, 1.2 equiv.) was added into the solution and stirred at room temperature for 1 hour. 4,4'-dibromobenzophenone (1.00 g, 2.9 mmol) was added to the solution and stirred at room temperature for 16 hours. Et_2O was added into the reaction mixture until a precipitate was formed and filtered. The filtrate was concentrated in vacuo and the residue was purified by flash column chromatography on silica gel (95:5 Cyclohexane/EtOAc) to afford 4,4'-(ethene-1,1-diyl)bis(bromobenzene) **1l** (713 mg, 72%) as a white solid.

^{1}**H NMR (300 MHz, CDCl3**) δ 7.57 (d, *J* = 8.7 Hz, 4H), 7.30 (d, *J* = 8.7 Hz, 4H), 5.54 (s, 2H).

Spectroscopic data are consistent with those reported in the literature.[3]

4.1.4. Synthesis of methyl (*S*)-2-((tert-butoxycarbonyl)amino)-3-(4-vinylphenyl)propanoate 1r

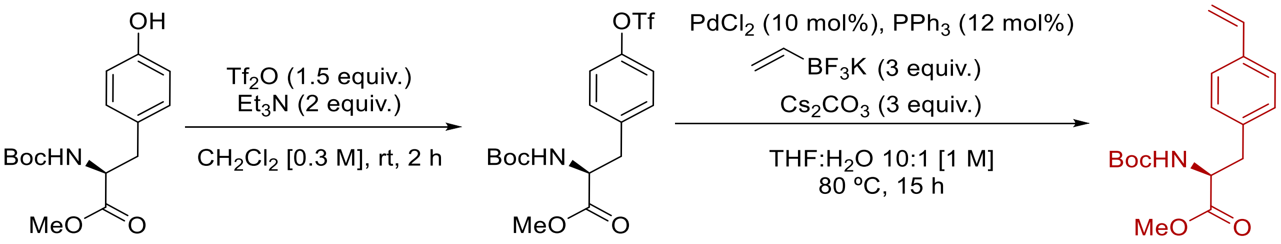

1Sa **1r**

To a solution of Boc-Tyr-OMe (1.18 g, 4.0 mmol) in dry CH_2Cl_2 (13.3 mL, 0.3 M) under a N_2 atmosphere, dry triethylamine (1.11 mL, 2 equiv.) was added. The reaction mixture was cooled to 0 °C, and trifluoromethanesulfonic anhydride (1.0 mL, 1.5 equiv.) was added dropwise. The resulting mixture was

warmed to room temperature and stirred for 2 h. After this time, the reaction was quenched with water (4.0 mL) and extracted with CH_2Cl_2 (3 x 10 mL). The combined organic layers were dried over $MgSO_4$, and the resulting residue was purified by silica gel flash chromatography (90:10 Cyclohexane/EtOAc) to give methyl (*S*)-2-((*tert*-butoxycarbonyl)amino)-3-(4-(((trifluoromethyl)sulfonyl)oxy)phenyl)propanoate (1.16 g, 68%) as a white solid.

^{1}H NMR (300 MHz, $CDCl_3$) δ 7.25 – 7.15 (m, 4H), 5.07 – 4.97 (br, 1H), 4.69 – 4.50 (br, 1H), 3.71 (s, 3H), 3.17 (dd, *J* = 13.9, 5.8 Hz, 1H), 3.03 (dd, *J* = 13.8, 6.6 Hz, 1H), 1.41 (s, 9H).

Spectroscopic data are consistent with those reported in the literature.[4]

A sealed tube was charged with (*S*)-2-((*tert*-butoxycarbonyl)amino)-3-(4-(((trifluoromethyl)sulfonyl)oxy)phenyl)propanoate **1Sa** (1.16 g, 2.72 mmol), potassium vinyltrifluoroborate (5.4 mmol, 2 equiv.), palladium (II) chloride (0.27 mmol, 10 mol%), triphenylphosphine (0.33 mmol, 12 mol%) and cesium carbonate (8.2 mmol, 3 equiv.). Then, three vacuum/N_2 cycles were performed. THF (2.7 mL, 1.0 M) and distilled water (0.27 mL) were added, the tube was sealed, and the reaction was stirred at 80 ºC for 15 h. The mixture was filtered, the filtrate was extracted with water and CH_2Cl_2 (3 x 30 mL). The combined organic layers were dried over $MgSO_4$, filtered and concentrated. The resulting residue was purified by silica gel flash chromatography (92:8 Cyclohexane/EtOAc) to give methyl (*S*)-2-((*tert*-butoxycarbonyl)amino)-3-(4-vinylphenyl)propanoate **1r** as a white solid.

^{1}H NMR (300 MHz, $CDCl_3$) δ 7.33 (d, *J* = 8.1 Hz, 2H), 7.08 (d, *J* = 8.1 Hz, 2H), 6.68 (dd, *J* = 17.6, 10.9 Hz, 1H), 5.71 (dd, *J* = 17.6, 1.0 Hz, 1H), 5.22 (dd, *J* = 10.9, 0.9 Hz, 1H), 5.05 – 4.94 (br, 1H), 4.63 – 4.53 (br, 1H), 3.71 (s, 3H), 3.16 – 2.97 (m, 2H), 1.41 (s, 9H).

Spectroscopic data are consistent with those reported in the literature.[4]

4.1.5. Synthesis of bexarotene methyl ester, methyl 4-(1-(3,5,5,8,8-pentamethyl-5,6,7,8-tetrahydronaphthalen-2-yl)vinyl)benzoate 1s.

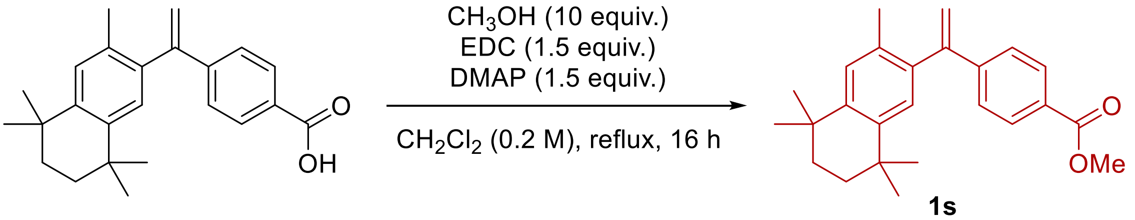

1s

Bexarotene (697.0 mg, 1.7 mmol) was dissolved in CH_2Cl_2 (8.5 mL, 0.2 M) and CH_3OH (17.0 mmol, 10 equiv.), *N*-(3-Dimethylaminopropyl)-*N*'-ethylcarbodiimide (2.5 mmol, 1.5 equiv.), 4-(dimethylamino)pyridine (0.3 mmol, 0.2 equiv.) were added. The mixture was stirred for 16 h at room temperature. The reaction crude was concentrated and the residue was purified by silica gel flash chromatography (95:5 Cyclohexane/EtOAc) to afford methyl 4-(1-(3,5,5,8,8-pentamethyl-5,6,7,8-tetrahydronaphthalen-2-yl)vinyl)benzoate **1s** (437.7 mg, 71%) as a white solid.

^{1}H NMR (300 MHz, $CDCl_3$) δ 8.01 – 7.91 (m, 2H), 7.38 – 7.32 (m, 2H), 7.10 (d, *J* = 14.5 Hz, 2H), 5.81 (d, *J* = 1.4 Hz, 1H), 5.32 (d, *J* = 1.4 Hz, 1H), 3.91 (s, 3H), 1.95 (s, 3H), 1.71 (s, 4H), 1.31 (s, 6H), 1.28 (s, 6H).

Spectroscopic data are consistent with those reported in the literature. [5]

4.2. General procedure B: Synthesis of 1,3-Diketones 2c, 2d, 2e, 2f, 2g and 2h

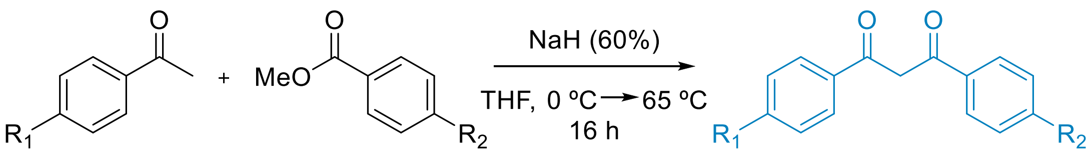

The corresponding acetophenone (3 mmol, 1 equiv.) was added dropwise to a dispersion of 60% NaH in mineral oil (9 mmol, 3 equiv.) in THF (8.4 mL) at 0 ºC and under nitrogen atmosphere. Then, a solution of the methyl benzoate derivative (3.3 mmol, 1.1 equiv.) in anhydrous THF (2.1 mL) was added dropwise over 15 min. The mixture was refluxed for 16 h under nitrogen and quenched with ice. Then a solution of HCl (0.1M) was added until a precipitate was formed. The solid was recovered by filtration and washed with distilled H_2O. The filtrate was extracted with AcOEt (3x10 mL); the organic phases were combined, washed with brine and dried over anhydrous $MgSO_4$. The solvent was removed under reduced pressure, and the product purified by flash chromatography with the eluent indicated in each case.

***1,3-di-p-tolylpropane-1,3-dione* (2c)**

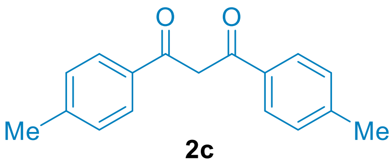

2c

Following the **general procedure B**, 4'-Methylacetophenone (402.5 mg, 3 mmol) and methyl 4-methylbenzoate (495.6 mg, 3.3 mmol) gave product **2c** (580.9 mg, 77%) as a white solid after purification (98:2 Cyclohexane/EtOAc).

^{1}H NMR (300 MHz, $CDCl_3$) δ 7.89 (d, J = 8.3 Hz, 4H), 7.29 (d, J = 7.9 Hz, 4H), 6.81 (s, 1H), 2.43 (s, 6H).

Spectroscopic data are consistent with those reported in the literature.[6]

***4,4'-malonyldibenzonitrile* (2d)**

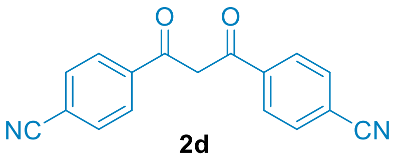

2d

Following the **general procedure B**, 1-(4-isocyanophenyl)ethan-1-one (435.5 mg, 3 mmol) and methyl 4-cyanobenzoate (531.8 mg, 3.3 mmol) gave product **2d** (354.9 mg, 43%) as a yellow solid after purification (80:20 Cyclohexane/EtOAc)

^{1}H NMR (300 MHz, $CDCl_3$) δ 8.08 (d, J = 8.8 Hz, 4H), 7.80 (d, J = 8.8 Hz, 4H), 6.86 (s, 1H).

Spectroscopic data are consistent with those reported in the literature.[6]

***1,3-bis(4-(trifluoromethyl)phenyl)propane-1,3-dione* (2e)**

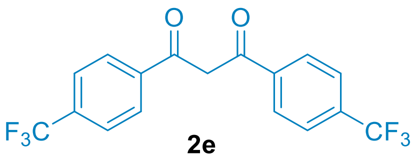

2e

Following the **general procedure B**, 4'-(Trifluoromethyl)acetophenone (564.5 mg, 3 mmol) and methyl 4-(trifluoromethyl)benzoate (673.7 mg, 3.3 mmol) gave product **2e** (418.23 mg, 39%) as a pale orange solid after purification (98:2 Cyclohexane/EtOAc)

^{1}H NMR (300 MHz, $CDCl_3$) δ 7.84 (d, J = 8.8 Hz, 4H), 7.63 (d, J = 8.8 Hz, 4H), 6.77 (s, 1H).

Spectroscopic data are consistent with those reported in the literature.[7]

***1,3-bis(4-bromophenyl)propane-1,3-dione* (2f)**

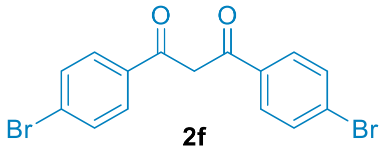
2f

Following the **general procedure B**, 4'-bromoacetophenone (597.1 mg, 3 mmol) and methyl 4-bromobenzoate (709.7 mg, 3.3 mmol) gave product **2f** (802.3 mg, 70%) as a white solid after purification (98:2 Cyclohexane/EtOAc)

^{1}H NMR (500 MHz, $CDCl_3$) δ 8.10 (d, J = 8.5 Hz, 4H), 7.77 (d, J = 8.5 Hz, 4H), 6.88 (s, 1H).

Spectroscopic data are consistent with those reported in the literature.[7]

***1-(4-methoxyphenyl)-3-phenylpropane-1,3-dione* (2g)**

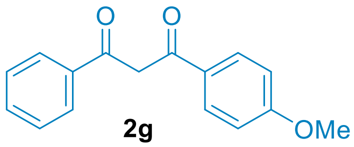
2g

Following the **general procedure B**, acetophenone (360.5 mg, 3 mmol) and methyl 4-methoxybenzoate (548.4 mg, 3.3 mmol) gave product **2g** (495.6 mg, 65%) as a white solid after purification (96:4 Cyclohexane/EtOAc)

^{1}H NMR (300 MHz, $CDCl_3$) δ 8.01 – 7.96 (m, 4H), 7.56 – 7.45 (m, 3H), 6.98 (d, J = 9.0 Hz, 1H), 6.80 (s, 1H), 3.89 (s, 3H).

Spectroscopic data are consistent with those reported in the literature.[8]

***1-(4-methoxyphenyl)-3-(4-(trifluoromethyl)phenyl)propane-1,3-dione* (2h)**

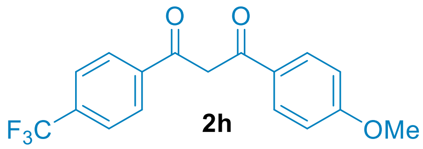
2h

Following the **general procedure B**, 4'-(Trifluoromethyl)acetophenone (564.5 mg, 3 mmol) and methyl 4-methoxybenzoate (548.4 mg, 3.3 mmol) gave product **2h** (369.9 mg, 38%) as a white solid after purification (96:4 Cyclohexane/EtOAc)

^{1}H NMR (300 MHz, $CDCl_3$) δ 8.06 (d, J = 8.1 Hz, 2H), 7.99 (d, J = 9.1 Hz, 2H), 7.73 (d, J = 8.1 Hz, 2H), 6.99 (d, J = 9.1 Hz, 2H), 6.80 (s, 1H), 3.89 (s, 3H).

Spectroscopic data are consistent with those reported in the literature.[8]

4.3. Synthesis of 2-benzoyl-2,3-dihydro-1H-inden-1-one 2k.

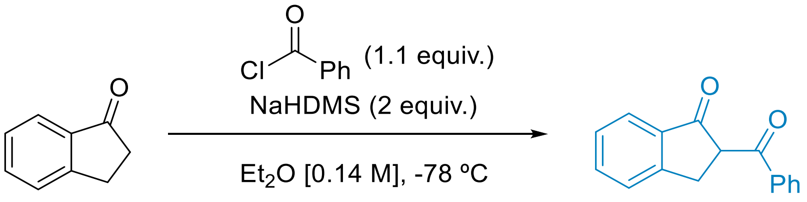

2k

Commercially available 1-indanone (500.0 mg, 3.8 mmol) was dissolved in Et_2O (30 mL) under argon atmosphere and treated with a solution of NaHDMS (3.8 mL of 2 M solution in THF, 7.57 mmol) at -78 °C. The reaction mixture was stirred for 30 minutes. Then, a solution of the acyl chloride (478 μL, 4.16 mmol) in Et_2O was added dropwise. After stirring for an additional 1.5 hours, the reaction was quenched with 15 mL of an aqueous NH_4Cl solution. The organic layer was extracted with Et_2O (3 x 10 mL). The combined organic layers were dried over $MgSO_4$, filtered, and concentrated to dryness**.** The crude product was purified by column chromatography on silica gel (97:3 Cyclohexane:EtOAc) to afford 2-benzoyl-2,3-dihydro-1H-inden-1-one **2k** (745 mg, 83%) as a brown solid.

^{1}H NMR (300 MHz, $CDCl_3$) δ 15.07 (s, 1H), 8.06 – 7.81 (m, 3H), 7.70 – 7.34 (m, 6H), 3.92 (s, 2H).

Spectroscopic data are consistent with those reported in the literature.[9]

4.4. General procedure C: Synthesis of 4-oxobenzoates

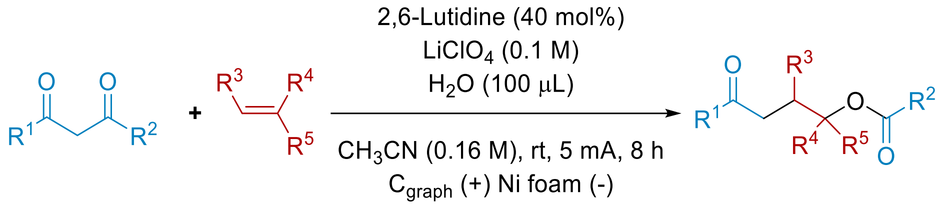

A 5 mL ElectraSyn vial with a stir bar was charged with a solution of the corresponding 1,3-diketone (0.5 mmol, 1.0 equiv.), lithium perchlorate (0.3 mmol, 0.1 M) and the corresponding olefin (1.0 mmol, 2 equiv.) in dry acetonitrile (3.0 mL, 0.16 M). Then, 2,6-lutidine (0.2 mmol, 40 mol%) and H_2O (100.0 μL) were added. The ElectraSyn vial was equipped with a graphite electrode (8 mm wide, 50 mm length, 1 mm thickness, anode), a nickel foam electrode (8 mm wide, 50 mm length, 1 mm thickness, cathode) and sealed. The reaction mixture was electrolyzed under galvanostatic conditions (5 mA, 8 h, 2.94 F/mol). After reaction completion, the ElectraSyn vial cap was removed and the electrodes were rinsed with acetone, which was combined with the crude mixture. The crude mixture was filtrated through Celite®, washed with acetone and the filtrate was concentrated under reduced pressure. The product was purified by flash column chromatography to give the corresponding 4-oxobenzoate.

4.5. Synthesis of 4-oxobenzoates under flow conditions

4.5.1. Optimization

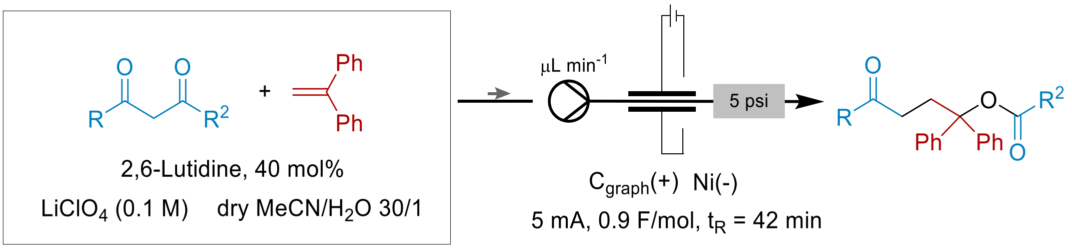

Table S1. Optimization of the flow electrochemical synthesis of 3

Entry	Deviation from optimized conditions	Yield (%)
**1**	**No deviation**	**63**
2	tR = 18 min, 0.4 F/mol	22
3	tR = 30 min, 0.7 F/mol	60
4	F = 200 μL/min	45

5	0.1 M; F = 200 µL/min	58
6	0.16 M	37
7	0.10 M	47

[a] Reaction conditions: **1** (0.14 mmol), **2** (0.28 mmol). 2,6-lutidine (40 mol %), $LiClO_4$ (0.1 M) and H_2O (31.0 µL) at constant current (5.0 mA) in dry acetone (0.055 M) at room temperature for 42 min of residence time, using C_{graph} as anode and Ni as cathode in a recirculating system.

4.5.2. General procedure D: Electro-flow synthesis of 4-oxobenzoates

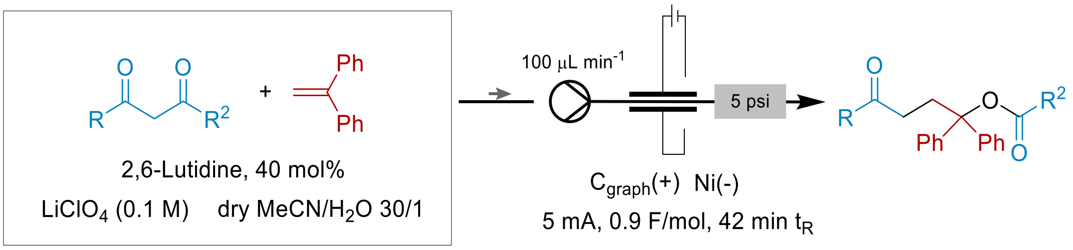

The corresponding 1,3-diketone (0.14 mmol), lithium perchlorate (0.26 mmol, 0.1 M) and the corresponding olefin (0.34 mmol, 2.0 equiv.) were dissolved in dry acetonitrile (2.72 mL, 0.055 M). Then, 2,6-lutidine (0.056 mmol, 40 mol%) and H_2O (31.5 µL) were added. The mixture was pumped through the electrochemical reactor of 0.6 mL volume at 100.0 µL min^{-1} under a constant current of 5 mA. The system pressure was controlled using a 5 psi back pressure regulator (BPR). The resulting solution was recirculated over a period of 196 min, with a total residence time of 42 minutes. The crude mixture was concentrated to dryness under reduced pressure and purified by flash column chromatography to afford the desired product.

<u>4-oxo-1,1,4-triphenylbutyl benzoate</u> (3a)

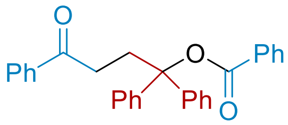

3a

Following the **general procedure C**, 1,3-diphenylpropane-1,3-dione **2a** (112.1 mg, 0.5 mmol) and diphenylethylene **1a** (177.0 µL, 1.0 mmol) gave product **3a** (203.9 mg, 97%) as a white solid after purification (95:5 Cyclohexane/EtOAc). Alternatively, following the **general procedure D**, 1,3-diphenylpropane-1,3-dione **2a** (31.4 mg, 0.14 mmol) and diphenylethylene **1a** (49.0 µL, 0.28 mmol) gave product **3a** (37.1 mg, 63%)

^{1}H NMR (500 MHz, $CDCl_3$) δ 8.07 (d, *J* = 7.5 Hz, 2H), 7.65 (d, *J* = 7.5 Hz, 2H), 7.49 (t, *J* = 7.4 Hz, 1H), 7.45 – 7.33 (m, 7H), 7.23 (td, *J* = 7.5, 4.8 Hz, 6H), 7.14 (t, *J* = 7.4 Hz, 2H), 3.34 – 3.28 (m, 2H), 2.83 – 2.77 (m, 2H).

^{13}C{^{1}H} NMR (125 MHz, $CDCl_3$) δ 99.2, 164.5, 144.5, 136.8, 133.2, 133.0, 131.3, 129.8, 128.7, 128.5, 128.5, 128.1, 127.4, 126.1, 86.8, 33.0, 32.0.

HRMS (ESI+): Calculated for $C_{22}H_{19}O$ [M - $C_7H_5O_2$]$^+$: 299.1436, found: 299.1428

<u>4-oxo-1,4-diphenylbutyl benzoate</u> (3b)

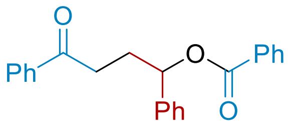

3b

Following the **general procedure C**, 1,3-diphenylpropane-1,3-dione **2a** (112.1 mg, 0.5 mmol) and styrene **1b** (115.0 µL, 1.0 mmol) gave product **3b** (139.4 mg, 81%) as a white solid after purification (95:5 Cyclohexane/EtOAc).

^{1}H NMR (300 MHz, $CDCl_3$) δ 8.15 – 8.07 (m, 2H), 7.95 – 7.89 (m, 2H), 7.61 – 7.28 (m, 11H), 6.17 (dd, *J* = 7.9, 5.6 Hz, 1H), 3.17 – 3.06 (m, 2H), 2.64 – 2.38 (m, 2H).

$^{13}C\{^1H\}$ **NMR** (75 MHz, $CDCl_3$) δ 198.9, 165.8, 140.3, 136.8, 133.1, 133.1, 130.3, 129.7, 128.7, 128.6, 128.5, 128.2, 128.0, 126.4, 76.0, 34.6, 31.0.

HRMS (ESI+): Calculated for $C_{23}H_{24}O_3N$ $[M + NH_4]^+$: 362.1750, found: 362.1751

***5-oxo-2,5-diphenylpentan-2-yl benzoate** (3c)*

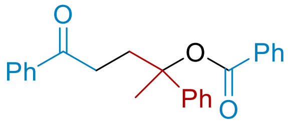

3c

Following the **general procedure C**, 1,3-diphenylpropane-1,3-dione **2a** (112.1 mg, 0.5 mmol) and α-methylstyrene **1c** (129.7 µL, 1.0 mmol) gave product **1c** (111.8 mg, 62%) as a white solid after purification (92:8 Cyclohexane/EtOAc).

^{1}**H NMR** (500 MHz, $CDCl_3$) δ 8.15 – 8.09 (m, 2H), 7.93 – 7.86 (m, 2H), 7.63 – 7.56 (m, 1H), 7.58 – 7.51 (m, 1H), 7.52 – 7.36 (m, 8H), 7.34 – 7.27 (m, 1H), 3.12 – 2.96 (m, 2H), 2.73 (ddd, *J* = 14.2, 10.3, 5.3 Hz, 1H), 2.61 (ddd, *J* = 14.2, 10.6, 5.3 Hz, 1H), 2.11 (s, 3H).

$^{13}C\{^1H\}$ **NMR** (125 MHz, $CDCl_3$) δ 199.3, 165.0, 144.2, 136.8, 133.1, 133.0, 131.3, 129.6, 128.6, 128.5, 128.5, 128.1, 127.3, 124.7, 84.2, 37.3, 33.4, 25.3.

HRMS (ESI+): Calculated for $C_{24}H_{26}O_3N$ $[M + NH_4]^+$: 376.1907, found 376.1901

***4-oxo-1,2,4-triphenylbutyl benzoate** (3d)*

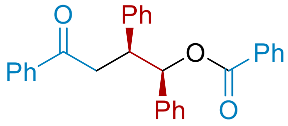

3d

Following the **general procedure C**, 1,3-diphenylpropane-1,3-dione **2a** (112.1 mg, 0.5 mmol) and *trans*-stilbene ***(E)*-1d** or *cis*-stilbene ***(Z)*-1d** (185.8 µL, 1.0 mmol) gave product **3d** (139.3 mg, 66% and 100.1 mg, 48% respectively) as a white solid after purification (95:5 Cyclohexane/EtOAc).

^{1}**H NMR** (300 MHz, $CDCl_3$) δ 7.94 – 7.89 (m, 2H), 7.77 – 7.72 (m, 2H), 7.44 – 7.33 (m, 2H), 7.32 – 7.20 (m, 5H), 7.12 – 7.01 (m, 9H), 6.13 (d, *J* = 8.6 Hz, 1H), 4.09 (ddd, *J* = 8.6, 7.9, 5.6 Hz, 1H), 3.57 – 3.36 (m, 2H).

$^{13}C\{^1H\}$ **NMR** (75 MHz, $CDCl_3$) δ 198.1, 165.7, 140.2, 138.8, 137.1, 133.1, 133.1, 130.1, 129.7, 128.8, 128.5, 128.4, 128.4, 128.2, 128.1, 128.0, 127.2, 127.0, 79.9, 47.1, 40.9.

HRMS (ESI+): Calculated for $C_{29}H_{25}O_3$ $[M + H]^+$: 421.1725, found: 421.1798

***5-oxo-2,3,5-triphenylpentan-2-yl benzoate** (3e)*

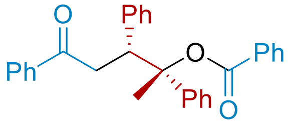

3e

Following the **general procedure C**, 1,3-diphenylpropane-1,3-dione **2a** (112.1 mg, 0.5 mmol) and (*E*)-alpha-methylstilbene **1e** (194.3 mg, 1.0 mmol) gave product **3e** (92.9 mg, 43%) as a white solid after purification (98:2 Cyclohexane/EtOAc).

^{1}**H NMR** (300 MHz, $CDCl_3$) δ 8.31 (d, *J* = 7.4 Hz, 2H), 7.97 (d, *J* = 7.4 Hz, 2H), 7.77 (t, *J* = 7.4 Hz, 1H), 7.71 – 7.62 (m, 3H), 7.55 (t, *J* = 7.4 Hz, 2H), 7.47 – 7.32 (m, 10H), 4.17 – 4.08 (m, 1H), 4.02 – 3.79 (m, 2H), 2.21 (s, 3H).

$^{13}C\{^1H\}$ **NMR** (125 MHz, $CDCl_3$) δ 198.2, 165.0, 143.2, 139.2, 137.1, 133.0, 133.0, 131.4, 129.9, 129.6, 128.6, 128.6, 128.2, 128.0, 127.9, 127.3, 127.0, 125.5, 86.8, 54.0, 39.6, 22.8.

HRMS (ESI+): Calculated for $C_{23}H_{21}O$ $[M - C_7H_5O_2]^+$: 313.1592, found 313.1594

***4-oxo-1,2,2,4-tetraphenylbutyl benzoate* (3f)**

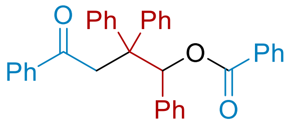

3f

Following the **general procedure C**, 1,3-diphenylpropane-1,3-dione **2a** (112.1 mg, 0.5 mmol) and triphenylethylene **1f** (256.3 mg, 1.0 mmol) gave product **3f** (62.1 mg, 25%) as a white solid after purification (95:5 Cyclohexane/EtOAc).

^{1}H NMR (500 MHz, $CDCl_3$) δ 7.97 – 7.92 (m, 2H), 7.81 – 7.77 (m, 2H), 7.55 – 7.45 (m, 4H), 7.43 – 7.33 (m, 7H), 7.31 – 7.27 (m, 5H), 7.08 – 6.98 (m, 3H), 6.89 – 6.83 (m, 2H), 5.43 (dd, *J* = 10.2, 3.1 Hz, 1H), 3.68 – 3.51 (m, 2H).

^{13}C{^{1}H} NMR (125 MHz, $CDCl_3$) δ 197.6, 164.8, 142.1, 140.7, 138.7, 137.2, 133.1, 132.9, 131.6, 130.5, 129.7, 128.8, 128.6, 128.5, 128.5, 128.1, 127.9, 127.7, 127.6, 127.5, 127.0, 90.0, 46.9, 41.7.

HRMS (ESI+): Calculated for $C_{28}H_{23}O$ $[M – C_7H_5O_2]^+$: 375.1749, found 375.1744

***2-(2-oxo-2-phenylethyl)-1-phenylcyclohexyl benzoate* (3h)**

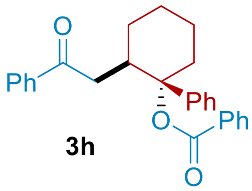

3h

Following the **general procedure C**, 1,3-diphenylpropane-1,3-dione **2a** (112.1 mg, 0.5 mmol) and 1-phenyl-1-cyclohexene **1h** (158.2 mg, 1.0 mmol) gave product **3h** (160.4 mg, 81%) as a white solid after purification (95:5 Cyclohexane/EtOAc). Alternatively, following the **general procedure D**, 1,3-diphenylpropane-1,3-dione **2a** (31.4 mg, 0.14 mmol) and 1-phenyl-1-cyclohexene **1h** (44.6 µL, 0.28 mmol) gave product **3h** (12.6 mg, 23%).

^{1}H NMR (500 MHz, $CDCl_3$) δ 8.24 – 8.21 (m, 2H), 7.68 – 7.63 (m, 3H), 7.57 – 7.53 (m, 2H), 7.52 – 7.49 (m, 1H), 7.40 – 7.30 (m, 6H), 7.29 – 7.27 (m, 1H), 3.29 (m, 1H), 3.07 (dd, *J* = 16.1, 2.8 Hz, 1H), 2.94 (dd, *J* = 16.1, 9.8 Hz, 1H), 2.43 (ddt, *J* = 11.6, 9.8, 3.4 Hz, 1H), 2.20 (ddd, *J* = 14.3, 12.9, 3.8 Hz, 1H), 1.95 – 1.91 (m, 1H), 1.86 – 1.70 (m, 3H), 1.63 – 1.47 (m, 2H).

^{13}C{^{1}H} NMR (125 MHz, $CDCl_3$) δ 199.5, 164.7, 142.0, 136.9, 133.1, 132.9, 131.3, 129.7, 128.7, 128.5, 128.3, 128.1, 127.2, 125.4, 87.3, 45.4, 39.9, 34.1, 28.9, 25.7, 21.7.

HRMS (ESI+): Calculated for $C_{20}H_{21}O$ $[M - C_7H_5O_2]^+$: 277.1592, found 277.1601

***(E)-6-oxo-1,6-diphenylhex-1-en-3-yl benzoate* (3i)**

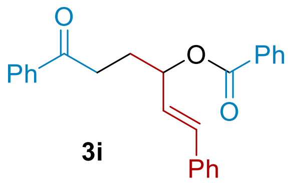

3i

Following the **general procedure C**, 1,3-diphenylpropane-1,3-dione **2a** (112.1 mg, 0.5 mmol) and (1*E*)-buta-1,3-dienyl]benzene **1i** (130.2 mg, 1.0 mmol) gave product **3i** (130.6 mg, 71%) as a yellow solid after purification (95:5 Cyclohexane/EtOAc).

^{1}H NMR (300 MHz, $CDCl_3$) δ 8.14 – 8.09 (m, 2H), 7.98 – 7.93 (m, 2H), 7.62 – 7.51 (m, 2H), 7.50 – 7.39 (m, 6H), 7.36 – 7.23 (m, 3H), 6.78 (dd, *J* = 16.0, 1.0 Hz, 1H), 6.32 (dd, *J* = 16.0, 6.8 Hz, 1H), 5.89 – 5.79 (m, 1H), 3.17 (dd, *J* = 8.2, 6.8 Hz, 2H), 2.46 – 2.33 (m, 2H).

^{13}C{^{1}H} NMR (75 MHz, $CDCl_3$) δ 199.0, 165.8, 136.8, 136.2, 133.1, 133.1, 133.1, 130.4, 129.7, 128.6, 128.4, 128.1, 128.1, 126.7, 74.7, 34.3, 29.1.

HRMS (ESI+): Calculated for $C_{18}H_{17}O$ $[M - C_7H_5O_2]^+$: 249.1279, found 249.1275

***4-oxo-4-phenyl-1,1-di-p-tolylbutyl benzoate* (3j)**

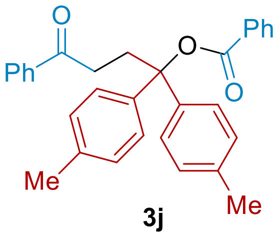

3j

Following the **general procedure C**, 1,3-diphenylpropane-1,3-dione **2a** (112.1 mg, 0.5 mmol) and 4,4'-(ethene-1,1-diyl)bis(methylbenzene) **1j** (208.0 mg, 1.0 mmol) gave product **3j** (92.9 mg, 43%) as a white solid after purification (95:5 Cyclohexane/EtOAc).

^{1}H NMR (300 MHz, $CDCl_3$) δ 8.19 – 8.13 (m, 2H), 7.79 – 7.74 (m, 2H), 7.64 – 7.57 (m, 1H), 7.53 – 7.45 (m, 3H), 7.42 – 7.33 (m, 6H), 7.17 – 7.12 (m, 4H), 3.40 – 3.32 (m, 2H), 2.93 – 2.85 (m, 2H), 2.32 (s, 6H).

^{13}C{^{1}H} NMR (75 MHz, $CDCl_3$) δ 199.4, 164.6, 137.0, 136.9, 133.1, 133.0, 131.5, 129.8, 129.2, 128.7, 128.6, 128.1, 126.0, 86.9, 33.2, 32.1, 21.2.

HRMS (ESI+): Calculated for $C_{24}H_{23}O$ [M – $C_7H_5O_2$]$^{+}$: 327.1749, found 327.1747

<u>4-oxo-4-phenyl-1,1-bis(4-(trifluoromethyl)phenyl)butyl benzoate</u> (3k)

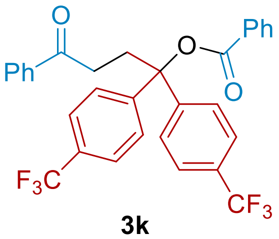

3k

Following the **general procedure C**, 1,3-diphenylpropane-1,3-dione **2a** (112.1 mg, 0.5 mmol) and 4,4'-(ethene-1,1-diyl)bis((trifluoromethyl)benzene) **1k** (316.2 mg, 1.0 mmol) gave product **3k** (138.0 mg, 50%) as a white solid after purification (90:10 Cyclohexane/EtOAc).

^{1}H NMR (500 MHz, $CDCl_3$) δ 8.18 (d, *J* = 6.9 Hz, 2H), 7.78 (d, *J* = 7.0 Hz, 2H), 7.71 – 7.62 (m, 9H), 7.56 (t, *J* = 7.8 Hz, 2H), 7.51 (t, *J* = 7.4 Hz, 1H), 7.38 (t, *J* = 7.8 Hz, 2H), 3.53 – 3.43 (m, 2H), 2.96 – 2.89 (m, 2H).

^{13}C{^{1}H} NMR (125 MHz, $CDCl_3$) δ 198.4, 164.3, 147.6, 136.6, 133.8, 133.3, 130.5, 130.0 (q, *J* = 33 Hz), 129.8, 128.9, 128.7, 128.0, 126.4, 125.8 (q, *J* = 3.7 Hz), 124.0 (q, *J* = 272 Hz), 85.8, 32.5, 31.6.

^{19}F{^{1}H} NMR (470 MHz, $CDCl_3$) δ -62.61.

HRMS (ESI+): Calculated for $C_{31}H_{26}F_6O_3N$ [M + NH_4]$^{+}$: 574.1811, found 574.1885

<u>1,1-bis(4-bromophenyl)-4-oxo-4-phenylbutyl benzoate</u> (3l)

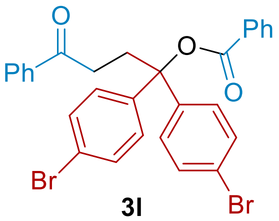

3l

Following the **general procedure C**, 1,3-diphenylpropane-1,3-dione **2a** (112.1 mg, 0.5 mmol) and 4,4'-(ethene-1,1-diyl)bis(bromobenzene) **1l** (338.04 mg, 1.0 mmol) gave product **3l** (187.4 mg, 65%) as a white solid after purification (95:5 Cyclohexane/EtOAc).

^{1}H NMR (500 MHz, $CDCl_3$) δ 8.18 (dd, *J* = 8.2, 1.5 Hz, 2H), 7.80 (dd, *J* = 8.2, 1.5 Hz, 2H), 7.68 – 7.61 (m, 1H), 7.57 – 7.47 (m, 7H), 7.47 – 7.34 (m, 6H), 3.44 – 3.37 (m, 2H), 2.97 – 2.90 (m, 2H).

^{13}C{^{1}H} NMR (125 MHz, $CDCl_3$) δ 198.5, 164.2, 143.0, 136.5, 133.5, 133.1, 131.7, 130.6, 129.6, 128.7, 128.5, 127.9, 127.8, 121.7, 85.8, 32.6, 31.6.

HRMS (ESI+): Calculated for $C_{22}H_{17}Br_2O$ [M – $C_7H_5O_2$]$^{+}$: 454.9646, found 454.9641

<u>1,1-bis(4-fluorophenyl)-4-oxo-4-phenylbutyl benzoate</u> (3m)

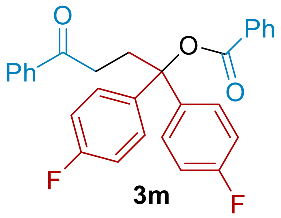

3m

Following the **general procedure C**, 1,3-diphenylpropane-1,3-dione **2a** (112.1 mg, 0.5 mmol) and 4,4'-(ethene-1,1-diyl)bis(fluorobenzene) **1m** (216.2 mg, 1.0 mmol) gave product **3m** (216.2 mg, 65%) as a white solid after purification (95:5 Cyclohexane/EtOAc).

^{1}H NMR (500 MHz, $CDCl_3$) δ 8.21 – 8.16 (m, 2H), 7.83 – 7.79 (m, 2H), 7.67 – 7.62 (m, 1H), 7.56 – 7.49 (m, 7H), 7.41 – 7.36 (m, 2H), 7.11 – 7.04 (m, 4H), 3.45 – 3.39 (m, 2H), 2.97 – 2.91 (m, 2H).

^{13}C{^{1}H} NMR (125 MHz, $CDCl_3$) δ 198.8, 164.4, 161.9 (d, *J* = 247 Hz), 140.1 (d, *J* = 3.2 Hz), 136.7, 133.4, 133.1, 130.9, 129.4, 128.7, 128.6, 128.0, 127.9 (d, *J* = 8 Hz), 115.4 (d, *J* = 21 Hz), 86.0, 32.8, 32.1.

^{19}F{^{1}H} NMR (470 MHz, $CDCl_3$) δ -114.54.

HRMS (ESI+): Calculated for $C_{22}H_{17}F_2O$ $[M – C_7H_5O_2]^+$: 335.1247, found 335.1242

***<u>4-oxo-4-phenyl-1-(pyridin-2-yl)butyl benzoate</u>* (3n)**

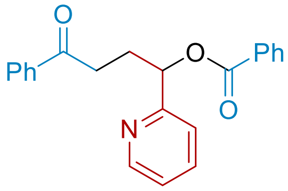

3n

Following the **general procedure C**, 1,3-diphenylpropane-1,3-dione **2a** (112.1 mg, 0.5 mmol) and 2-vinylpyridine **1n** (107.8 µL, 1.0 mmol) gave product **3n** (81.0 mg, 47%) as a yellow solid after purification (85:15 Cyclohexane/EtOAc).

^{1}H NMR (500 MHz, $CDCl_3$) δ 8.59 (ddd, *J* = 4.9, 1.8, 0.9 Hz, 1H), 8.12 – 8.08 (m, 2H), 7.93 – 7.89 (m, 2H), 7.67 (td, *J* = 7.6, 1.8 Hz, 1H), 7.58 – 7.48 (m, 2H), 7.46 – 7.38 (m, 5H), 7.19 (ddd, *J* = 7.6, 4.9, 1.2 Hz, 1H), 6.18 (dd, *J* = 7.2, 6.0 Hz, 1H), 3.19 – 3.12 (m, 2H), 2.59 (dtd, *J* = 8.4, 6.6, 1.7 Hz, 2H).

^{13}C{^{1}H} NMR (125 MHz, $CDCl_3$) δ 199.0, 165.9, 159.3, 149.5, 136.9, 136.8, 133.3, 133.1, 130.0, 129.8, 128.6, 128.5, 128.1, 122.9, 120.8, 76.6, 34.5, 29.5.

HRMS (ESI+): Calculated for $C_{15}H_{14}NO$ $[M – C_7H_5O_2]^+$: 224.1075, found 224.1071

***<u>1-(4-methoxyphenyl)-4-oxo-4-phenylbutyl benzoate</u>* (3o)**

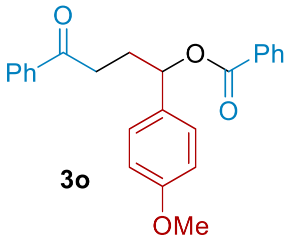

3o

Following the **general procedure C**, 1,3-diphenylpropane-1,3-dione **2a** (112.1 mg, 0.5 mmol) and 4-methoxystyrene **1o** (135.5 µL, 1.0 mmol) gave product **3o** (81.0 mg, 47%) as a white solid after purification (92:8 Cyclohexane/EtOAc). Alternatively, following the **general procedure D**, 1,3-diphenylpropane-1,3-dione **2a** (31.4 mg, 0.14 mmol) and 4-methoxystyrene **1o** (37.5 µL, 0.28 mmol) gave product **3o** (31.7 mg, 62%).

^{1}H NMR (300 MHz, $CDCl_3$) δ 8.11 – 8.06 (m, 2H), 7.94 – 7.89 (m, 2H), 7.58 – 7.49 (m, 2H), 7.47 – 7.37 (m, 6H), 6.94 – 6.89 (m, 2H), 6.12 (dd, *J* = 7.9, 5.9 Hz, 1H), 3.78 (s, 3H), 3.18 – 2.98 (m, 2H), 2.63 – 2.36 (m, 2H).

^{13}C{^{1}H} NMR (75 MHz, $CDCl_3$) δ 198.9, 165.8, 159.5, 136.8, 133.1, 133.0, 132.3, 130.4, 129.6, 128.6, 128.4, 128.0, 127.9, 114.0, 75.7, 55.2, 34.6, 30.7.

HRMS (ESI+): Calculated for $C_{17}H_{17}O_2$ $[M - C_7H_5O_2]^+$: 253.1229, found 253.1240

***1-(4-cyanophenyl)-4-oxo-4-phenylbutyl benzoate* (3p)**

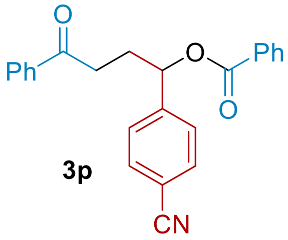

3p

Following the **general procedure C**, 1,3-diphenylpropane-1,3-dione **2a** (112.1 mg, 0.5 mmol) and 4-cyanostyrene **1p** (129.2 µL, 1.0 mmol) gave product **3p** (81.0 mg, 47%) as a yellow solid after purification (85:15 Cyclohexane/EtOAc).

^{1}H NMR (500 MHz, $CDCl_3$) δ 8.09 – 8.05 (m, 2H), 7.93 – 7.89 (m, 2H), 7.67 – 7.64 (m, 2H), 7.60 – 7.52 (m, 4H), 7.48 – 7.41 (m, 4H), 6.12 (dd, *J* = 8.5, 4.9 Hz, 1H), 3.15 – 3.11 (m, 2H), 2.53 – 2.38 (m, 2H).

^{13}C{^{1}H} NMR (125 MHz, $CDCl_3$) δ 198.5, 165.7, 145.8, 136.6, 133.5, 133.4, 132.6, 129.7, 129.6, 128.7, 128.6, 128.0, 127.0, 118.6, 112.0, 75.1, 34.3, 30.8.

HRMS (ESI+): Calculated for $C_{17}H_{14}NO$ $[M - C_7H_5O_2]^+$: 248.1075, found 248.1079

***1-(2-bromophenyl)-4-oxo-4-phenylbutyl benzoate* (3q)**

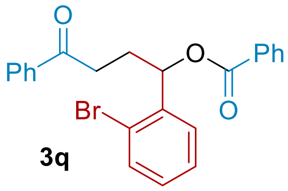

3q

Following the **general procedure C**, 1,3-diphenylpropane-1,3-dione **2a** (112.1 mg, 0.5 mmol) and 2-bromostyrene **1q** (125.4 µL, 1.0 mmol) gave product **3q** (114.9 mg, 54%) as a white solid after purification (92:8 Cyclohexane/EtOAc). Alternatively, following the **general procedure D**, 1,3-diphenylpropane-1,3-dione **2a** (31.4 mg, 0.14 mmol) and 2-bromostyrene **1q** (35.1 µL, 0.28 mmol) gave product **3q** (19.6 mg, 33%).

^{1}H NMR (500 MHz, $CDCl_3$) δ 8.13 – 8.09 (m, 2H), 7.96 – 7.92 (m, 2H), 7.60 – 7.51 (m, 4H), 7.48 – 7.40 (m, 4H), 7.32 (td, *J* = 7.6, 1.3 Hz, 1H), 7.17 – 7.13 (m, 1H), 6.46 (dd, *J* = 8.0, 5.3 Hz, 1H), 3.27 – 3.11 (m, 2H), 2.55 – 2.42 (m, 2H).

^{13}C{^{1}H} NMR (125 MHz, $CDCl_3$) δ 198.8, 165.5, 140.0, 136.7, 133.3, 133.2, 133.0, 130.0, 129.7, 129.4, 128.6, 128.5, 128.1, 127.9, 127.2, 122.2, 75.0, 34.6, 30.1.

HRMS (ESI+): Calculated for $C_{23}H_{23}BrO_3N$ $[M + NH_4]^+$: 440.0856 , found 440.0855

***4-(4-methoxyphenyl)-4-oxo-1,1-diphenylbutyl 4-methoxybenzoate* (3r)**

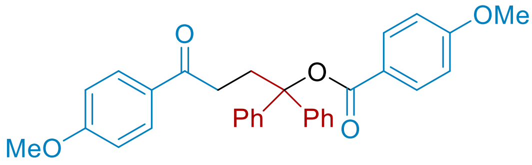

3r

Following the **general procedure C**, 1,3-bis(4-methoxyphenyl)propane-1,3-dione **2b** (142.2 mg, 0.5 mmol) and diphenylethylene **1a** (177.0 µL, 1.0 mmol) gave product **3r** (174.9 mg, 73%) as a yellow solid after purification (80:20 Cyclohexane/EtOAc). Alternatively, following the **general procedure D**, 1,3-bis(4-methoxyphenyl)propane-1,3-dione **2b** (39.8 mg, 0.14 mmol) and diphenylethylene **1a** (60.0 µL, 0.28 mmol) gave product **3r** (43.5 mg, 65%).

^{1}H NMR (300 MHz, $CDCl_3$) δ 8.21 – 8.12 (m, 2H), 7.82 – 7.73 (m, 2H), 7.60 – 7.51 (m, 4H), 7.41 – 7.33 (m, 4H), 7.30 – 7.23 (m, 2H), 7.05 – 6.98 (m, 2H), 6.88 – 6.81 (m, 2H), 3.90 (s, 3H), 3.83 (s, 3H), 3.46 – 3.36 (m, 2H), 2.93 – 2.82 (m, 2H).

^{13}C{^{1}H} NMR (75 MHz, $CDCl_3$) δ 197.9, 164.2, 163.6, 163.4, 144.7, 131.8, 130.3, 130.0, 128.4, 127.3, 126.1, 123.7, 113.9, 113.7, 86.4, 55.6, 55.5, 32.7, 32.4.

HRMS (ESI+): Calculated for $C_{23}H_{21}O_2$ $[M - C_8H_7O_3]^+$ 329.1536:, found: 329.1593

***4-oxo-1,1-diphenyl-4-(p-tolyl)butyl 4-methylbenzoate* (3s)**

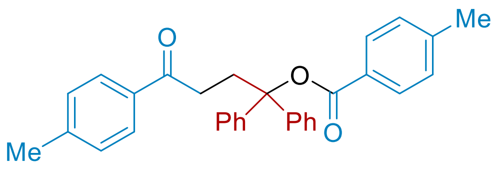

3s

Following the **general procedure C**, 1,3-di-p-tolylpropane-1,3-dione **2c** (126.2 mg, 0.5 mmol) and diphenylethylene **1a** (177.0 µL, 1.0 mmol) gave product **3s** (189.3 mg, 84%) as a white solid after purification (95:5 Cyclohexane/EtOAc).

^{1}H NMR (300 MHz, $CDCl_3$) δ 8.17 – 8.10 (m, 2H), 7.75 – 7.69 (m, 2H), 7.63 – 7.56 (m, 4H), 7.45 – 7.26 (m, 8H), 7.20 (d, *J* = 7.8 Hz, 2H), 3.51 – 3.39 (m, 2H), 2.99 – 2.88 (m, 2H), 2.50 (s, 3H), 2.40 (s, 3H).

^{13}C{^{1}H} NMR (75 MHz, $CDCl_3$) δ 198.9, 164.6, 144.6, 143.9, 143.7, 134.4, 129.8, 129.4, 129.2, 128.6, 128.5, 128.2, 126.1, 86.6, 32.9, 32.2, 21.8, 21.7.

HRMS (ESI+): Calculated for $C_{23}H_{21}O$ [M - $C_8H_7O_2$]$^+$: 313.1592, found: 313.1587

***4-(4-cyanophenyl)-4-oxo-1,1-diphenylbutyl 4-cyanobenzoate* (3t)**

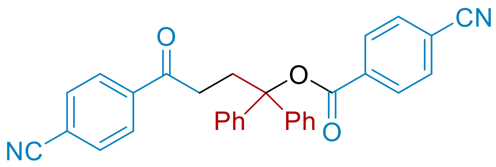

3t

Following the **general procedure C**, 4,4'-malonyldibenzonitrile **2d** (137.1 mg, 0.5 mmol) and diphenylethylene **1a** (177.0 µL, 1.0 mmol) gave product **3t** (197.4 mg, 84%) as a colorless solid after purification (85:15 Cyclohexane/EtOAc). Alternatively, following the **general procedure D**, 4,4'-malonyldibenzonitrile **2d** (38.4 mg, 0.14 mmol) and diphenylethylene **1a** (60.0 µL, 0.28 mmol) gave product **3t** (32.4 mg, 49%).

^{1}H NMR (300 MHz, $CDCl_3$) δ 8.26 – 8.19 (m, 2H), 7.83 – 7.77 (m, 4H), 7.69 – 7.63 (m, 2H), 7.51 – 7.45 (m, 4H), 7.41 – 7.33 (m, 4H), 7.32 – 7.27 (m, 2H), 3.47 – 3.35 (m, 2H), 2.94 – 2.83 (m, 2H).

^{13}C{^{1}H} NMR (75 MHz, $CDCl_3$) δ 197.5, 163.0, 143.6, 139.6, 134.9, 132.6, 132.5, 130.2, 128.7, 128.5, 127.9, 126.1, 118.0, 117.9, 116.8, 116.5, 87.9, 33.4, 31.7.

HRMS (ESI+): Calculated for $C_{23}H_{18}NO$ [M - $C_8H_4NO_2$]$^+$: 324.1388, found: 324.1383

***4-oxo-1,1-diphenyl-4-(4-(trifluoromethyl)phenyl)butyl 4-(trifluoromethyl)benzoate* (3u)**

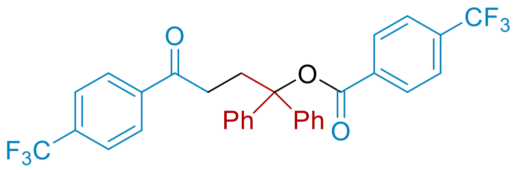

3u

Following the **general procedure C**, 1,3-bis(4-(trifluoromethyl)phenyl)propane-1,3-dione **2e** (180.1 mg, 0.5 mmol) and diphenylethylene **1a** (177.0 µL, 1.0 mmol) gave product **3u** (204.5 mg, 73%) as a yellow solid after purification (95:5 Cyclohexane/EtOAc).

^{1}H NMR (300 MHz, $CDCl_3$) δ 8.30 (d, *J* = 8.0 Hz, 2H), 7.86 (d, *J* = 8.0 Hz, 2H), 7.79 (d, *J* = 8.1 Hz, 2H), 7.64 (d, *J* = 8.1 Hz, 2H), 7.59 – 7.53 (m, 4H), 7.44 – 7.37 (m, 4H), 7.35 – 7.28 (m, 2H), 3.54 – 3.43 (m, 2H), 3.01 – 2.91 (m, 2H).

^{13}C{^{1}H} NMR (75 MHz, $CDCl_3$) 198.0, 163.4, 143.9, 139.4, 134.8 (q, *J* = 33 Hz), 134.4 (q, *J* = 33 Hz), 134.4, 130.1, 128.7, 128.4, 127.8, 126.1, 123.7 (q, *J* = 273 Hz), 123.7 (q, *J* = 273 Hz), 87.6, 33.3, 31.9.

^{19}F{^{1}H} NMR (282 MHz, $CDCl_3$) δ -63.07, -63.12.

HRMS (ESI+): Calculated for $C_{24}H_{18}F_3O$ [M – $C_8H_4F_3O_2$]$^+$: 367.1310, found: 368.1318

***4-(4-Bromophenyl)-4-oxo-1,1-diphenylbutyl 4-bromobenzoate* (3v)**

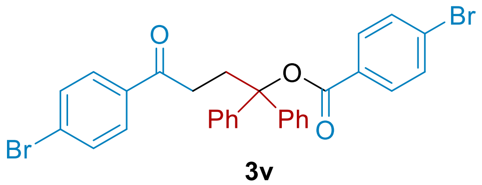

3v

Following the **general procedure C**, 1,3-bis(4-bromophenyl)propane-1,3-dione **2f** (191.2 mg, 0.5 mmol) and diphenylethylene **1a** (177.0 µL, 1.0 mmol) gave product **3v** (186.4 mg, 64%) as a white solid after purification (98:2 Cyclohexane/EtOAc). Alternatively, following the **general procedure D**, 1,3-bis(4-bromophenyl)propane-1,3-dione **2f** (53.5 mg, 0.14 mmol) and diphenylethylene **1a** (60.0 µL, 0.28 mmol) gave product **3v** (24.9 mg, 37%).

^{1}H NMR (300 MHz, $CDCl_3$) δ 7.89 (d, *J* = 8.6 Hz, 2H), 7.55 – 7.44 (m, 4H), 7.43 – 7.32 (m, 6H), 7.29 – 7.19 (m, 4H), 7.14 (t, *J* = 7.1 Hz, 2H), 3.34 – 3.22 (m, 2H), 2.81 – 2.66 (m, 2H).

^{13}C{^{1}H} NMR (75 MHz, $CDCl_3$) δ 198.0, 163.8, 144.1, 135.4, 132.0, 131.8, 131.2, 130.0, 129.6, 128.5, 128.4, 128.2, 127.6, 126.0, 87.1, 32.9, 32.0.

HRMS (ESI+): Calculated for $C_{22}H_{18}BrO$ [M - $C_7H_4BrO_2$]$^+$: 377.0541, found: 377.0536

***4-(4-methoxyphenyl)-4-oxo-1,1-diphenylbutyl benzoate* (3w)**

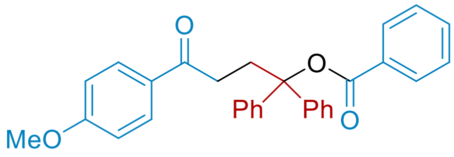

3w

Following the **general procedure C**, 1-(4-methoxyphenyl)-3-phenylpropane-1,3-dione **2g** (127.1 mg, 0.5 mmol) and diphenylethylene **1a** (177.0 µL, 1.0 mmol) gave product **3w** (96.9 mg, 43%) as a white solid after purification (95:5 Cyclohexane/EtOAc).

^{1}H NMR (300 MHz, $CDCl_3$) δ 8.19 – 8.14 (m, 2H), 7.76 – 7.71 (m, 2H), 7.65 – 7.58 (m, 1H), 7.55 – 7.46 (m, 6H), 7.34 (tt, *J* = 6.8, 0.9 Hz, 4H), 7.29 – 7.21 (m, 2H), 6.86 – 6.79 (m, 2H), 3.82 (s, 3H), 3.43 – 3.33 (m, 2H), 2.88 – 2.78 (m, 2H).

^{13}C{^{1}H} NMR (75 MHz, $CDCl_3$) δ 197.9, 164.5, 163.5, 144.6, 133.2, 131.4, 130.4, 130.0, 129.8, 128.7, 128.5, 127.4, 126.1, 113.7, 86.9, 55.5, 32.7, 32.3.

HRMS (ESI+): Calculated for $C_{23}H_{21}O_2$ [M – $C_7H_5O_2$]$^+$: 329.1542, found: 329.1548

***4-oxo-1,1,4-triphenylbutyl 4-methoxybenzoate* (3w')**

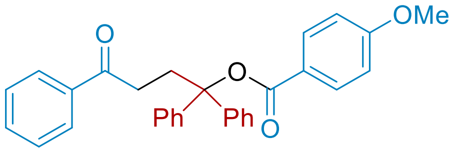

3w'

Following the **general procedure C**, 1-(4-methoxyphenyl)-3-phenylpropane-1,3-dione **2g** (127.1mg, 0.5 mmol) and diphenylethylene **1a** (177.0 µL, 1.0 mmol) gave product **3w'** (92.3 mg, 41%) as a white solid after purification (95:5 Cyclohexane/EtOAc).

^{1}H NMR (300 MHz, $CDCl_3$) δ 8.17 – 8.07 (m, 2H), 7.79 – 7.71 (m, 2H), 7.55 – 7.44 (m, 5H), 7.39 – 7.30 (m, 6H), 7.29 – 7.20 (m, 2H), 7.03 – 6.94 (m, 2H), 3.89 (s, 3H), 3.43 – 3.34 (m, 2H), 2.93 – 2.84 (m, 2H).

^{13}C{^{1}H} NMR (75 MHz, $CDCl_3$) δ 199.4, 164.3, 163.7, 144.7, 136.9, 133.0, 131.9, 128.6, 128.5, 128.1, 127.4, 126.1, 123.7, 114.0, 86.4, 55.6, 33.1, 32.2.

HRMS (ESI+): Calculated for $C_{22}H_{19}O$ [M – $C_8H_7O_3$]$^+$: 299.1436, found: 299.1434

4-(4-methoxyphenyl)-4-oxo-1,1-diphenylbutyl 4-(trifluoromethyl)benzoate ***(3x)***

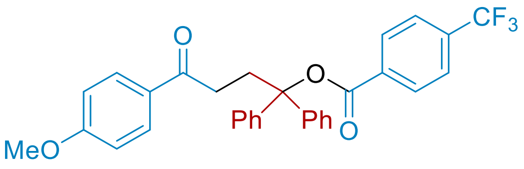

3x

Following the **general procedure C**, 1-(4-methoxyphenyl)-3-(4-(trifluoromethyl)phenyl)propane-1,3-dione **2h** (161.1 mg, 0.5 mmol) and diphenylethylene **1a** (177.0 µL, 1.0 mmol) gave product **3x** (72.6 mg, 28%) as a white solid after purification (94:6 Cyclohexane/EtOAc).

^{1}H NMR (500 MHz, $CDCl_3$) δ 8.25 (d, *J* = 8.0 Hz, 2H), 7.76 (d, *J* = 8.0 Hz, 2H), 7.74 – 7.70 (m, 2H), 7.52 – 7.48 (m, 4H), 7.36 (dd, *J* = 8.5, 7.0 Hz, 4H), 7.29 – 7.25 (m, 2H), 6.84 – 6.81 (m, 2H), 3.82 (s, 3H), 3.41 – 3.34 (m, 2H), 2.86 – 2.78 (m, 2H).

^{13}C{^{1}H} NMR (125 MHz, $CDCl_3$) δ 197.6, 163.5, 163.3, 134.7 (q, *J* = 33 Hz), 130.4, 130.2, 129.9, 128.6, 127.7, 126.1, 125.8 (q, *J* = 3.7 Hz), 123.8 (q, *J* = 273 Hz), 113.7, 87.8, 55.6, 32.6, 32.2.

^{19}F{^{1}H} NMR (282 MHz, $CDCl_3$) δ -63.09.

HRMS (ESI+): Calculated for $C_8H_4F_3O_2$ [M - $C_{23}H_{21}O_2$]$^+$: 329.1542, found: 329.1543

4-oxo-1,1-diphenyl-4-(4-(trifluoromethyl)phenyl)butyl 4-methoxybenzoate ***(3x')***

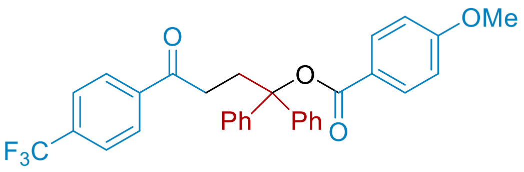

3x'

Following the **general procedure C**, 1-(4-methoxyphenyl)-3-(4-(trifluoromethyl)phenyl)propane-1,3-dione **2h** (161.1 mg, 0.5 mmol) and diphenylethylene **2a** (177.0 µL, 1.0 mmol) gave product **3x'** (72.6 mg, 28%) as a white solid after purification (94:6 Cyclohexane/EtOAc).

^{1}H NMR (500 MHz, $CDCl_3$) δ 8.14 – 8.10 (m, 2H), 7.84 (d, *J* = 8.2 Hz, 2H), 7.62 (d, *J* = 8.2 Hz, 2H), 7.53 – 7.49 (m, 4H), 7.37 – 7.33 (m, 4H), 7.28 – 7.24 (m, 2H), 7.01 – 6.96 (m, 2H), 3.89 (s, 3H), 3.42 – 3.38 (m, 2H), 2.94 – 2.88 (m, 2H).

^{13}C{^{1}H} NMR (125 MHz, $CDCl_3$) δ 198.4, 164.4, 163.8, 144.6, 134.3 (q, *J* = 32.6 Hz), 131.9, 128.6, 128.5, 127.5, 126.0, 125.7 (q, *J* = 4 Hz), 123.7 (q, *J* = 273 Hz),123.5, 114.0, 86.3, 55.6, 33.4, 32.1.

^{19}F{^{1}H} NMR (282 MHz, $CDCl_3$) δ -63.12.

HRMS (ESI+): Calculated for $C_{23}H_{19}F_3O$ [M – $C_6H_7O_3$]$^+$: 367.1310, found: 367.1302

4-oxo-1,1-diphenyl-4-(pyridin-2-yl)butyl picolinate ***(3y)***

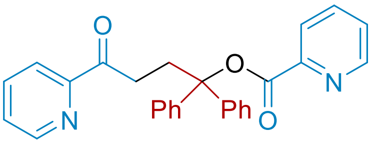

3y

Following the **general procedure C**, 1,3-di(pyridin-2-yl)propane-1,3-dione **2i** (113.1 mg, 0.5 mmol) and diphenylethylene **1a** (177.0 µL, 1.0 mmol) gave product **3y** (44.5 mg, 21%) as a brown solid after purification (50:50 Cyclohexane/EtOAc).

^{1}H NMR (500 MHz, $CDCl_3$) δ 8.80 (ddd, *J* = 4.9, 1.8, 0.9 Hz, 1H), 8.49 (ddd, *J* = 4.9, 1.8, 0.9 Hz, 1H), 8.16 (dt, *J* = 7.9, 1.1 Hz, 1H), 7.90 (dt, *J* = 7.9, 1.1 Hz, 1H), 7.84 (td, *J* = 7.7, 1.8 Hz, 1H), 7.72 (td, *J* = 7.7, 1.8 Hz, 1H), 7.62 – 7.56 (m, 4H), 7.47 (ddd, *J* = 7.6, 4.7, 1.2 Hz, 1H), 7.38 – 7.28 (m, 5H), 7.26 – 7.19 (m, 2H), 3.47 – 3.40 (m, 2H), 3.22 – 3.16 (m, 2H).

^{13}C{^{1}H} NMR (125 MHz, $CDCl_3$) δ 201.0, 163.2, 153.2, 150.1, 149.2, 148.9, 144.4, 136.9, 136.8, 128.4, 127.3, 127.0, 126.8, 126.3, 125.1, 121.7, 87.5, 32.4, 31.3.

HRMS (ESI+): Calculated for $C_{27}H_{22}N_2O_3Na$ $[M + Na]^+$: 445.1522, found: 445.1523

***4-oxo-1,1-diphenyl-4-(thiophen-2-yl)butyl thiophene-2-carboxylate** (3z)*

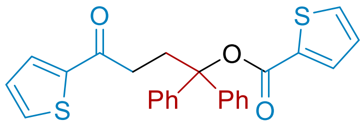

3z

Following the **general procedure C**, 1,3-di(thiophen-2-yl)propane-1,3-dione **2j** (118.1 mg, 0.5 mmol) and diphenylethylene **1a** (177.0 µL, 1.0 mmol) gave product **3z** (101.0 mg, 47%) as a yellow solid after purification (90:10 Cyclohexane/EtOAc).

^{1}H NMR (500 MHz, $CDCl_3$) δ 7.90 (dd, *J* = 3.8, 1.3 Hz, 1H), 7.61 (dd, *J* = 5.0, 1.2 Hz, 1H), 7.54 (m, 5H), 7.47 (dd, *J* = 4.0, 1.2 Hz, 1H), 7.37 (t, *J* = 7.8 Hz, 4H), 7.28 (t, *J* = 6.7 Hz, 2H), 7.16 (dd, *J* = 5.0, 3.7 Hz, 1H), 7.02 (dd, *J* = 5.0, 3.8 Hz, 1H), 3.44 – 3.38 (m, 2H), 2.90 – 2.84 (m, 2H).

^{13}C{^{1}H} NMR (125 MHz, $CDCl_3$) δ 192.1, 160.0, 144.0, 134.5, 133.8, 133.6, 132.5, 131.9, 128.5, 128.1, 128.0, 127.5, 126.0, 87.1, 33.7, 32.4.

HRMS (ESI+): Calculated for $C_{20}H_{17}OS$ $[M - C_5H_3O_2S]^+$: 305.1000, found: 305.0990

***2-(1-oxo-2,3-dihydro-1H-inden-2-yl)-1,1-diphenylethyl benzoate** (3aa)*

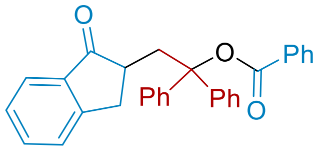

3aa

Following the **general procedure C**, 2-benzoyl-2,3-dihydro-1H-inden-1-one **2k** (95.1 mg, 0.5 mmol) and diphenylethylene **1a** (177.0 µL, 1.0 mmol) gave product **3aa** (101.6 mg, 47%) as a yellow solid after purification (95:5 Cyclohexane/EtOAc).

^{1}H NMR (500 MHz, $CDCl_3$) δ 8.21 (dd, *J* = 8.3, 1.4 Hz, 2H), 7.70 (d, *J* = 7.5 Hz, 1H), 7.65 (t, *J* = 7.5 Hz, 1H), 7.62 – 7.59 (m, 2H), 7.57 – 7.52 (m, 4H), 7.48 (td, *J* = 7.5, 1.4 Hz, 1H), 7.41 – 7.37 (m, 2H), 7.36 – 7.32 (m, 2H), 7.32 – 7.22 (m, 4H), 3.68 (dd, *J* = 14.4, 2.0 Hz, 1H), 3.27 (dd, *J* = 14.4, 10.6 Hz, 1H), 3.10 (dd, *J* = 17.0, 8.0 Hz, 1H), 2.67 (dd, *J* = 17.0, 5.2 Hz, 1H), 2.55 (m, 1H).

^{13}C{^{1}H} NMR (125 MHz, $CDCl_3$) δ 207.6, 164.9, 153.6, 144.7, 144.4, 136.1, 134.7, 133.3, 131.6, 129.8, 128.8, 128.6, 128.5, 127.5, 127.4, 127.4, 126.4, 126.3, 126.0, 123.9, 87.4, 44.5, 37.7, 34.0.

HRMS (ESI+): Calculated for $C_{23}H_{19}O$ $[M -C_7H_5O_2]^+$: 311.1436, found: 311.1432

***4-oxo-1,1,4-triphenylbutyl acetate** (3ab)*

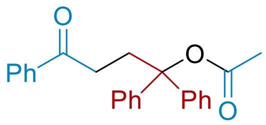

3ab

Following the **general procedure C**, 1-phenylbutane-1,3-dione **2l** (162.2 mg, 0.5 mmol) and diphenylethylene **1a** (177.0 µL, 1.0 mmol) gave product **3ab** (78.9 mg, 44%) as a white solid after purification (90:10 Cyclohexane/EtOAc).

^{1}H NMR (500 MHz, $CDCl_3$) δ 7.72 (d, *J* = 7.0 Hz, 2H), 7.43 (t, *J* = 7.4 Hz, 1H), 7.36 – 7.29 (m, 6H), 7.26 – 7.20 (m, 4H), 7.17 – 7.11 (m, 2H), 3.21 – 3.14 (m, 2H), 2.78 – 2.71 (m, 2H), 2.08 (s, 3H).

^{13}C{^{1}H} NMR (125 MHz, $CDCl_3$) δ 199.2, 168.9, 144.3, 136.9, 133.1, 128.6, 128.4, 128.0, 127.3, 126.0, 86.1, 33.0, 31.7, 22.3.

HRMS (ESI+): Calculated for $C_{24}H_{26}NO_3$ $[M + NH_4]^+$: 376.1907, found: 376.2309

***5,5-dimethyl-4-oxo-1,1-diphenylhexyl pivalate* (3ac)**

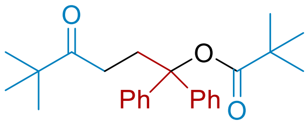

3ac

Following a modified version of **general procedure C**, 2,2,6,6-tetramethyl-3,5-heptanedione **2m** (92.1 mg, 0.5 mmol), diphenylethylene **1a** (177.0 µL, 1.0 mmol) and DBU (instead of 2,6-lutidine) (30.0 µL, 0.2 mmol) gave product **3ac** (159.3 mg, 84%) as a white solid after purification (98:2 Cyclohexane/EtOAc).

^{1}H NMR (300 MHz, $CDCl_3$) δ 7.39 – 7.33 (m, 4H), 7.33 – 7.25 (m, 4H), 7.24 – 7.17 (m, 2H), 3.10 – 3.02 (m, 2H), 2.35 – 2.27 (m, 2H), 1.31 (s, 9H), 1.02 (s, 9H).

^{13}C{^{1}H} NMR (75 MHz, $CDCl_3$) δ 214.9, 175.8, 144.7, 128.3, 127.2, 125.9, 85.6, 44.3, 39.8, 31.2, 30.6, 27.5, 26.5.

HRMS (ESI+): Calculated for $C_{25}H_{32}O_3$ [M + Na]$^+$: 403.2243, found: 403.2244

***1-(2-(tert-butyl)-2-hydroxy-5,5-diphenyltetrahydrofuran-3-yl)-2,2-dimethylpropan-1-one* (3ac')**

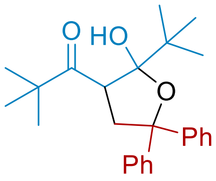

3ac'

Following the **general procedure C**, 2,2,6,6-tetramethyl-3,5-heptanedione **2m** (92.1 mg, 0.5 mmol) and diphenylethylene **1a** (177.0 µL, 1.0 mmol) gave product **3ac'** (171.2 mg, 90%) as a white solid after purification (98:2 Cyclohexane/EtOAc).

^{1}H NMR (500 MHz, $CDCl_3$) δ 7.51 – 7.45 (m, 4H), 7.37 – 7.33 (m, 2H), 7.26 (dd, *J* = 11.4, 4.0 Hz, 3H), 7.18 – 7.13 (m, 1H), 6.11 (s, 1H), 3.61 (dd, *J* = 12.5, 6.6 Hz, 1H), 3.25 (dd, *J* = 12.5, 6.6 Hz, 1H), 2.73 (t, *J* = 12.5 Hz, 1H), 1.23 (s, 9H), 0.98 (s, 9H).

^{13}C{^{1}H} NMR (75 MHz, $CDCl_3$) δ 221.1, 148.4, 145.8, 128.4, 128.1, 127.1, 126.7, 126.0, 125.8, 111.3, 86.6, 46.0, 45.9, 45.8, 39.0, 27.1, 25.8.

HRMS (ESI+): Calculated for $C_{25}H_{31}O_2$ [M - OH]$^+$: 363.2424, found: 363.243.

***1-(4-((S)-2-((tert-butoxycarbonyl)amino)-3-methoxy-3-oxopropyl)phenyl)-4-oxo-4-phenylbutyl benzoate* (4)**

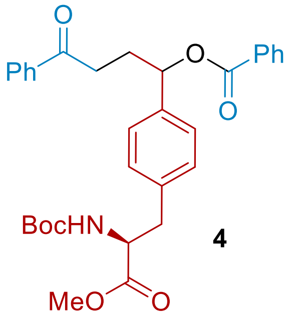

4

Following the **general procedure C**, 1,3-diphenylpropane-1,3-dione **2a** (112.1 mg, 0.5 mmol) and methyl (*S*)-2-((*tert*-butoxycarbonyl)amino)-3-(4-vinylphenyl)propanoate **1r** (305.4 mg, 1.0 mmol) gave product **4** (233.5 mg, 86%) as a yellow solid after purification (80:20 Cyclohexane/EtOAc).

^{1}H NMR (300 MHz, $CDCl_3$) δ 8.08 – 8.03 (m, 2H), 7.92 – 7.86 (m, 2H), 7.57 – 7.47 (m, 2H), 7.45 – 7.35 (m, 6H), 7.14 (d, *J* = 8.2 Hz, 2H), 6.10 (dd, *J* = 8.1, 5.4 Hz, 1H), 5.05 (d, *J* = 8.3 Hz, 1H), 4.58 (q, *J* = 7.4 Hz, 1H), 3.67 (s, 3H), 3.18 – 2.95 (m, 4H), 2.57 – 2.31 (m, 2H), 1.37 (s, 9H).

^{13}C{^{1}H} NMR (75 MHz, $CDCl_3$) δ 198.9, 172.3, 165.7, 155.1, 139.0, 136.8, 136.1, 133.1, 133.1, 130.2, 129.7, 129.6, 128.6, 128.4, 128.0, 126.6, 79.9, 75.6, 54.4, 52.2, 38.1, 34.5, 30.8, 28.3.

HRMS (ESI+): Calculated for $C_{25}H_{30}NO_5$ $[M - C_7H_5O_2]^+$: 424.2124, found 424.2118

Methyl 4-(1-(benzoyloxy)-4-oxo-1-(3,5,5,8,8-pentamethyl-5,6,7,8-tetrahydronaphthalen-2-yl)-4-phenylbutyl)benzoate (5)

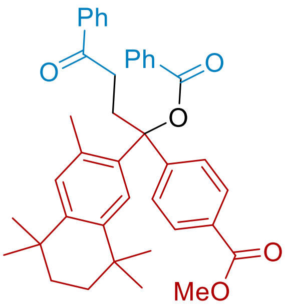

5

Following the **general procedure C**, 1,3-diphenylpropane-1,3-dione **2a** (112.1 mg, 0.5 mmol) and methyl 4-(1-(3,5,5,8,8-pentamethyl-5,6,7,8-tetrahydronaphthalen-2-yl)vinyl)benzoate **1s** (362.5 mg, 1.0 mmol) gave product **5** (225.3 mg, 80%) as a yellow solid after purification (95:5 Cyclohexane/EtOAc).

^{1}H NMR (500 MHz, $CDCl_3$) δ 8.21 – 8.15 (m, 2H), 8.08 – 8.02 (m, 2H), 7.90 (s, 1H), 7.74 (dd, *J* = 8.4, 1.4 Hz, 2H), 7.62 – 7.57 (m, 3H), 7.53 – 7.45 (m, 3H), 7.36 – 7.32 (m, 2H), 6.91 (s, 1H), 3.90 (s, 3H), 3.72 (ddd, *J* = 14.7, 10.8, 4.9 Hz, 1H), 3.13 (ddd, *J* = 14.3, 10.8, 4.3 Hz, 1H), 2.98 (ddd, *J* = 16.0, 10.8, 4.9 Hz, 1H), 2.80 (ddd, *J* = 16.8, 10.8, 4.3 Hz, 1H), 1.86 (s, 3H), 1.77 – 1.67 (m, 4H), 1.46 (s, 3H), 1.40 (s, 3H), 1.25 (s, 3H), 1.24 (s, 3H).

^{13}C{^{1}H} NMR (125 MHz, $CDCl_3$) δ 198.9, 166.7, 163.7, 149.0, 144.4, 141.8, 137.5, 136.7, 133.1, 133.0, 132.5, 130.8, 130.6, 129.7, 129.4, 129.0, 128.7, 128.5, 127.9, 126.6, 125.0, 87.0, 52.1, 35.3, 35.2, 34.2, 33.8, 32.8, 32.3, 32.0, 31.8, 31.7, 21.1.

HRMS (ESI+): Calculated for $C_{33}H_{37}O_3$ $[M - C_7H_5O_2]^+$: 481.2743, found 481.2609

4.6. Scale up of the reaction

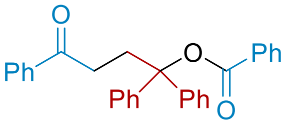

3a

The reaction was scaled up to 1.0 mmol following the **general procedure C** but keeping the charge constant to 2.9 F/mol instead of 8 hours of duration.

Following a slightly modified **general procedure C**, 1,3-diphenylpropane-1,3-dione **2a** (224.3 mg, 1 mmol) and diphenylethylene **1a** (352 µL, 2.0 mmol) gave product **3a** (328 mg, 78 %) as a white solid after purification (95:5 Cyclohexane/EtOAc).

Spectroscopic data are consistent with the previous NMR results obtained using the optimized reaction conditions.

4.7. Unsuccessful examples

Substrate structure	Conditions used	Observations
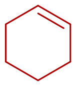	Standard conditions	No reaction
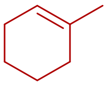	Standard conditions	No reaction
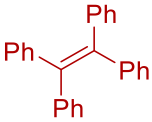	i) Standard conditions<br>ii) 10 mA, 8 h<br>iii) 15 mA, 8h	No reaction in all cases
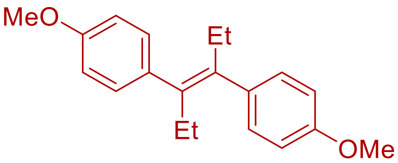	Standard conditions	No reaction
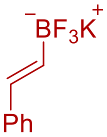	Standard conditions	No reaction
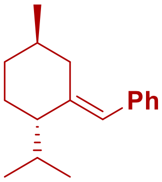	Standard conditions	Complex mixture
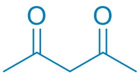	Standard conditions	Complex mixture
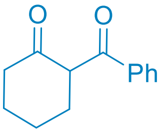	Standard conditions	Complex mixture
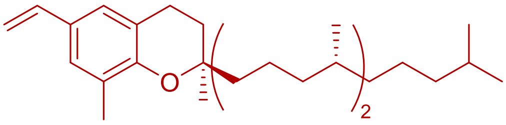	Standard conditions	No reaction

5. Derivatizations

5.1. Synthesis of 4-hydroxy-1,1,4-triphenylbutyl benzoate 6.

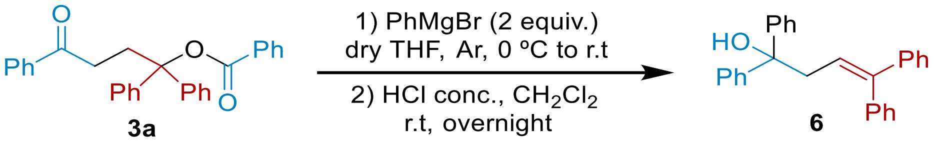

Under argon atmosphere, 4-oxo-1,1,4-triphenylbutyl benzoate **3a** (42.0 mg, 0.1 mmol) was dissolved in dry THF (2 mL). Then, 2 equiv. of PhMgBr were added to the reaction mixture at 0 ºC and it was stirred until complete consumption of the starting material was observed by TLC. Then, the reaction was diluted with ethyl acetate and quenched with cold water. The solution was extracted twice with ethyl acetate. The organic layers were combined, washed with brine, dried over Na_2SO_4, filtered and the solvent was removed under vacuum. Then, the residue was dissolved in CH_2Cl_2 and one drop of HCl 37% was added. The reaction mixture was stirred and monitored by TLC until complete consumption of the starting material. After completion, the reaction was quenched with $NaHCO_3$. The solution was extracted twice with CH_2Cl_2. The organic layers were combined, washed with brine, dried over Na_2SO_4, filtered and the solvent was removed under vacuum. The final product **6** was characterized without further purification (29 mg, 73%)

^{1}H NMR (300 MHz, $CDCl_3$) δ 7.40 – 7.26 (m, 11H), 7.25 – 7.17 (m, 5H), 7.14 – 7.05 (m, 4H), 6.04 (t, *J* = 7.2 Hz, 1H), 3.15 (d, *J* = 7.2 Hz, 2H).

^{13}C{^{1}H} NMR (75 MHz, $CDCl_3$) δ 146.8, 145.4, 142.6, 134.0, 130.0, 128.4, 128.3, 128.2, 127.4, 127.3, 127.3, 127.1, 126.3, 124.2, 78.4, 42.3.

HRMS (ESI+): Calculated for $C_{28}H_{25}O$ $[M + H]^+$: 376.1827, found 376.1830

5.2. General procedure E: Elimination reactions

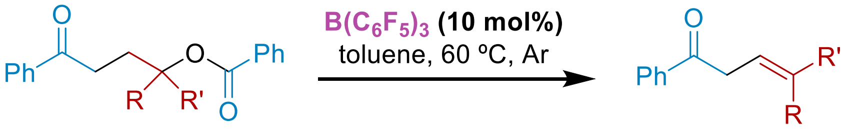

Under argon atmosphere, the corresponding 4-benzoate derivative (0.1 mmol) and tris(pentafluorophenyl)borane $[B(C_6F_5)_3]$ (10 mol%) were dissolved in degassed toluene (0.5 mL). Then, the reaction mixture was stirred at 60 ºC overnight and the resulting solution was concentrated under reduced pressure. The product was purified by flash column chromatography to give the corresponding but-3-en-1-one.

***<u>1,4,4-triphenylbut-3-en-1-one</u>* (7)**

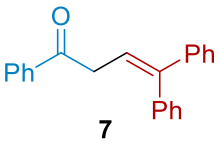

7

Following the **general procedure E**, 4-oxo-1,1,4-triphenylbutyl benzoate **3a** (42.1 mg, 0.1 mmol) and $[B(C_6F_5)_3]$ (5 mg, 10 mol%) gave product **7** (27.8 mg, 93 %) as a yellow solid after purification (94:6 Cyclohexane/EtOAc).

^{1}H NMR (500 MHz, $CDCl_3$) δ 7.88 – 7.82 (m, 2H), 7.59 – 7.49 (m, 1H), 7.45 – 7.30 (m, 6H), 7.30 – 7.18 (m, 6H), 6.42 (t, *J* = 7.1 Hz, 1H), 3.81 (d, *J* = 7.1 Hz, 2H).

^{13}C{^{1}H} NMR (125 MHz, $CDCl_3$) δ 198.3, 144.9, 142.2, 139.7, 136.8, 133.3, 129.9, 128.7, 128.6, 128.4, 128.3, 127.6, 127.6, 127.5, 121.3, 40.0.

HRMS (ESI+): Calculated for $C_{22}H_{19}O$ $[M + H]^+$: 299.1358, found 299.1375

***<u>1-phenyl-2-(2,3,4,5-tetrahydro-[1,1'-biphenyl]-2-yl)ethan-1-one</u>* (10)**

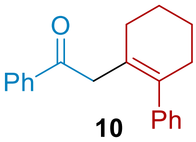

10

Following the **general procedure E**, 2-(2-oxo-2-phenylethyl)-1-phenylcyclohexyl benzoate **3h** (39.8 mg, 0.1 mmol) and $[B(C_6F_5)_3]$ (5 mg, 10 mol%) gave product **10** (26.3 mg, 95%) as a yellow solid after purification (98:2 Cyclohexane/EtOAc).

^{1}H NMR (500 MHz, $CDCl_3$) δ 7.88 – 7.82 (m, 2H), 7.54 – 7.49 (m, 1H), 7.43 – 7.38 (m, 2H), 7.37 – 7.33 (m, 2H), 7.32 – 7.28 (m, 2H), 7.24 – 7.19 (m, 1H), 6.03 (td, *J* = 4.0, 1.3 Hz, 1H), 3.56 – 3.49 (m, 1H), 2.99 (dd, *J* = 17.0, 10.7 Hz, 1H), 2.84 (dd, *J* = 17.0, 2.0 Hz, 1H), 2.23 – 2.17 (m, 2H), 1.93 – 1.84 (m, 1H), 1.71 – 1.62 (m, 3H).

^{13}C{^{1}H} NMR (125 MHz, $CDCl_3$) δ 200.0, 142.0, 140.9, 137.4, 133.1, 128.6,128.6, 128.2, 127.4, 127.0, 126.5, 42.4, 31.7, 27.8, 26.2, 18.5.

HRMS (ESI+): Calculated for $C_{20}H_{21}O$ $[M + H]^+$: 277.1514, found 277.1510

5.3. Synthesis of 4-hydroxy-1,1,4-triphenylbutyl benzoate 8.

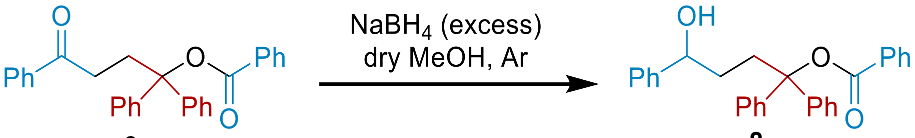

3a **8**

Under argon atmosphere, 4-oxo-1,1,4-triphenylbutyl benzoate **3a** (42.1 mg, 0.1 mmol) was dissolved in degassed MeOH (2 mL, 0.05M). Then, an excess of $NaBH_4$ was added to the reaction mixture and it was stirred until complete consumption of the starting material was observed by TLC. Then, the reaction was diluted with ethyl acetate and quenched with NH_4Cl. The solution was extracted twice with ethyl acetate. The combined organic layers extracts were dried over anhydrous $MgSO_4$, filtered and the solvent was removed under vacuum. The final product 4-hydroxy-1,1,4-triphenylbutyl benzoate **8** was characterized without further purification (41 mg, 97%).

^{1}H NMR (300 MHz, $CDCl_3$) δ 8.17 – 8.11 (m, 2H), 7.64 – 7.56 (m, 1H), 7.54 – 7.38 (m, 7H), 7.36 – 7.27 (m, 6H), 7.24 – 7.15 (m, 4H), 4.62 (m, 1H), 3.14 (m, 1H), 2.89 (m, 1H), 1.84 – 1.59 (m, 2H).

^{13}C{^{1}H} NMR (75 MHz, $CDCl_3$) δ 164.6, 144.9, 144.3, 133.1, 131.4, 129.8, 128.6, 128.4, 127.8, 127.8, 127.2, 126.1, 126.0, 87.1, 74.5, 33.5, 32.6.

HRMS (ESI+): Calculated for $C_{22}H_{20}$ $[M - C_7H_5O_2 - OH]^+$: 284,1565, found 284,1500

5.4. Synthesis of 1,1,4-triphenylbutane-1,4-diol 9.

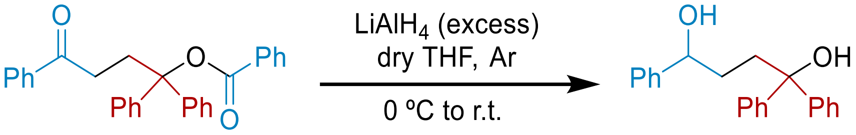

3a **9**

Under argon atmosphere, excess of $LiAlH_4$ was suspended in dry THF (1 mL). Then, a THF (2 mL) solution of the corresponding 4-oxobenzoate derivative (42.1 mg, 0.1 mmol) was added dropwise to the reaction mixture at 0 ºC. The reaction mixture was stirred at room temperature until the complete consumption

of the starting material was observed by TLC. Then, the reaction was diluted with diethyl ether and quenched with NaCl. The solution was extracted twice with diethyl ether. The combined organic layers extracts were dried over anhydrous $MgSO_4$, filtered and the solvent was removed under vacuum. The final product was purified by flash column chromatography (60:40 Cyclohexane/EtOAc) to give 1,1,4-triphenylbutane-1,4-diol **9** (32 mg, 98%).

^{1}H NMR (300 MHz, $CDCl_3$) δ 7.41 – 7.36 (m, 4H), 7.32 – 7.27 (m, 9H), 7.23 – 7.18 (m, 2H), 4.72 (t, *J* = 6.0 Hz, 1H), 2.90 (br, 1H), 2.52 – 2.35 (m, 2H), 2.21 (br, 1H), 1.84 – 1. 77 (m, 2H).

Spectroscopic data are consistent with those reported in the literature.[10]

6. Mechanism experiments

2-(2-methoxy-2,2-diphenylethyl)-1,3-diphenylpropane-1,3-dione (11)

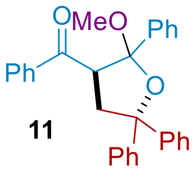

11

Following a slightly modified **general procedure C**, 1,3-diphenylpropane-1,3-dione **2a** (112.1 mg, 0.5 mmol), diphenylethylene **1a** (177.0 µL, 1.0 mmol) and methanol (instead of water) (61.0 µL, 3 equiv.) gave product **11** (134.7 mg, 62 %) as a white solid after purification (97:3 Cyclohexane/EtOAc).

^{1}H NMR (300 MHz, $CDCl_3$) δ 7.94 (d, *J* = 7.3 Hz, 2H), 7.68 – 7.58 (m, 4H), 7.56 – 7.48 (m, 1H), 7.42 – 7.33 (m, 6H), 7.31 – 7.23 (m, 2H), 7.08 (d, *J* = 3.4 Hz, 5H), 4.57 (dd, *J* = 12.1, 6.4 Hz, 1H), 3.28 (s, 3H), 3.25 – 3.20 (m, 1H), 3.06 (dd, *J* = 13.0, 6.5 Hz, 1H).

^{13}C{^{1}H} NMR (76 MHz, $CDCl_3$) δ 196.61, 146.72, 146.28, 138.10, 137.78, 133.15, 129.25, 128.48, 128.39, 128.35, 127.89, 127.75, 127.07, 127.00, 125.86, 125.58, 112.04, 88.71, 58.42, 51.75, 39.62.

HRMS (ESI+): Calculated for $C_{29}H_{23}O_2$ [M - CH_3O]$^+$: 403.1698, found 403.1696.

((3S,3aS,7aR)-2-methoxy-2,7a-diphenyloctahydrobenzofuran-3-yl)(phenyl)methanone (12)

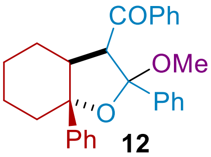

12

Following a slightly modified **general procedure C**, 1,3-diphenylpropane-1,3-dione **2a** (112.1 mg, 0.5 mmol), 1-phenyl-1-cyclohexene **1h** (158.2 mg, 1.0 mmol) and methanol (instead of water) (61.0 µL, 3 equiv.) gave product **12** (36.8 mg, 18 %) as a white solid after purification (95:5 Cyclohexane/EtOAc).

^{1}H NMR (300 MHz, $CDCl_3$) δ 7.71 – 7.66 (m, 2H), 7.40 – 7.32 (m, 5H), 7.29 – 7.21 (m, 3H), 7.21 – 7.07 (m, 5H), 4.26 (d, *J* = 12.3 Hz, 1H), 3.99 – 3.89 (m, 1H), 2.88 (s, 3H), 2.45 (d, *J* = 12.1 Hz, 1H), 2.18 (td, *J* = 13.1, 3.7 Hz, 1H), 1.90 – 1.60 (m, 4H), 1.39 – 1.26 (m, 2H).

^{13}C{^{1}H} NMR (75 MHz, $CDCl_3$) δ 195.58, 145.58, 139.82, 138.16, 132.68, 128.81, 128.16, 128.09, 128.07, 127.47, 127.12, 126.68, 107.02, 84.52, 61.13, 50.27, 43.46, 37.95, 24.79, 22.77, 21.15.

HRMS (ESI+): Calculated for $C_{27}H_{25}O_2$ [M - CH_3O]$^+$: 381.1855, found 381.1858.

6.1. Isotopic labelling experiments

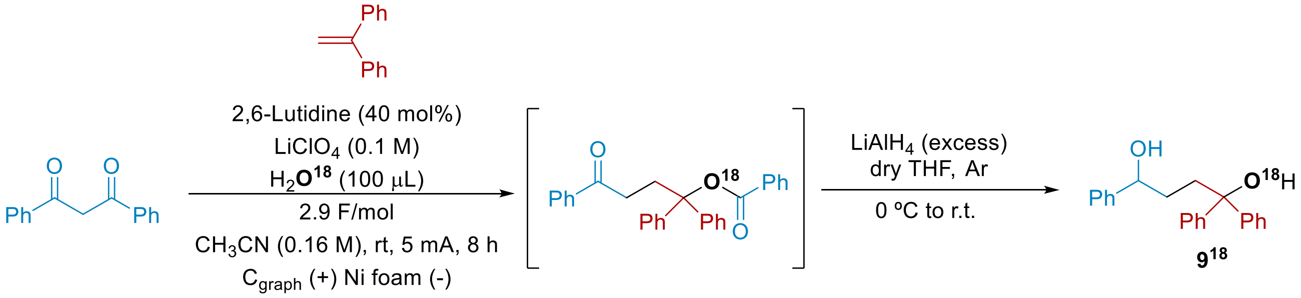

Following a modified general procedure C, 1,3-diphenylpropane-1,3-dione 1a (112.1 mg, 0.5 mmol), diphenylethylene **2a** (177.0 µL, 1.0 mmol) and H_2O^{18} (100 µL) gave product 3a (161 mg, 77%) as a white solid after purification (95:5 Cyclohexane/EtOAc). Then, the resulting labelled 3a was used as starting material (0.1 mmol) to follow the procedure described above for the synthesis of 4. Product **9**[18] was achieved (29 mg, 91%) as a white solid after purification (60:40 Cyclohexane/EtOAc).

^{1}H NMR (300 MHz, $CDCl_3$) δ 7.41 – 7.36 (m, 4H), 7.32 – 7.27 (m, 9H), 7.23 – 7.18 (m, 2H), 4.72 (t, *J* = 6.0 Hz, 1H), 2.90 (br, 1H), 2.52 – 2.35 (m, 2H), 2.21 (br, 1H), 1.84 – 1. 77 (m, 2H).

Spectroscopic data are consistent with the previous NMR results obtained using the **general procedure C**.

HRMS (ESI+): Calculated for $C_{22}H_{22}OO^{18}Na$ [M+Na]+: 343.1554, found 343.1550

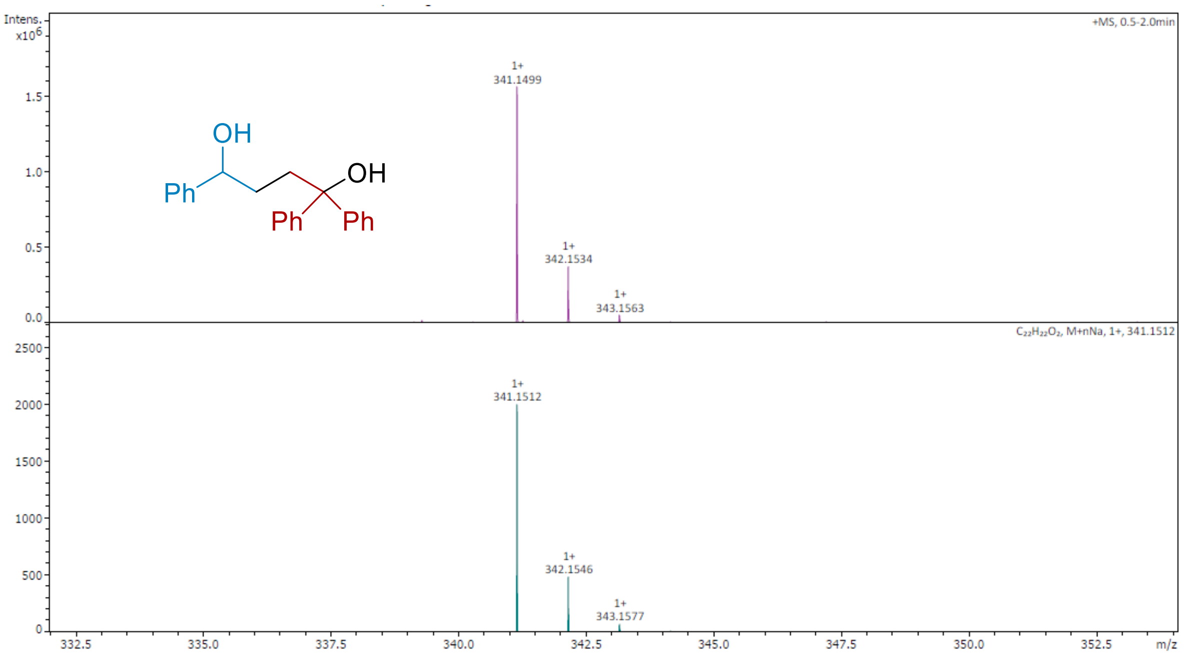

Figure S1: High resolution mass spectrum of **9** using electrospray ionization technique. The experimental spectrum for molecule **9** is shown above, with the base peak corresponding to the ion $[M+Na]^+$ observed at m/z 431.1499. Below, the calculated spectrum for the ion $[M+Na]^+$ is presented for comparison.

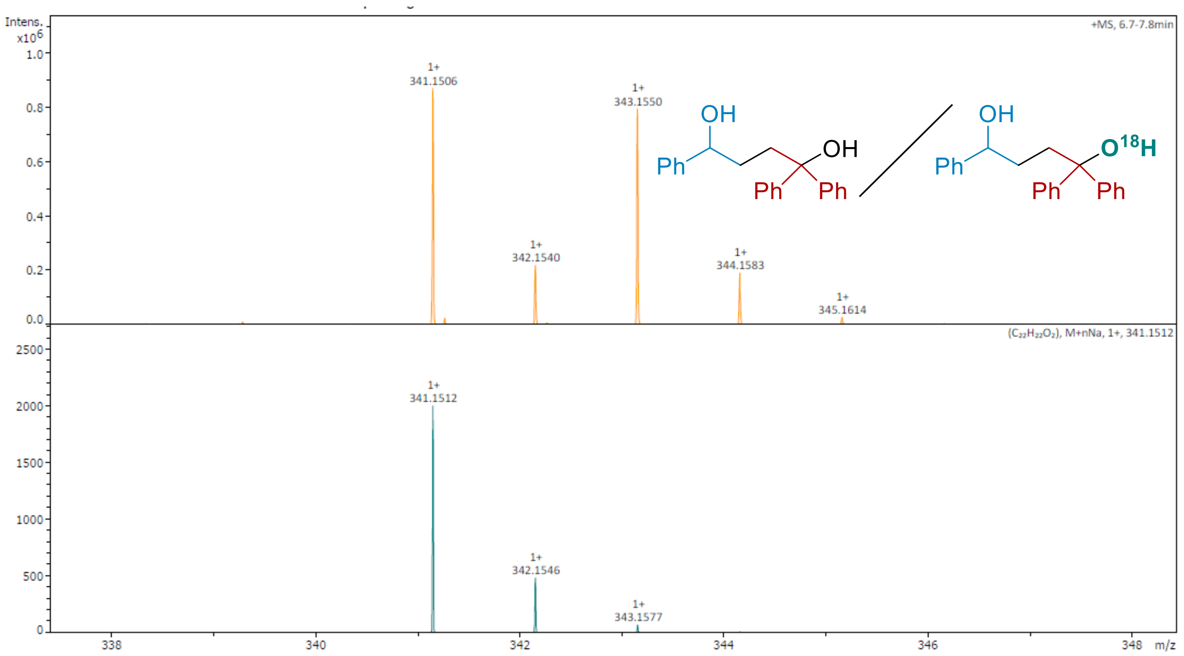

Figure S2: High resolution mass spectrum of **9**[18] using electrospray ionization technique. The experimental spectrum for molecule **9**[18] is shown above. Both the unlabeled and labeled molecules are detected, with the labeled molecule displaying a shift of two mass units to m/z 433.1550. The labeled proportion is approximately 50%. Below, the calculated spectrum for the ion $[M+Na]^+$ is presented for comparison.

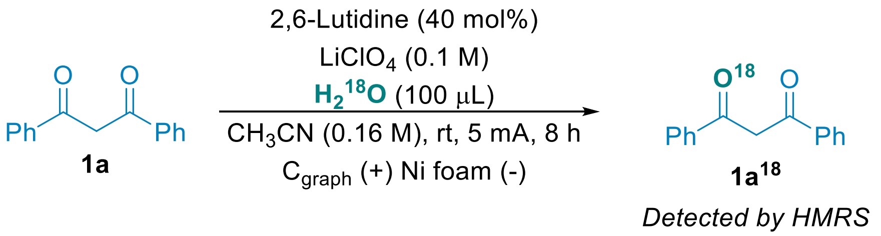

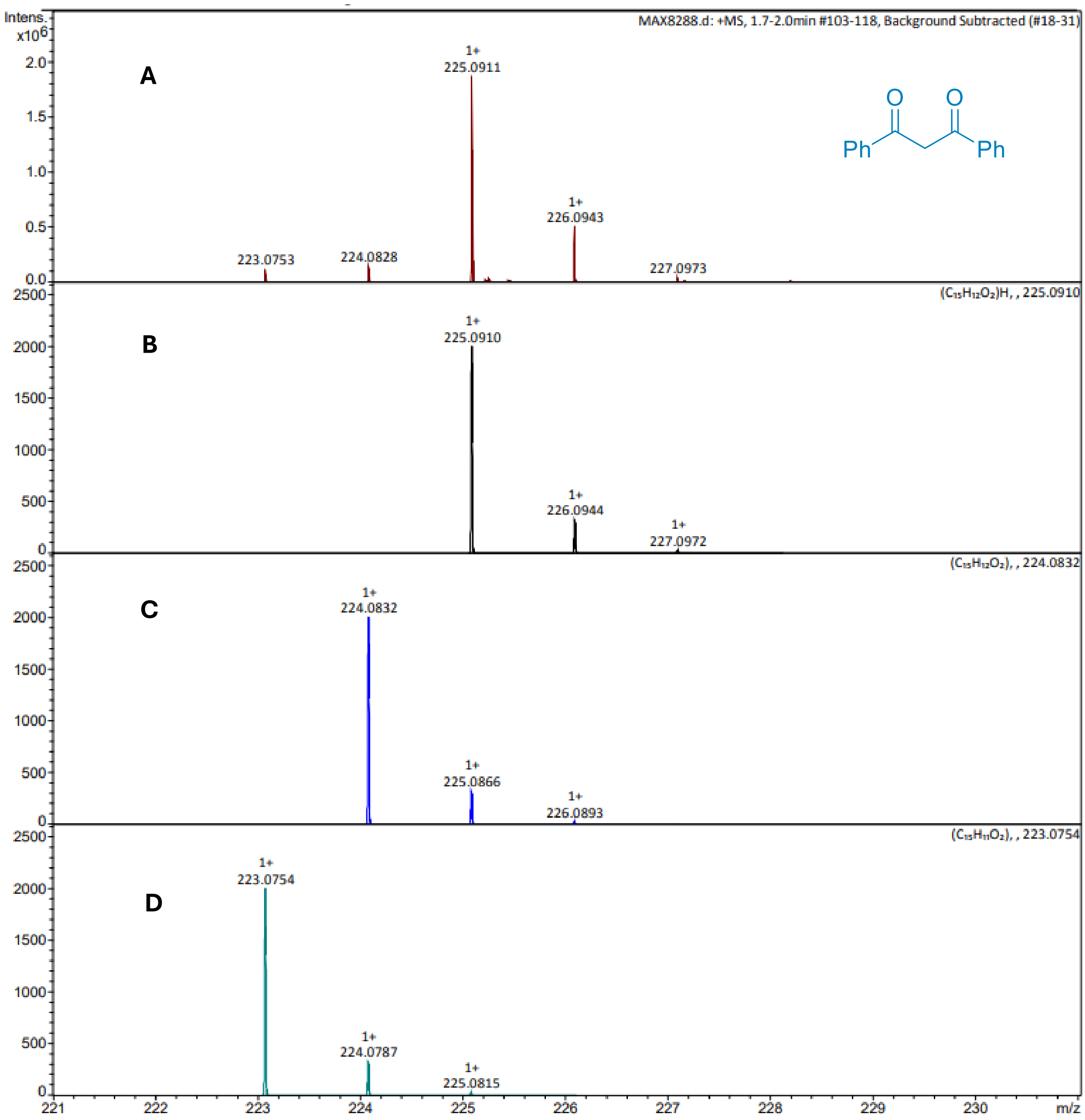

Figure S3: High resolution mass spectrum of **1a** using atmospheric-pressure chemical ionization technique. The experimental spectrum for molecule **1a** is shown above (**A**), with the base peak corresponding to the ion $[M+H]^+$ observed at m/z 225.0911, combined with M+ (m/z = 224.0828) and M-1 (m/z = 223.0753). Below, the calculated spectrum for the ion $[M+H]^+$ (**B**), M+ (**C**) and M-1 (**D**) are presented for comparison.

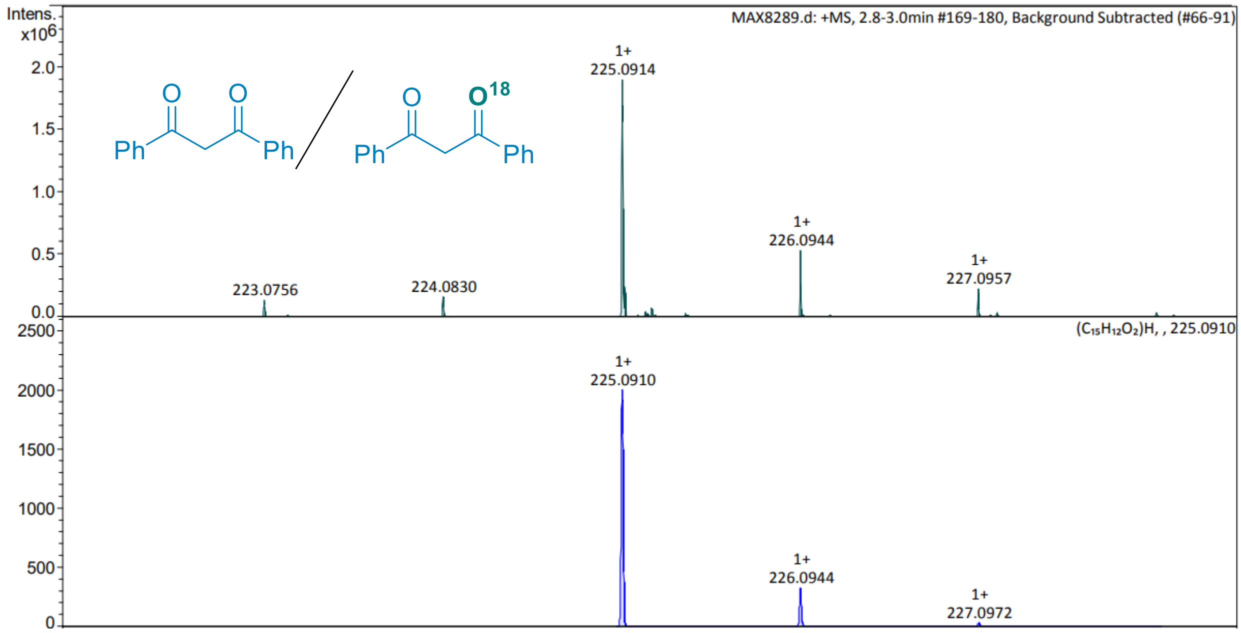

Figure S4: High resolution mass spectrum of **1a**[18] using atmospheric-pressure chemical ionization technique. The experimental spectrum for molecule **1a**[18] is shown above. Both the unlabeled and labeled molecules are detected, with the labeled molecule displaying a shift of two mass units to m/z 227.0957. The labeled proportion is approximately 10%. Below, the calculated spectrum for the ion $[M+H]^+$ is presented for comparison.

7. NMR spectroscopic data

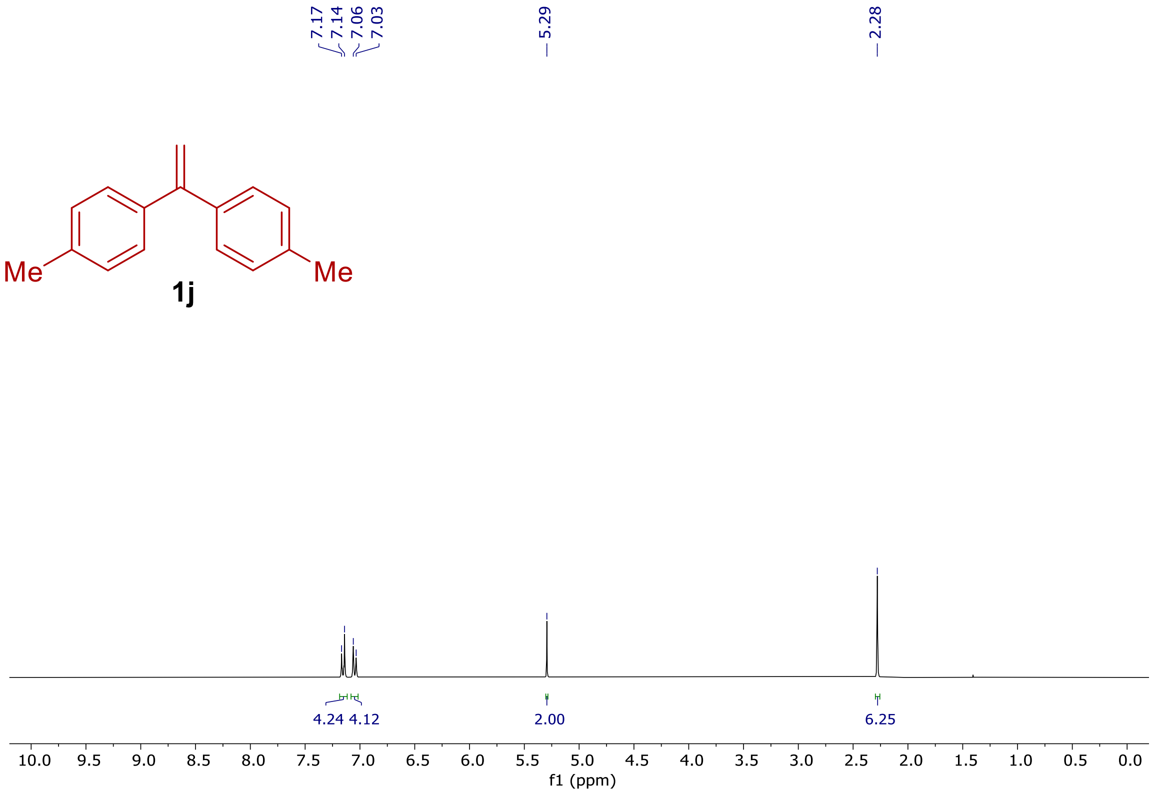

Figure S5. ^1H NMR spectrum of **1j** in $CDCl_3$ (300 MHz)

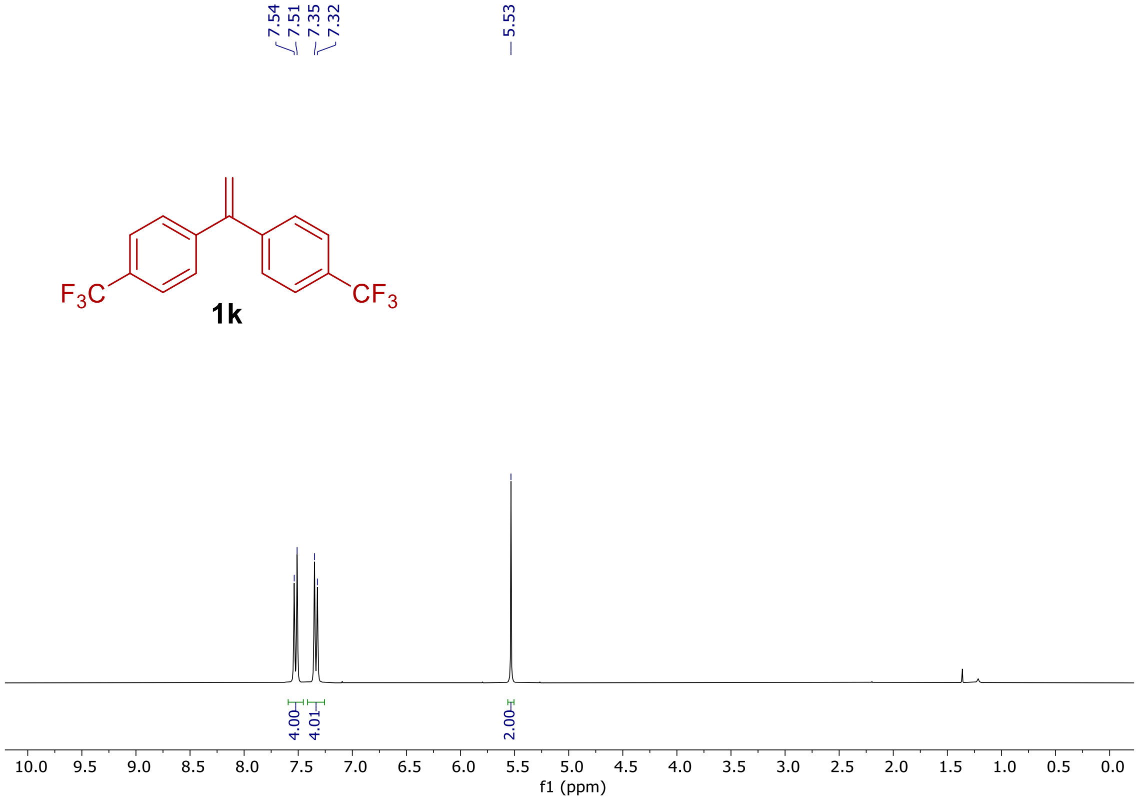

Figure S2. ^{1}H NMR spectrum of **1k** in $CDCl_3$ (300 MHz)

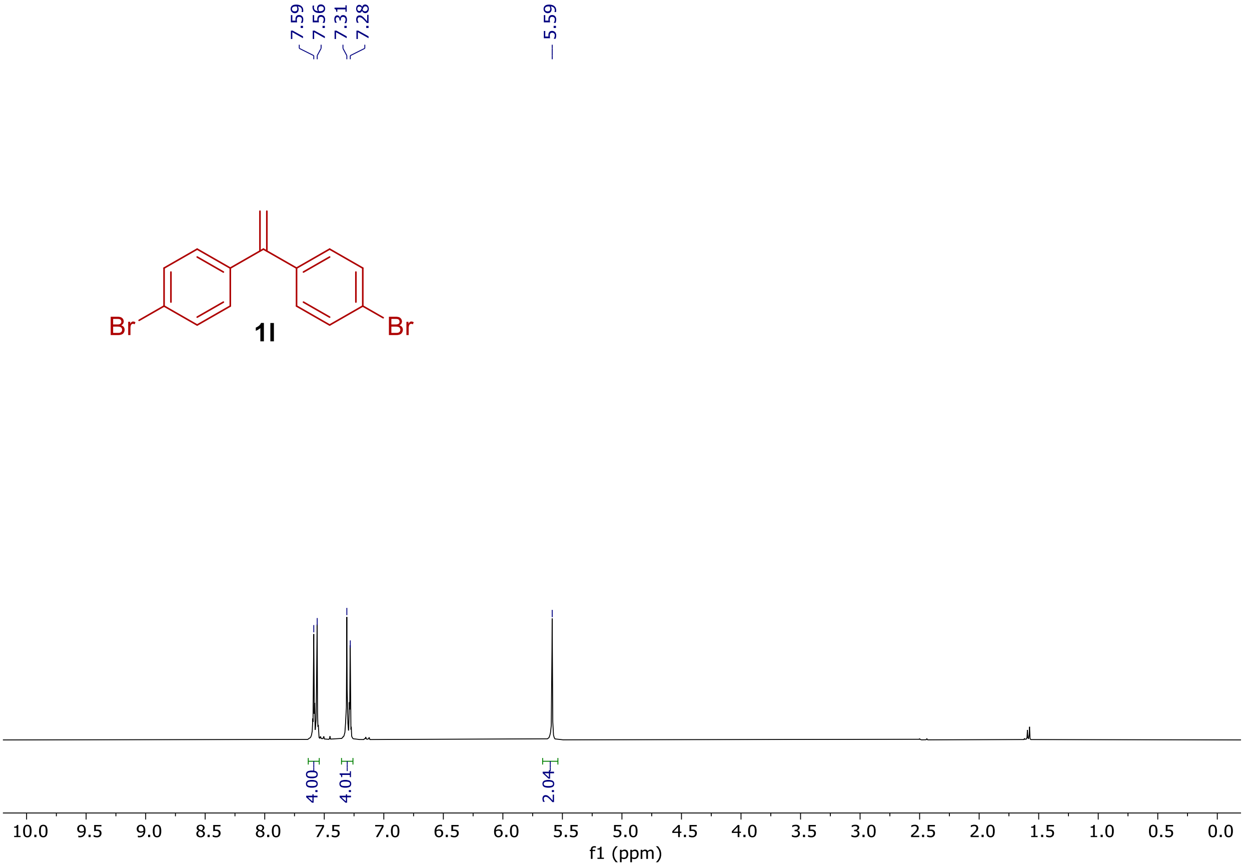

Figure S3. ^{1}H NMR spectrum of **1l** in $CDCl_3$ (300 MHz)

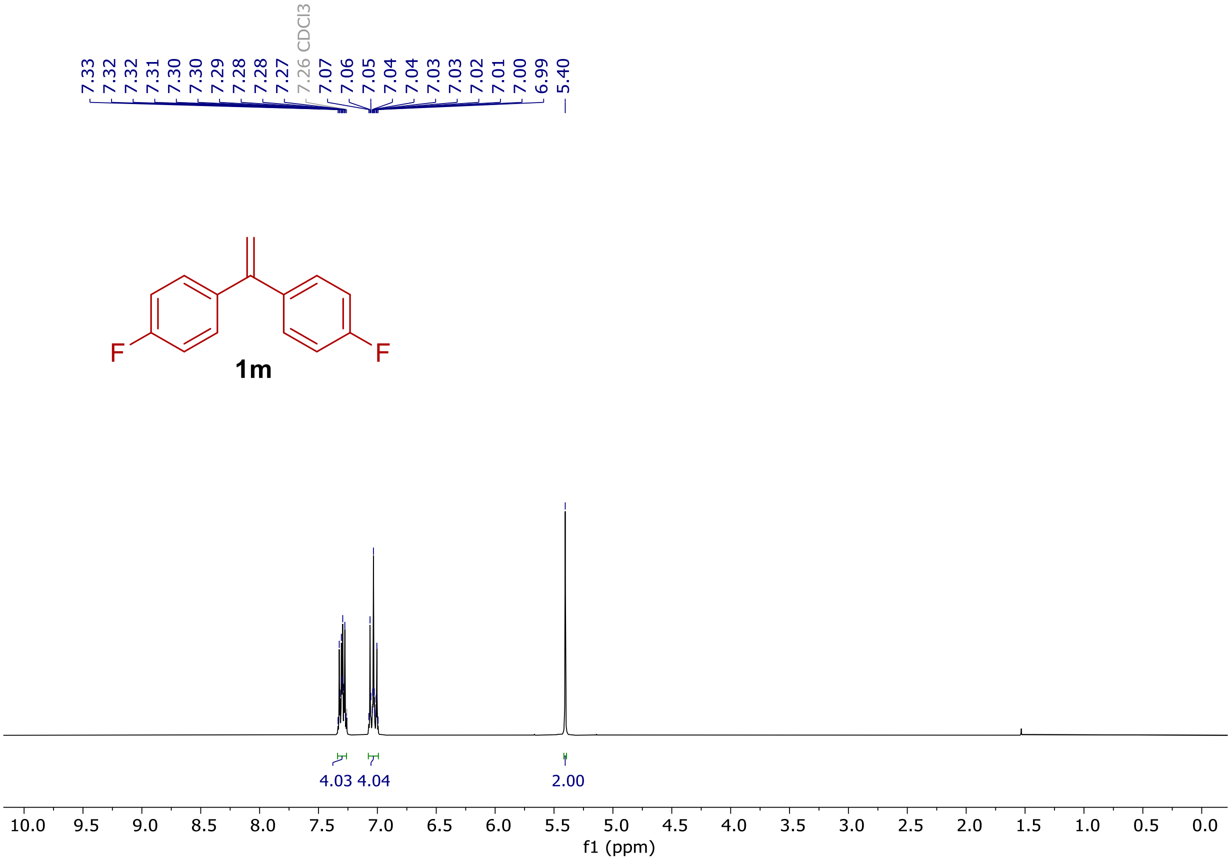

Figure S4. ^{1}H NMR spectrum of **1m** in $CDCl_3$ (300 MHz)

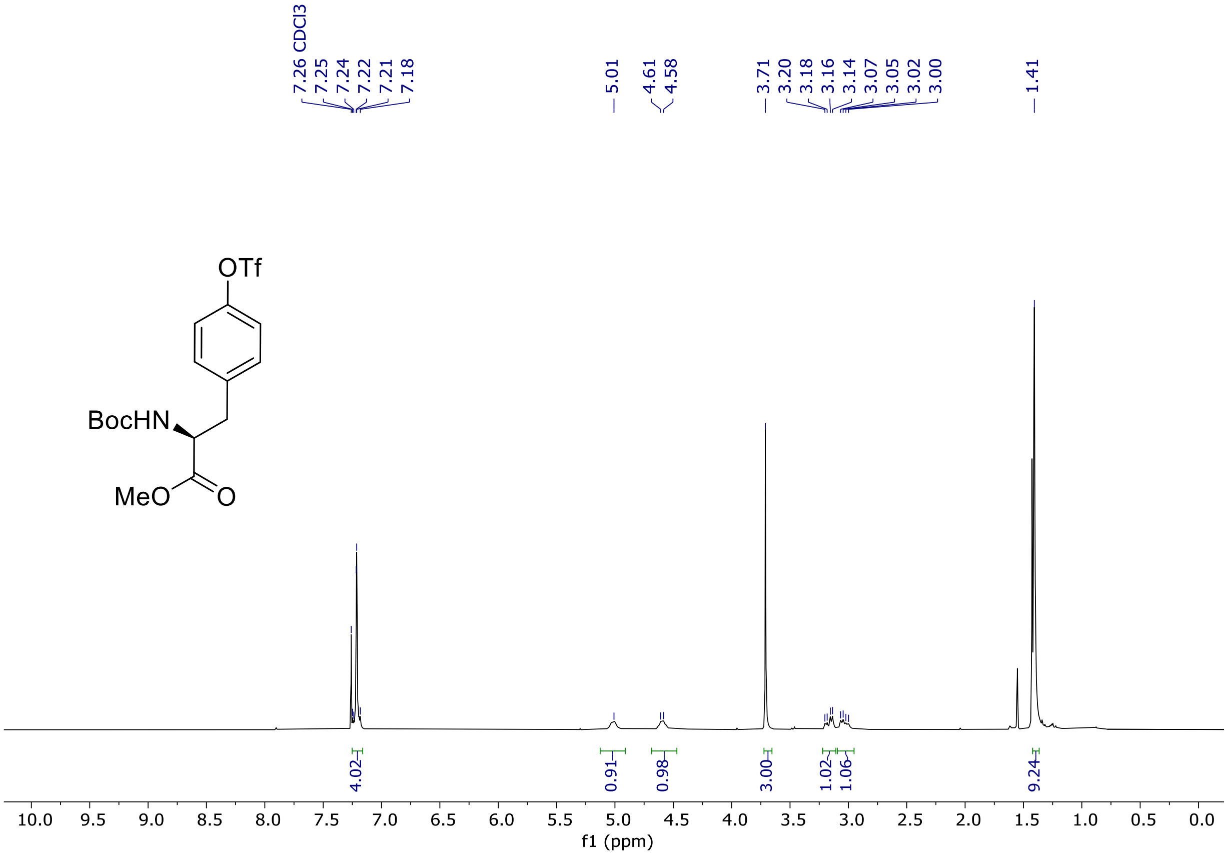

Figure S5. ^{1}H NMR spectrum of **1Sa** in $CDCl_3$ (300 MHz)

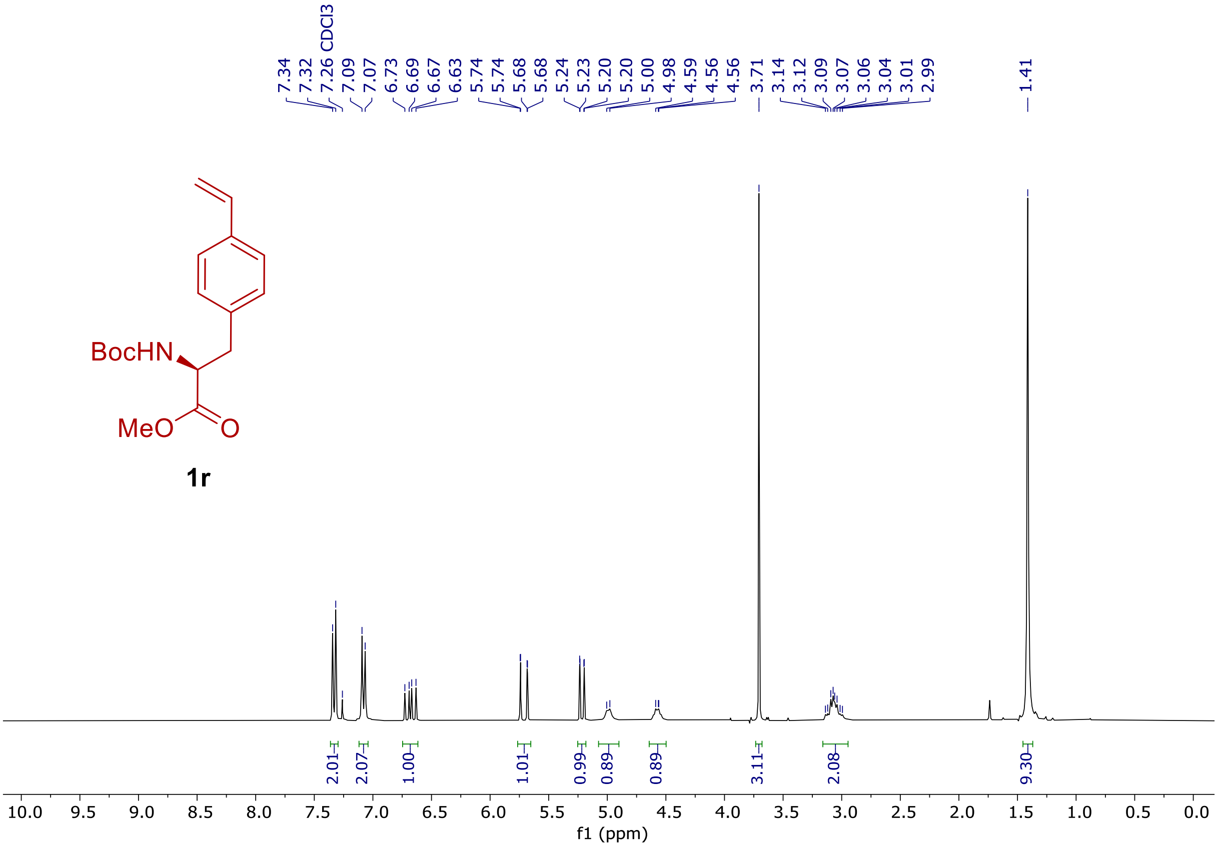

Figure S6. ^{1}H NMR spectrum of **1r** in $CDCl_3$ (300 MHz)

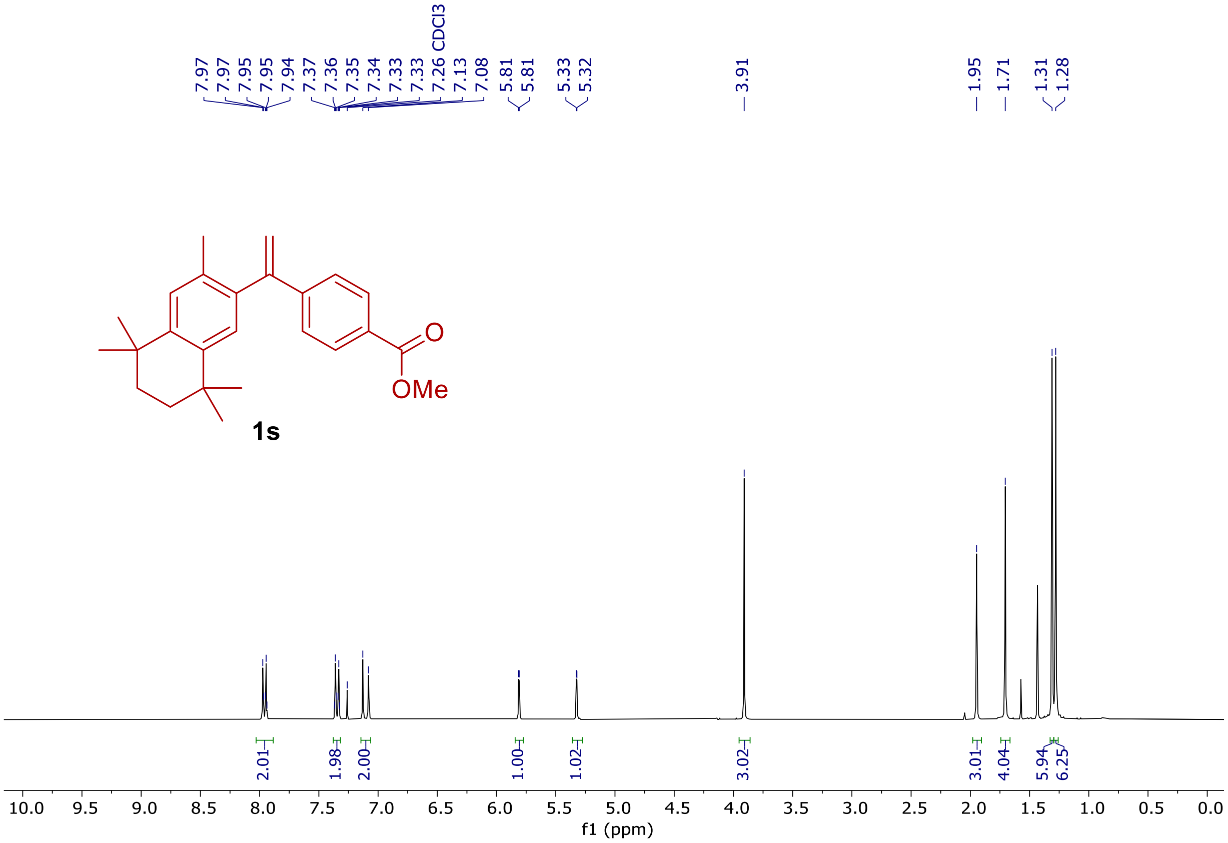

Figure S7. ^{1}H NMR spectrum of **1s** in $CDCl_3$ (300 MHz)

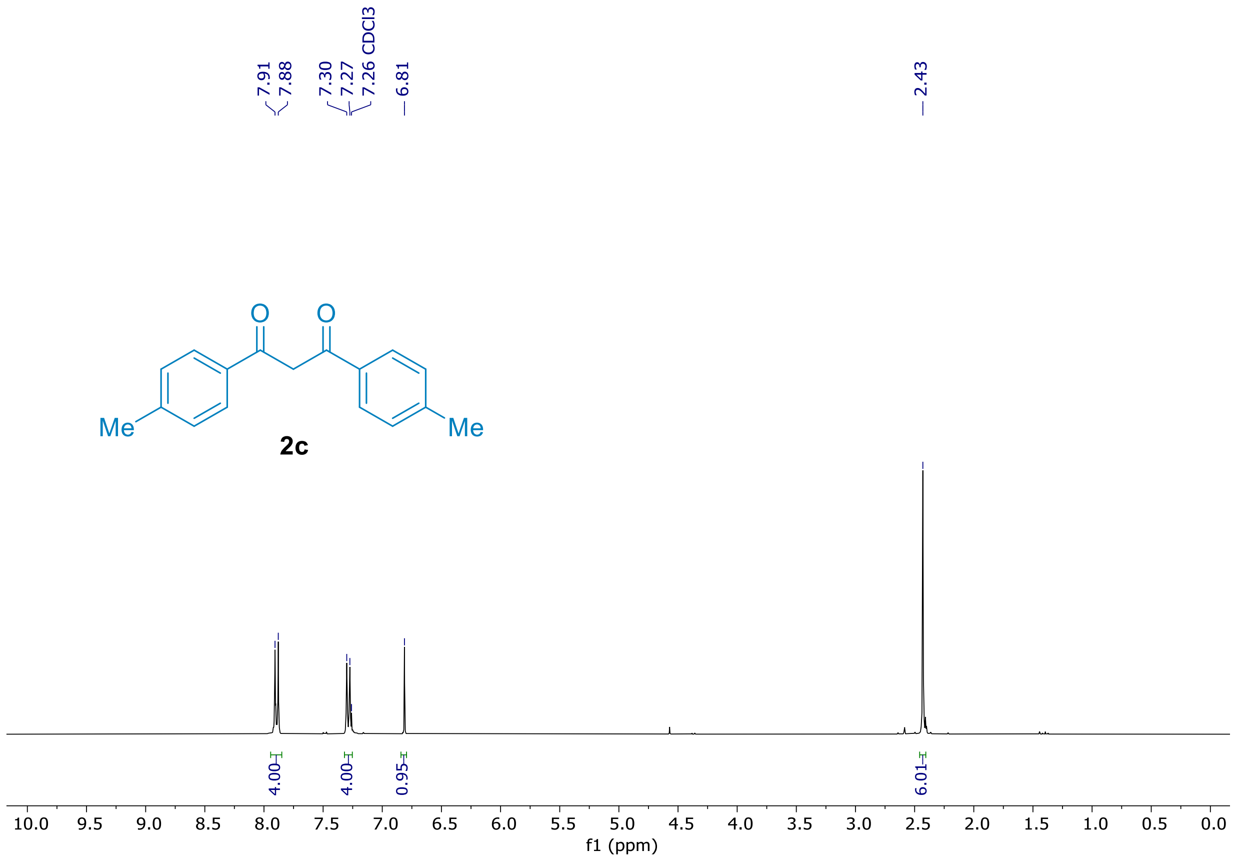

Figure S8. ^{1}H NMR spectrum of **2c** in $CDCl_3$ (300 MHz)

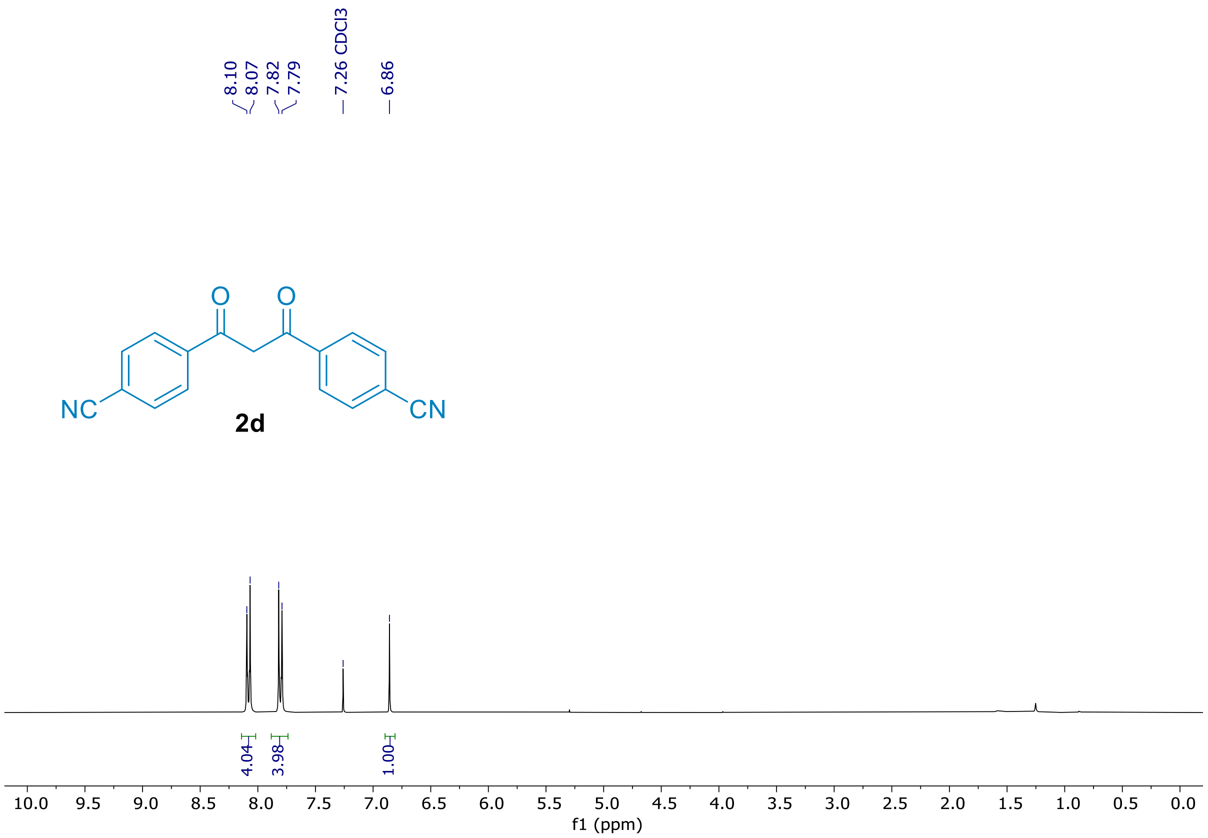

Figure S9. ^{1}H NMR spectrum of **2d** in $CDCl_3$ (300 MHz)

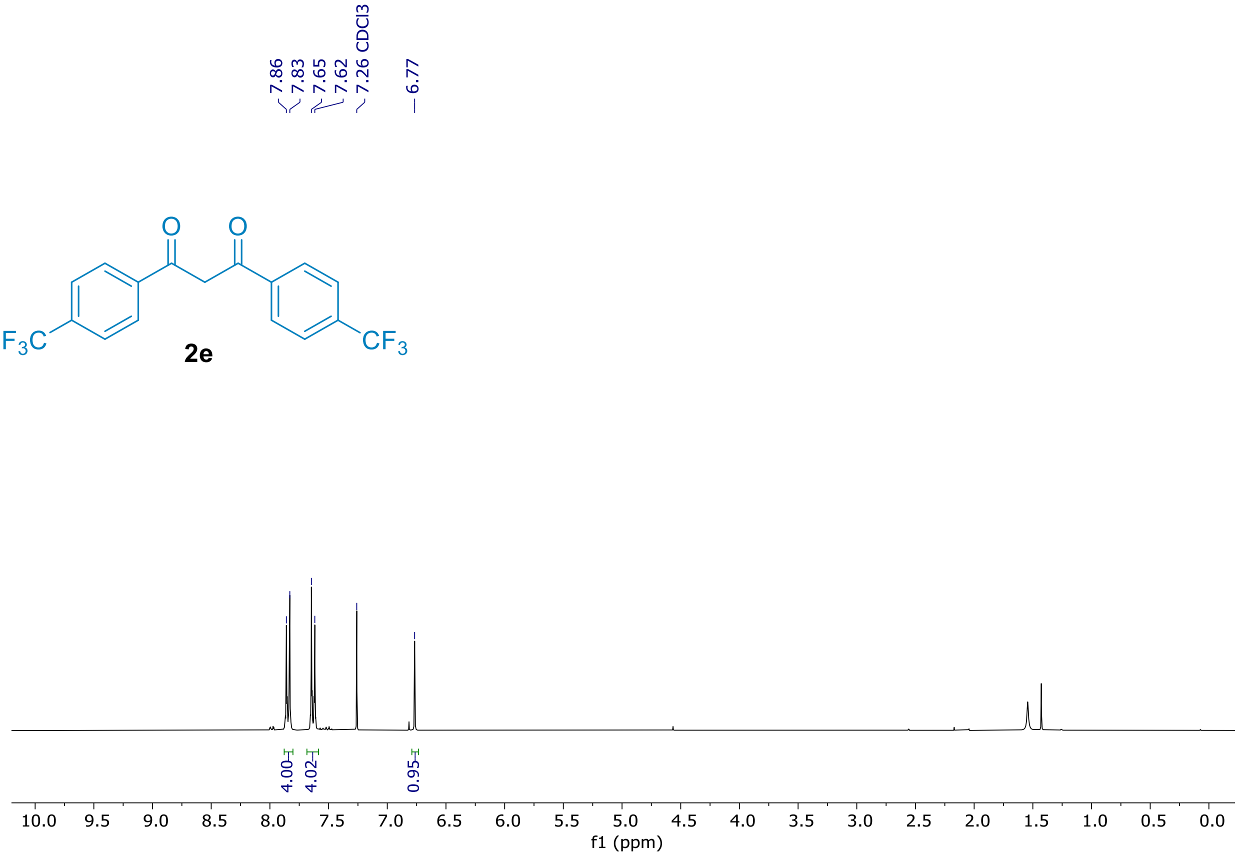

Figure S10. ^{1}H NMR spectrum of **2e** in $CDCl_3$ (300 MHz)

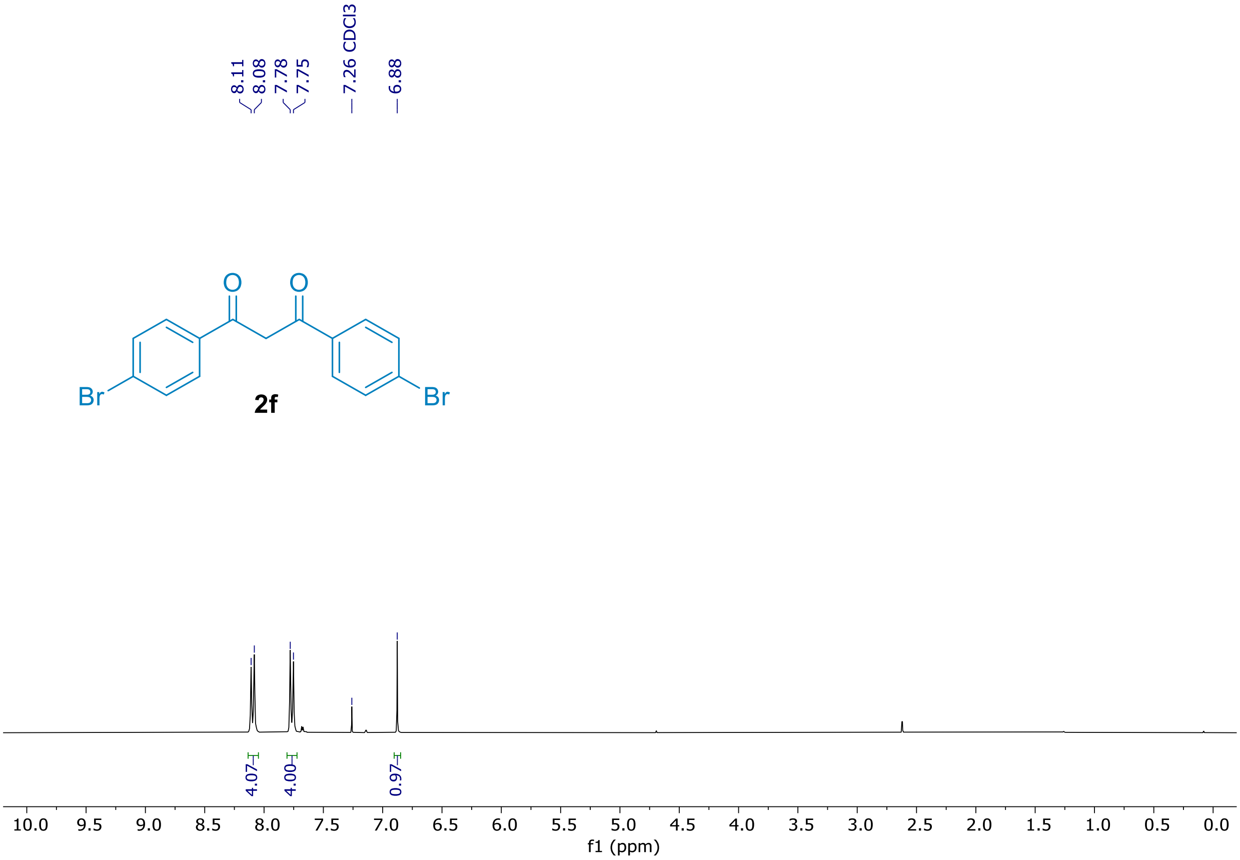

Figure S11. ^{1}H NMR spectrum of **2f** in $CDCl_3$ (500 MHz)

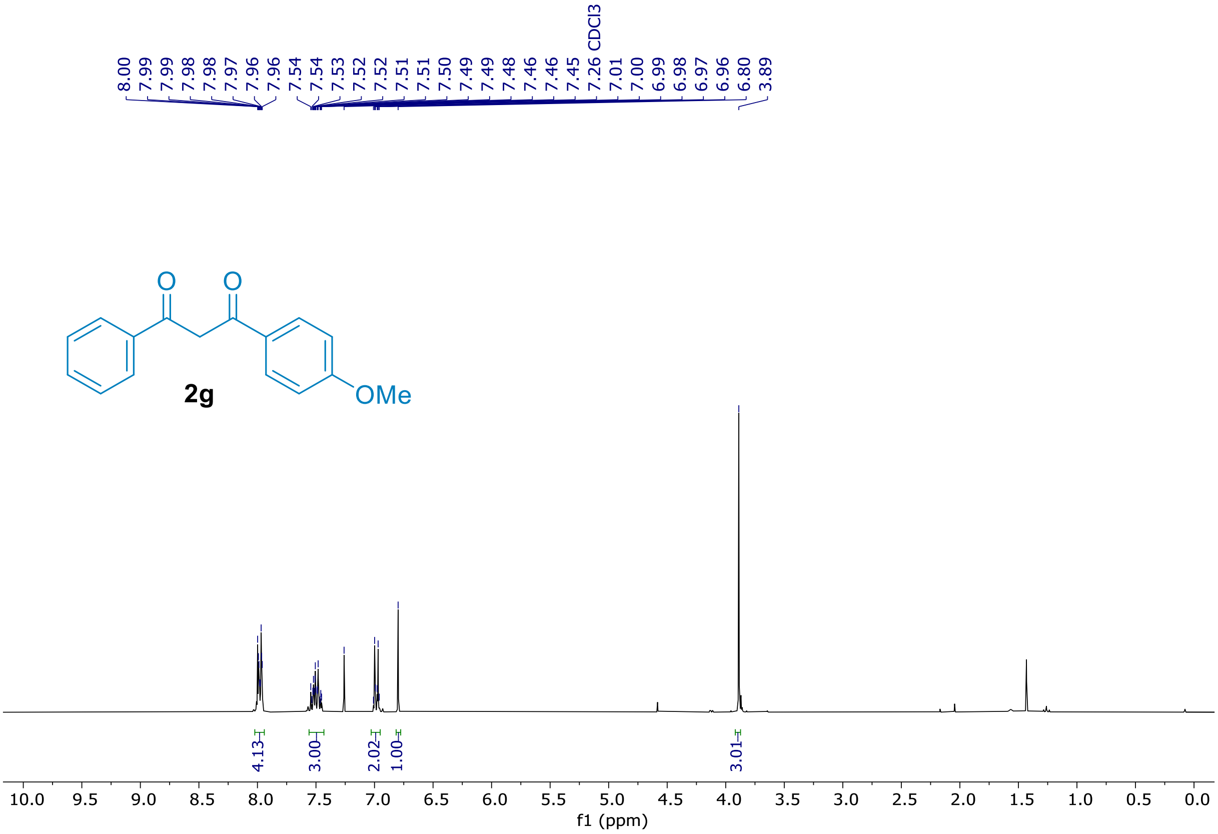

Figure S12. ^{1}H NMR spectrum of **2g** in $CDCl_3$ (500 MHz)

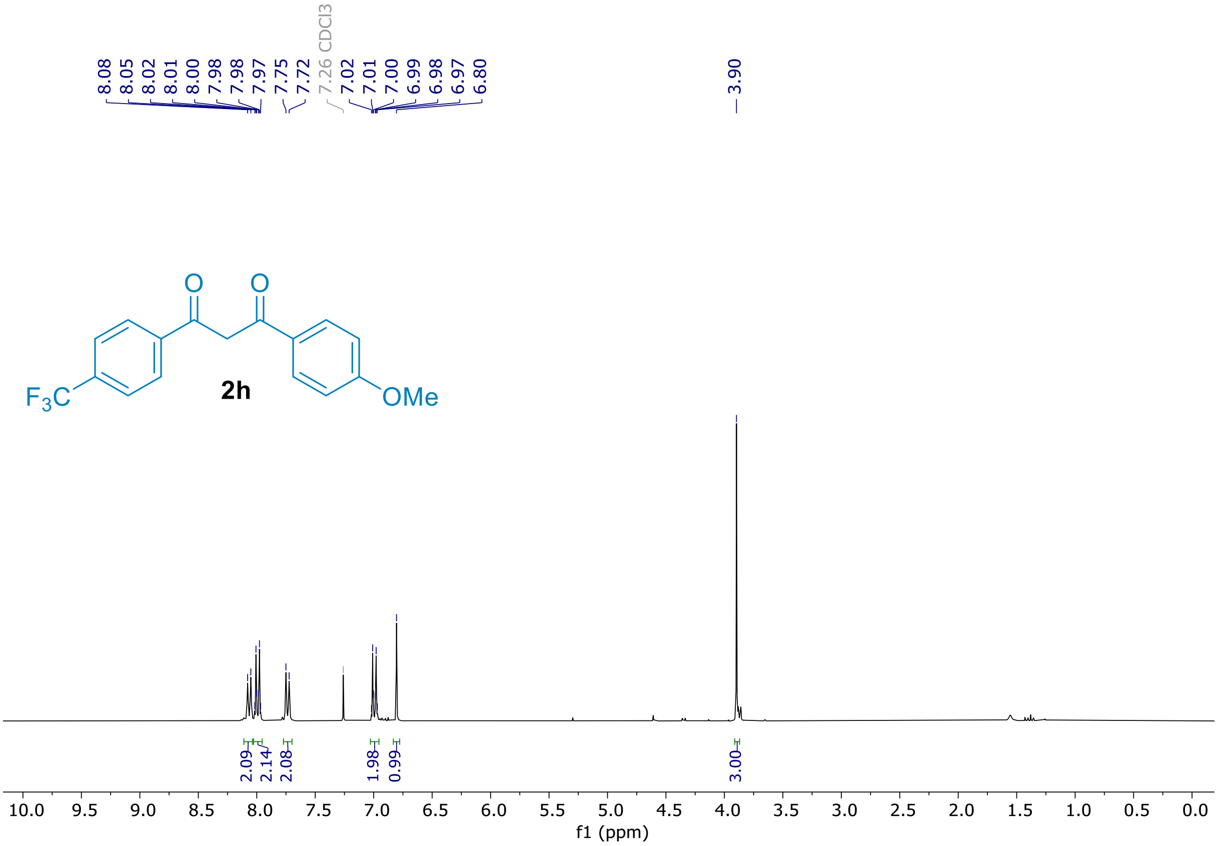

Figure S13. ^{1}H NMR spectrum of **2h** in $CDCl_3$ (300 MHz)

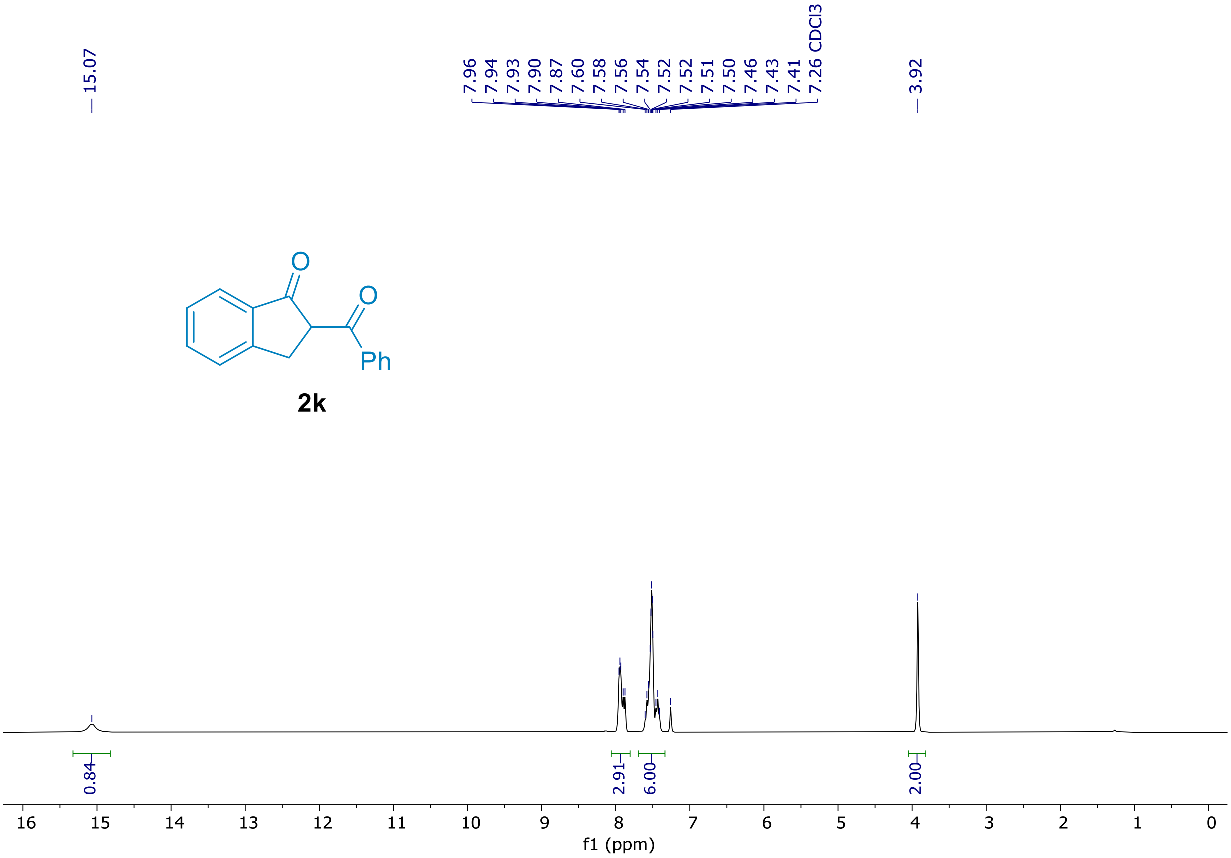

Figure S14. ^{1}H NMR spectrum of **2k** in $CDCl_3$ (300 MHz)

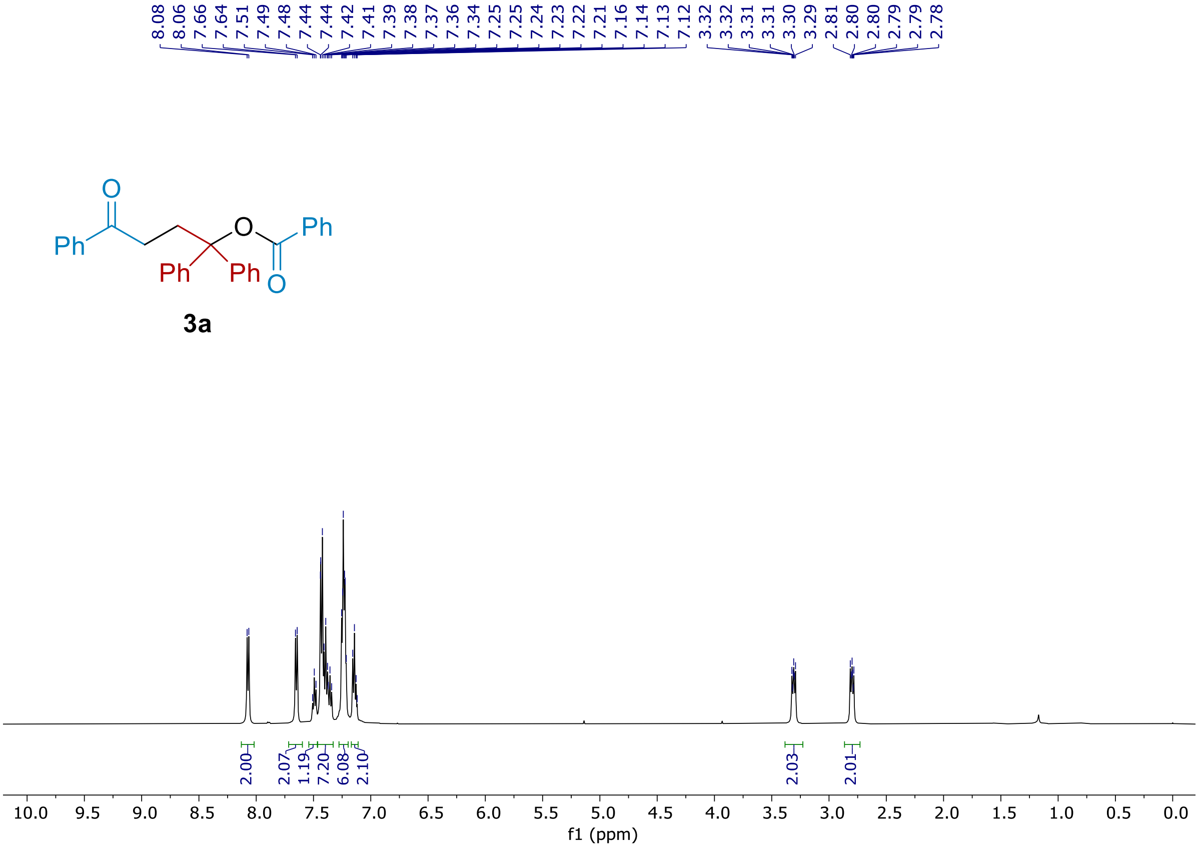

Figure S15. ^{1}H NMR spectrum of **3a** in $CDCl_3$ (500 MHz)

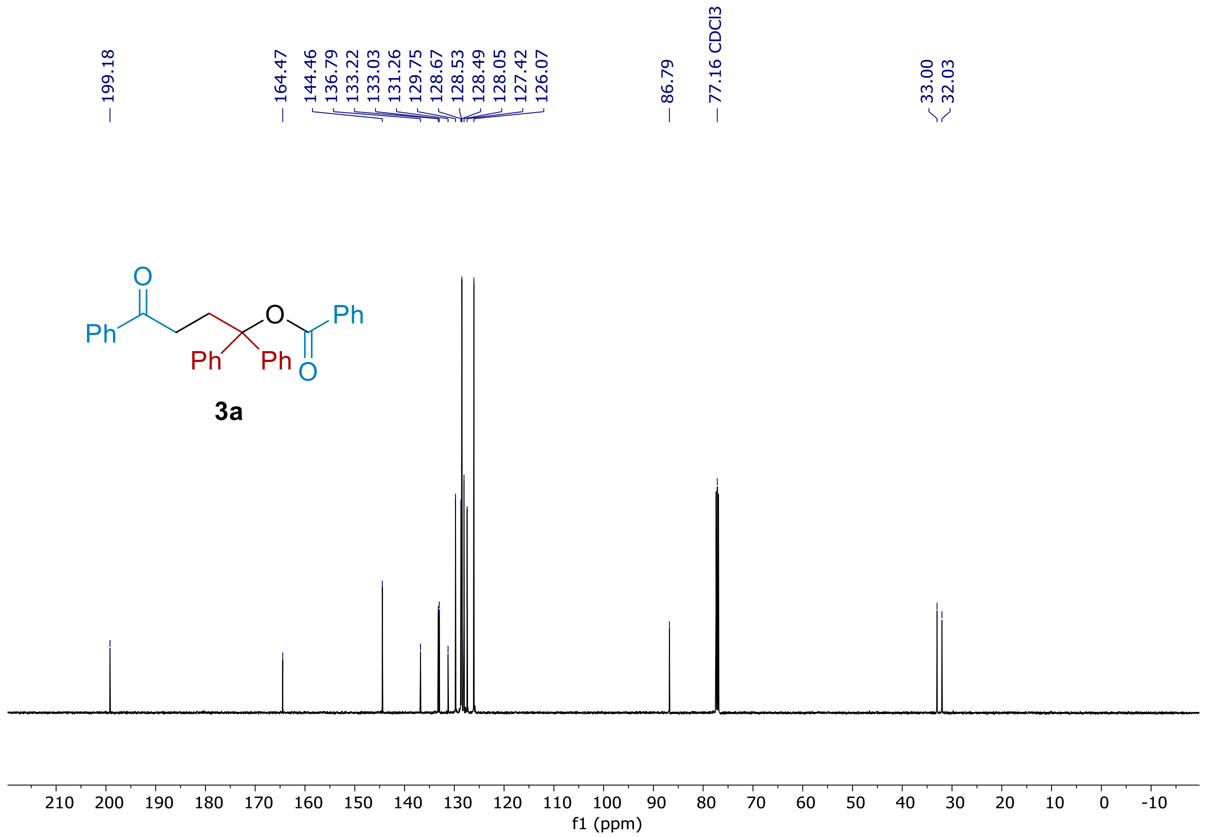

Figure S16. $^{13}C\{^1H\}$ NMR spectrum of **3a** in $CDCl_3$ (125 MHz)

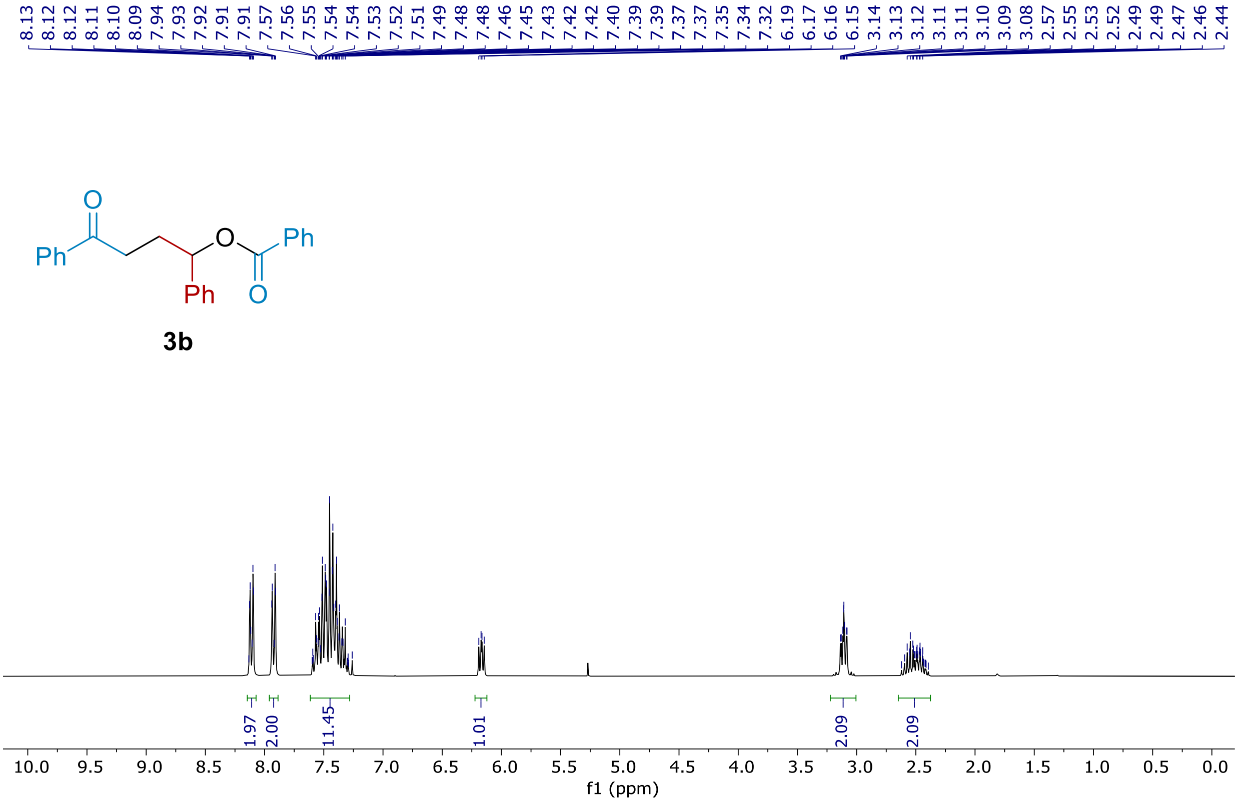

Figure S17. ^1H NMR spectrum of **3b** in $CDCl_3$ (300 MHz)

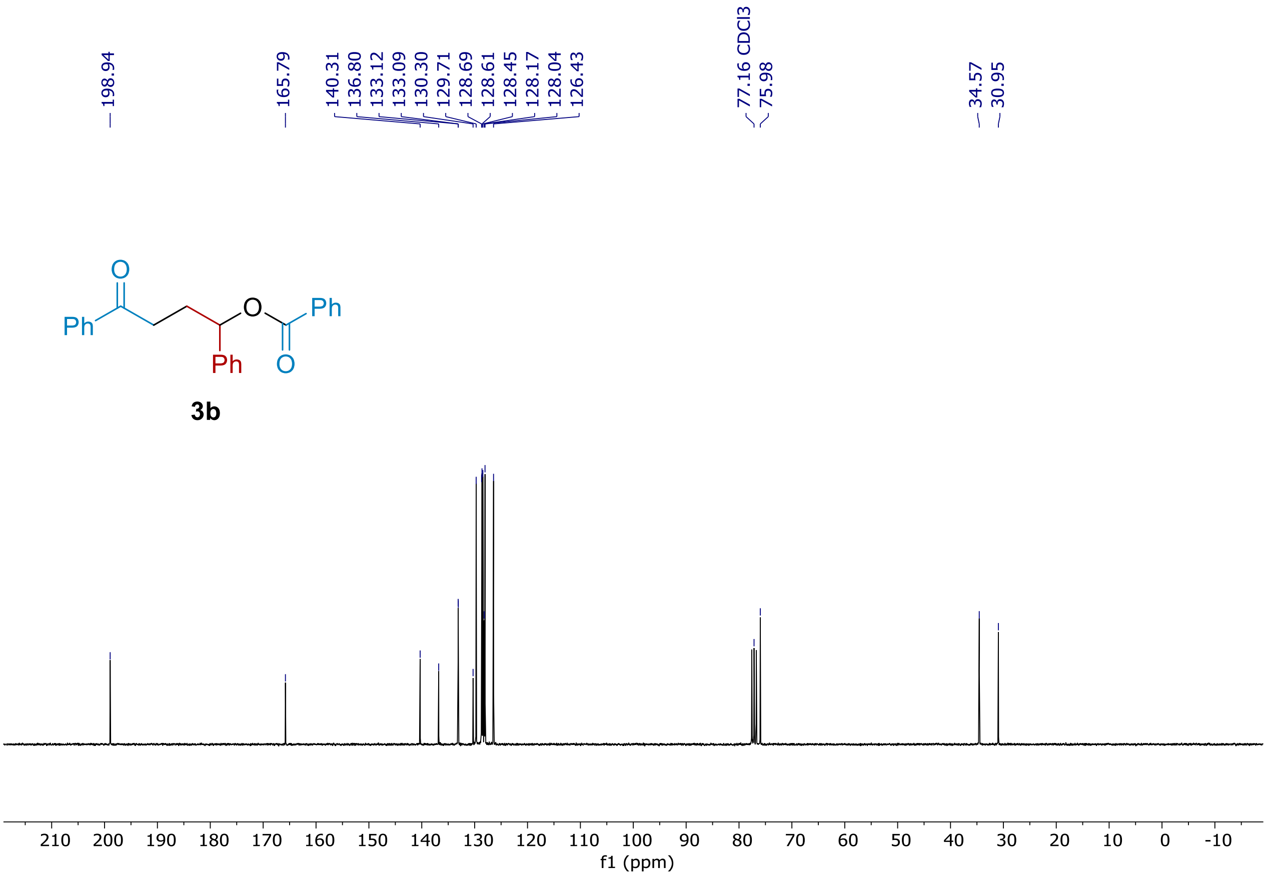

Figure S18. $^{13}C\{^{1}H\}$ NMR spectrum of **3b** in $CDCl_3$ (75 MHz)

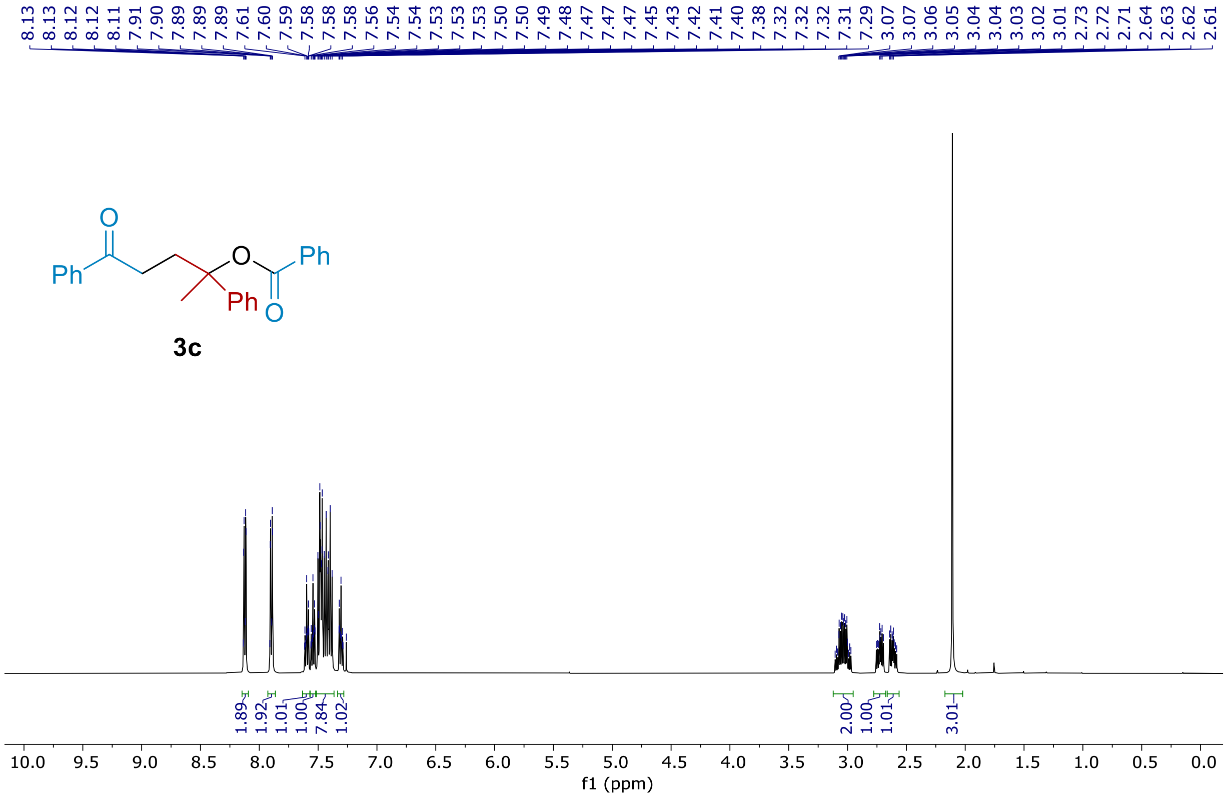

Figure S19. ^{1}H NMR spectrum of **3c** in $CDCl_3$ (500 MHz)

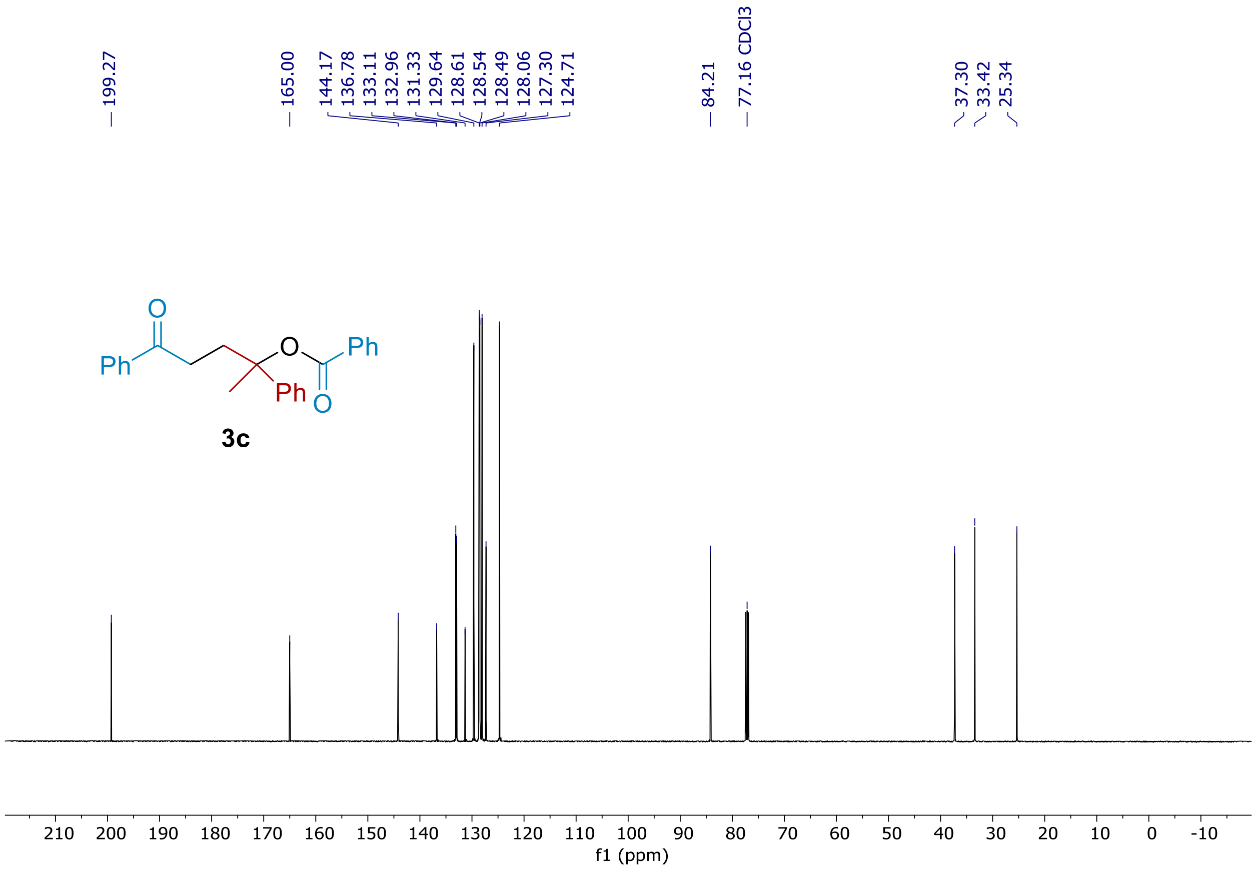

Figure S20. $^{13}C\{^{1}H\}$ NMR spectrum of **3c** in $CDCl_3$ (125 MHz)

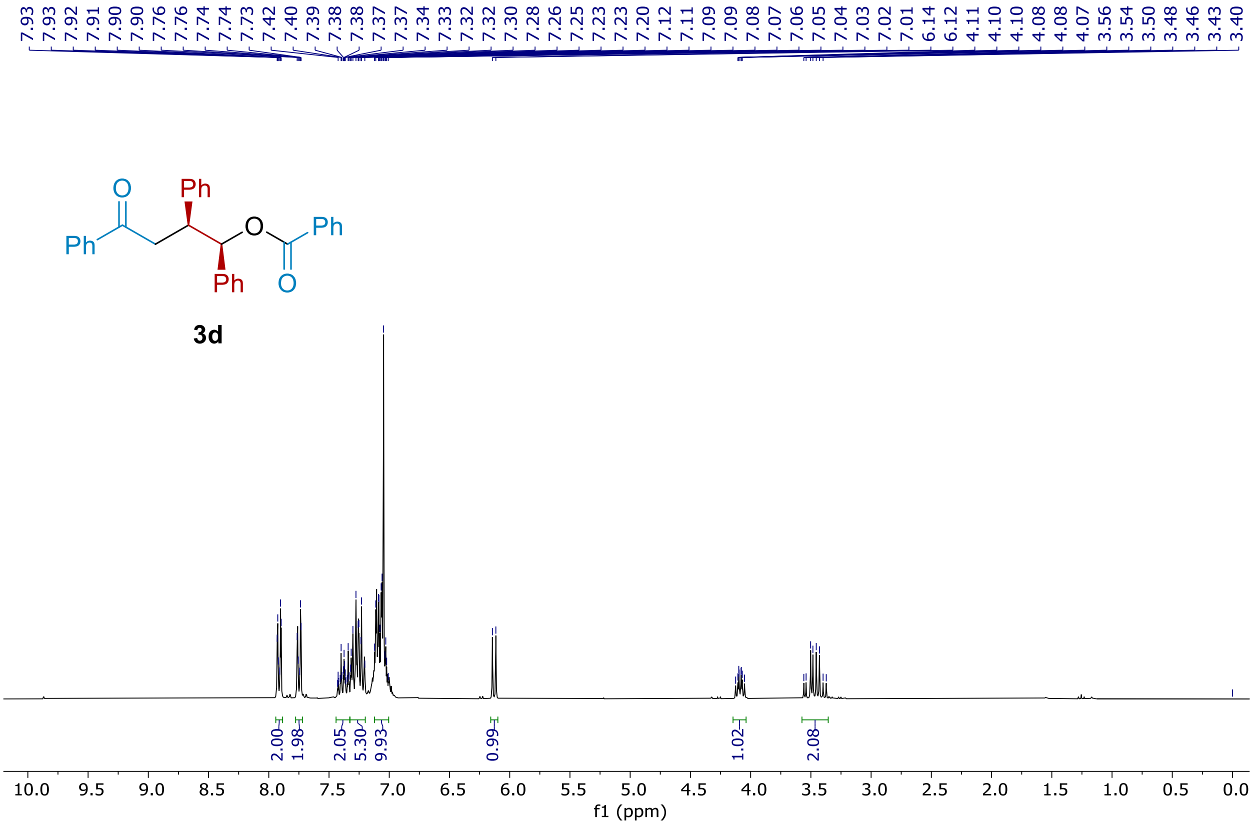

Figure S21. ^{1}H NMR spectrum of **3d** in $CDCl_3$ (300 MHz)

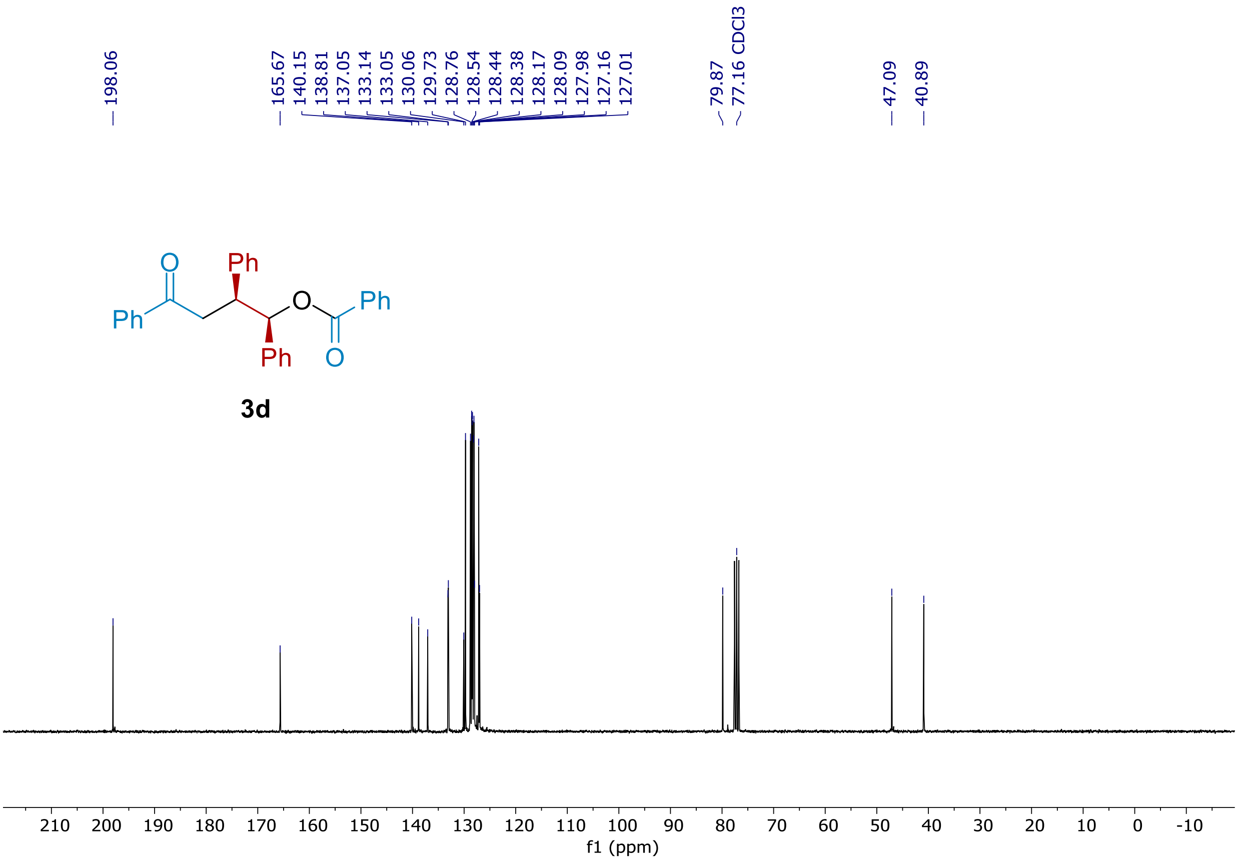

Figure S22. $^{13}C\{^{1}H\}$ NMR spectrum of **3d** in $CDCl_3$ (75 MHz)

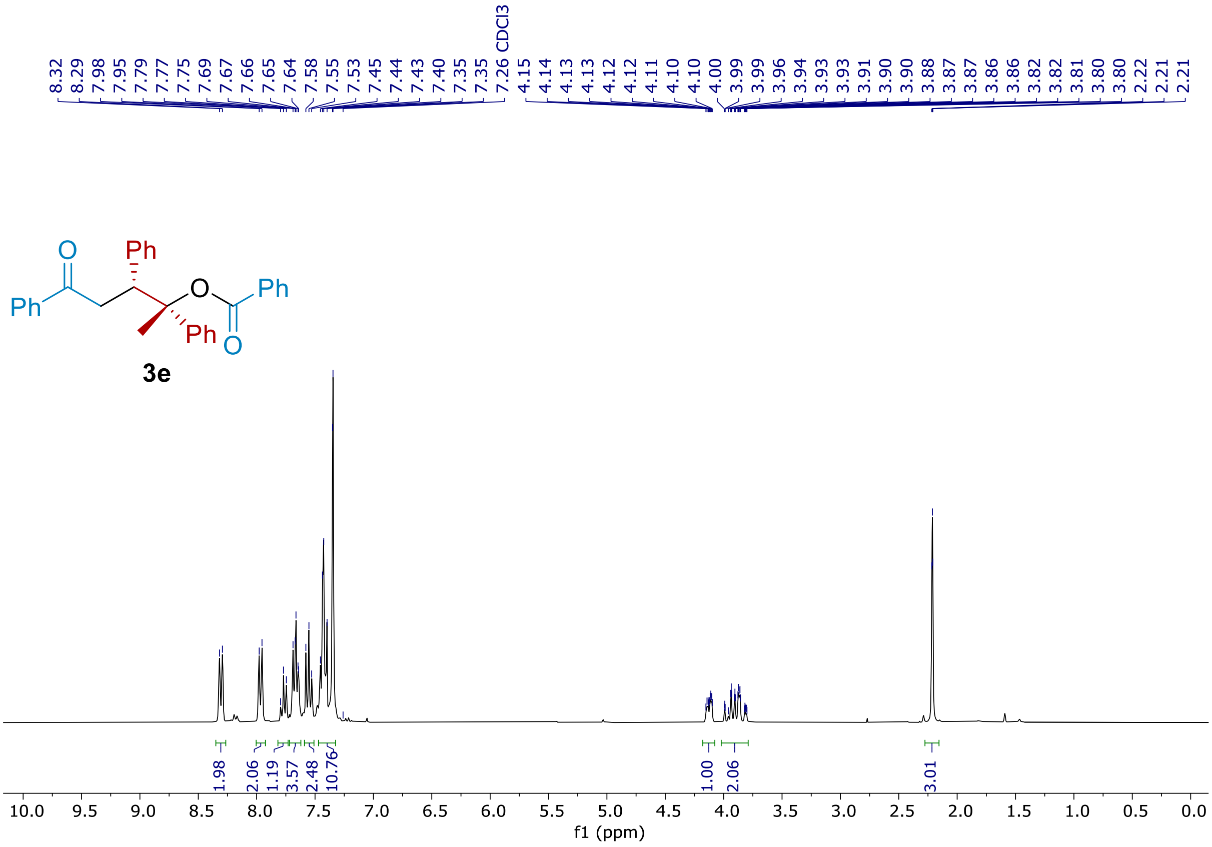

Figure S23. ^{1}H NMR spectrum of **3e** in $CDCl_3$ (300 MHz)

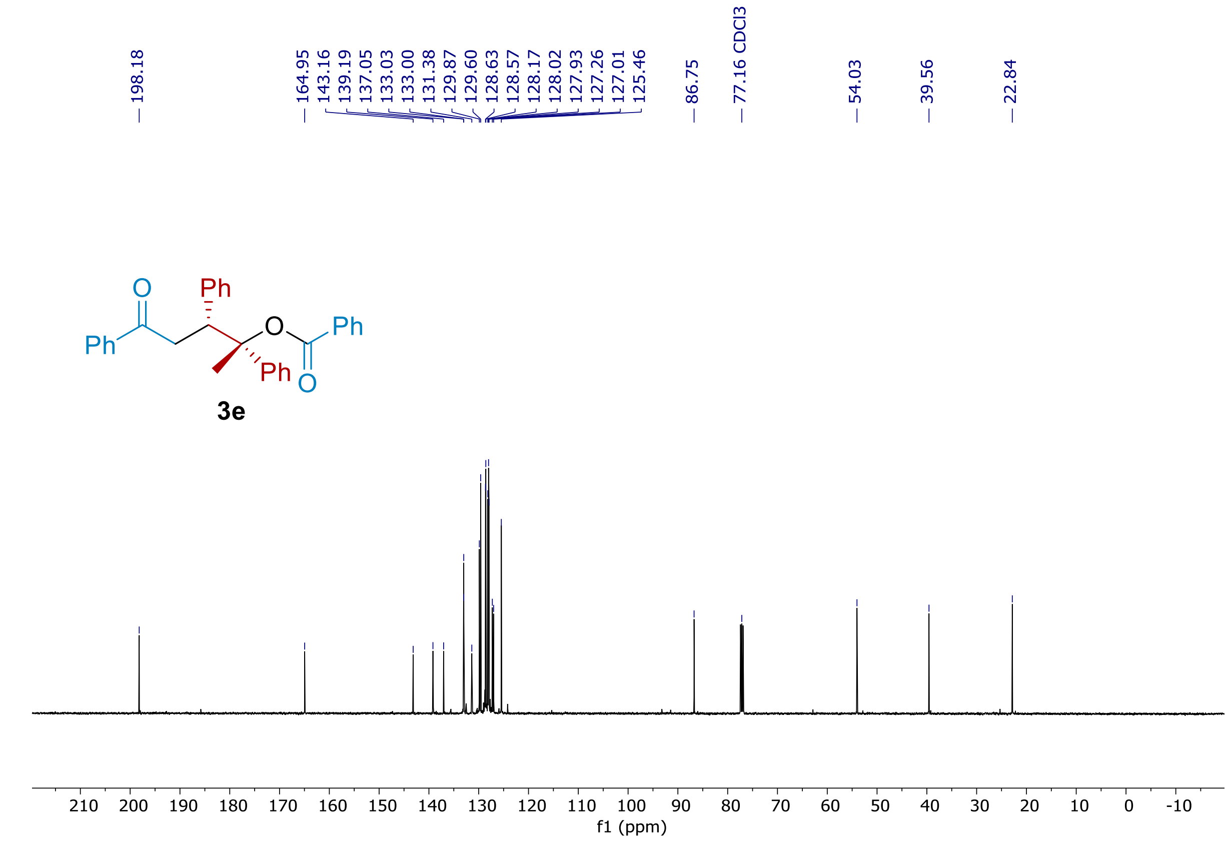

Figure S24. $^{13}C\{^{1}H\}$ NMR spectrum of **3e** in $CDCl_3$ (125 MHz)

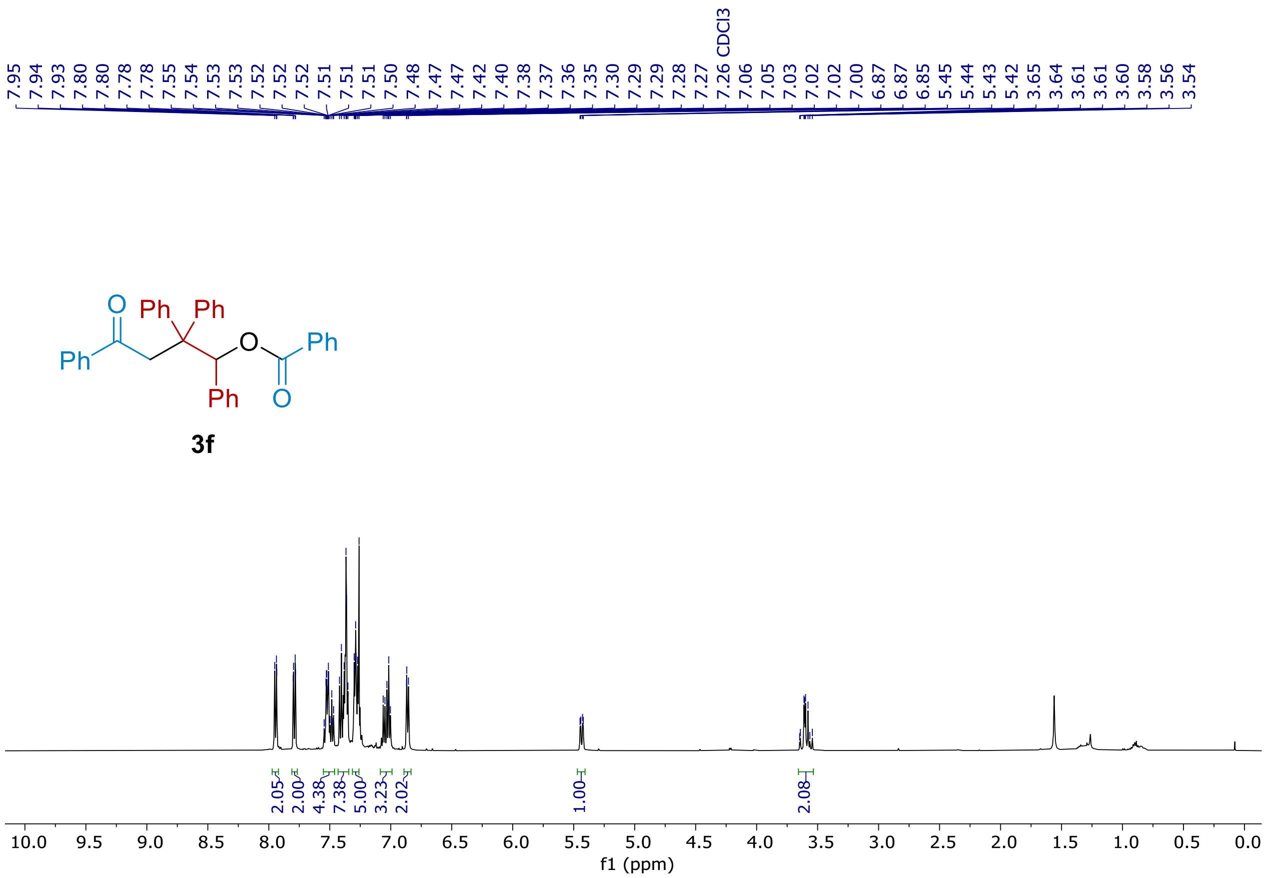

Figure S25. ^{1}H NMR spectrum of **3f** in $CDCl_3$ (500 MHz)

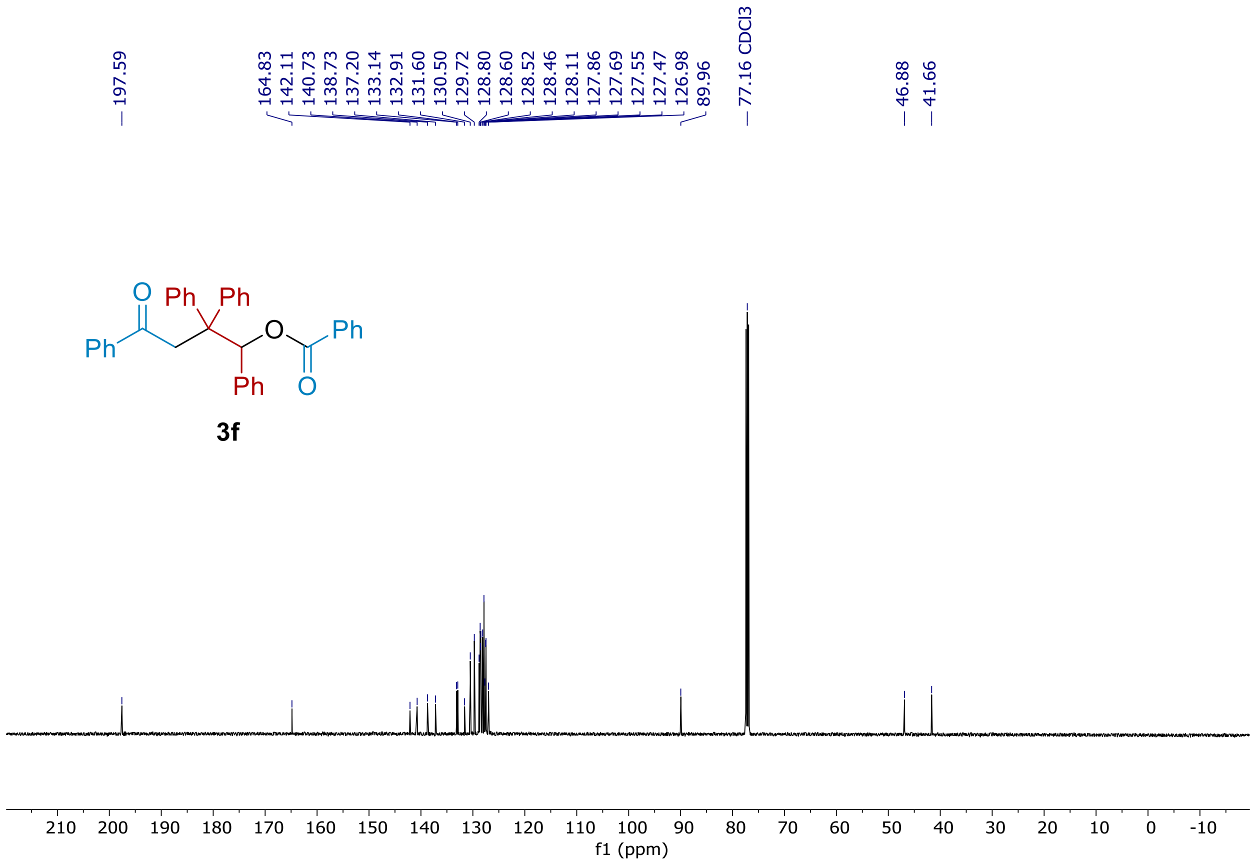

Figure S26. $^{13}C\{^{1}H\}$ NMR spectrum of **3f** in $CDCl_3$ (125 MHz)

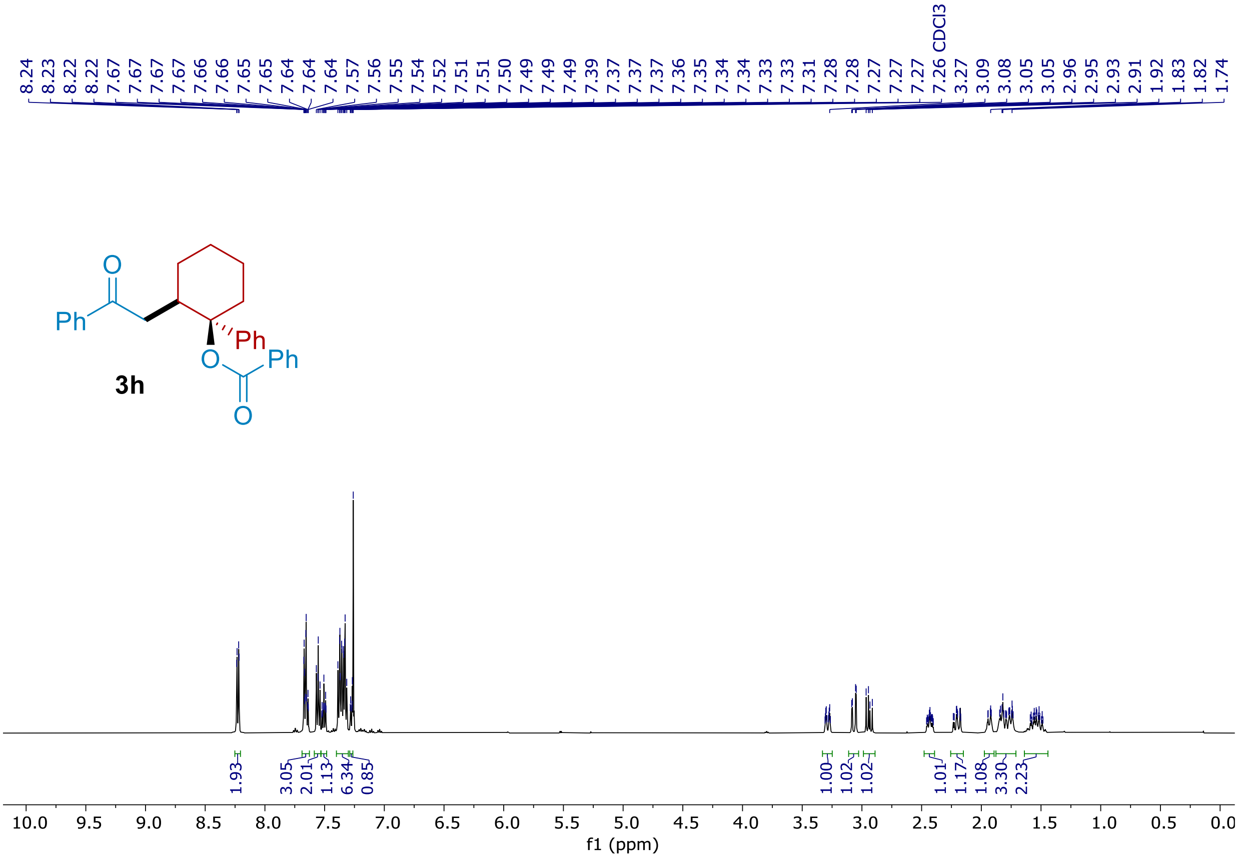

Figure S27. ^{1}H NMR spectrum of **3h** in $CDCl_3$ (500 MHz)

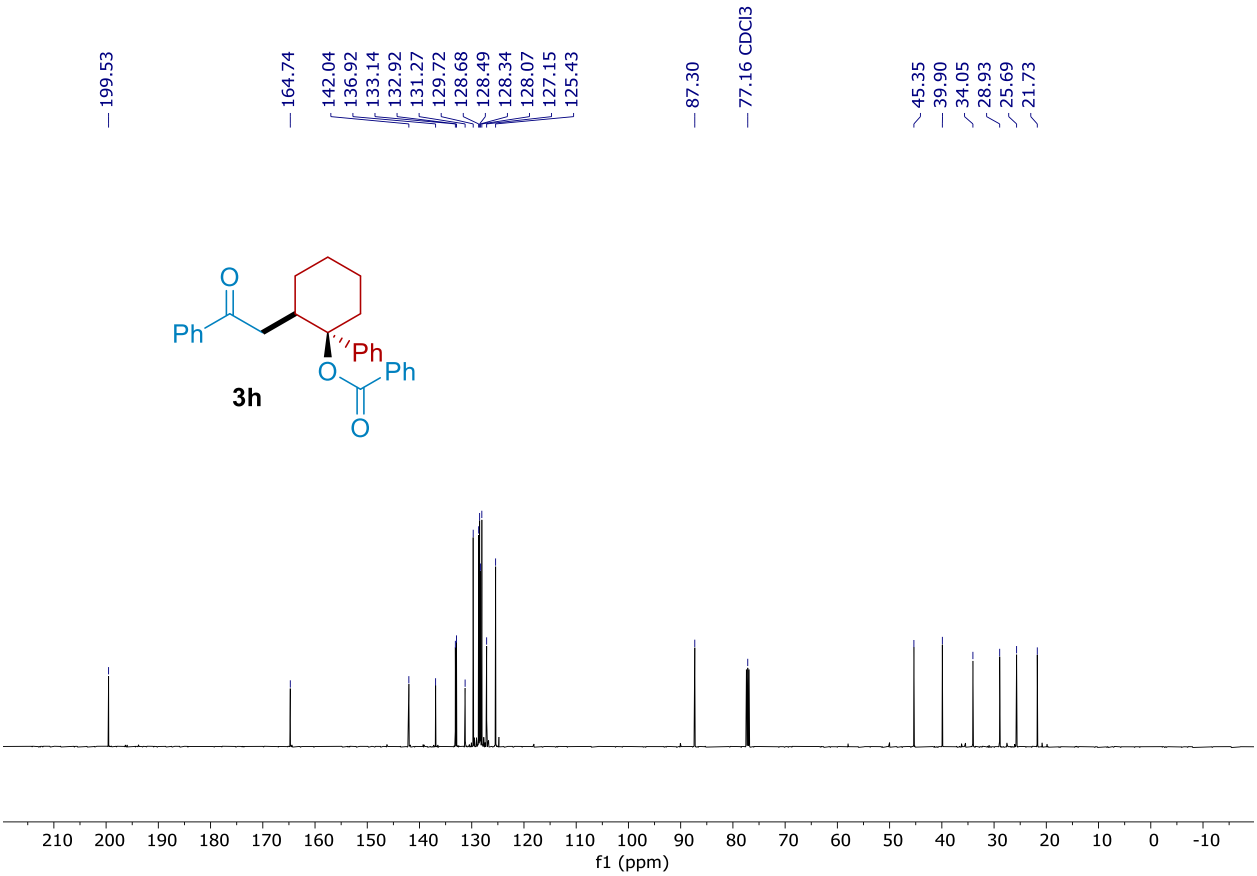

Figure S28. $^{13}C\{^{1}H\}$ NMR spectrum of **3h** in $CDCl_3$ (125 MHz)

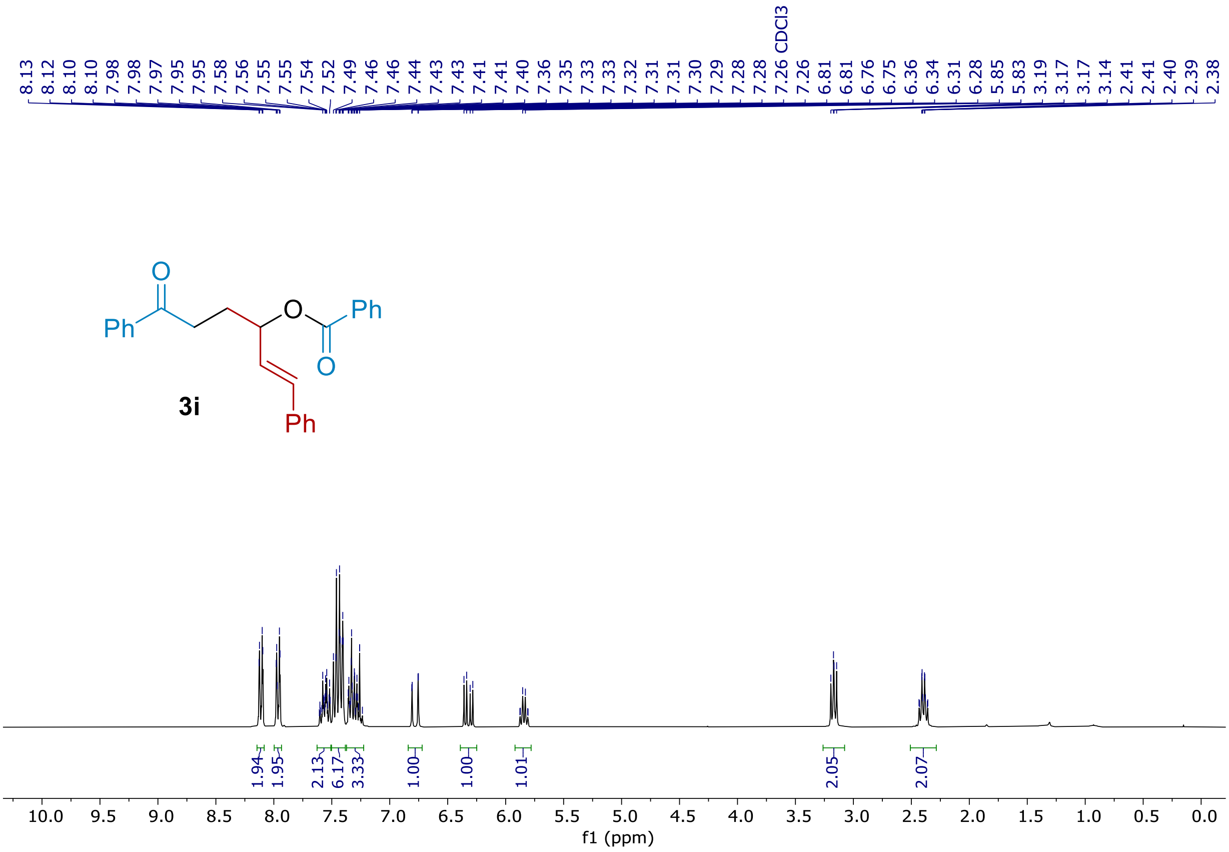

Figure S29. ^{1}H NMR spectrum of **3i** in $CDCl_3$ (300 MHz)

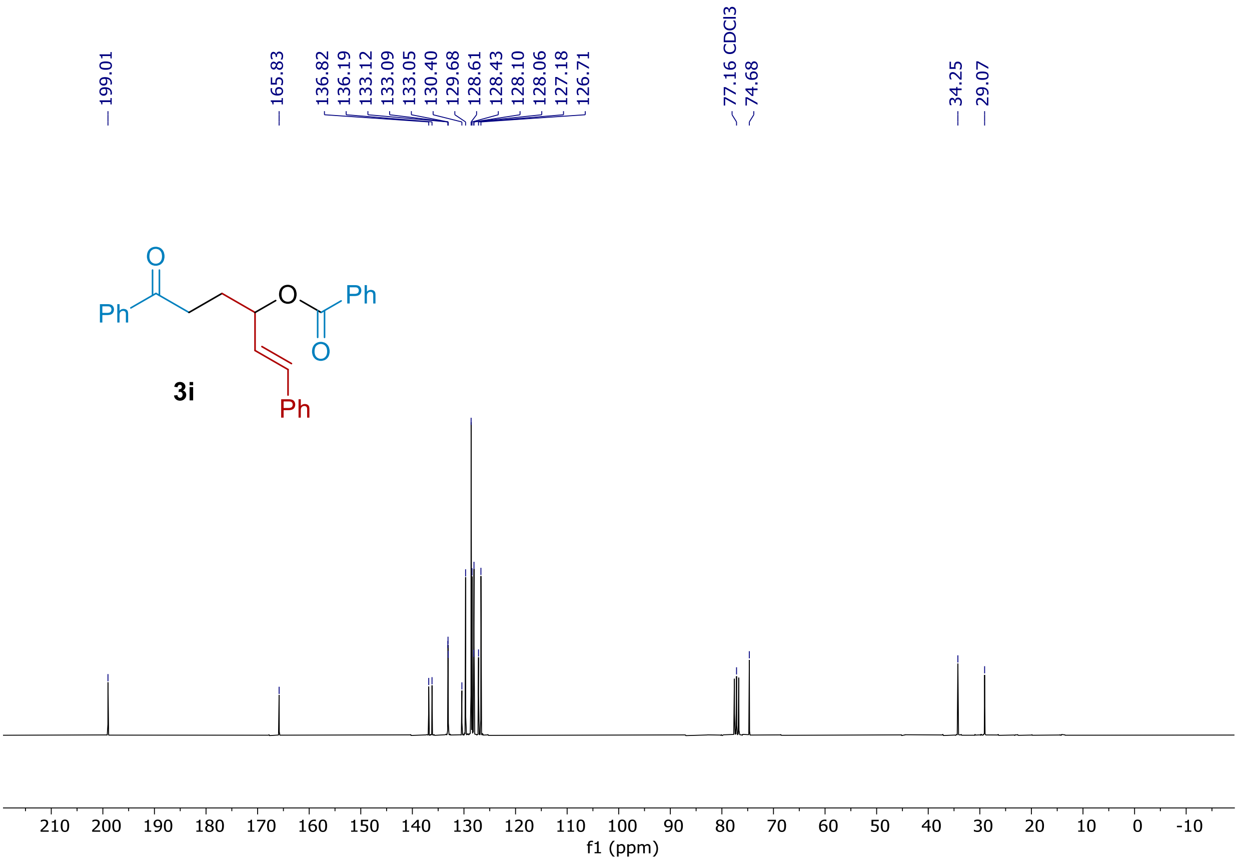

Figure S30. $^{13}C\{^{1}H\}$ NMR spectrum of **3i** in $CDCl_3$ (75 MHz)

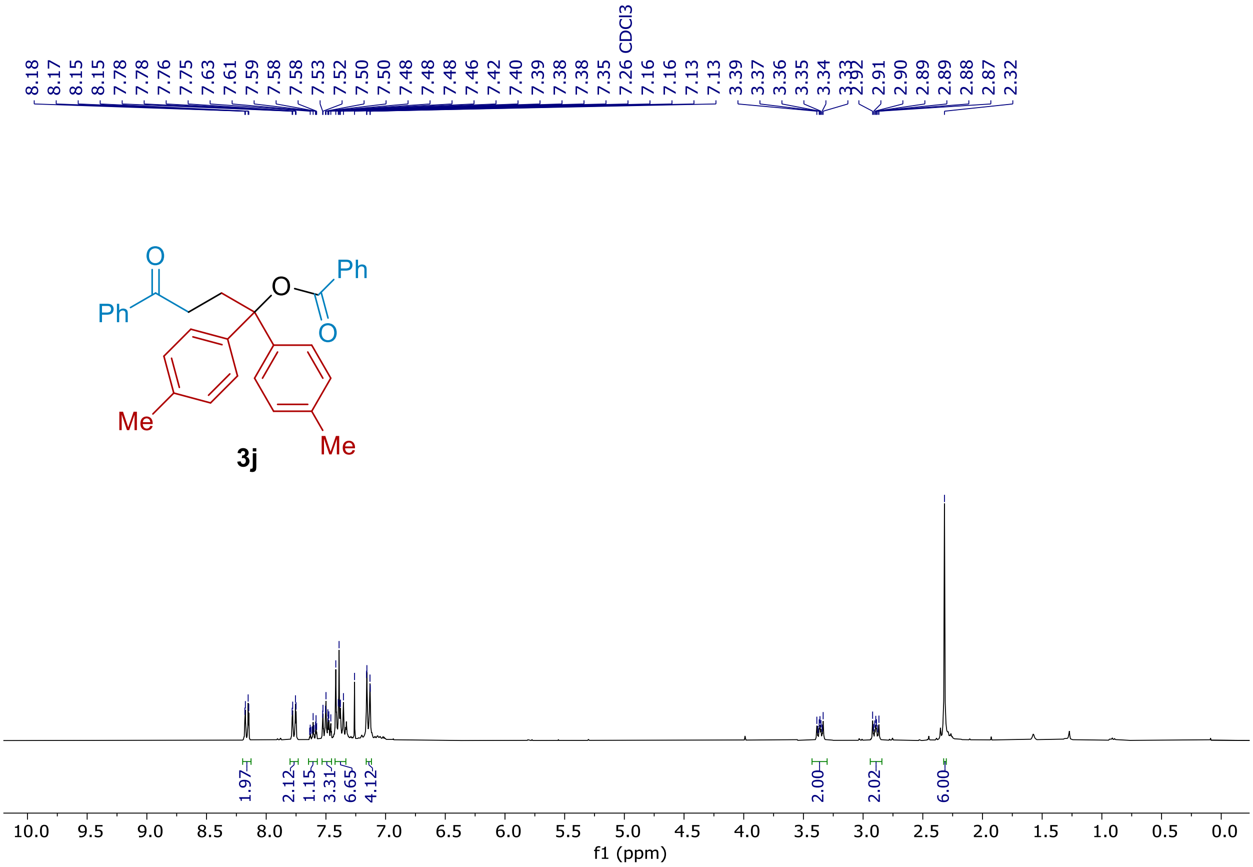

Figure S31. ^{1}H NMR spectrum of **3j** in $CDCl_3$ (300 MHz)

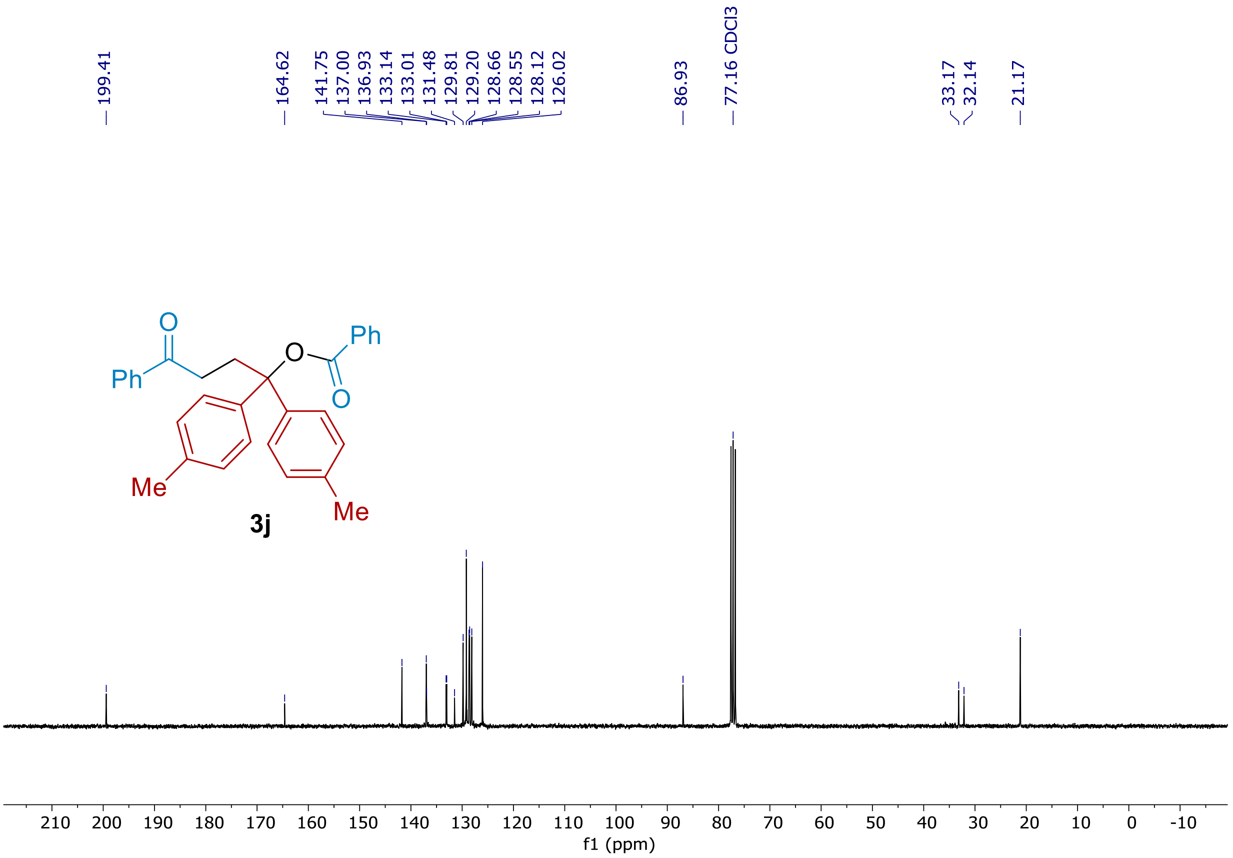

Figure S32. $^{13}C\{^{1}H\}$ NMR spectrum of **3j** in $CDCl_3$ (75 MHz)

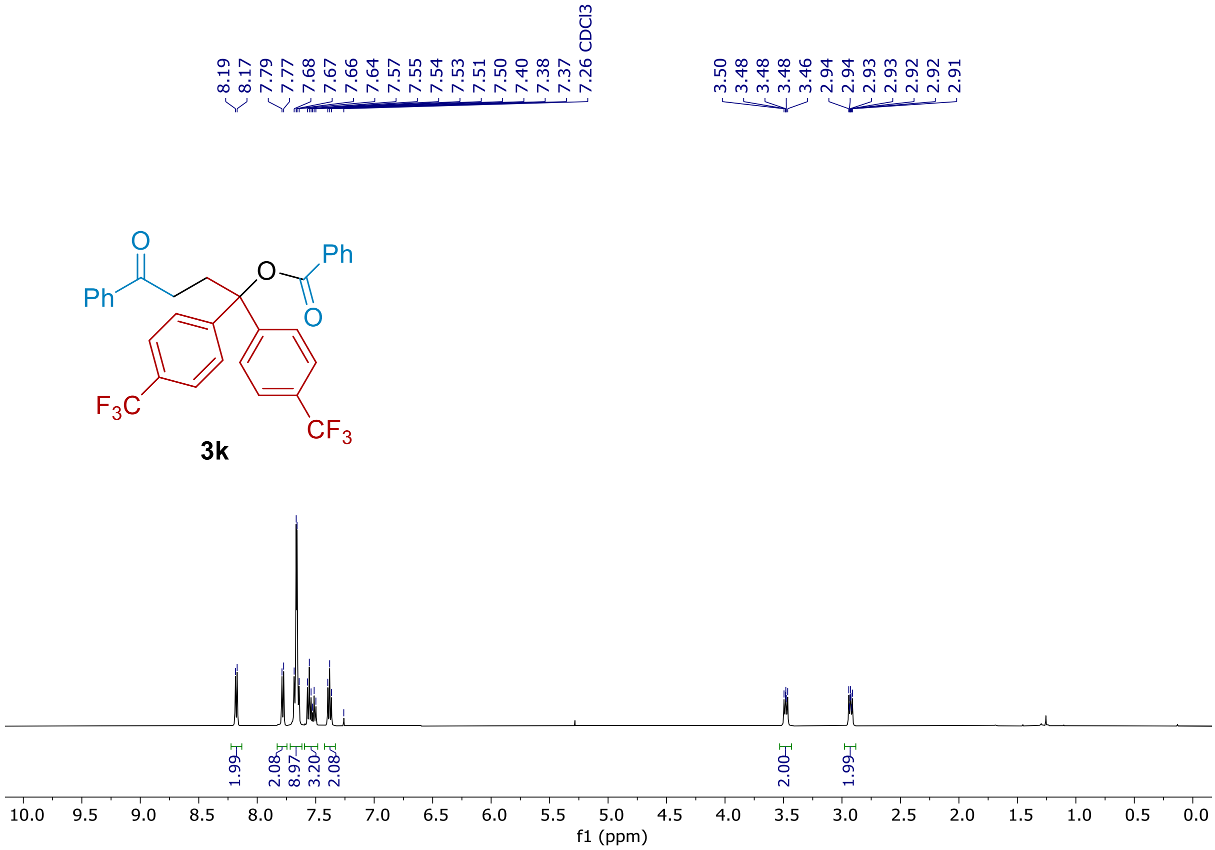

Figure S33. ^{1}H NMR spectrum of **3k** in $CDCl_3$ (500 MHz)

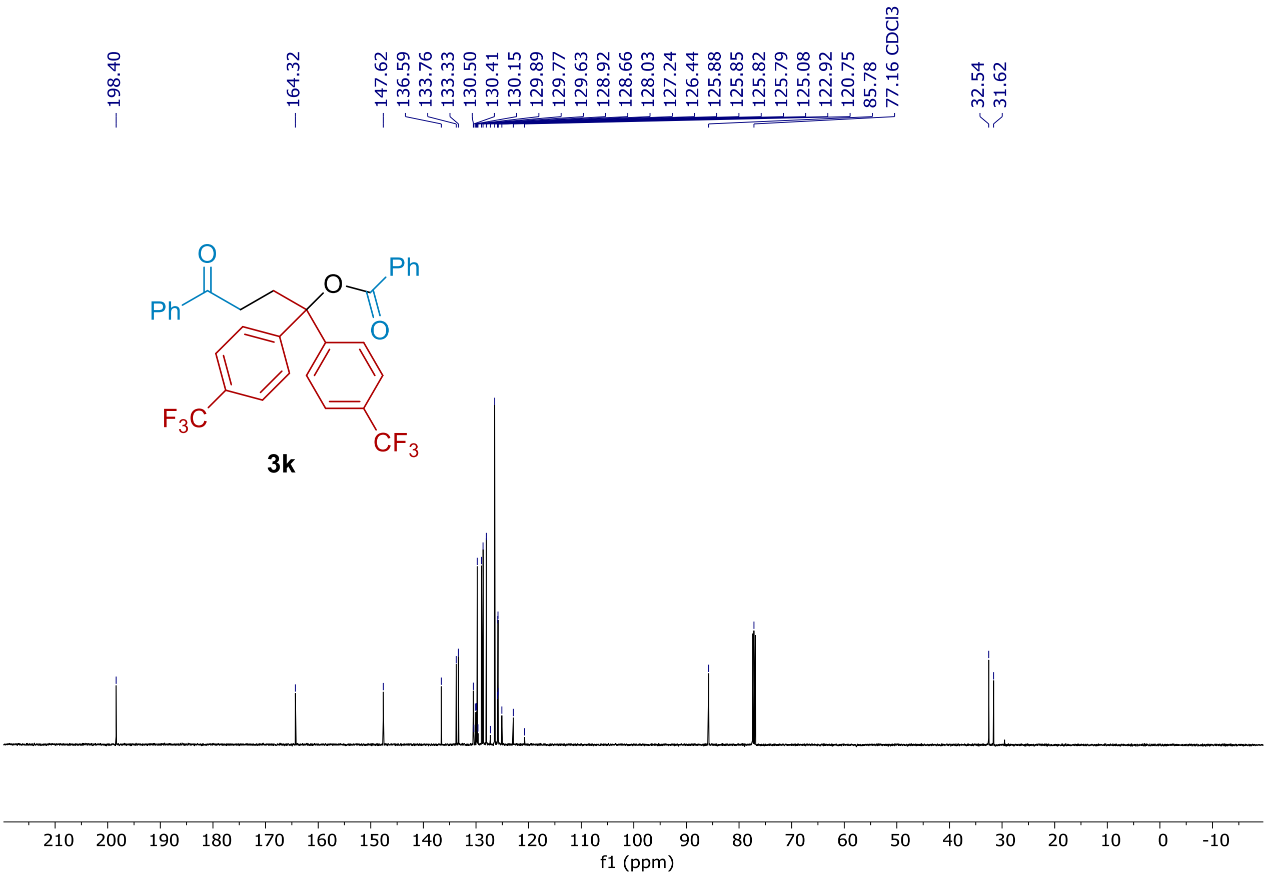

Figure S34. $^{13}C\{^1H\}$ NMR spectrum of **3k** in $CDCl_3$ (125 MHz)

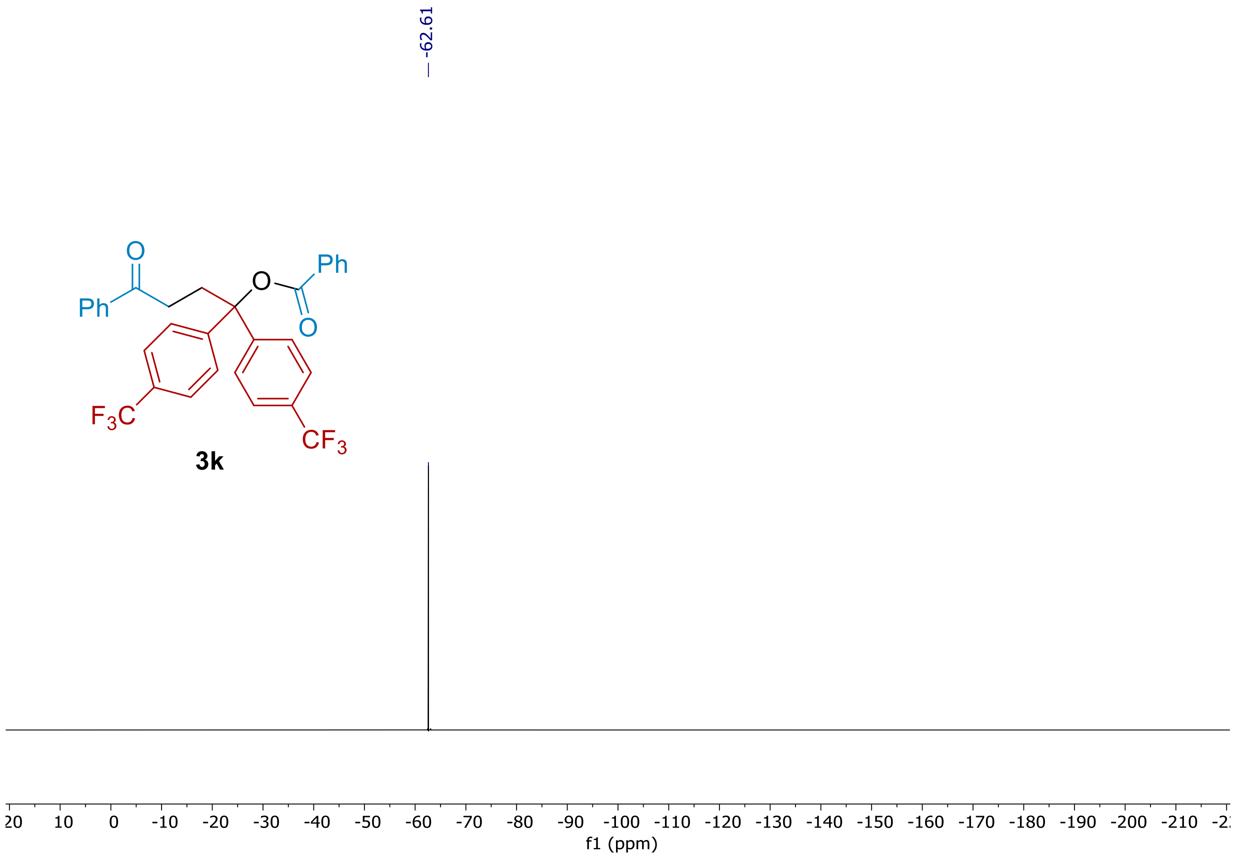

Figure S35. $^{19}F\{^1H\}$ NMR spectrum of **3k** in $CDCl_3$ (470 MHz)

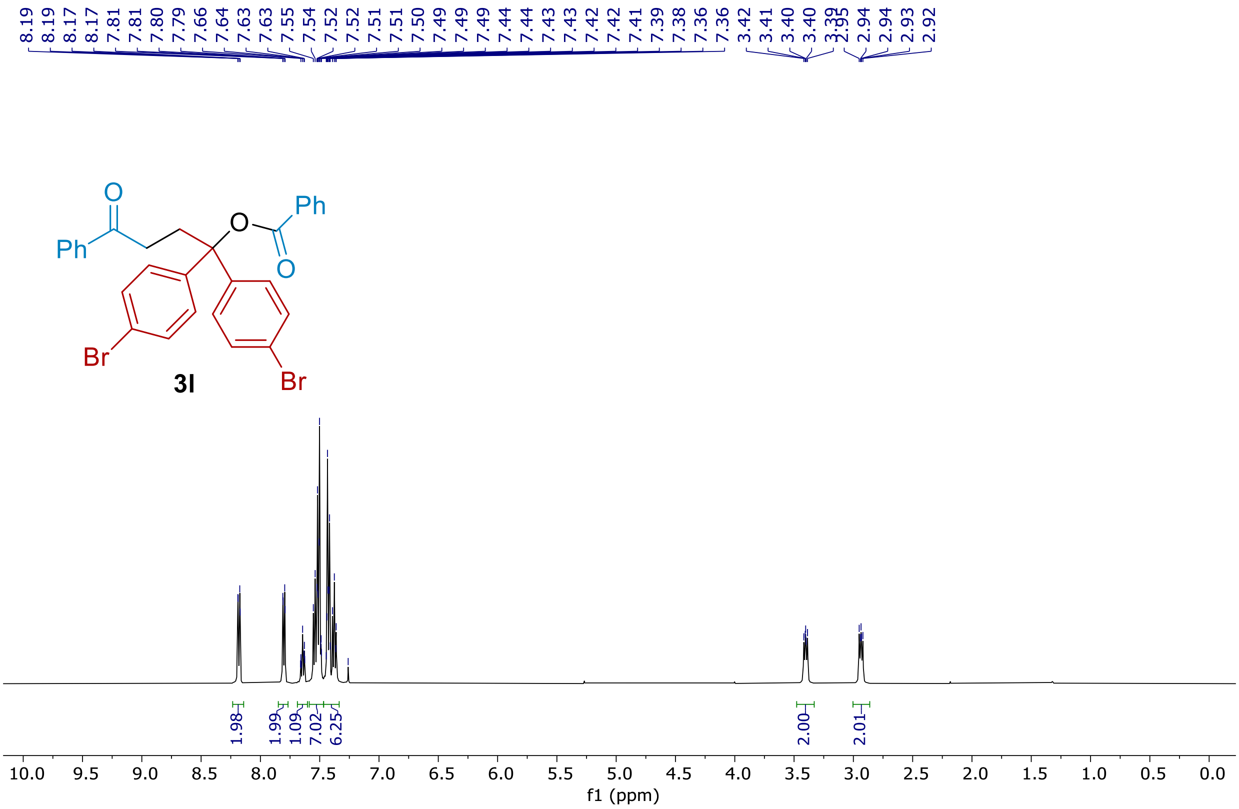

Figure S36. ^{1}H NMR spectrum of **3l** in $CDCl_3$ (500 MHz)

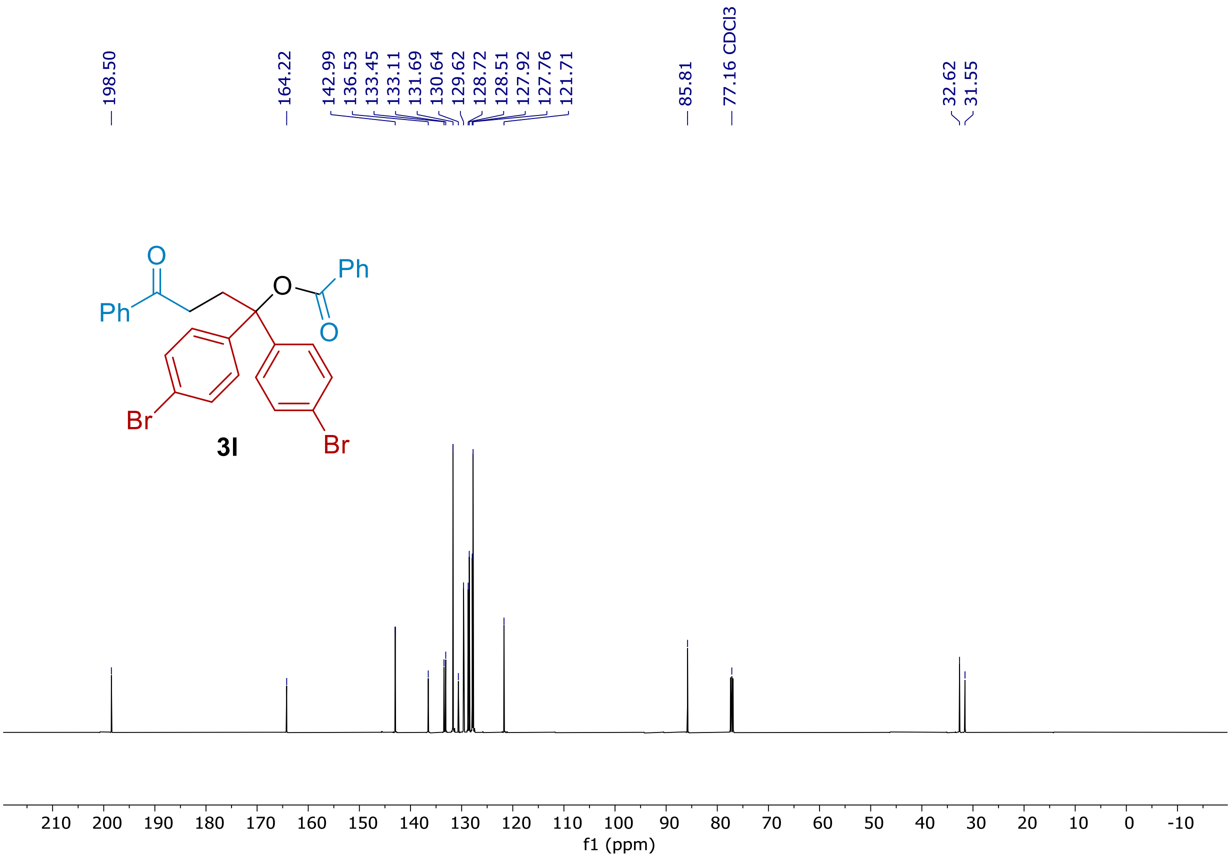

Figure S37. $^{13}C\{^{1}H\}$ NMR spectrum of **3l** in $CDCl_3$ (125 MHz)

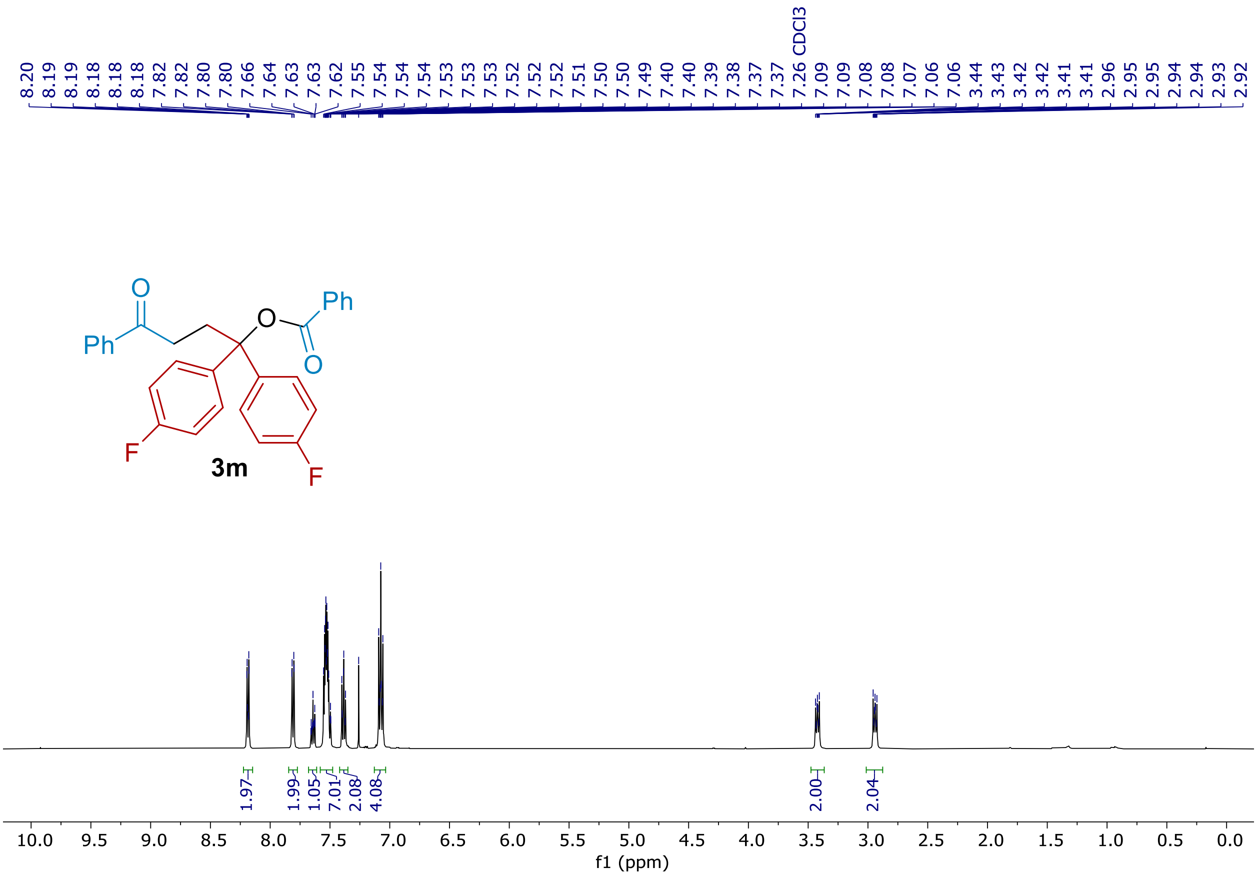

Figure S38. ^{1}H NMR spectrum of **3m** in $CDCl_3$ (500 MHz)

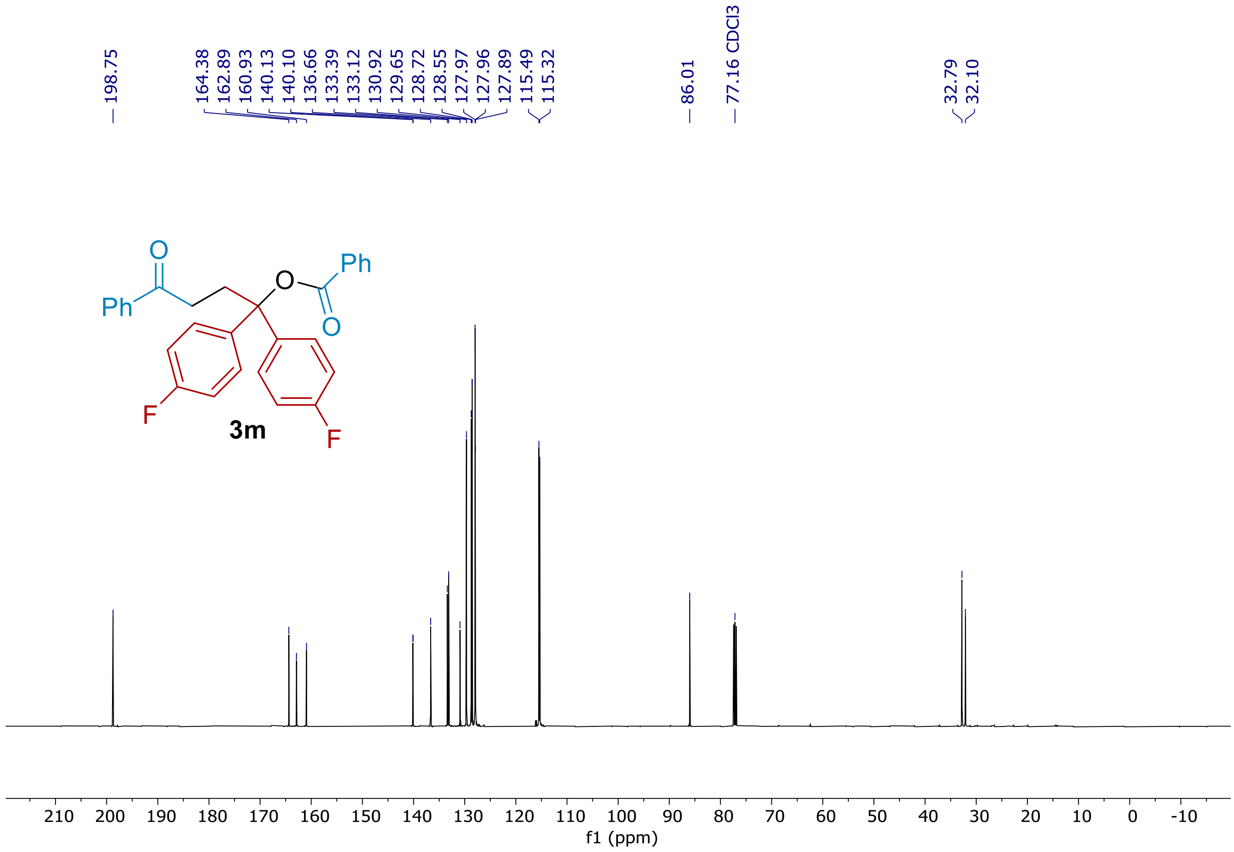

Figure S39. $^{13}C\{^{1}H\}$ NMR spectrum of **3m** in $CDCl_3$ (125 MHz)

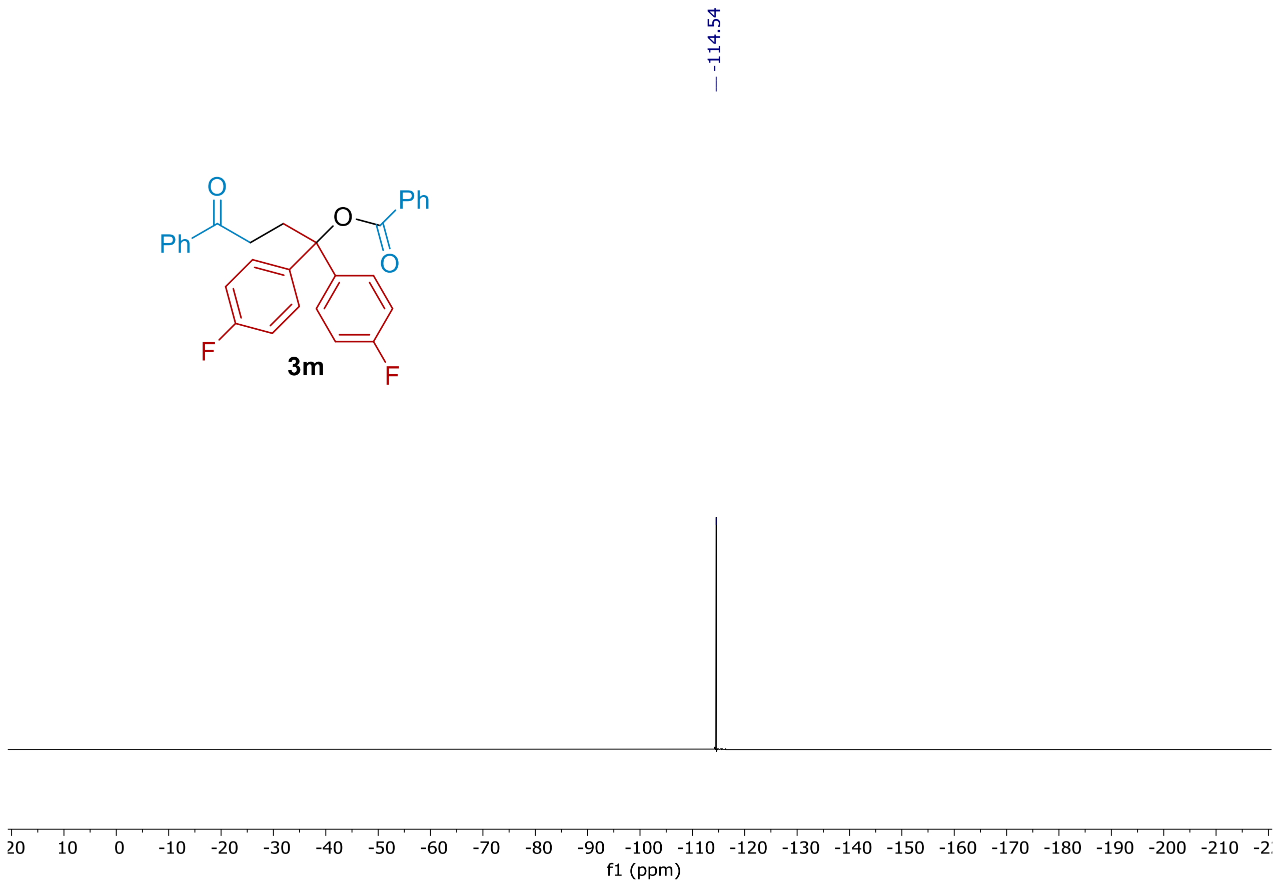

Figure S40. $^{19}F\{^{1}H\}$ NMR spectrum of **3m** in $CDCl_3$ (470 MHz)

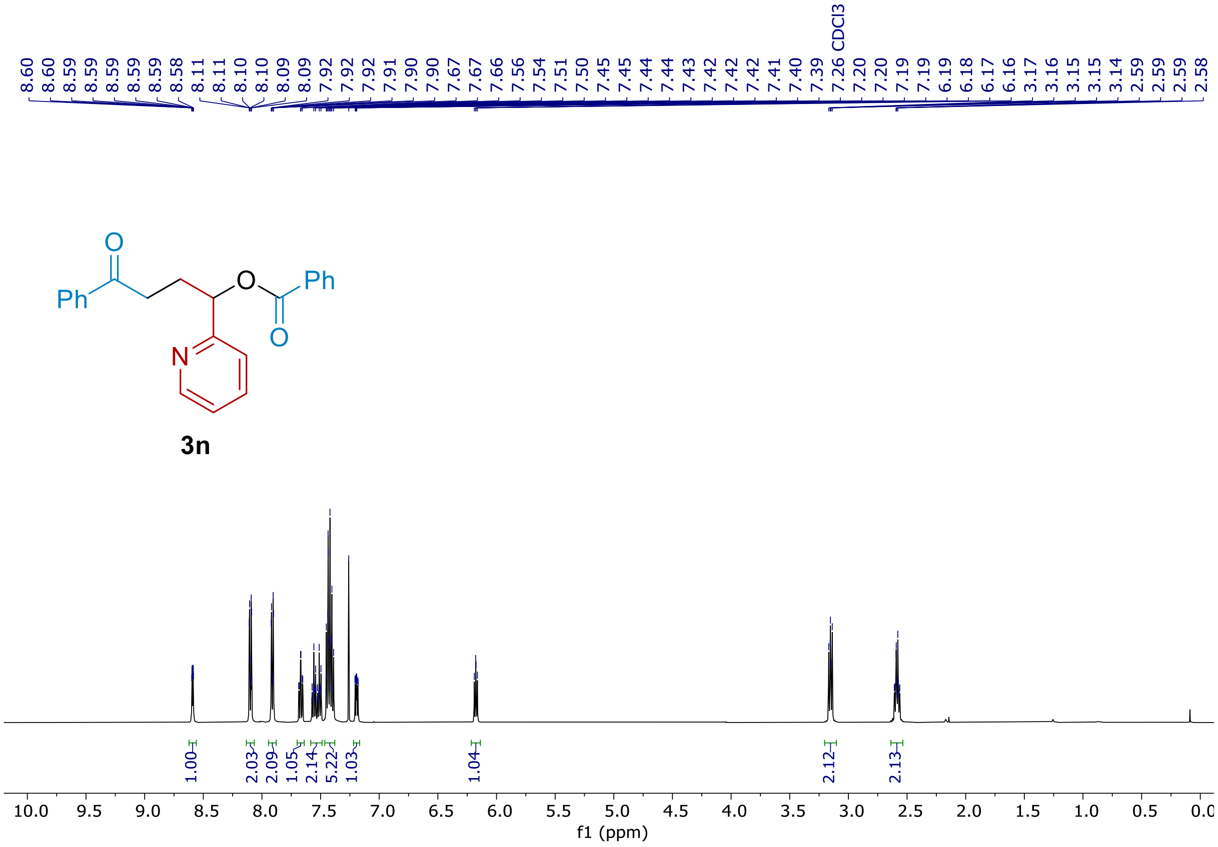

Figure S41. ^{1}H NMR spectrum of **3n** in $CDCl_3$ (500 MHz)

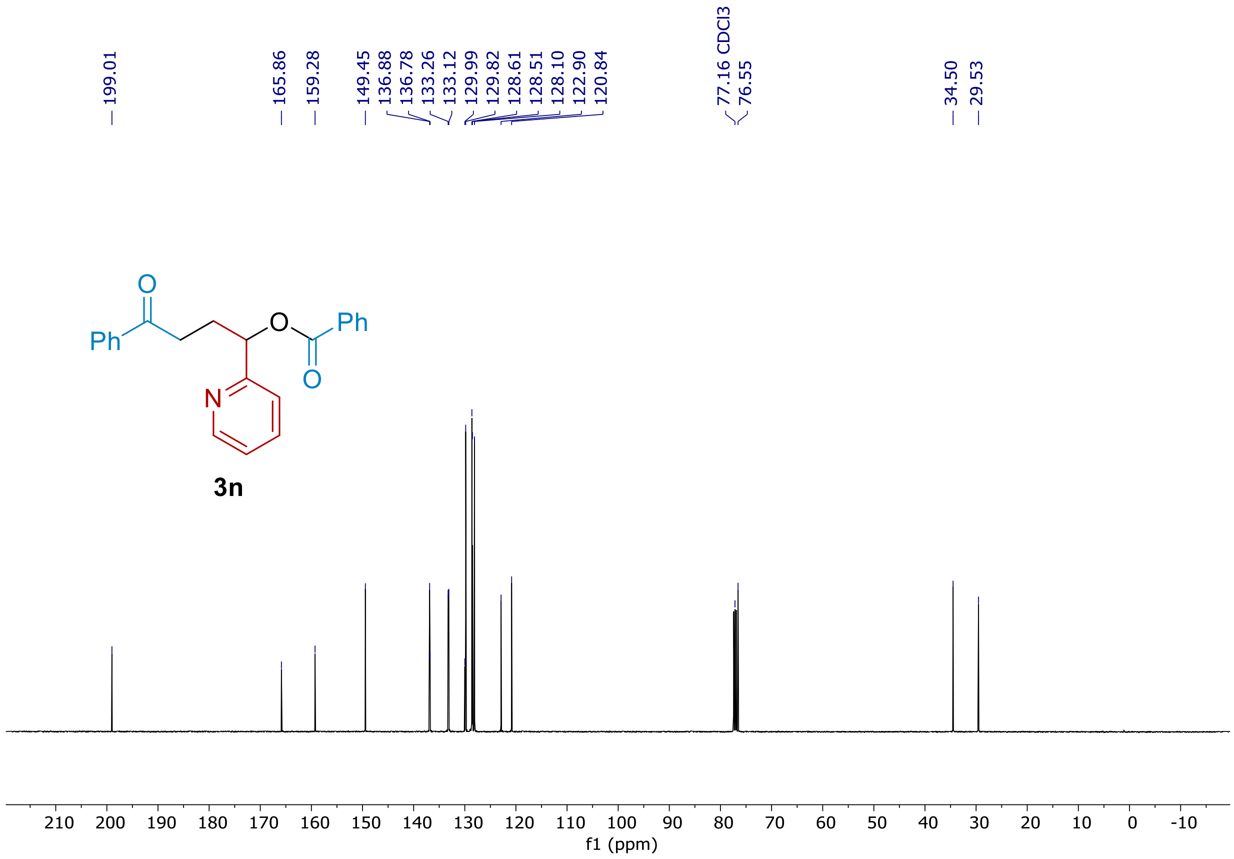

Figure S42. $^{13}C\{^{1}H\}$ NMR spectrum of **3n** in $CDCl_3$ (125 MHz)

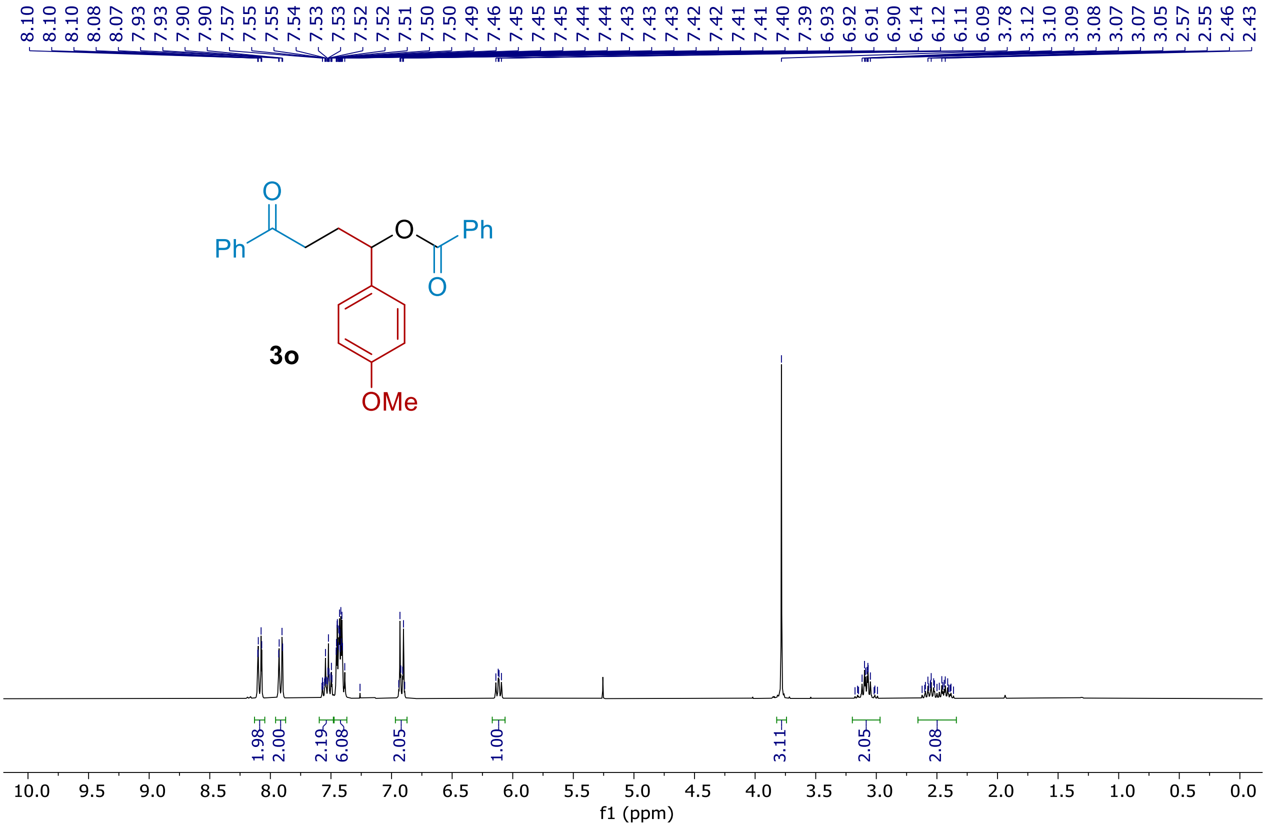

Figure S43. ^{1}H NMR spectrum of **3o** in $CDCl_3$ (300 MHz)

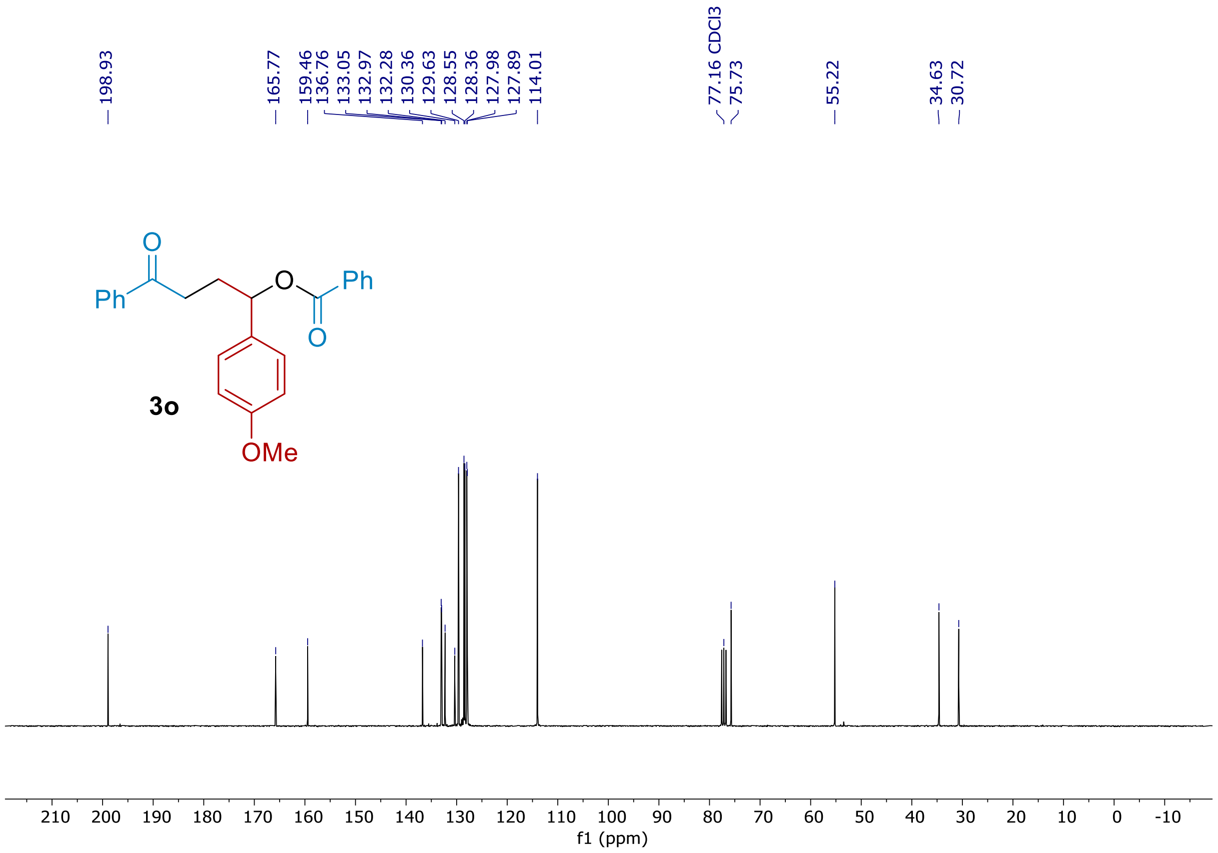

Figure S44. $^{13}C\{^{1}H\}$ NMR spectrum of **3o** in $CDCl_3$ (75 MHz)

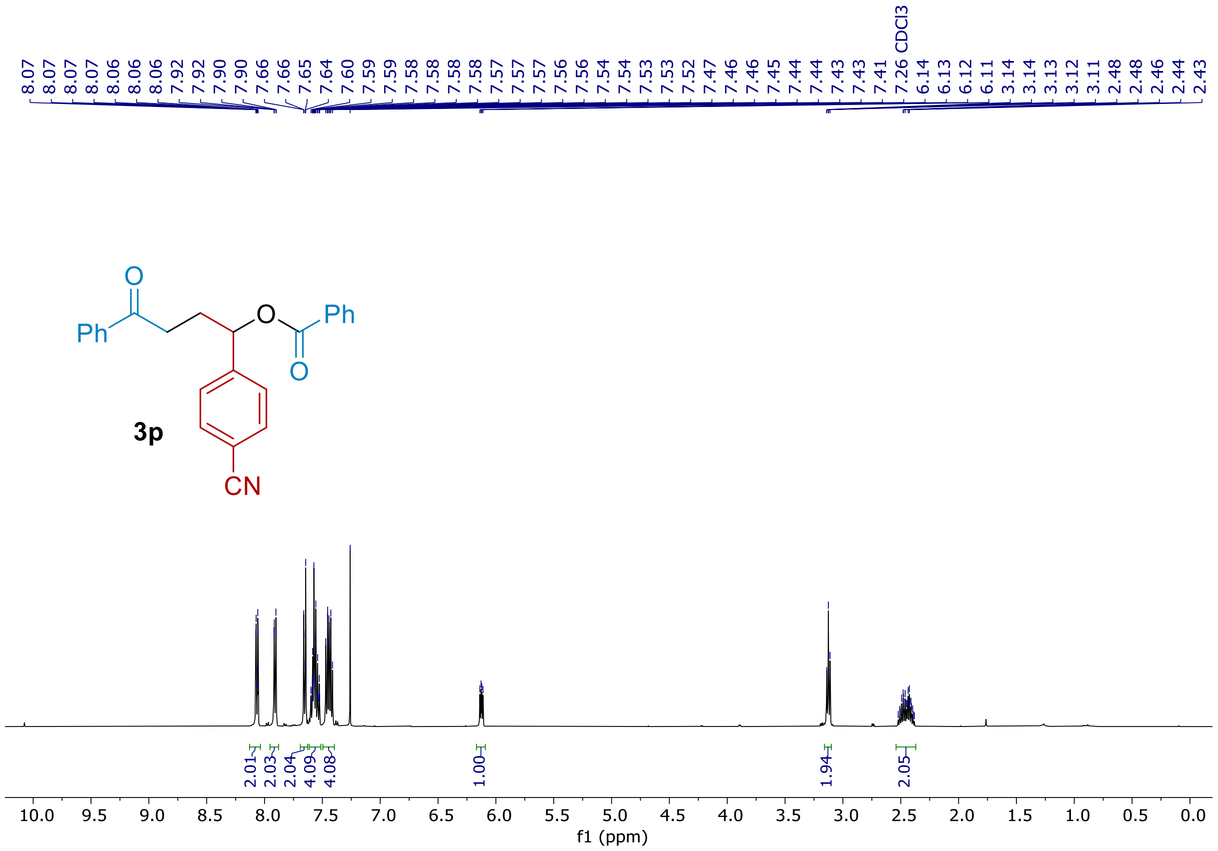

Figure S45. ^{1}H NMR spectrum of **3p** in $CDCl_3$ (500 MHz)

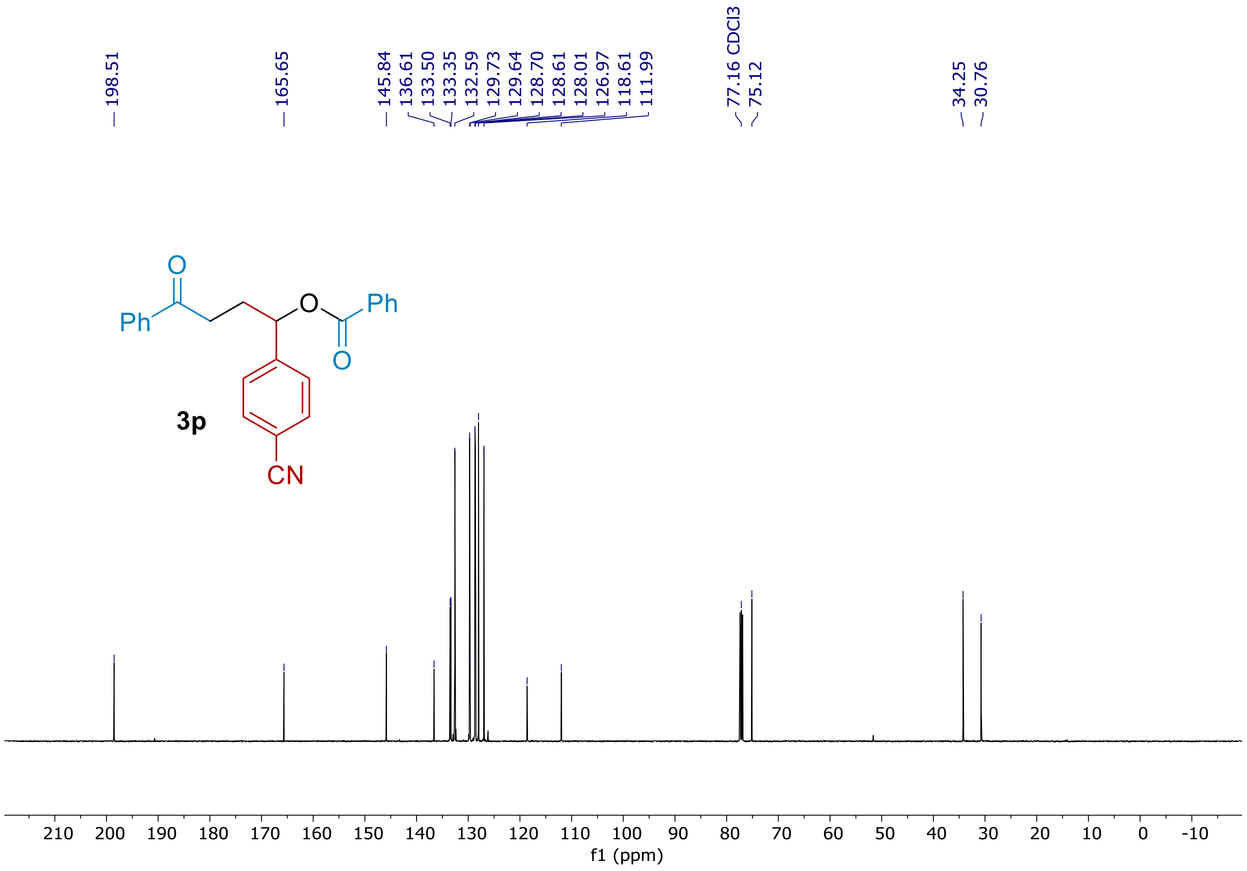

Figure S46. $^{13}C\{^{1}H\}$ NMR spectrum of **3p** in $CDCl_3$ (125 MHz)

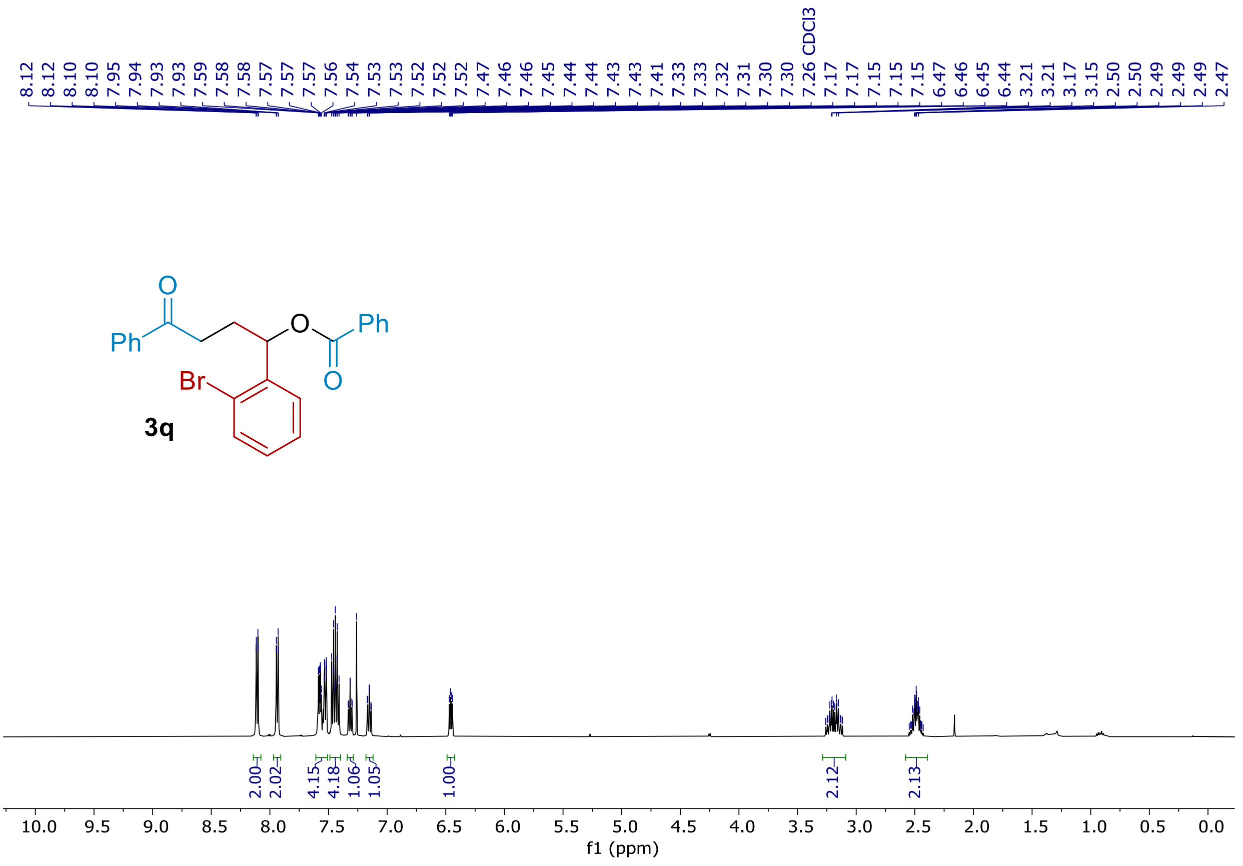

Figure S47. ^{1}H NMR spectrum of **3q** in $CDCl_3$ (500 MHz)

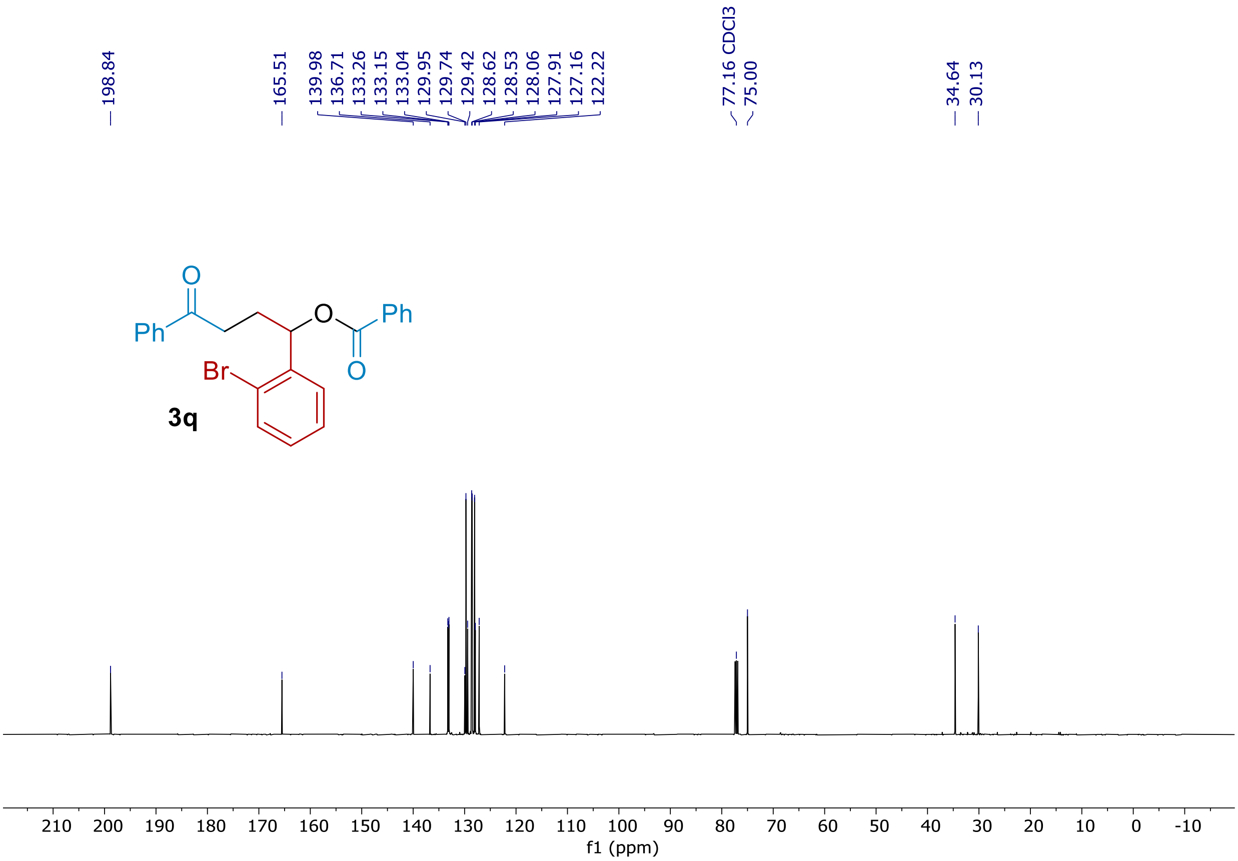

Figure S48. $^{13}C\{^1H\}$ NMR spectrum of **3q** in $CDCl_3$ (125 MHz)

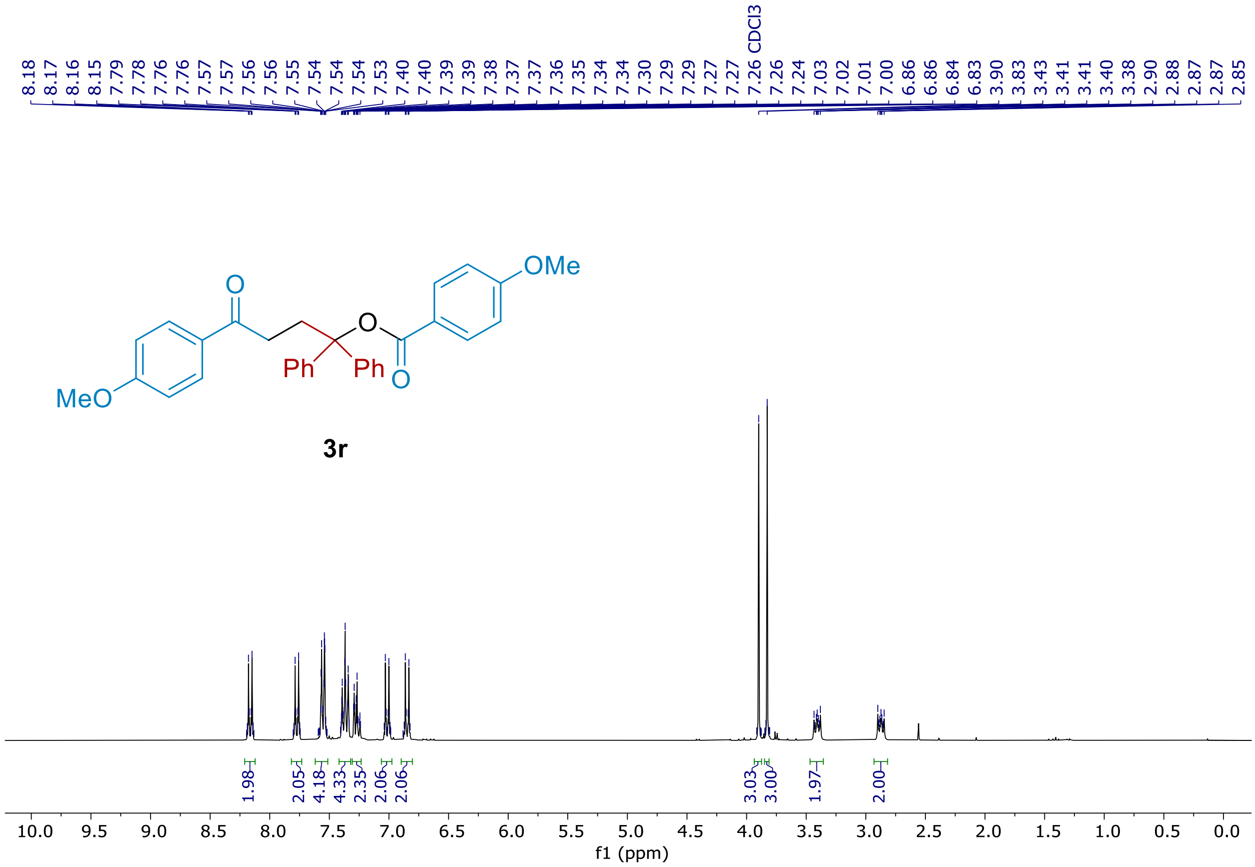

Figure S49. ^1H NMR spectrum of **3r** in $CDCl_3$ (300 MHz)

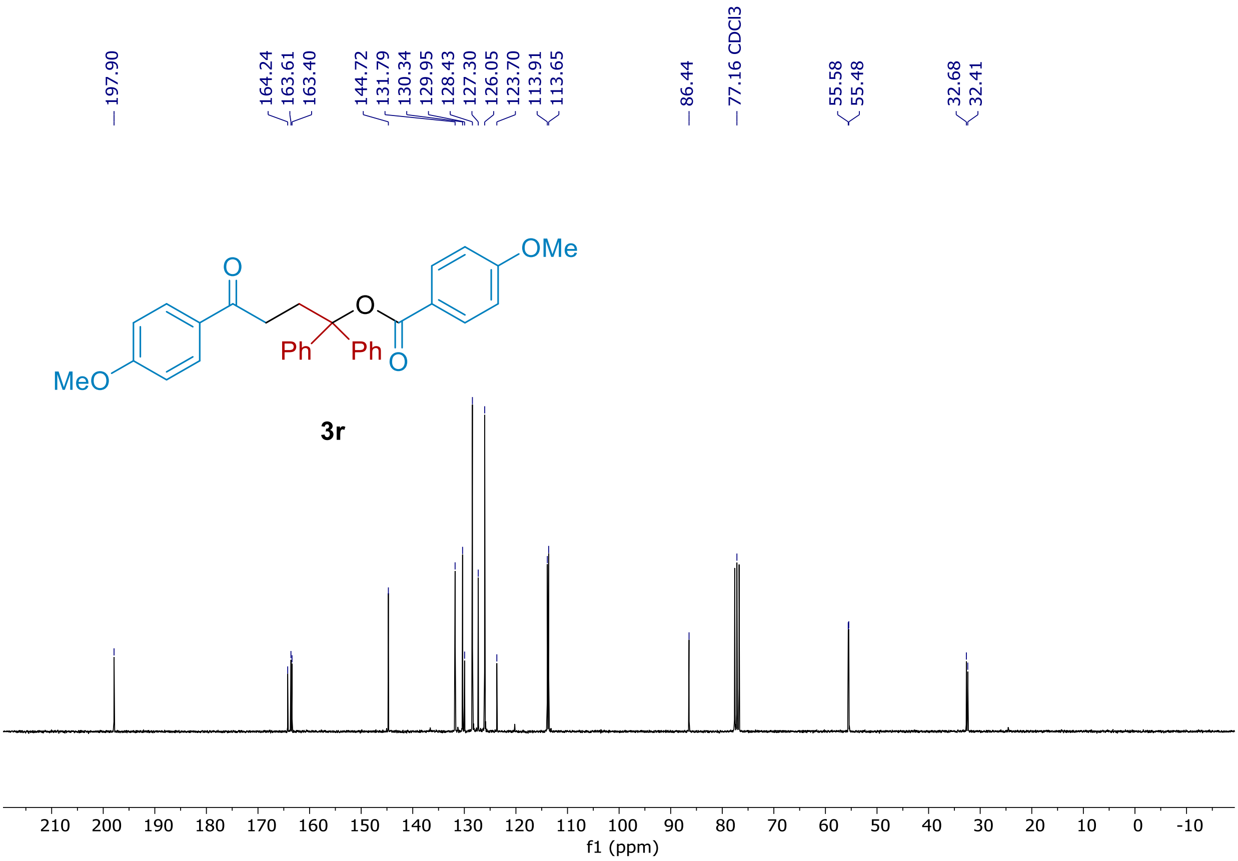

Figure S50. $^{13}C\{^{1}H\}$ NMR spectrum of **3r** in $CDCl_3$ (75 MHz)

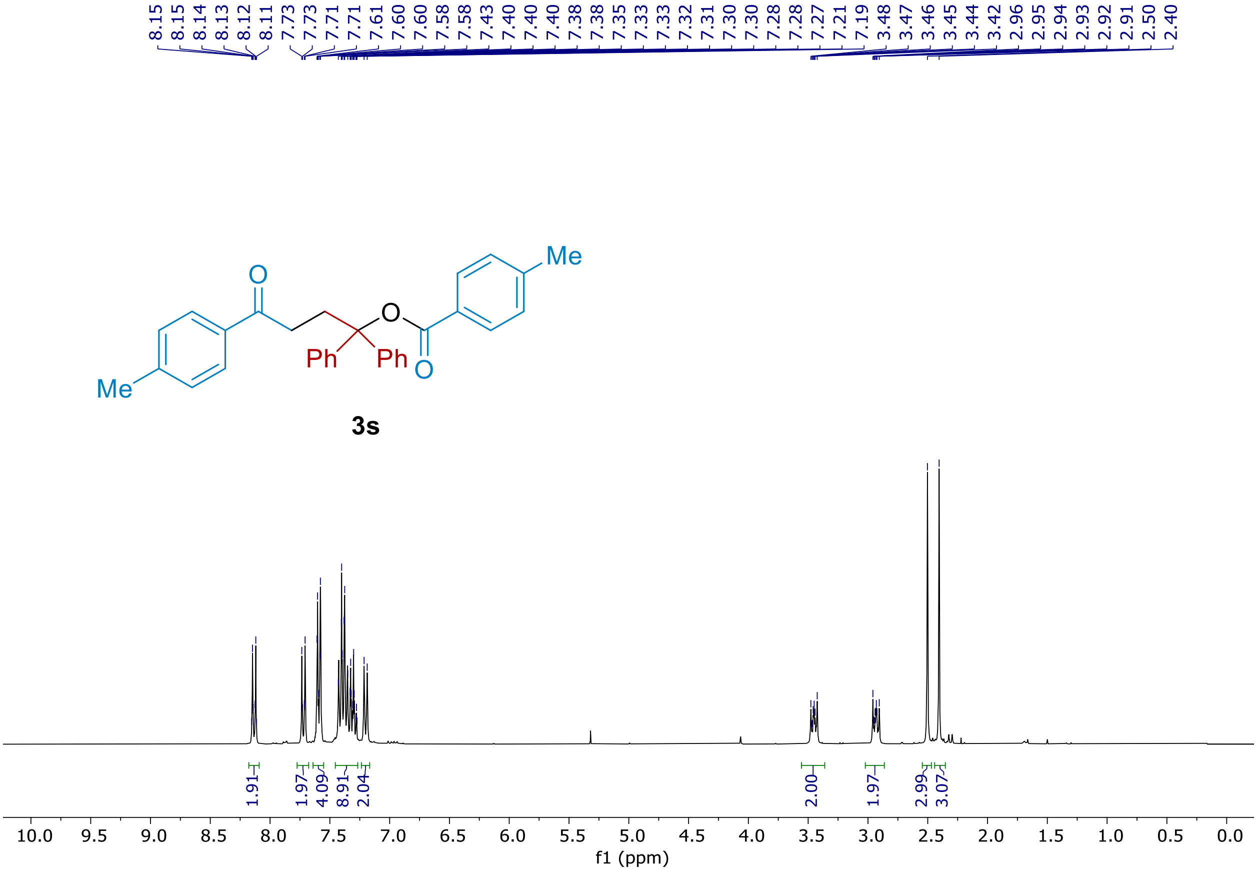

Figure S51. ^{1}H NMR spectrum of **3s** in $CDCl_3$ (300 MHz)

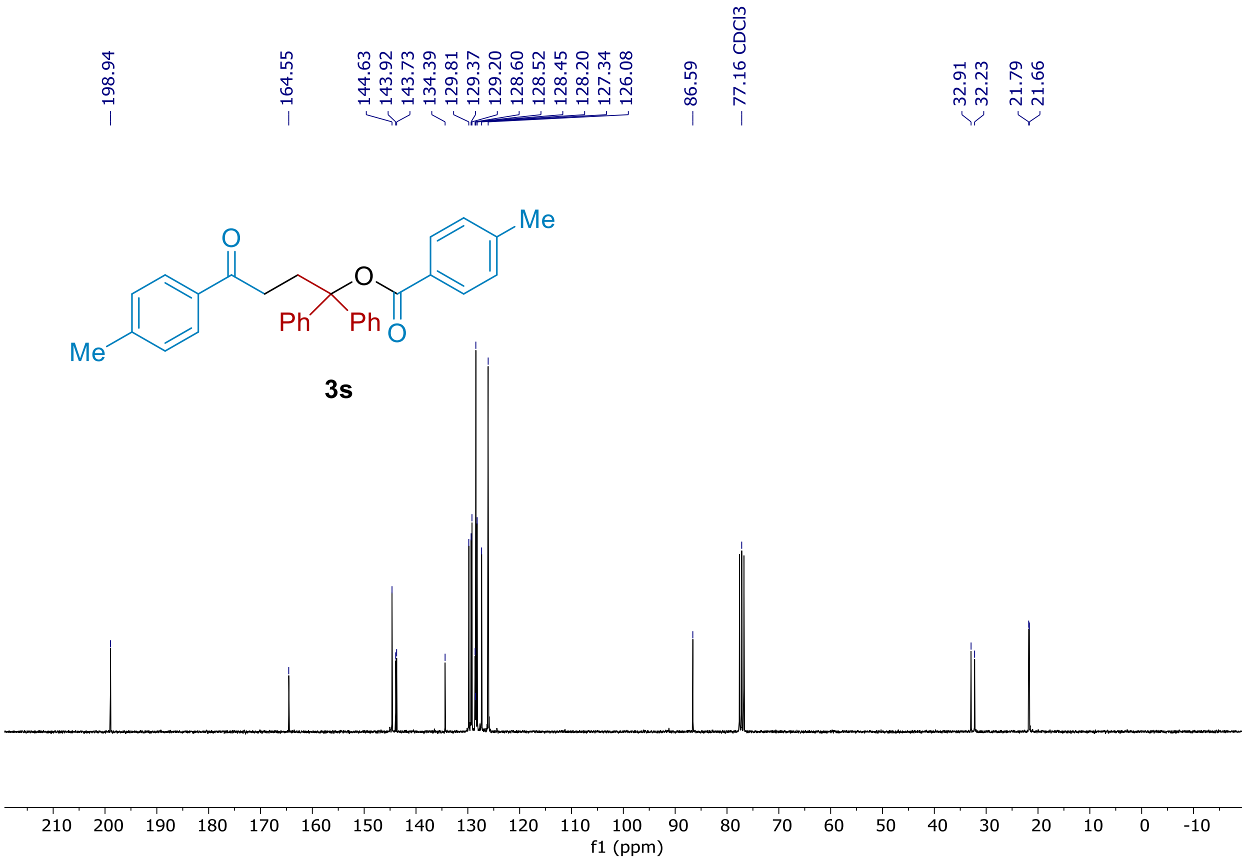

Figure S52. $^{13}C\{^{1}H\}$ NMR spectrum of **3s** in $CDCl_3$ (75 MHz)

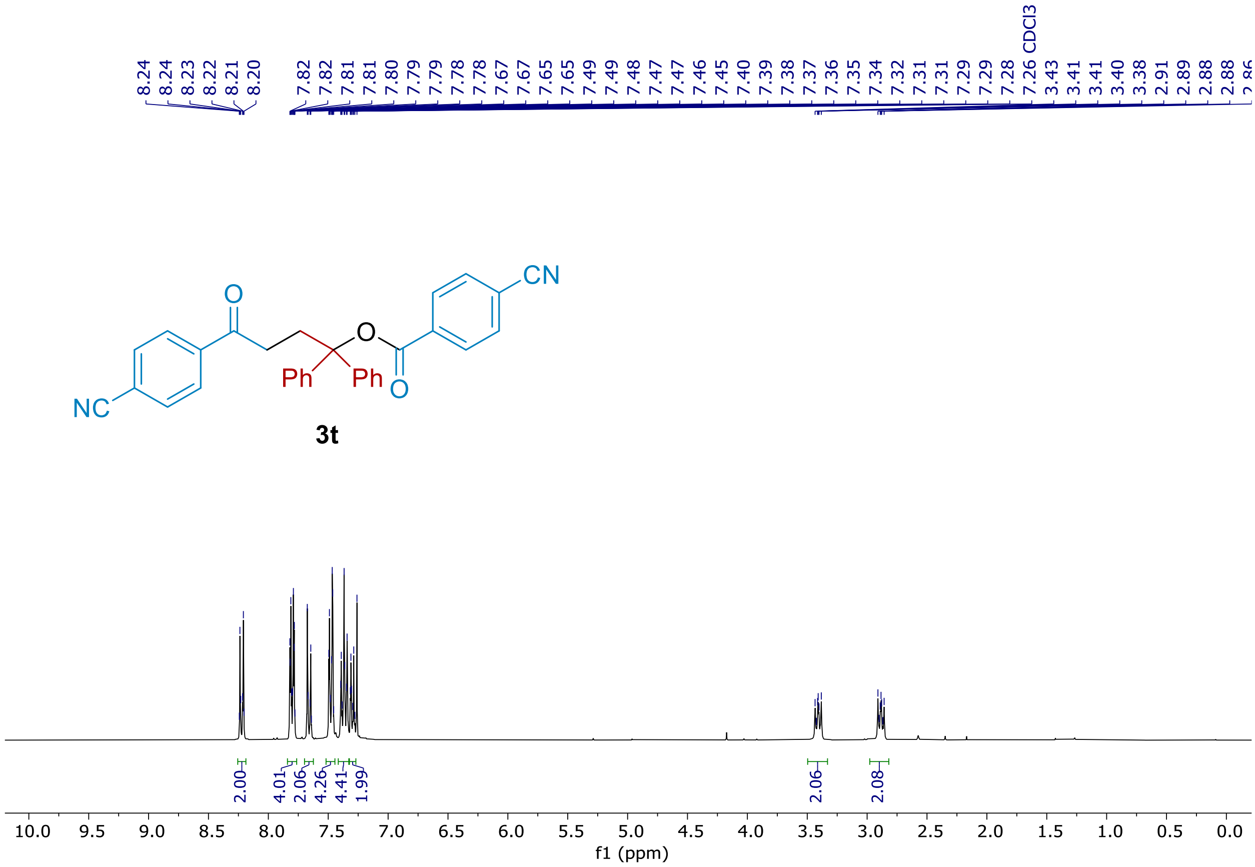

Figure S53. ^{1}H NMR spectrum of **3t** in $CDCl_3$ (300 MHz)

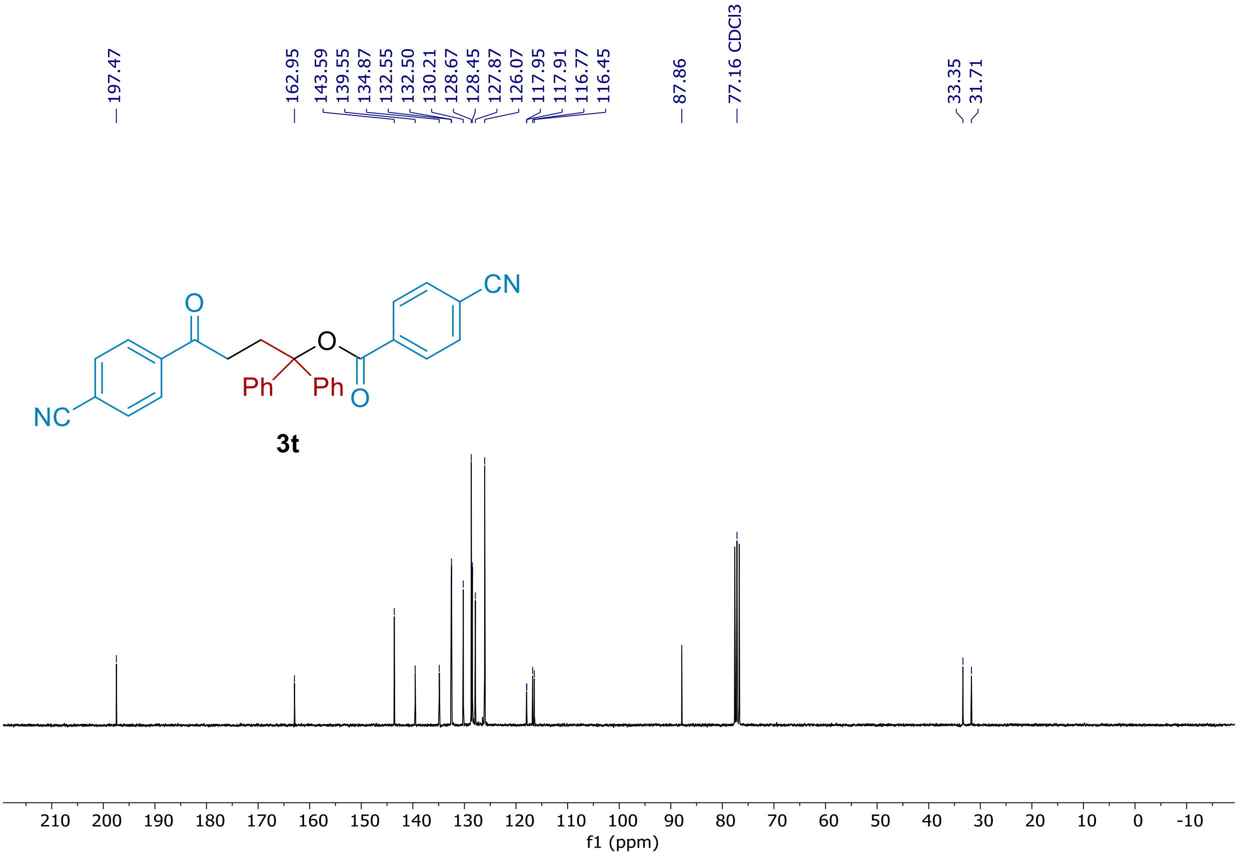

Figure S54. $^{13}C\{^{1}H\}$ NMR spectrum of **3t** in $CDCl_3$ (75 MHz)

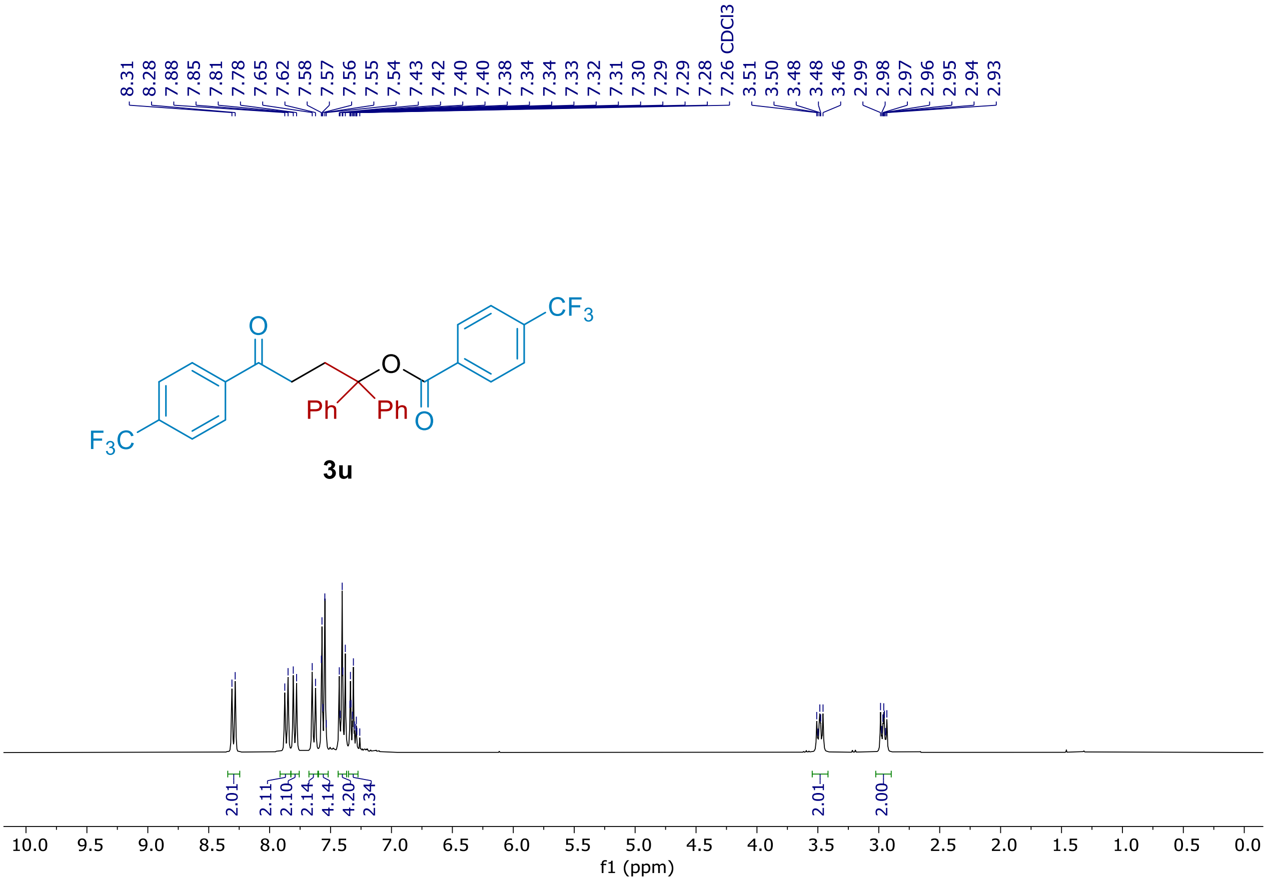

Figure S55. ^{1}H NMR spectrum of **3u** in $CDCl_3$ (300 MHz)

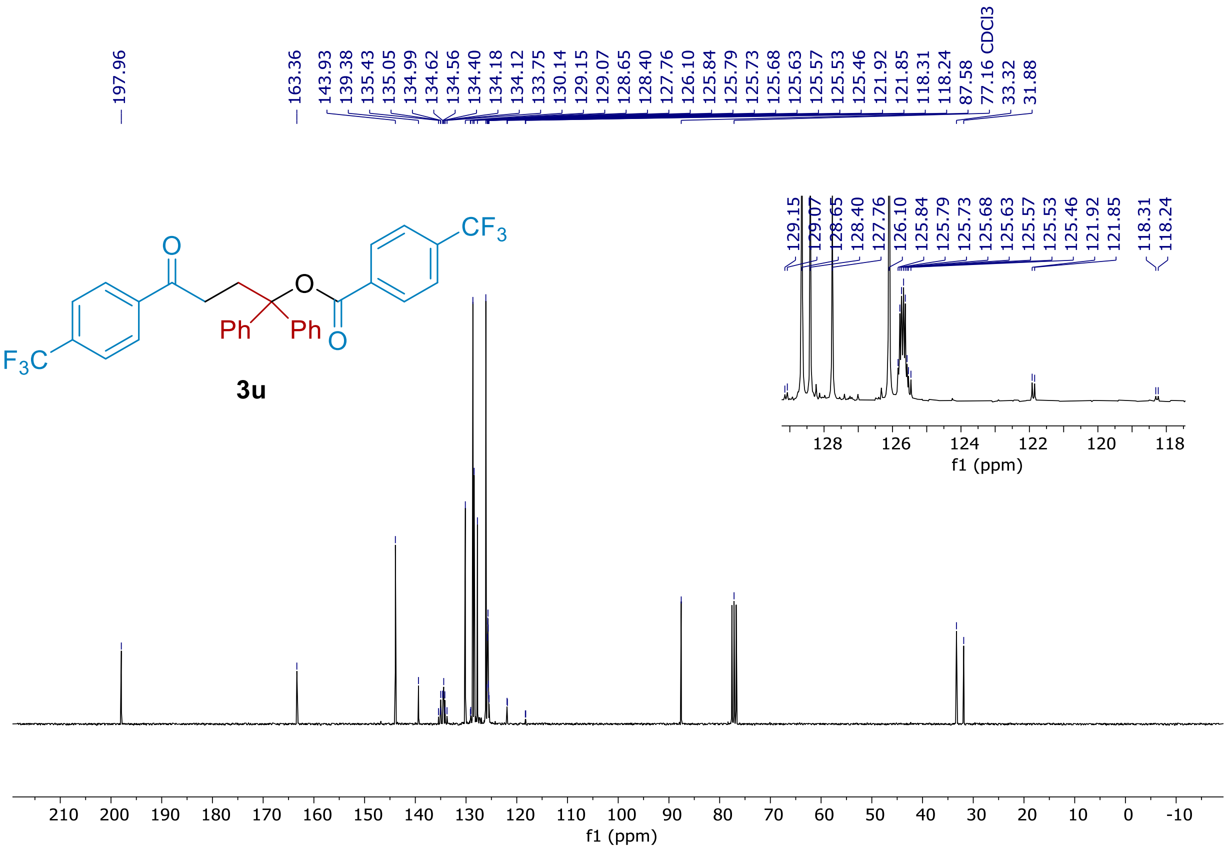

Figure S56. $^{13}C\{^{1}H\}$ NMR spectrum of **3u** in $CDCl_3$ (75 MHz)

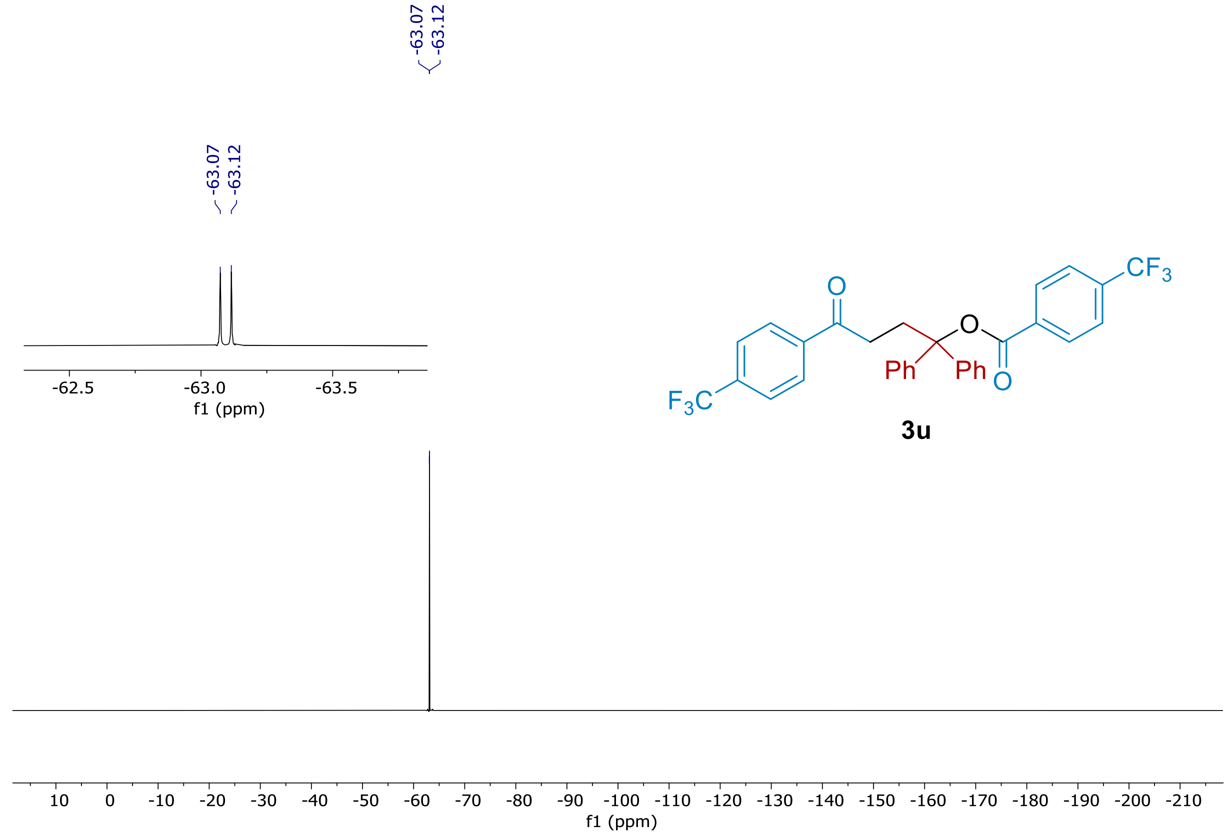

Figure S57. $^{19}F\{^{1}H\}$ NMR spectrum of **3u** in $CDCl_3$ (282 MHz)

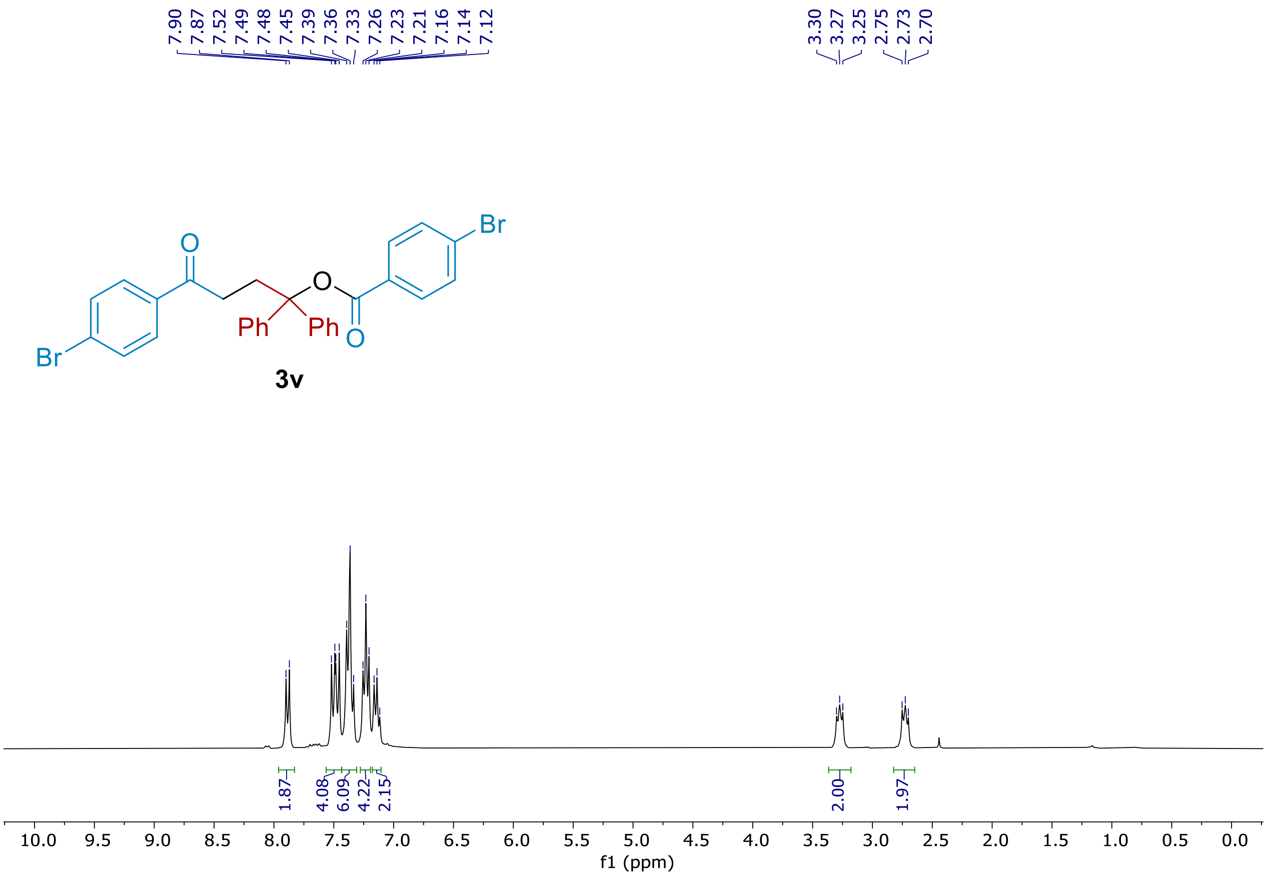

Figure S58. ^{1}H NMR spectrum of **3v** in $CDCl_3$ (300 MHz)

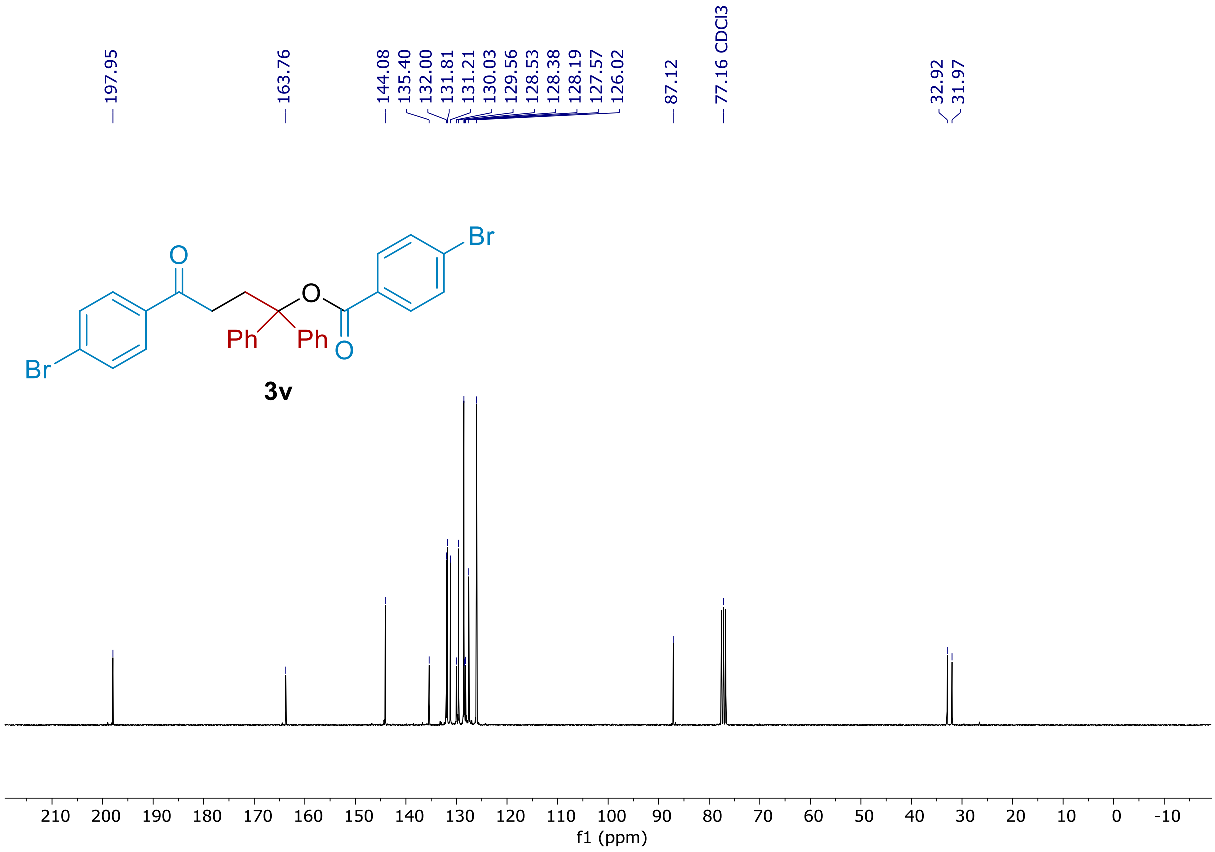

Figure S59. $^{13}C\{^{1}H\}$ NMR spectrum of **3v** in $CDCl_3$ (75 MHz)

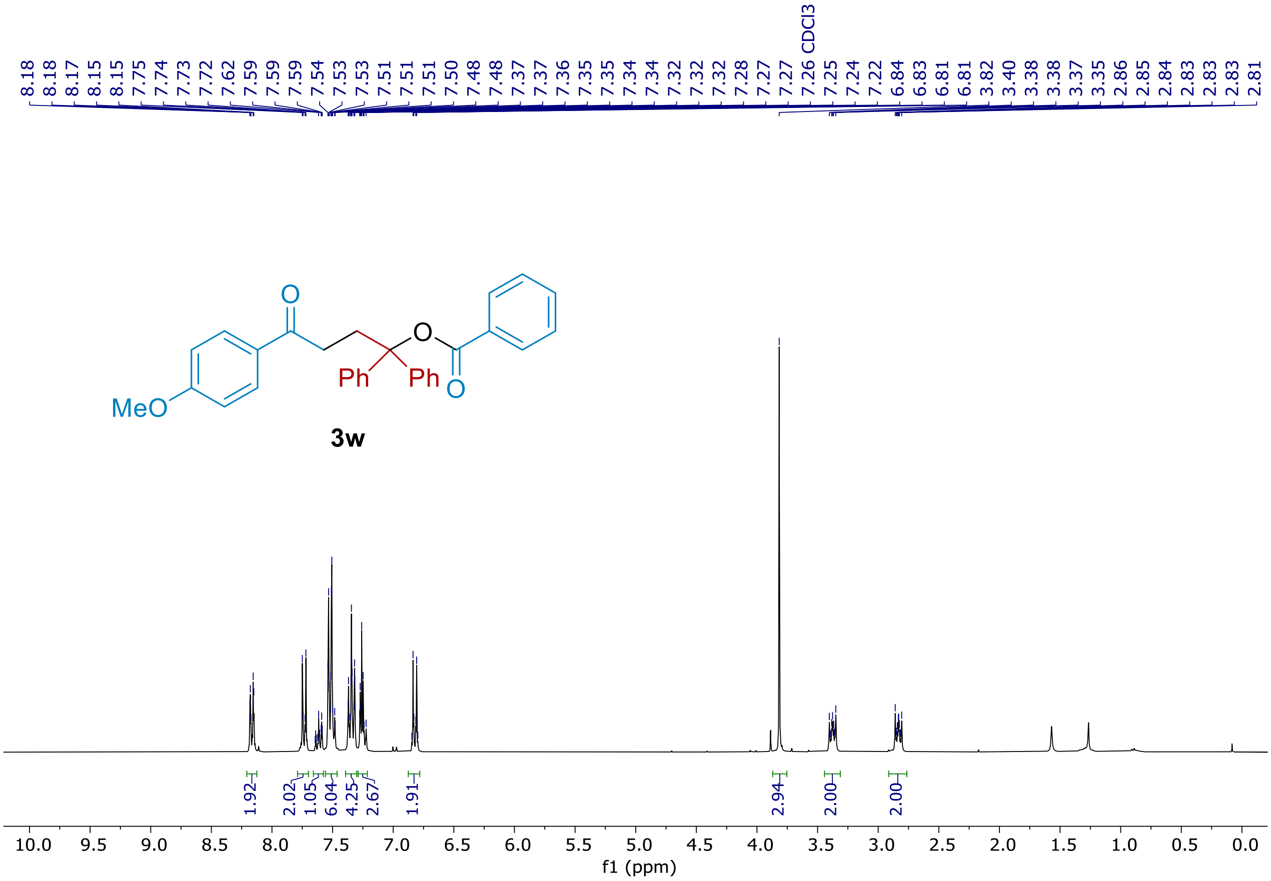

Figure S60. ^{1}H NMR spectrum of **3w** in $CDCl_3$ (300 MHz)

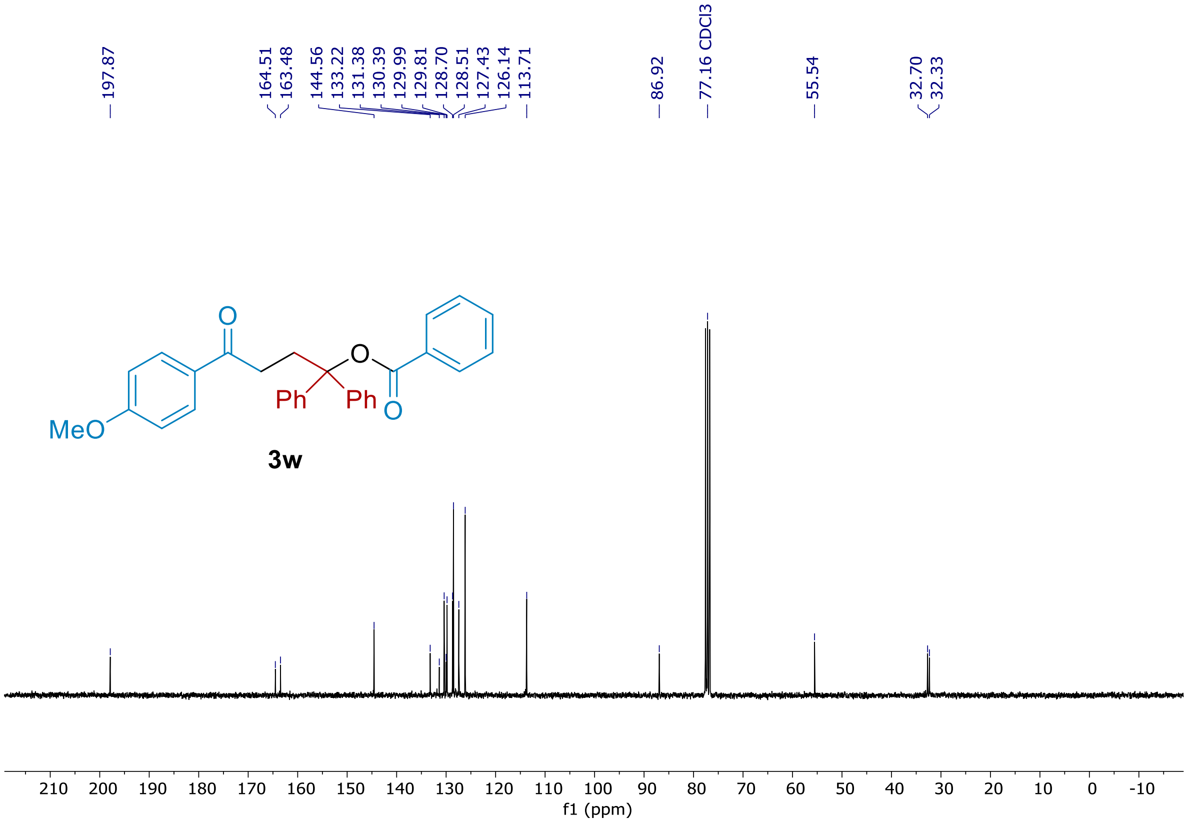

Figure S61. $^{13}C\{^{1}H\}$ NMR spectrum of **3w** in $CDCl_3$ (75 MHz)

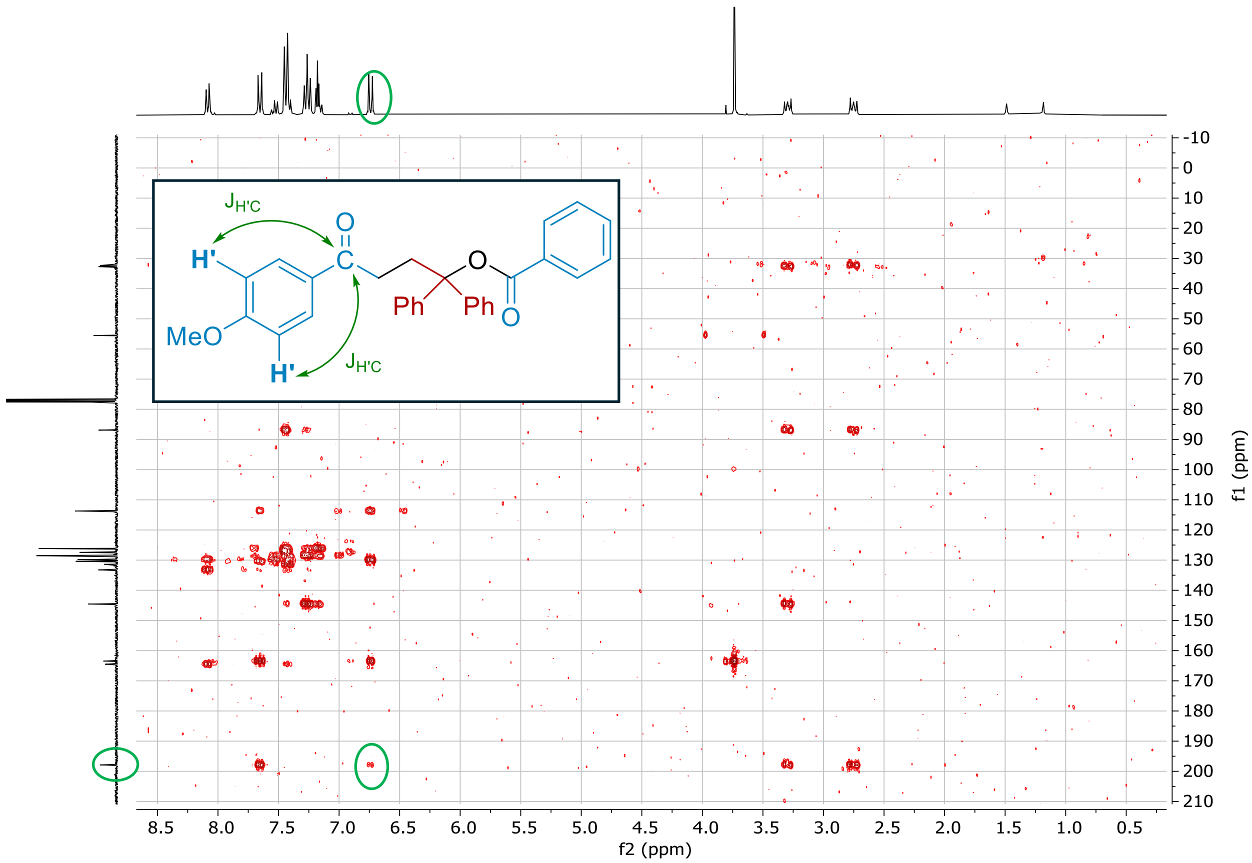

Figure S62. 2D ^{1}H-^{13}C HMBC spectrum of **3w** in $CDCl_3$. The **2D ^{1}H-^{13}C HMBC** spectrum of **3w** shows coupling between the protons in the ortho position relative to the methoxy group and the ketone carbonyl, aiding in the determination of the correct regioisomer.

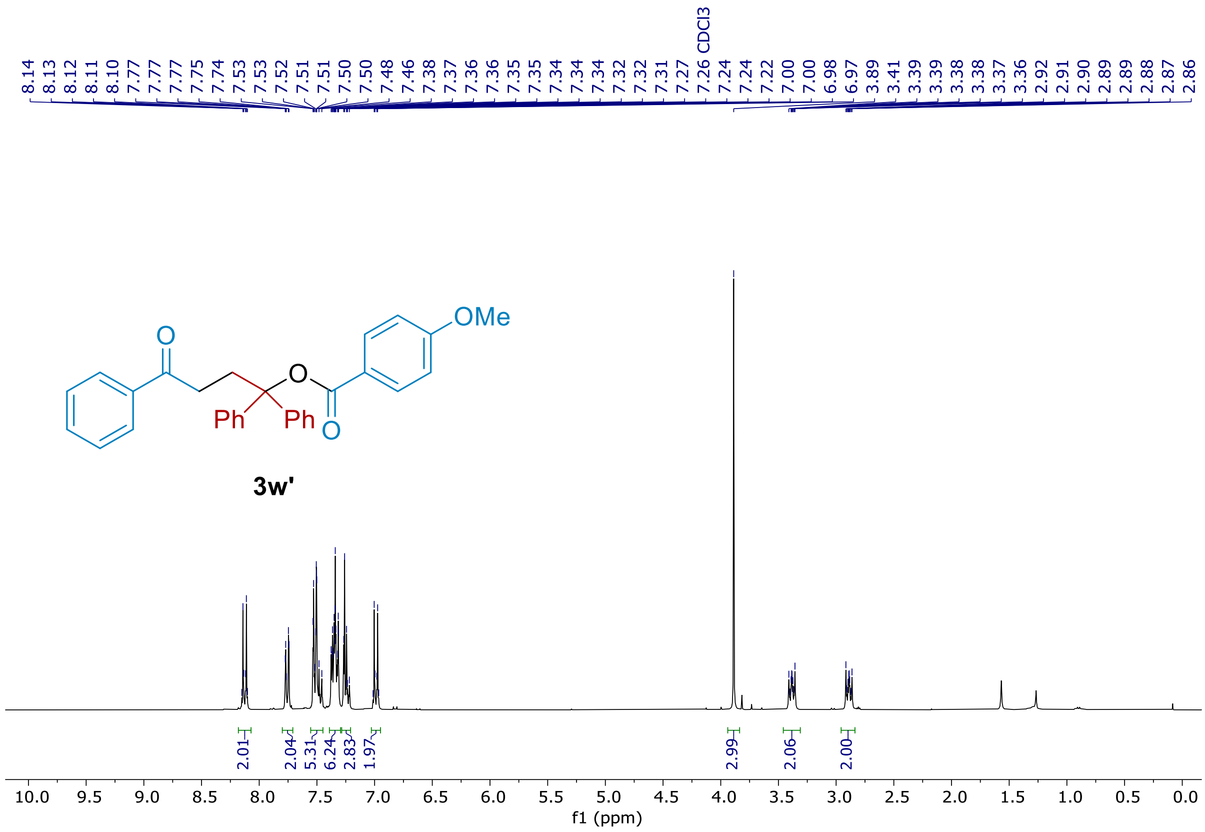

Figure S63. ^{1}H NMR spectrum of **3w'** in $CDCl_3$ (300 MHz)

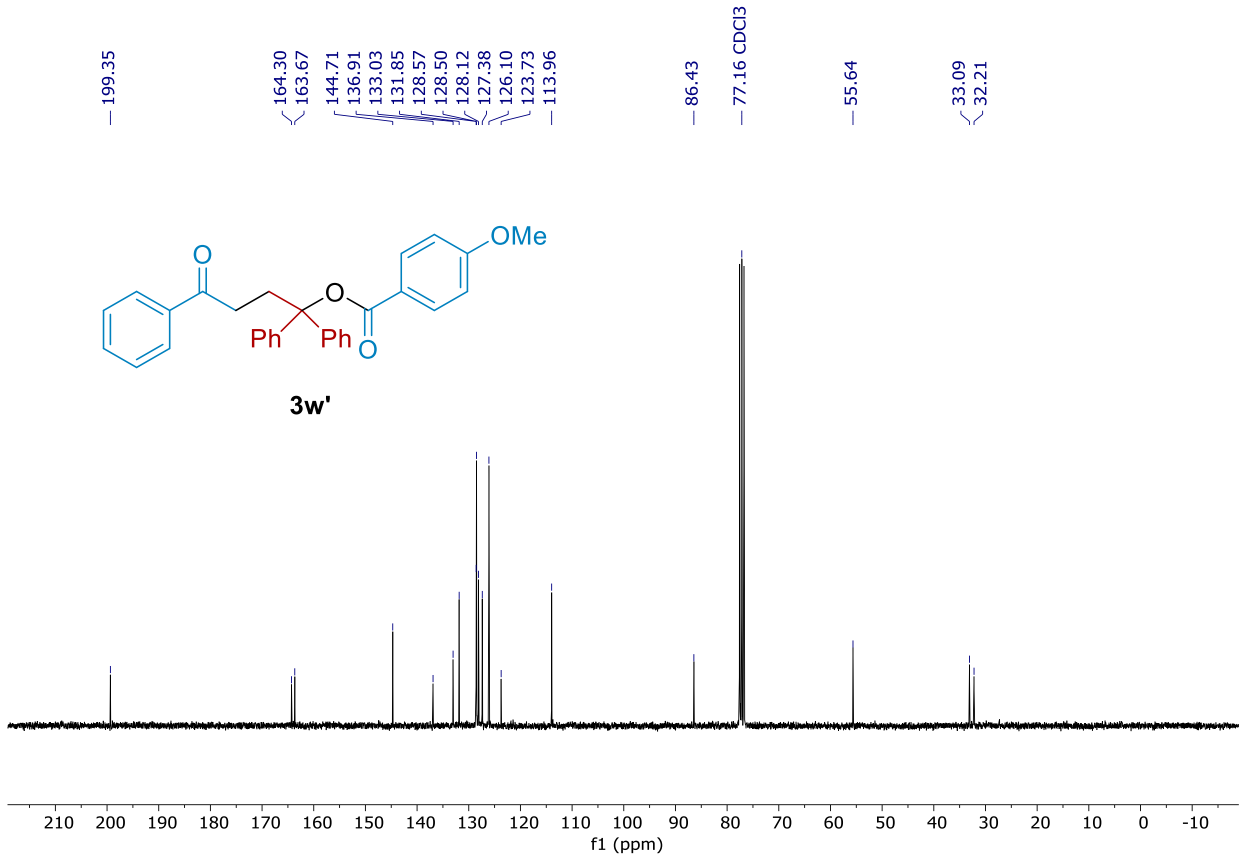

Figure S64. $^{13}C\{^{1}H\}$ NMR spectrum of **3w'** in $CDCl_3$ (75 MHz)

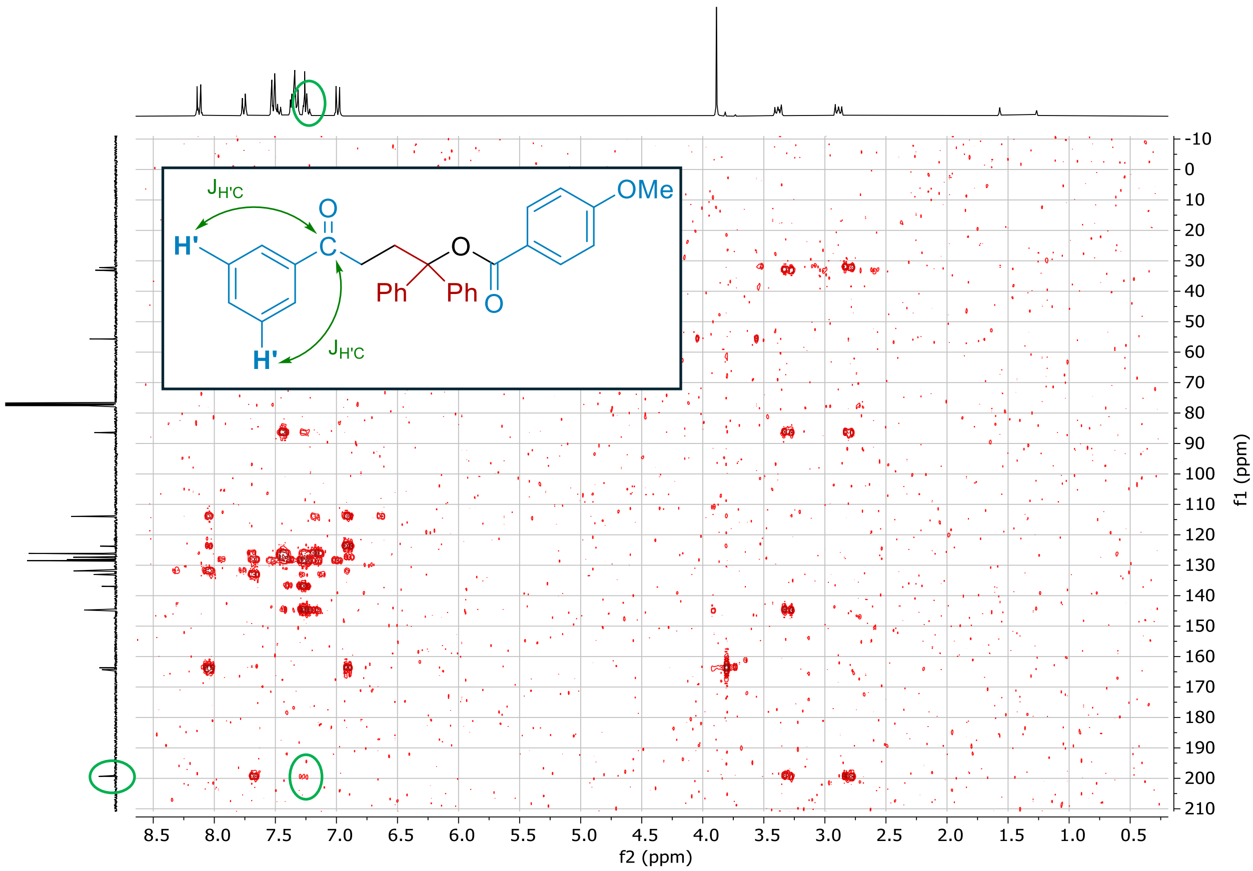

Figure S65. 2D ^{1}H-^{13}C HMBC spectrum of **3w'** in $CDCl_3$

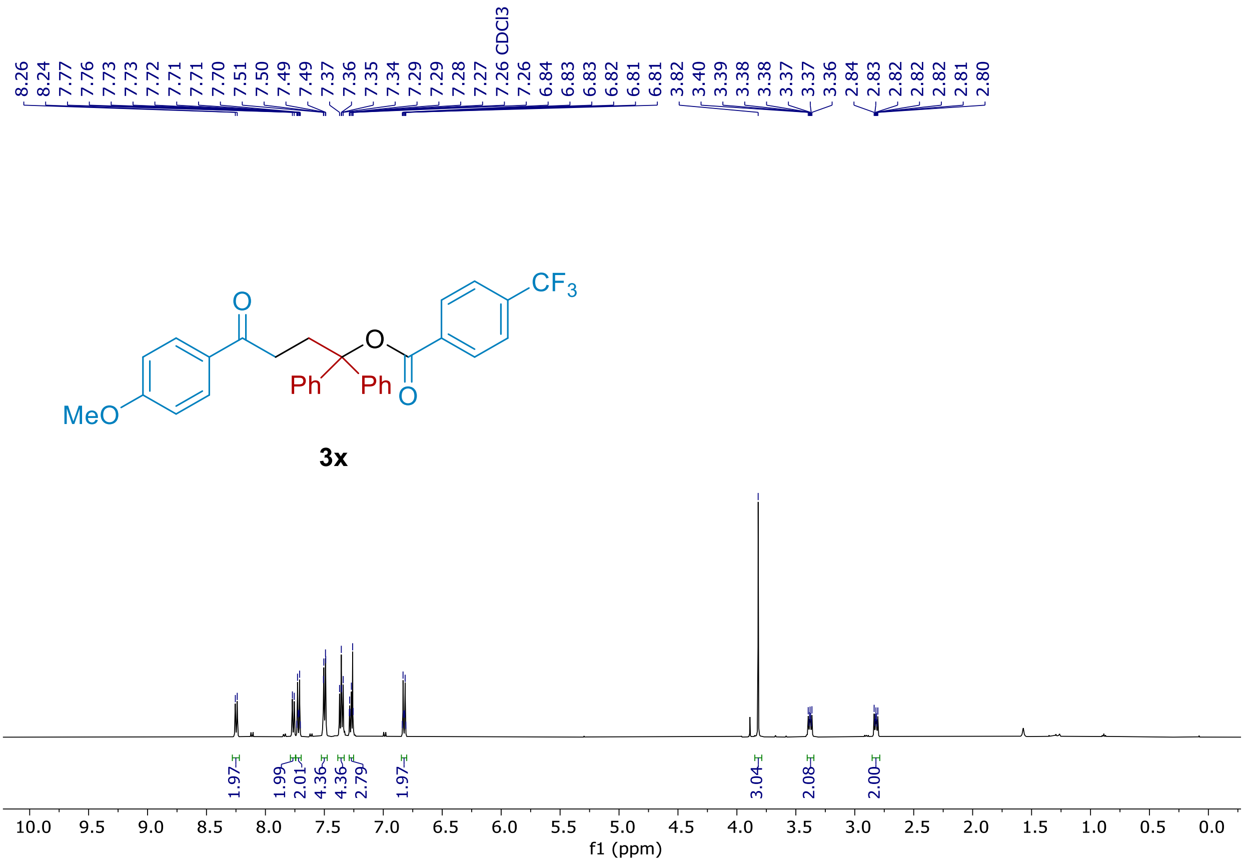

Figure S66. ^{1}H NMR spectrum of **3x** in $CDCl_3$ (500 MHz)

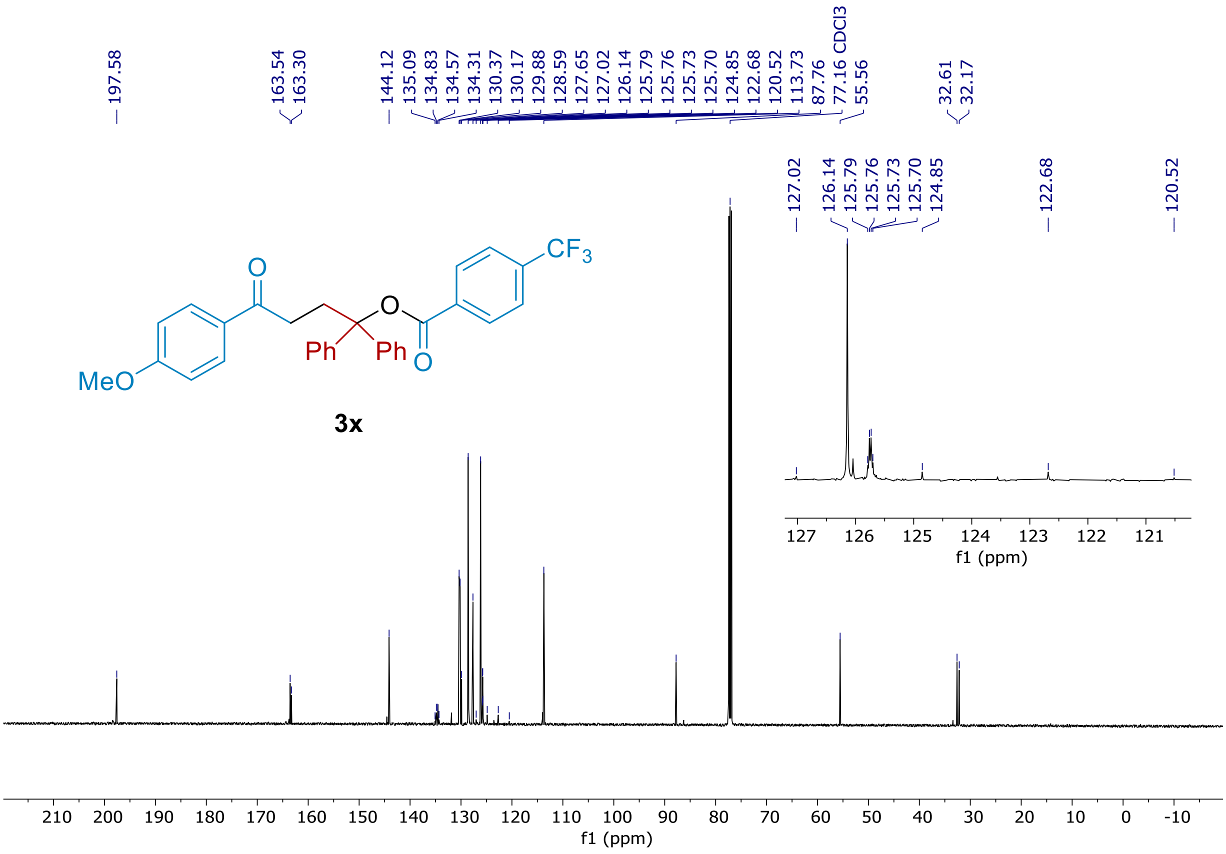

Figure S67. $^{13}C\{^{1}H\}$ NMR spectrum of **3x** in $CDCl_3$ (125 MHz)

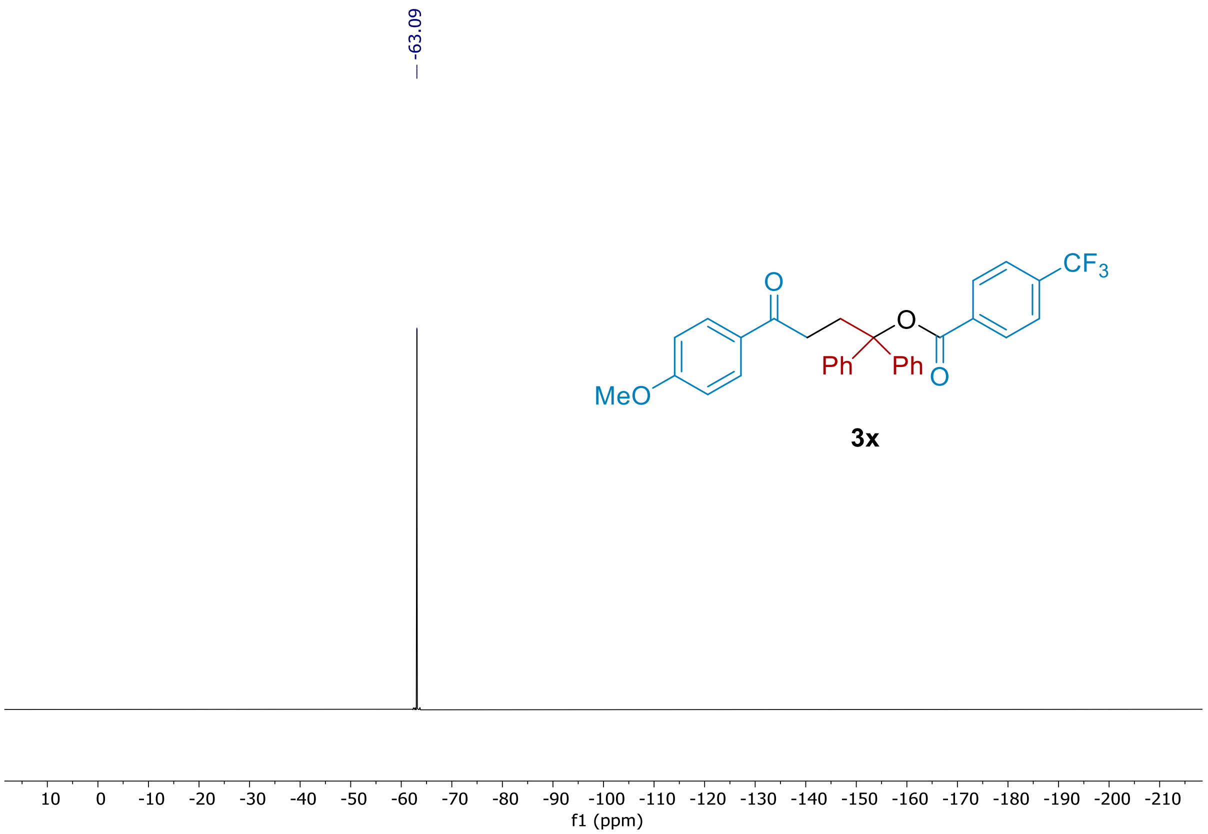

Figure S68. $^{19}F\{^{1}H\}$ NMR spectrum of **3x** in $CDCl_3$ (282 MHz)

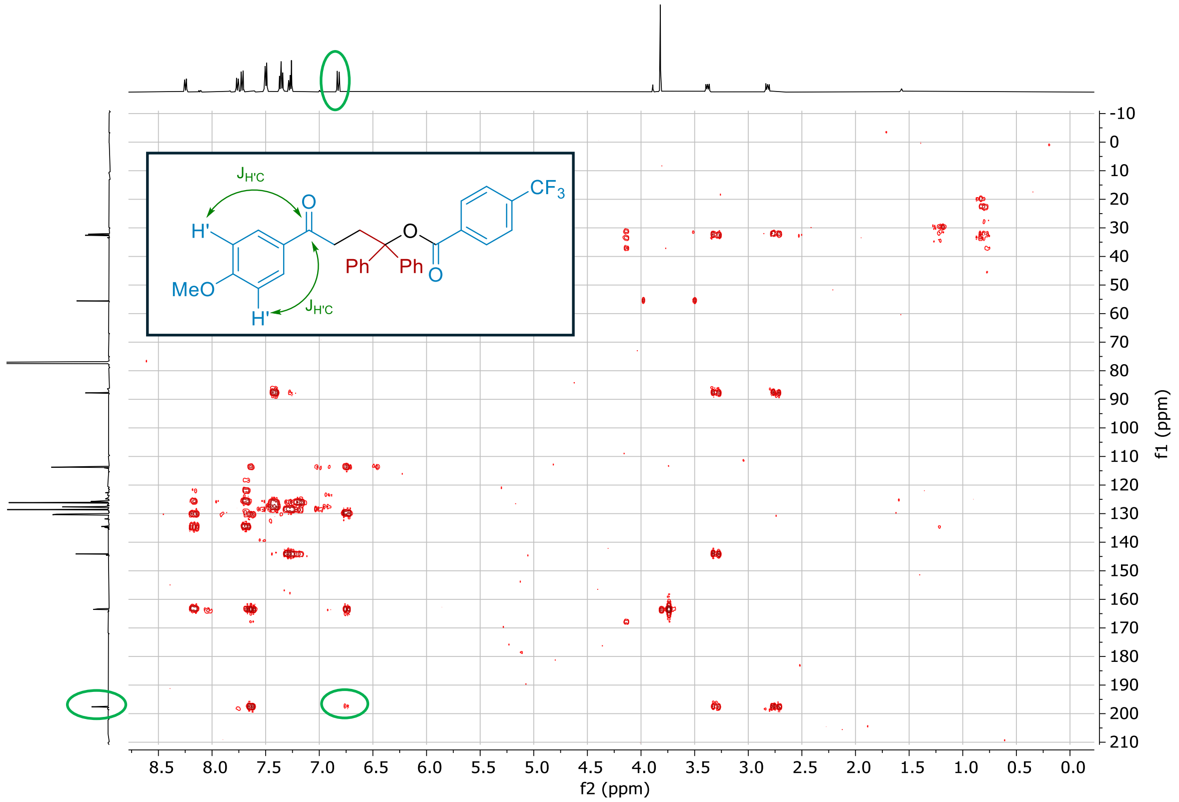

Figure S69. 2D ^{1}H-^{13}C HMBC spectrum of **3x** in $CDCl_3$. The **2D ^{1}H-^{13}C HMBC** spectrum of **3x** shows coupling between the protons in the ortho position relative to the methoxy group and the ketone carbonyl, aiding in the determination of the correct regioisomer.

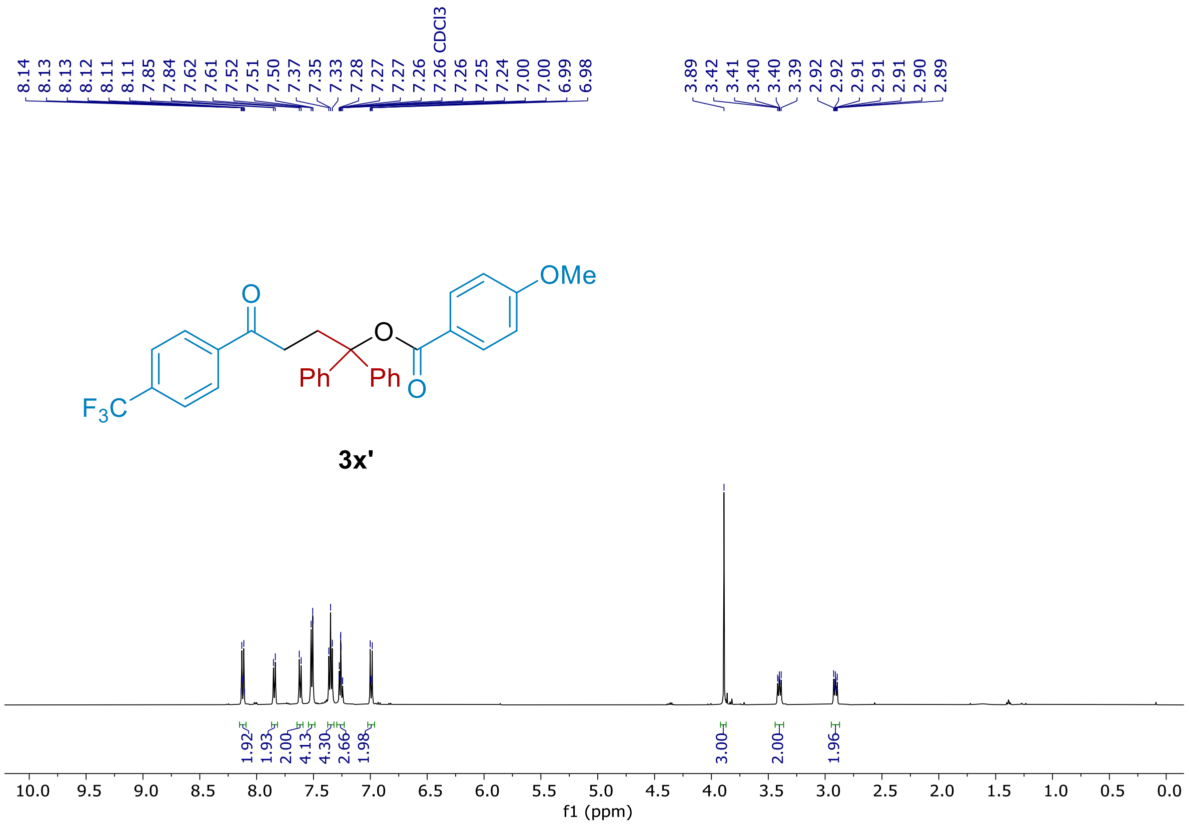

Figure S70. ^{1}H NMR spectrum of **3x’** in $CDCl_3$ (500 MHz)

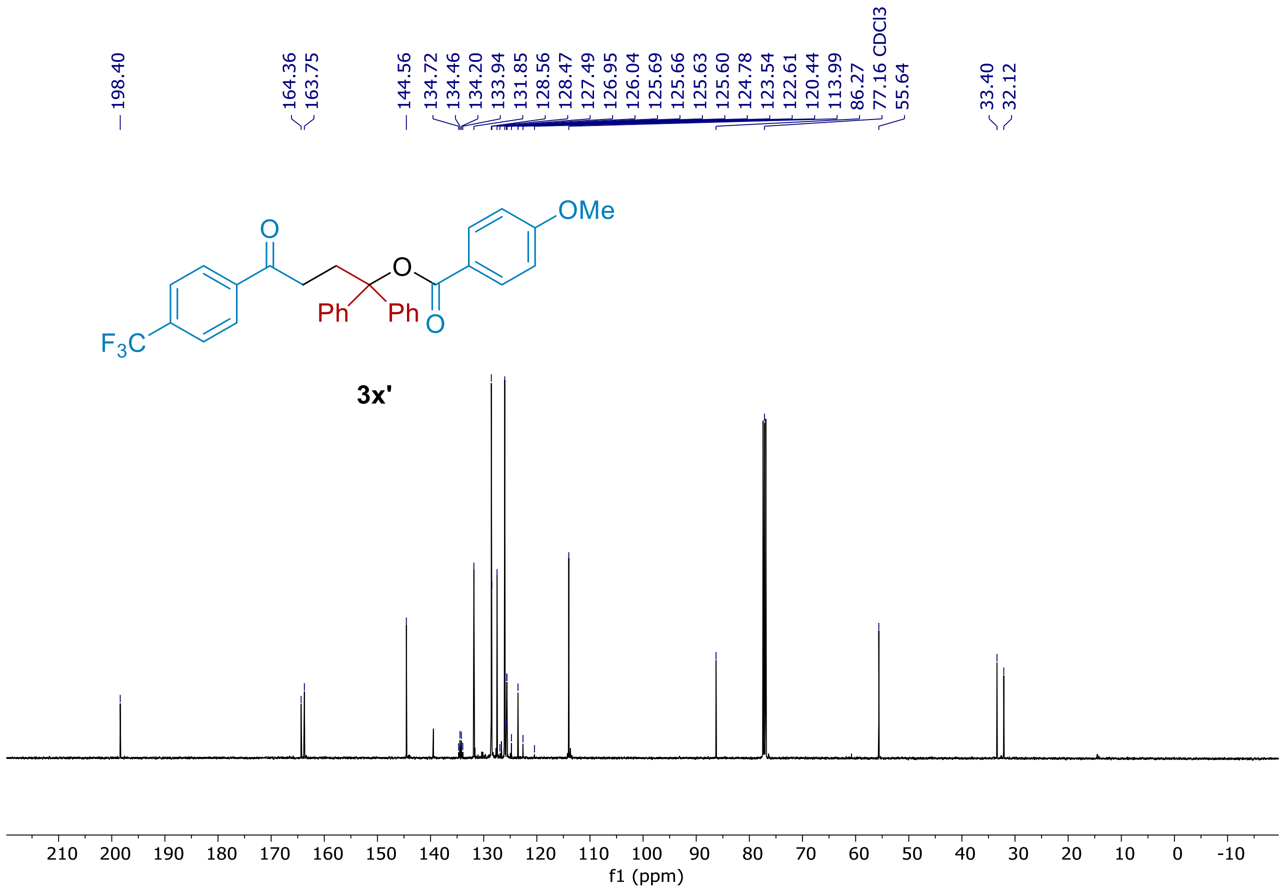

Figure S71. $^{13}C\{^{1}H\}$ NMR spectrum of **3x’** in $CDCl_3$ (125 MHz)

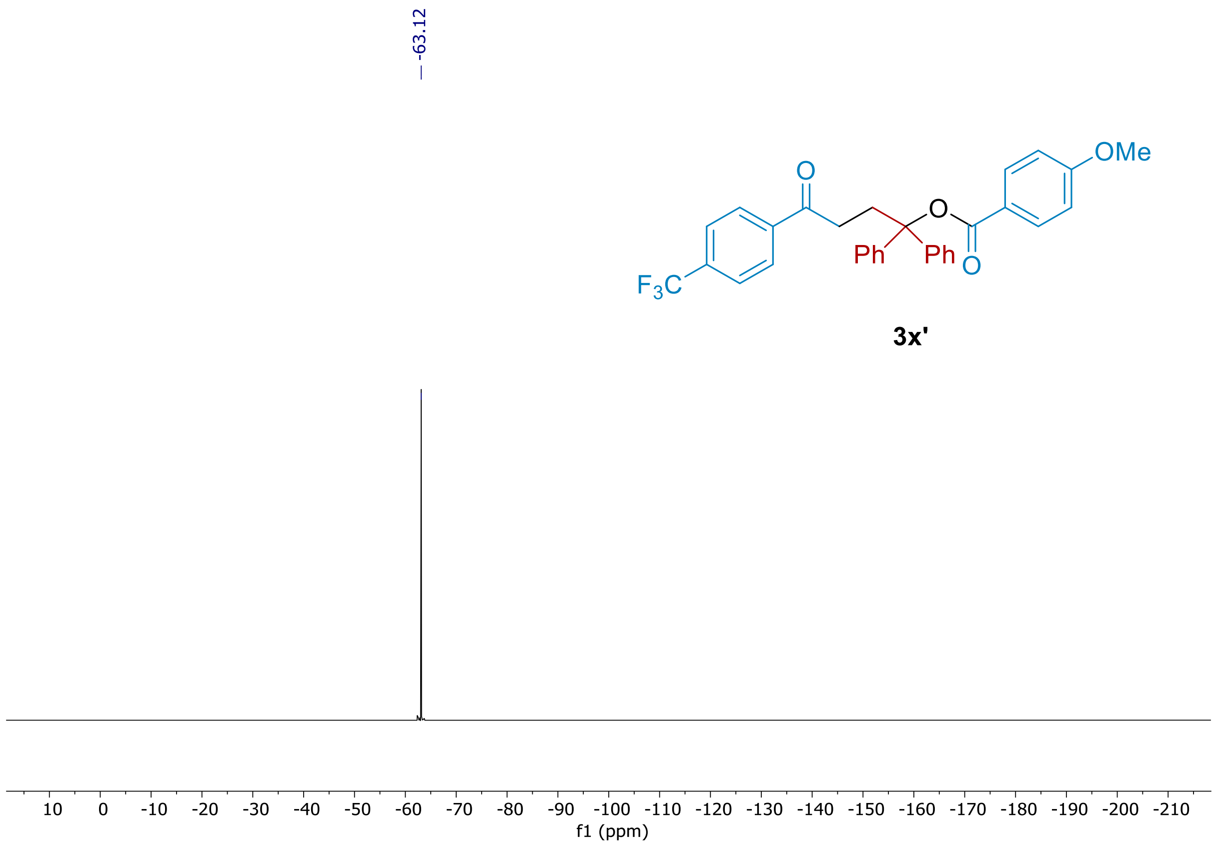

Figure S72. $^{19}F\{^{1}H\}$ NMR spectrum of **3x'** in $CDCl_3$ (282 MHz)

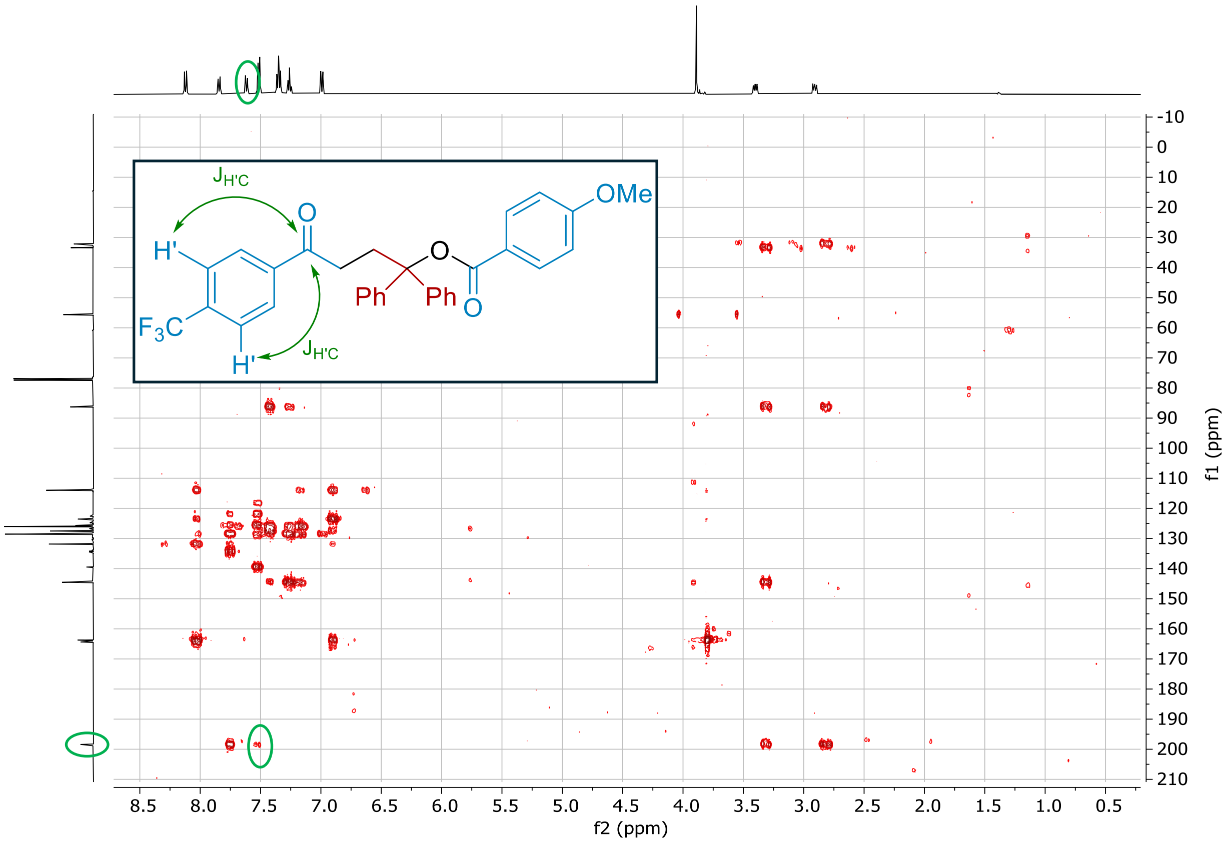

Figure S73. 2D ^{1}H-^{13}C HMBC spectrum of **3x'** in $CDCl_3$

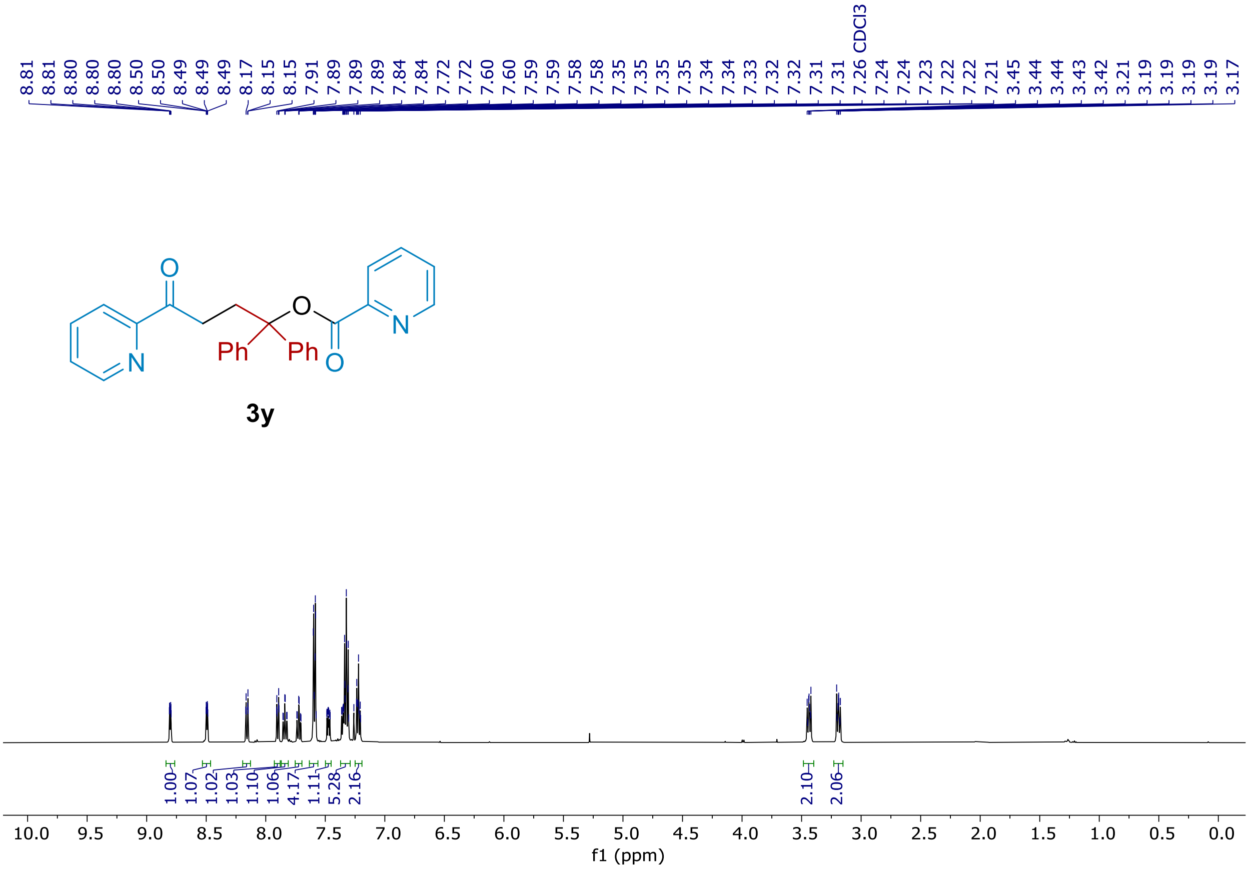

Figure S74. ^{1}H NMR spectrum of **3y** in $CDCl_3$ (500 MHz)

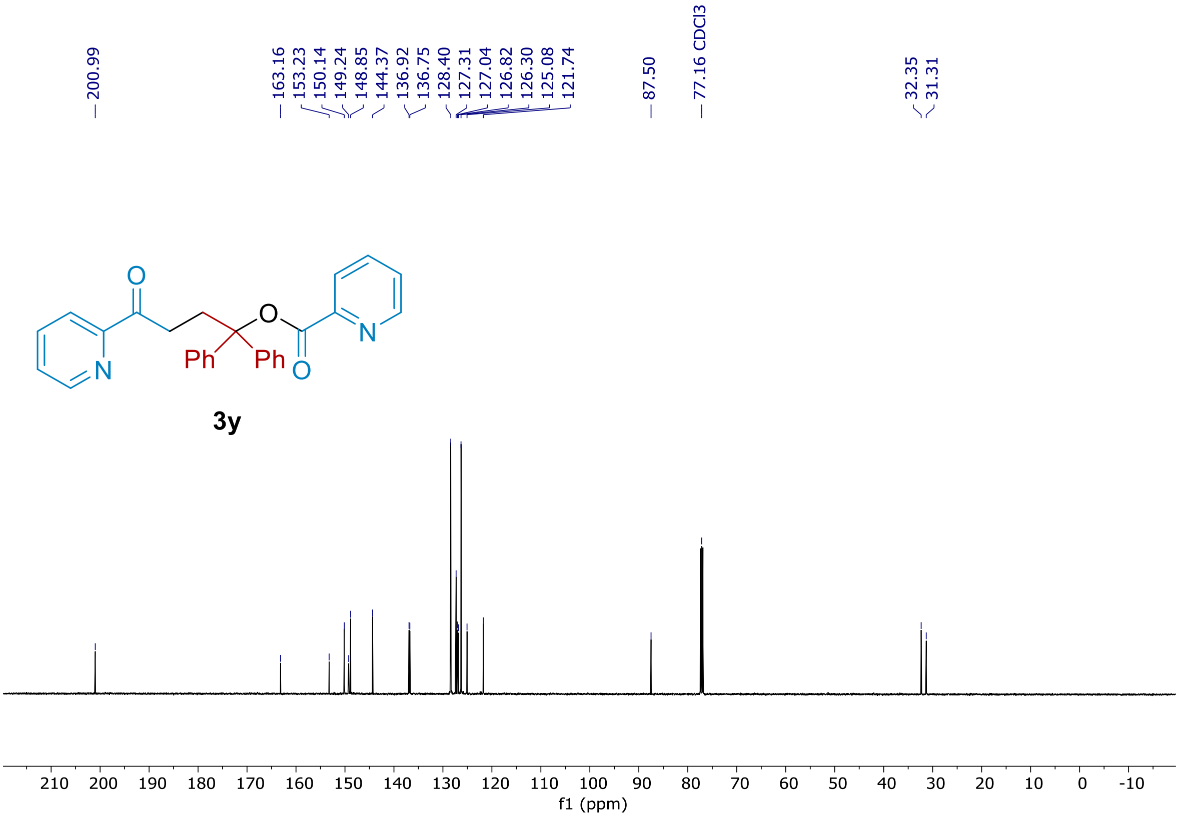

Figure S75. $^{13}C\{^{1}H\}$ NMR spectrum of **3y** in $CDCl_3$ (125 MHz)

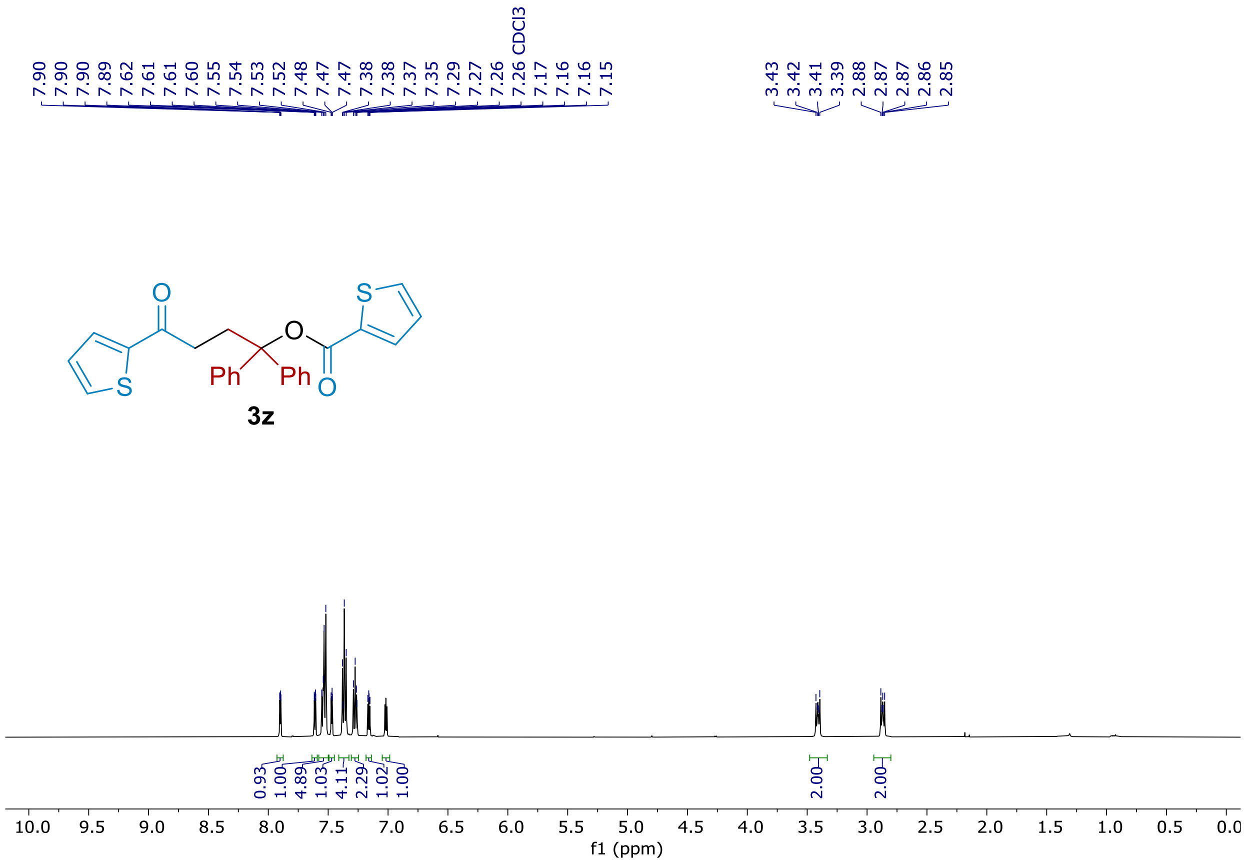

Figure S76. ^{1}H NMR spectrum of **3z** in $CDCl_3$ (500 MHz)

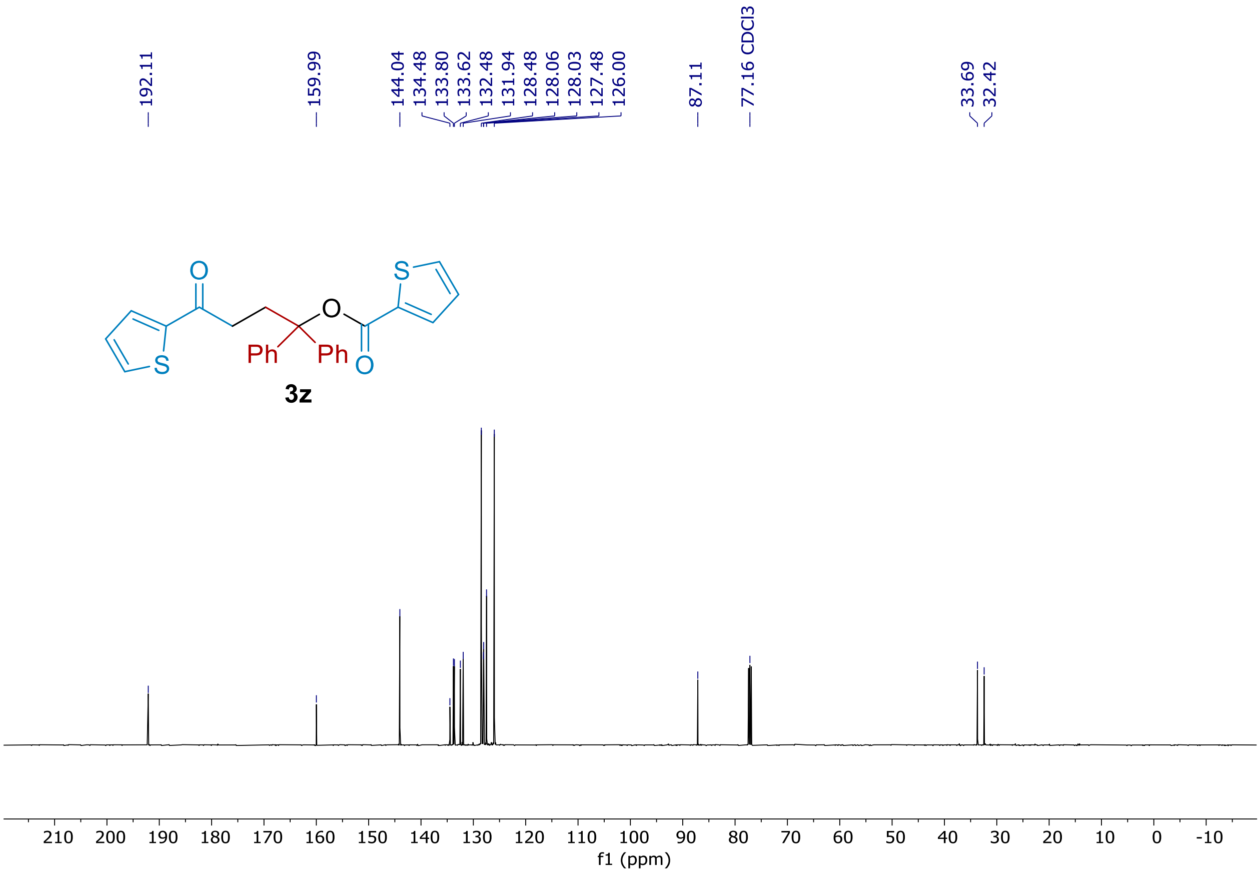

Figure S77. $^{13}C\{^{1}H\}$ NMR spectrum of **3z** in $CDCl_3$ (125 MHz)

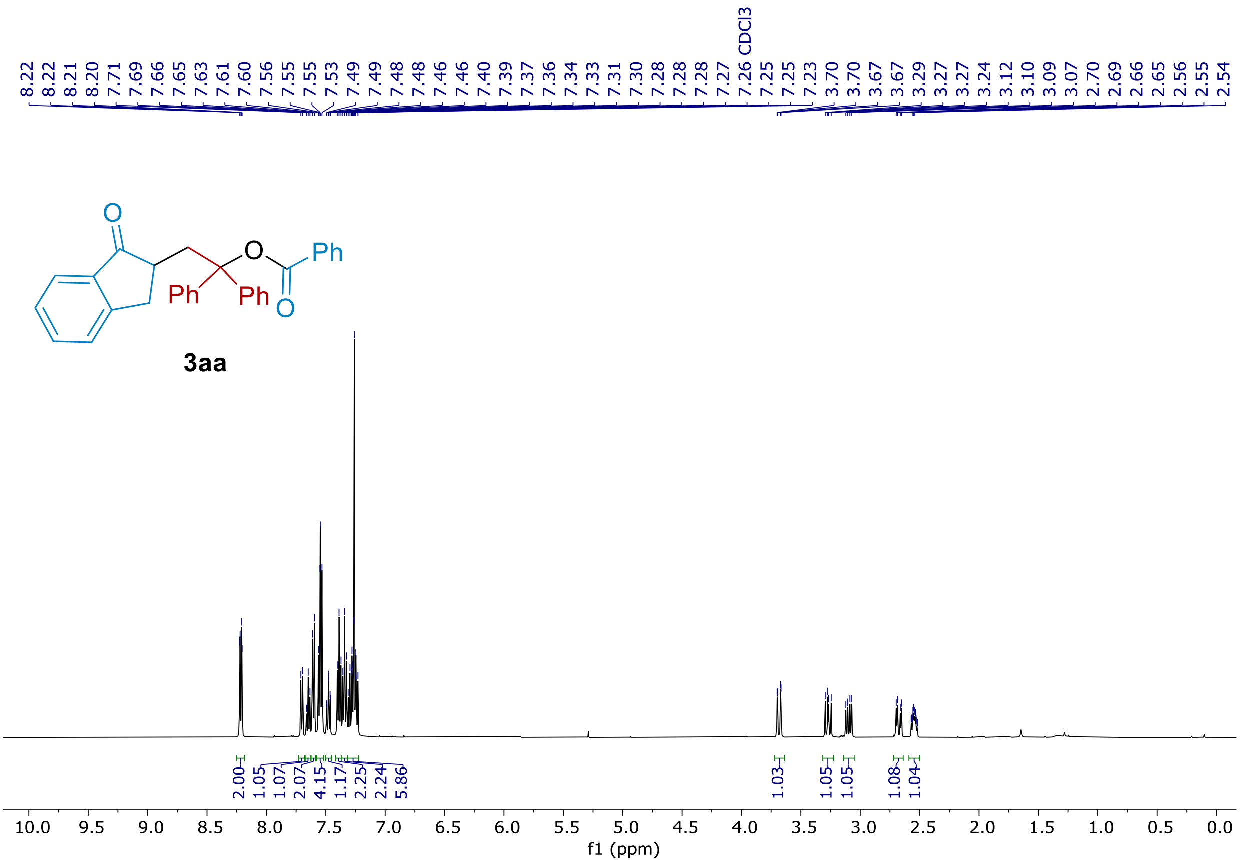

Figure S78. ^{1}H NMR spectrum of **3aa** in $CDCl_3$ (500 MHz)

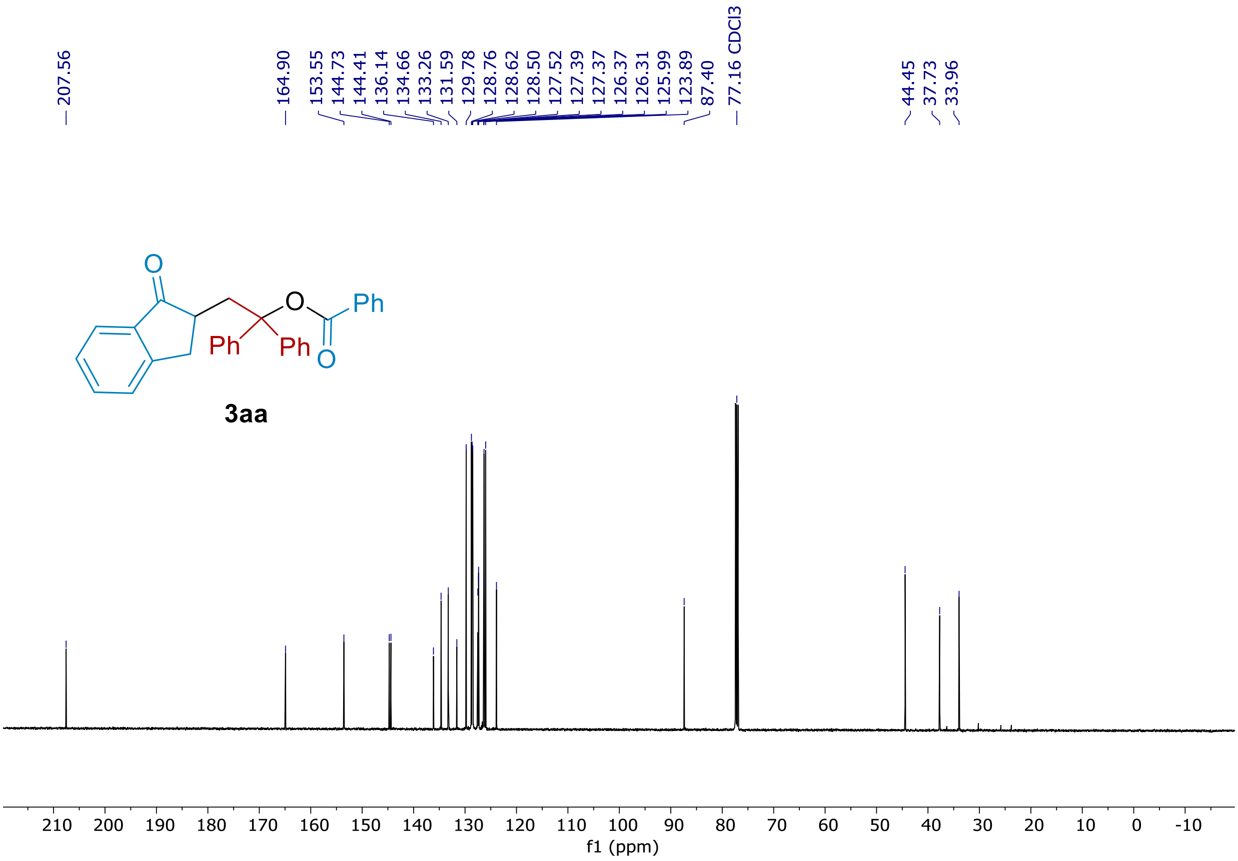

Figure S79. $^{13}C\{^{1}H\}$ NMR spectrum of **3aa** in $CDCl_3$ (125 MHz)

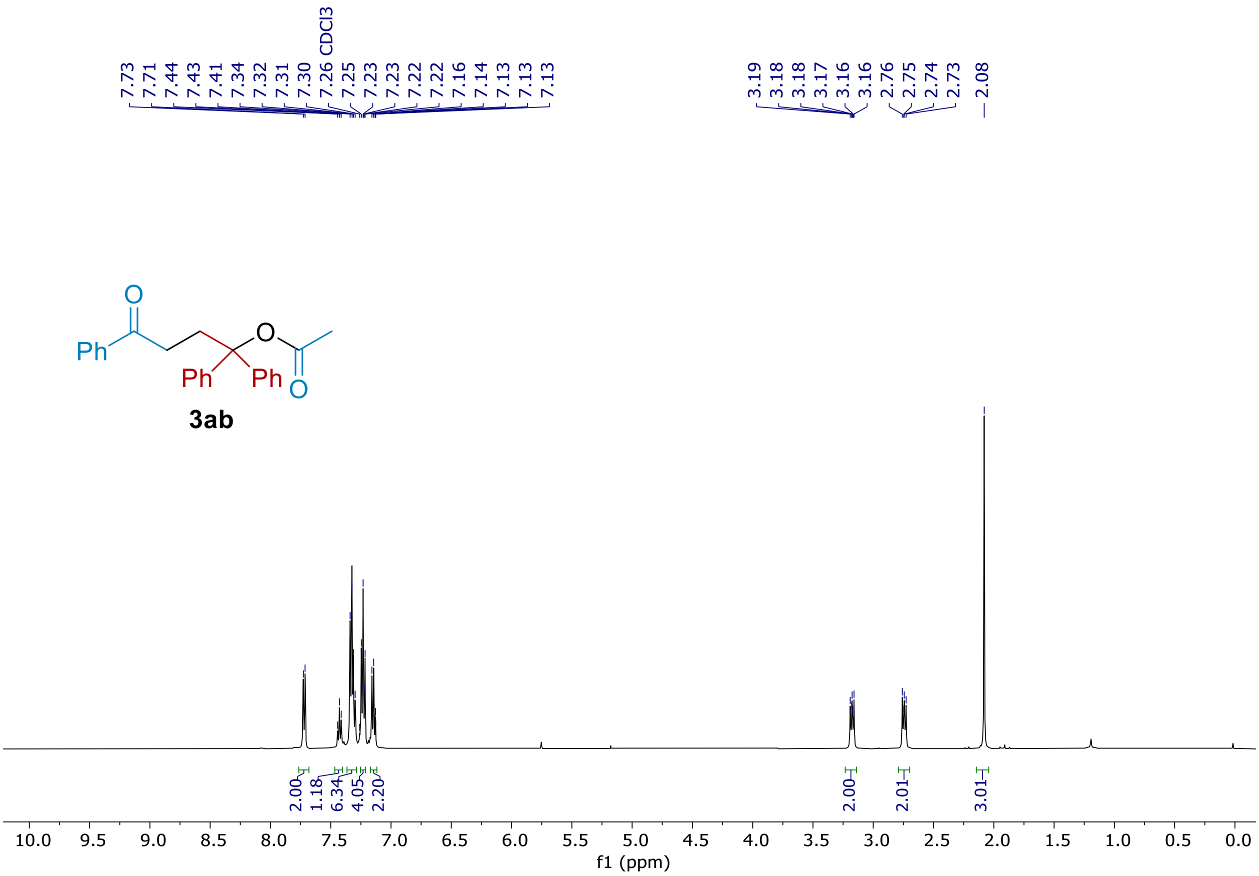

Figure S80. ^{1}H NMR spectrum of **3ab** in $CDCl_3$ (500 MHz)

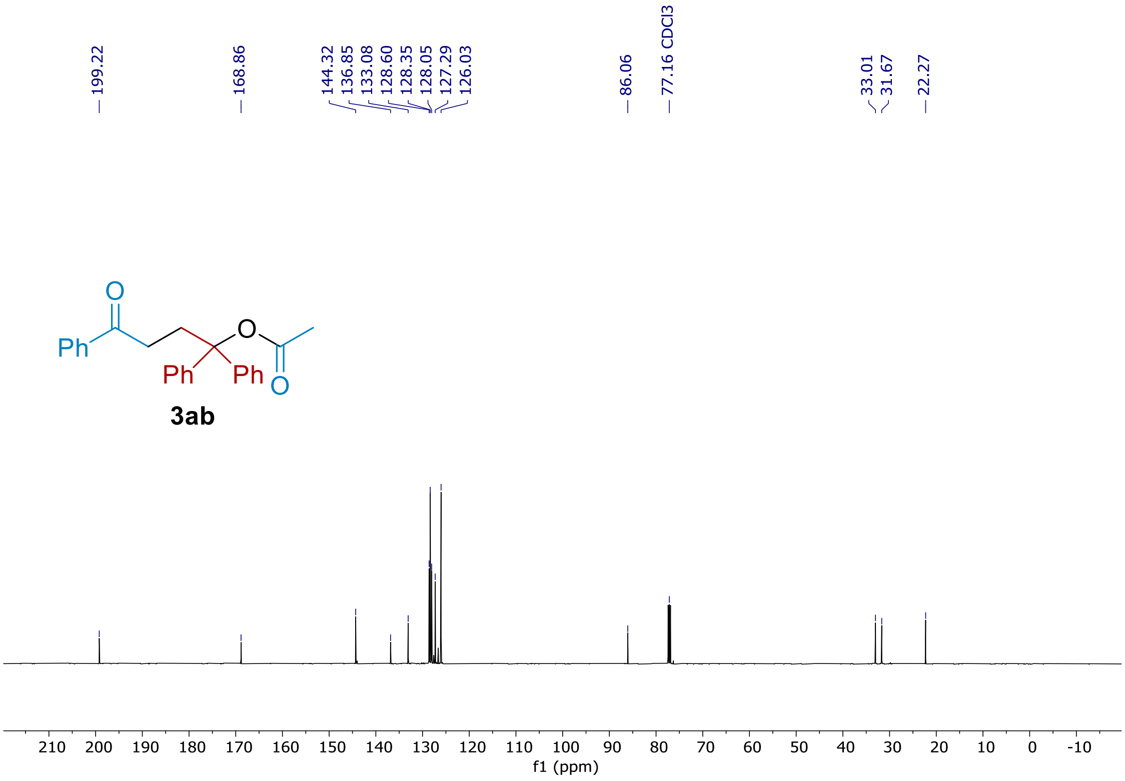

Figure S81. $^{13}C\{^{1}H\}$ NMR spectrum of **3ab** in $CDCl_3$ (125 MHz)

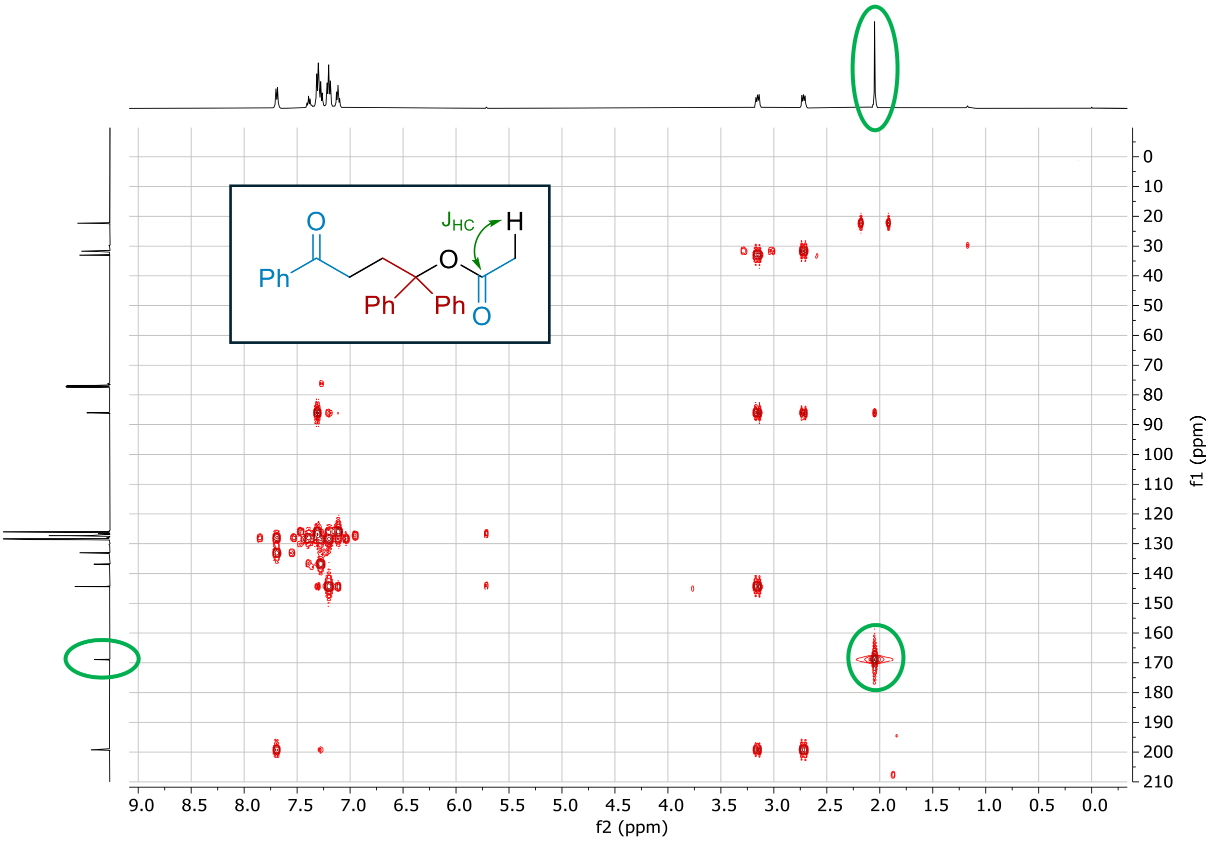

Figure S82. 2D ^{1}H-^{13}C HMBC spectrum of **3ab** in $CDCl_3$. The **2D ^{1}H-^{13}C HMBC** spectrum reveals coupling between the three protons of the methyl group and the ester carbonyl, facilitating the determination of the correct regioisomer.

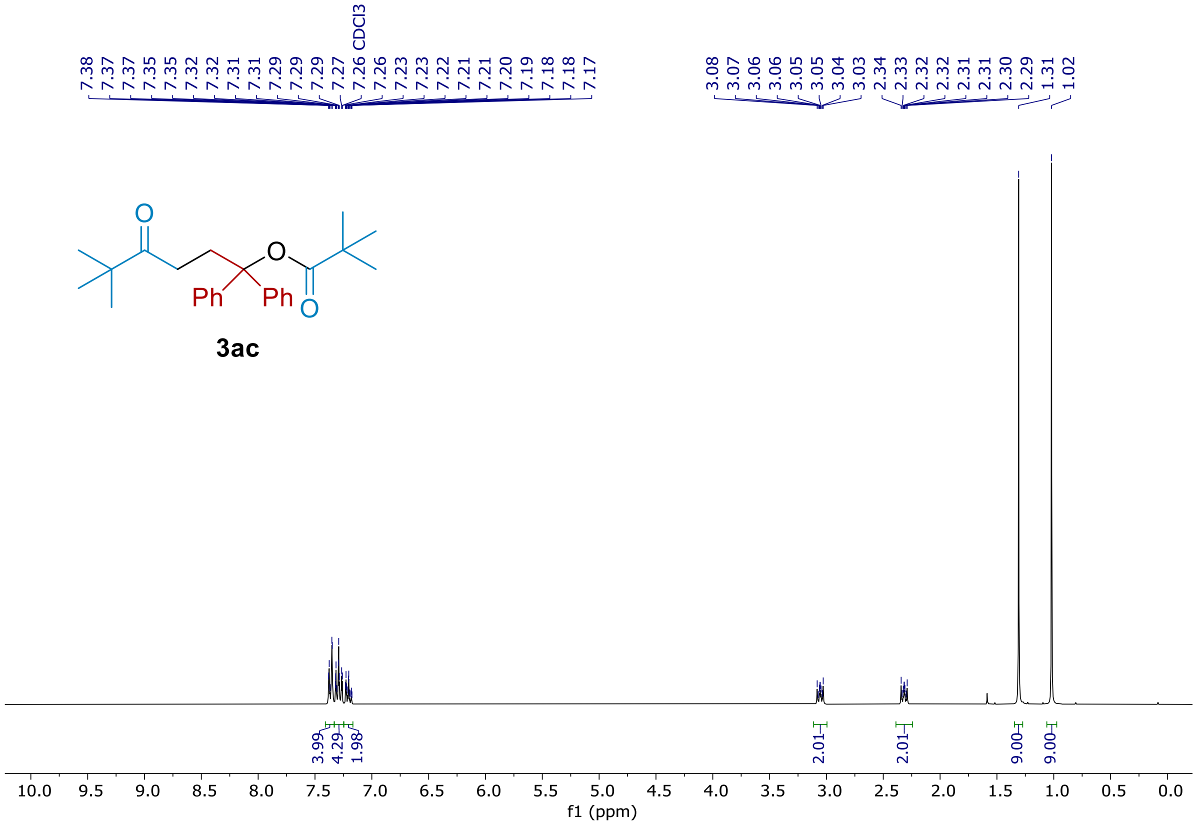

Figure S83. ^{1}H NMR spectrum of **3ac** in $CDCl_3$ (300 MHz)

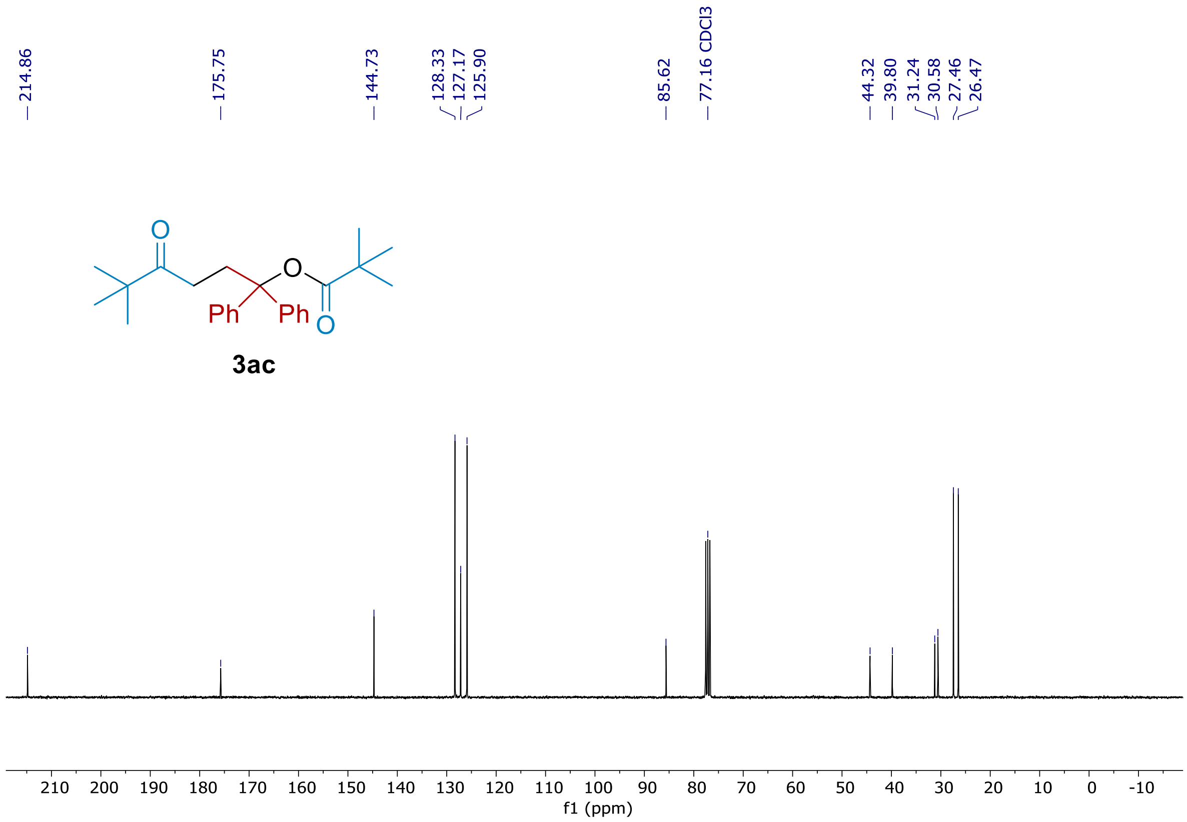

Figure S84. $^{13}C\{^{1}H\}$ NMR spectrum of **3ac** in $CDCl_3$ (75 MHz)

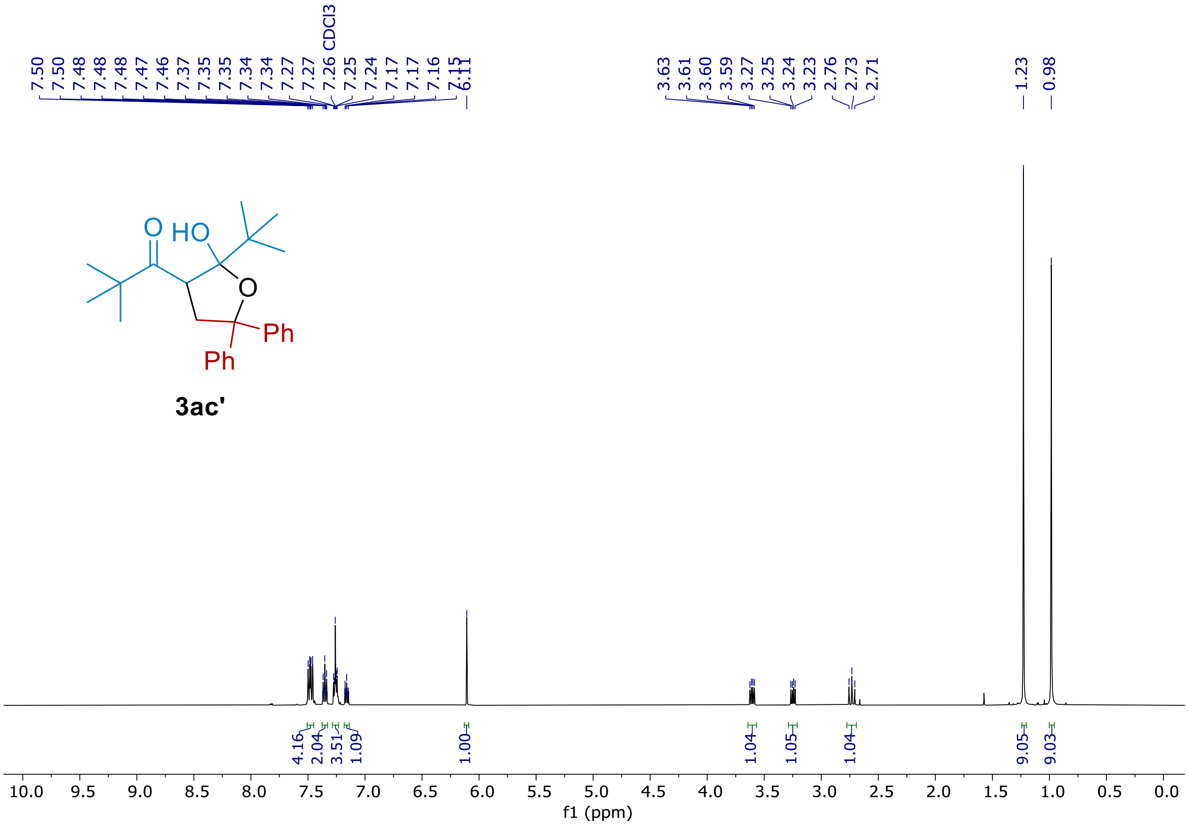

Figure S85. ^{1}H NMR spectrum of **3ac'** in $CDCl_3$ (500 MHz)

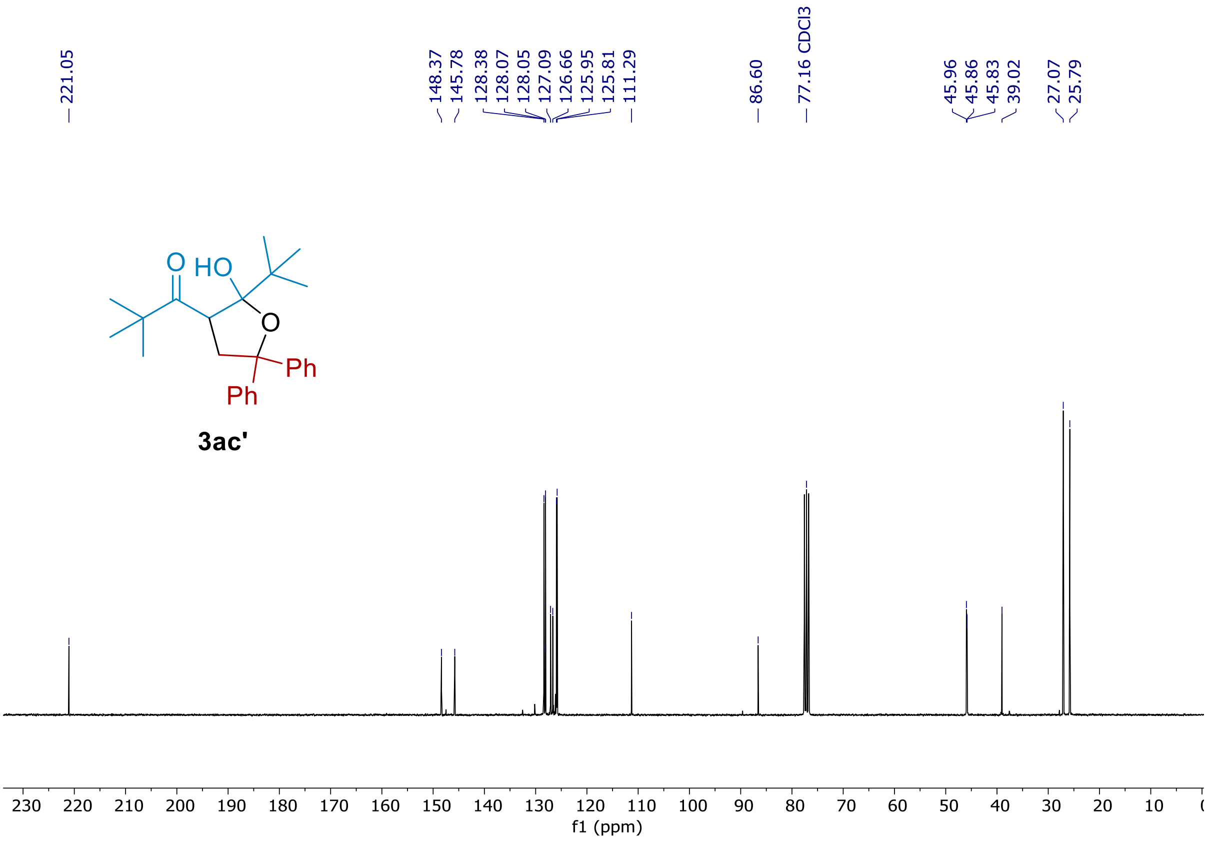

Figure S86. $^{13}C\{^{1}H\}$ NMR spectrum of **3ac'** in $CDCl_3$ (75 MHz)

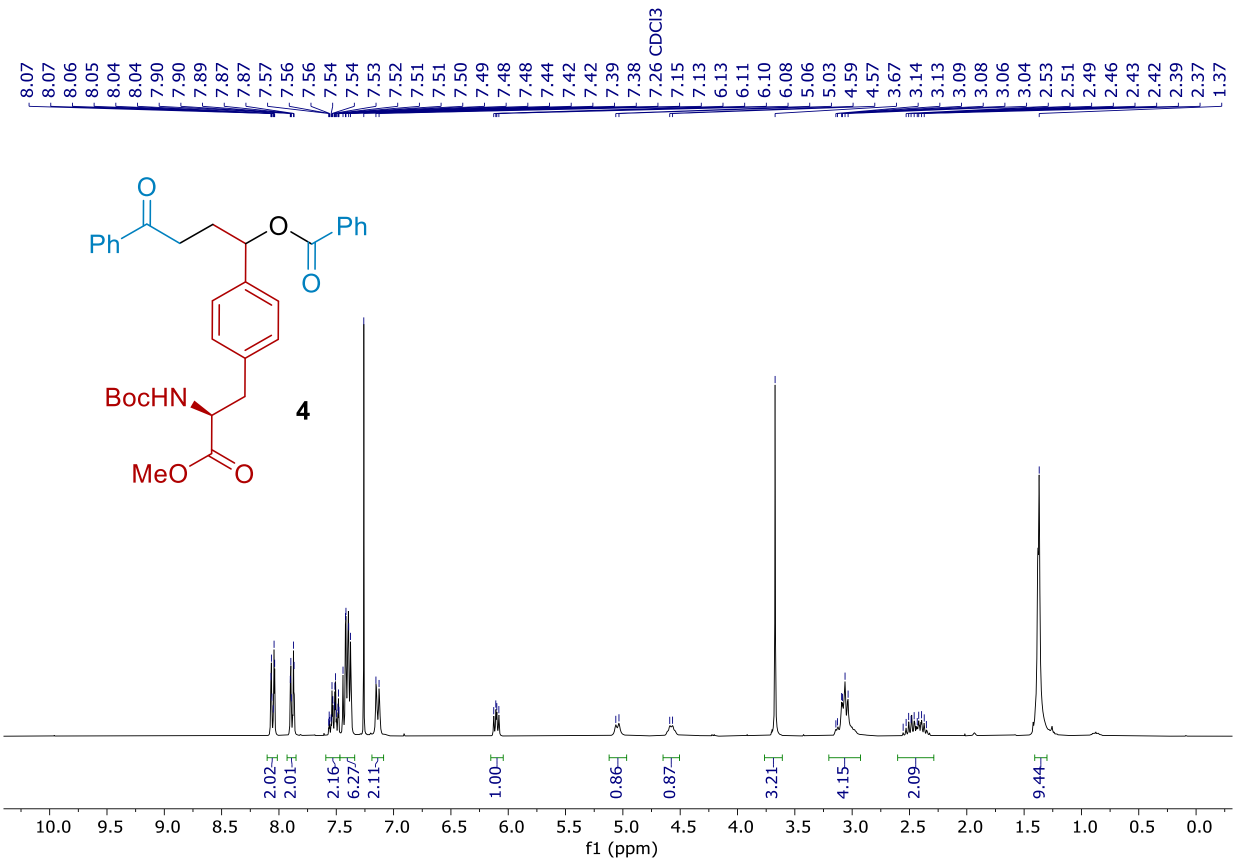

Figure S87. ^{1}H NMR spectrum of **4** in $CDCl_3$ (300 MHz)

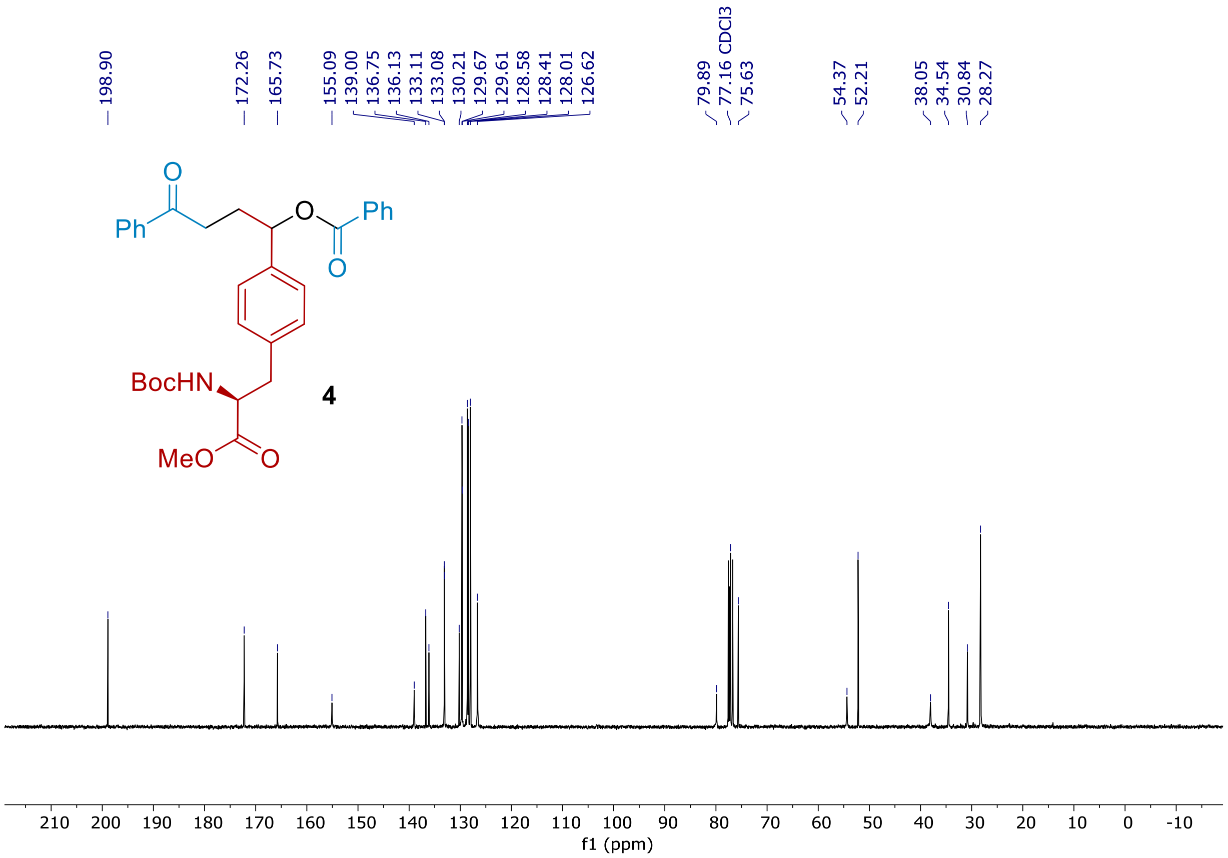

Figure S88. $^{13}C\{^{1}H\}$ NMR spectrum of **4** in $CDCl_3$ (75 MHz)

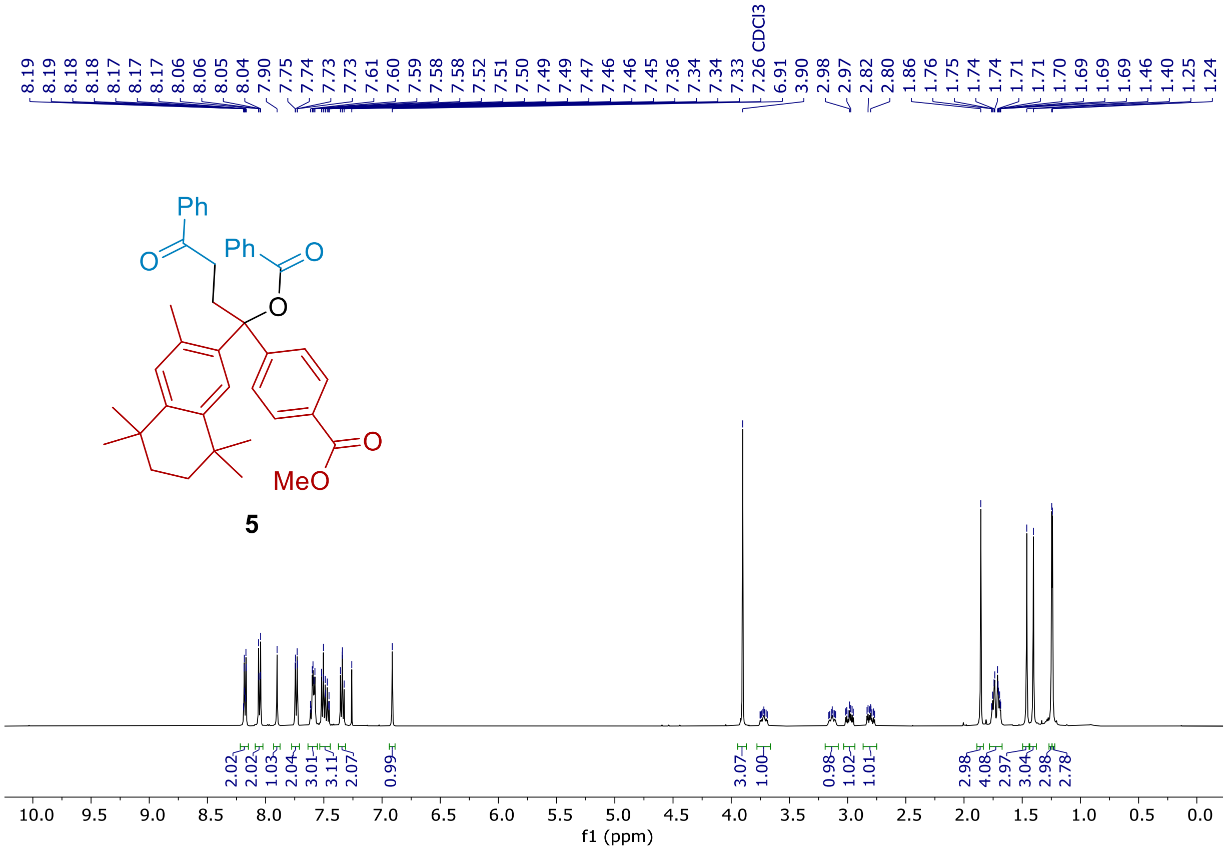

Figure S89. ^{1}H NMR spectrum of **5** in $CDCl_3$ (500 MHz)

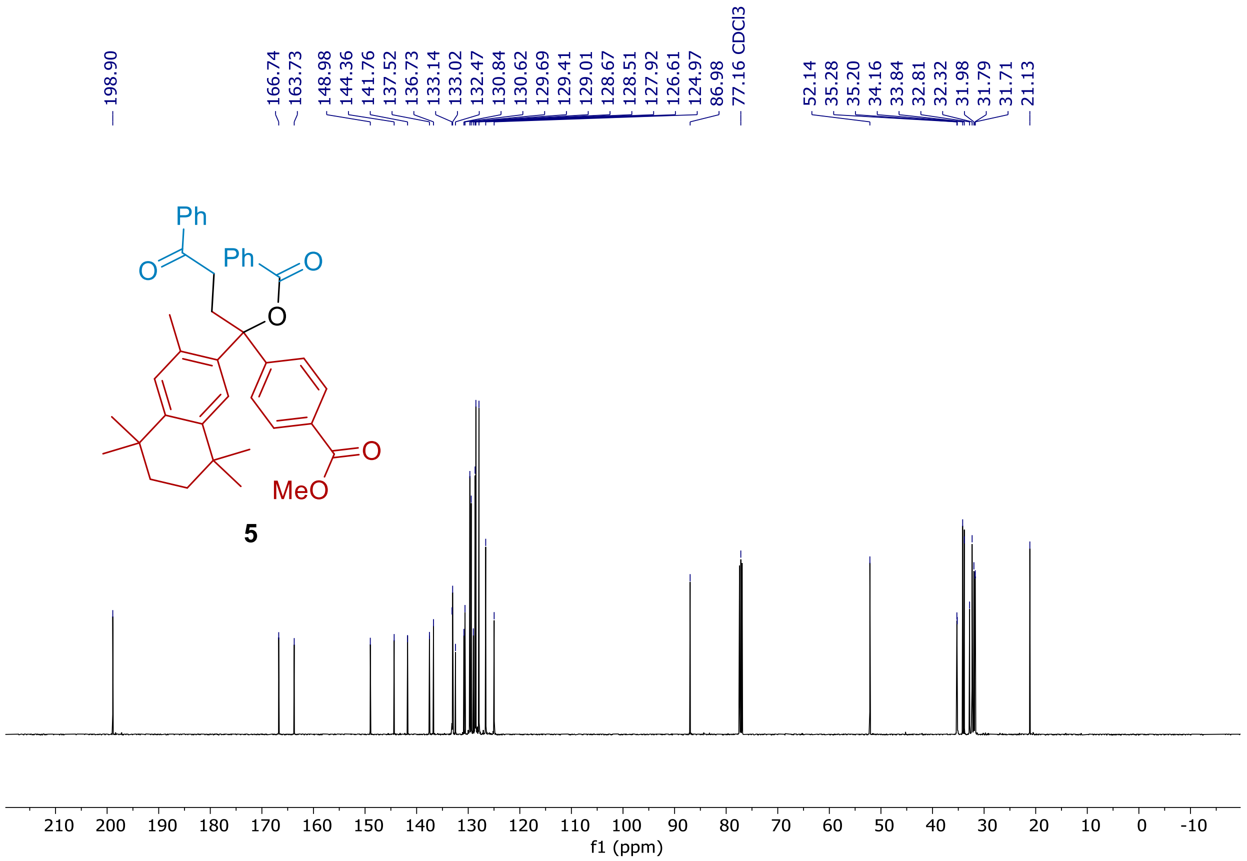

Figure S90. $^{13}C\{^{1}H\}$ NMR spectrum of **5** in $CDCl_3$ (125 MHz)

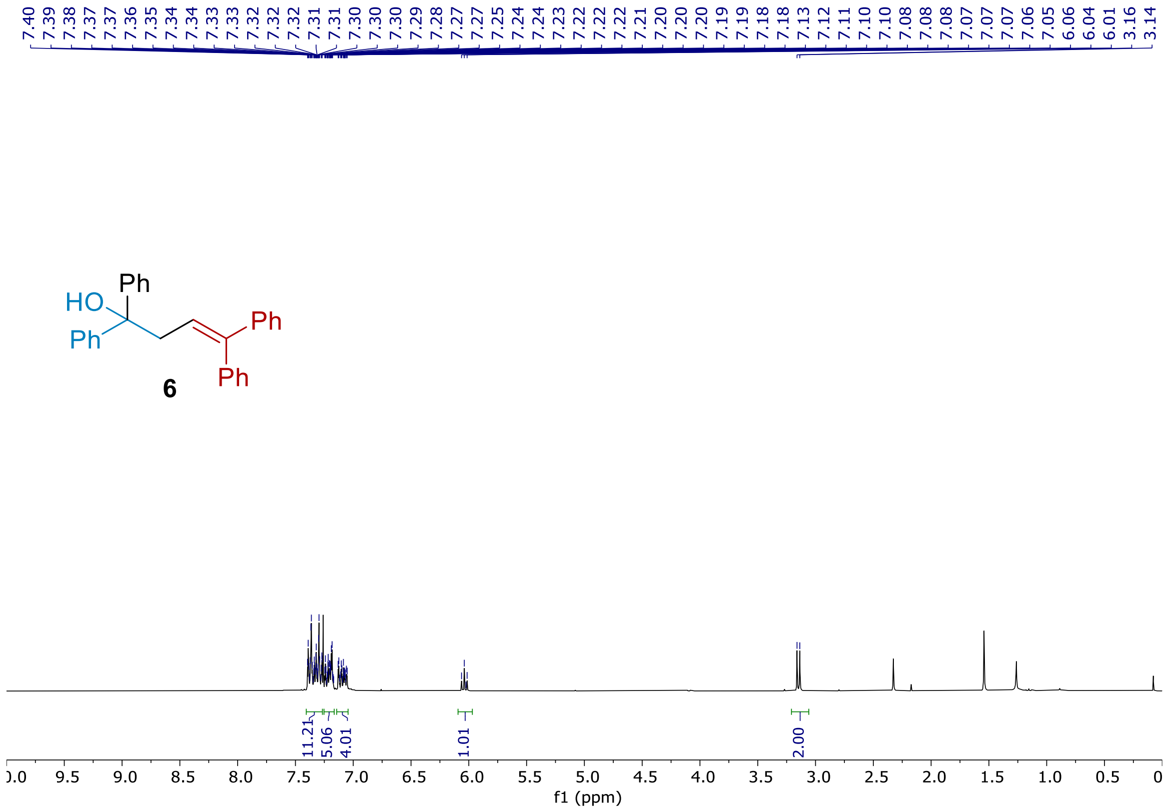

Figure S91. ^{1}H NMR spectrum of **6** in $CDCl_3$ (300 MHz)

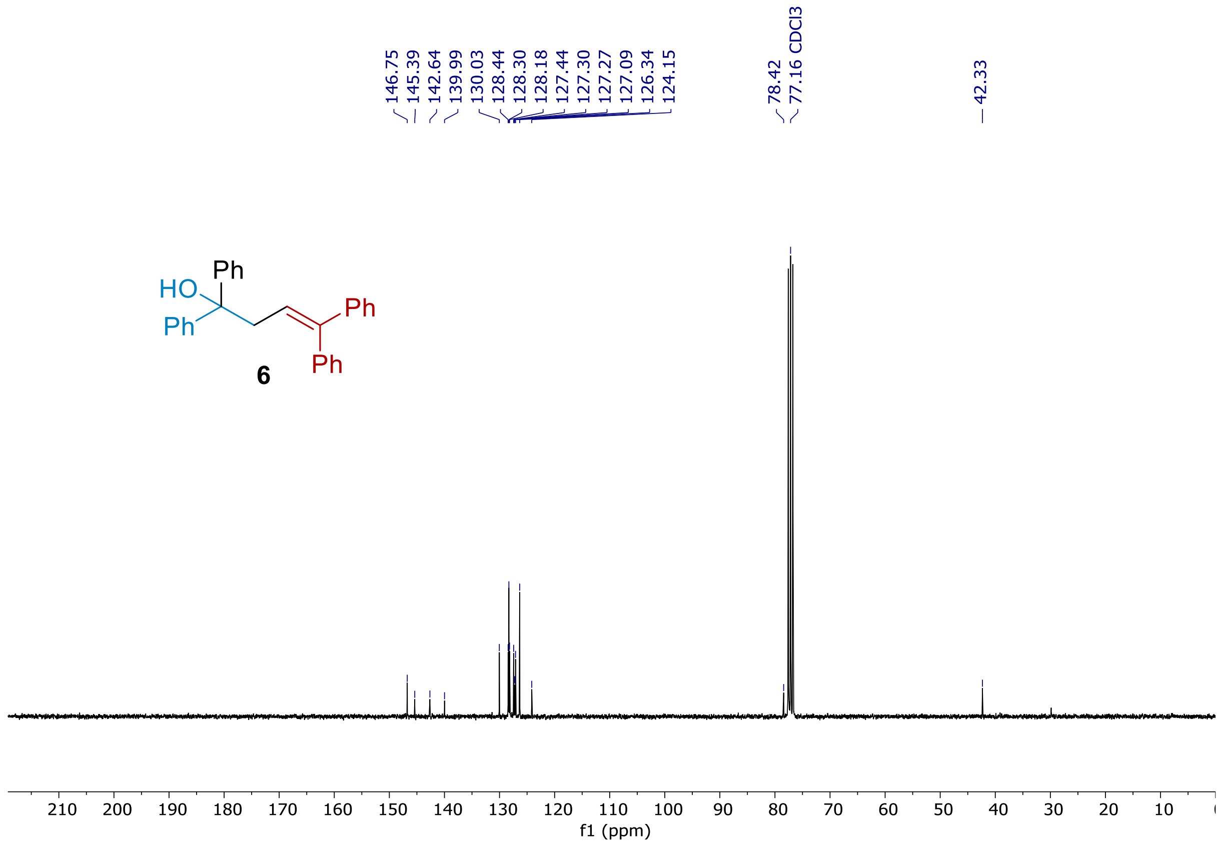

Figure S92. $^{13}C\{^{1}H\}$ NMR spectrum of **6** in $CDCl_3$ (75 MHz)

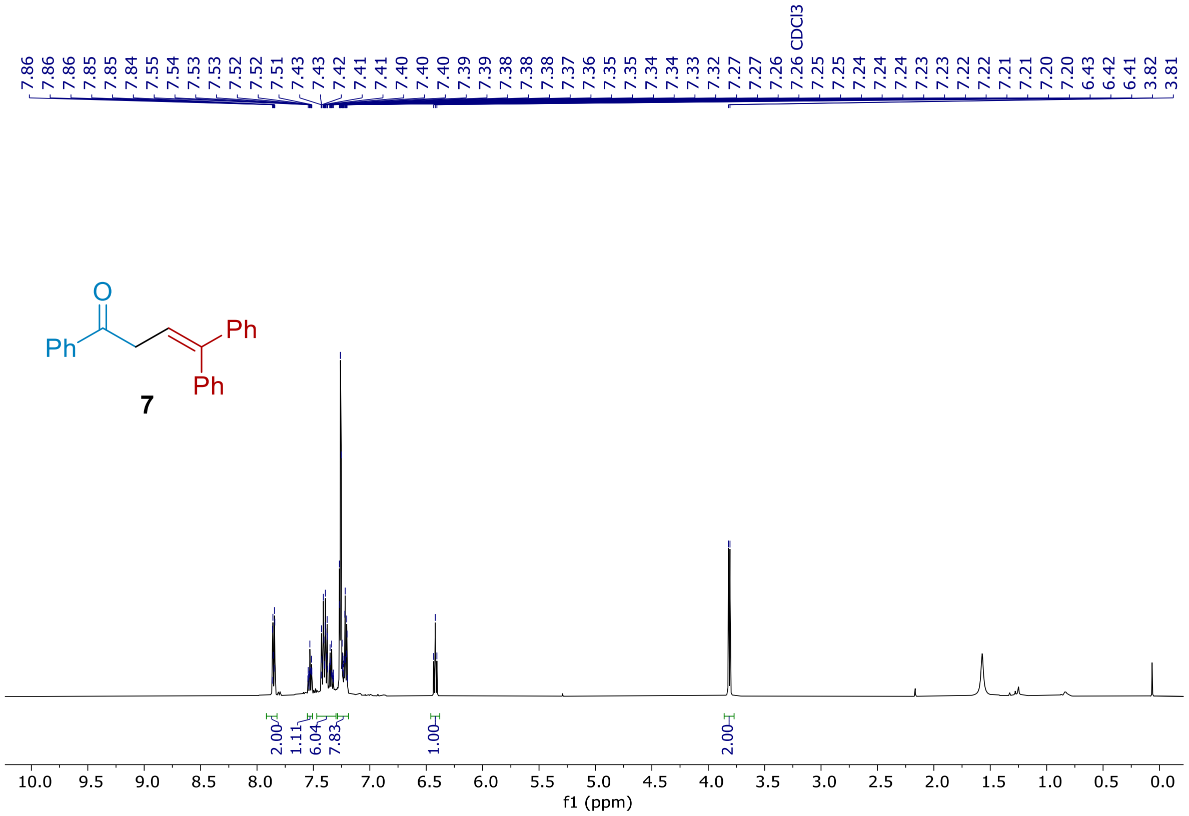

Figure S93. ^{1}H NMR spectrum of **7** in $CDCl_3$ (500 MHz)

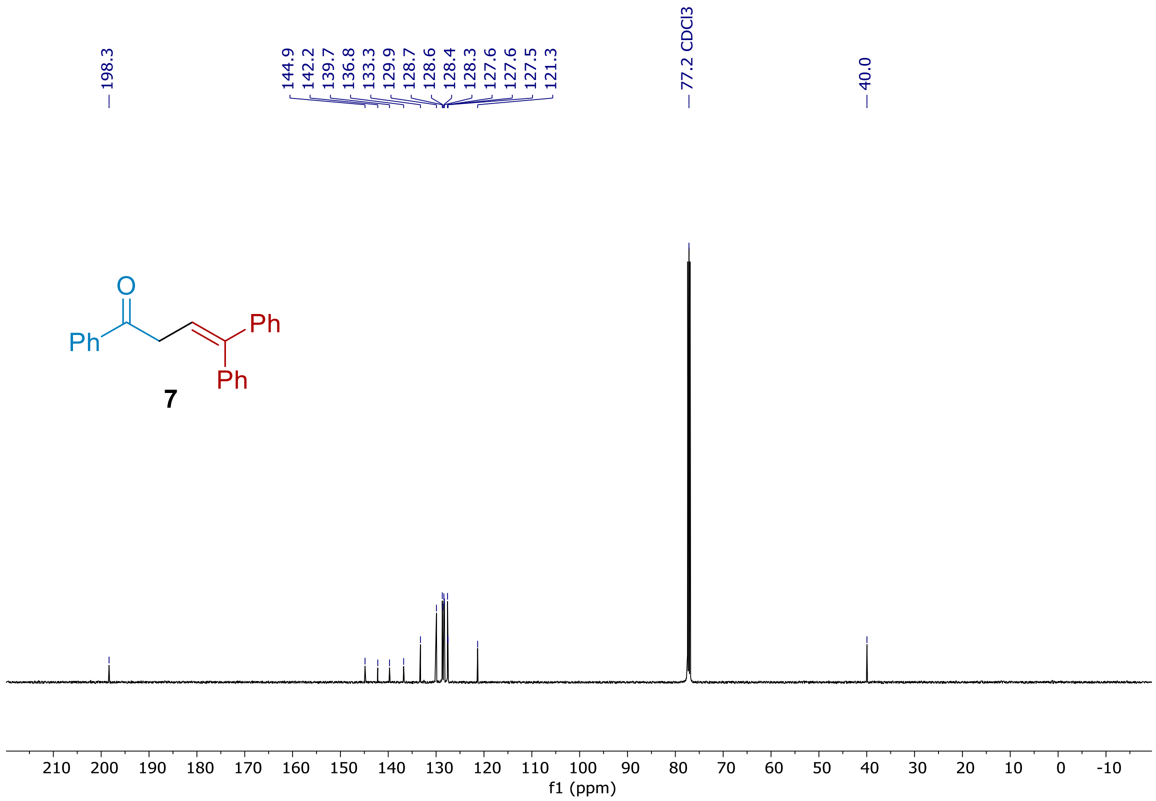

Figure S94. $^{13}C\{^{1}H\}$ NMR spectrum of **7** in $CDCl_3$ (125 MHz)

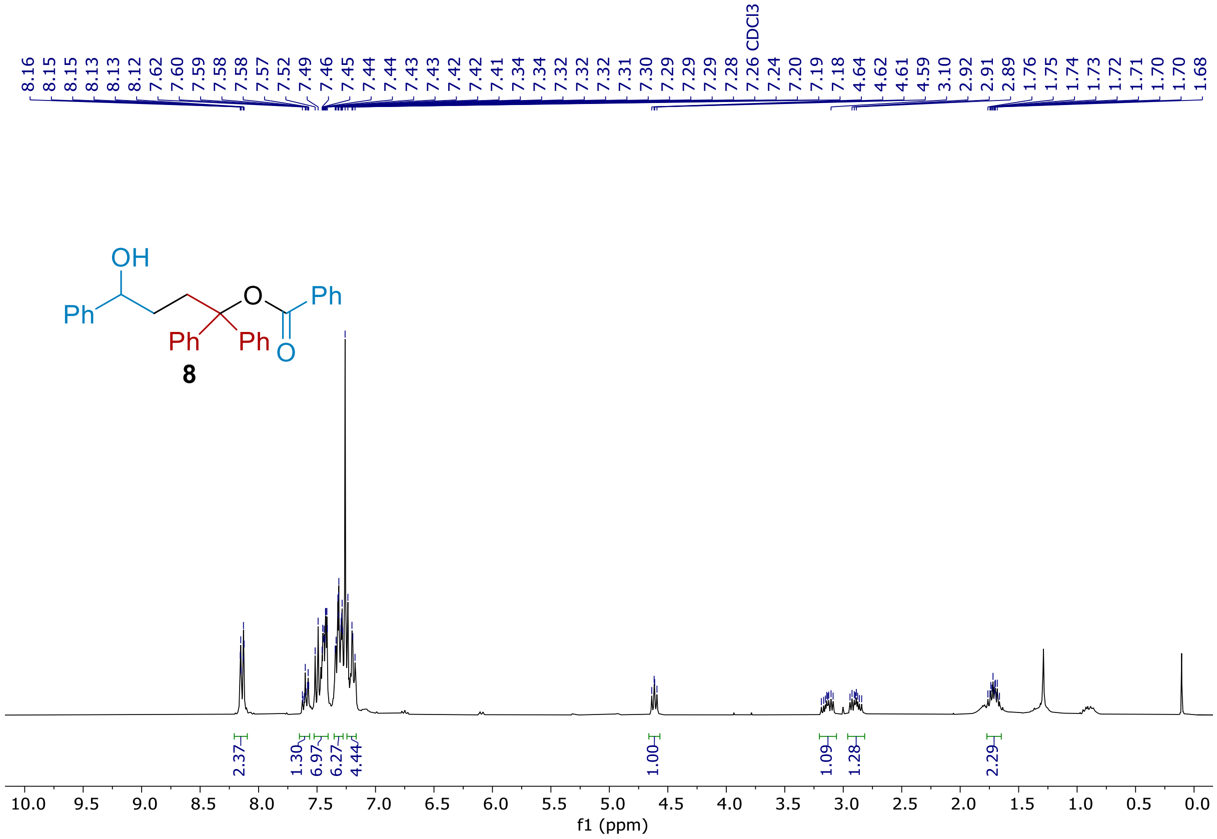

Figure S95. ^{1}H NMR spectrum of **8** in $CDCl_3$ (300 MHz)

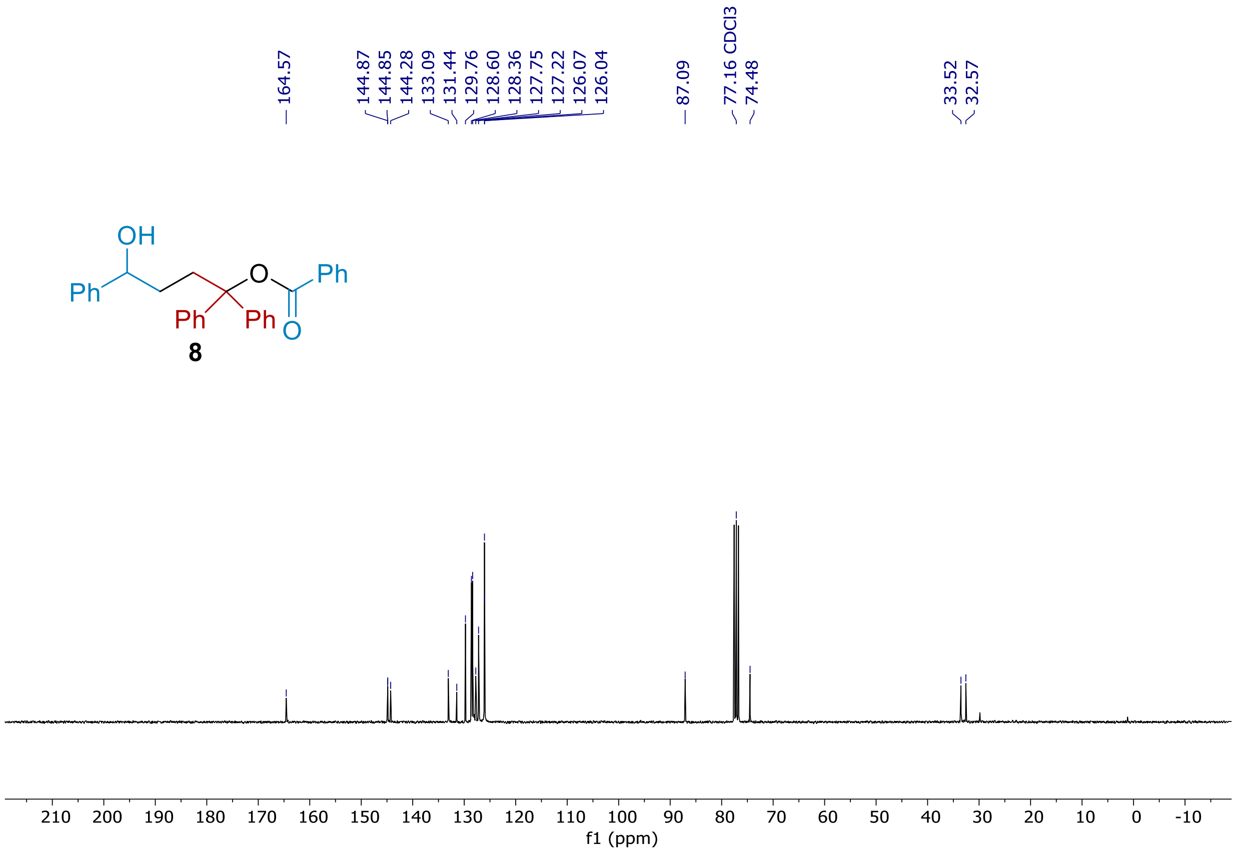

Figure S96. $^{13}C\{^{1}H\}$ NMR spectrum of **8** in $CDCl_3$ (75 MHz)

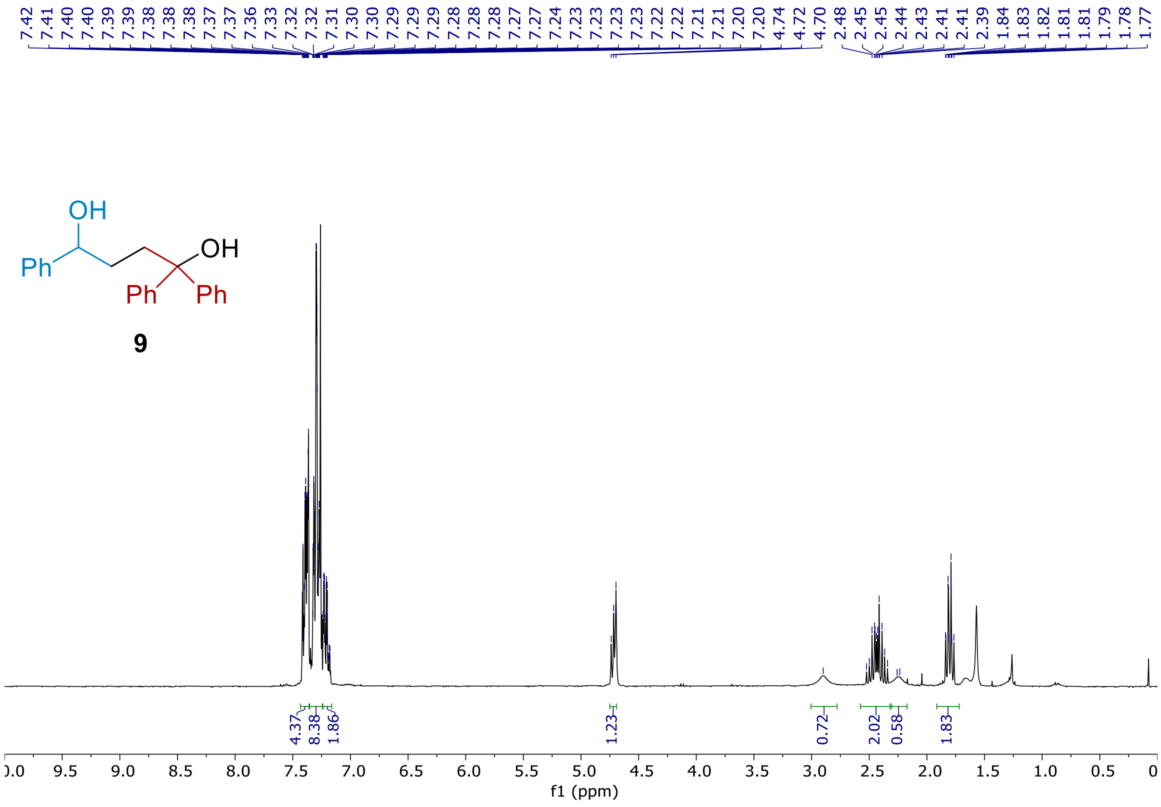

Figure S97. ^{1}H NMR spectrum of **9** in $CDCl_3$ (300 MHz)

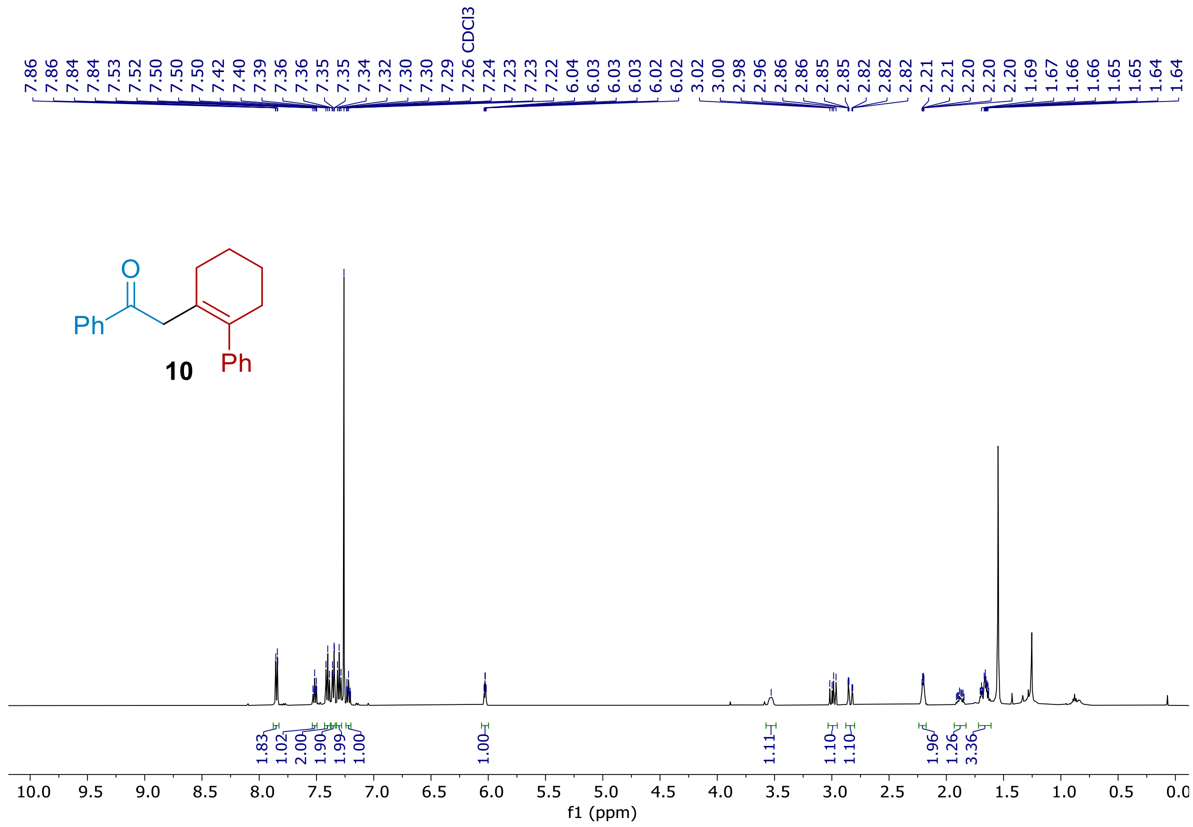

Figure S98. ^{1}H NMR spectrum of **10** in $CDCl_3$ (500 MHz)

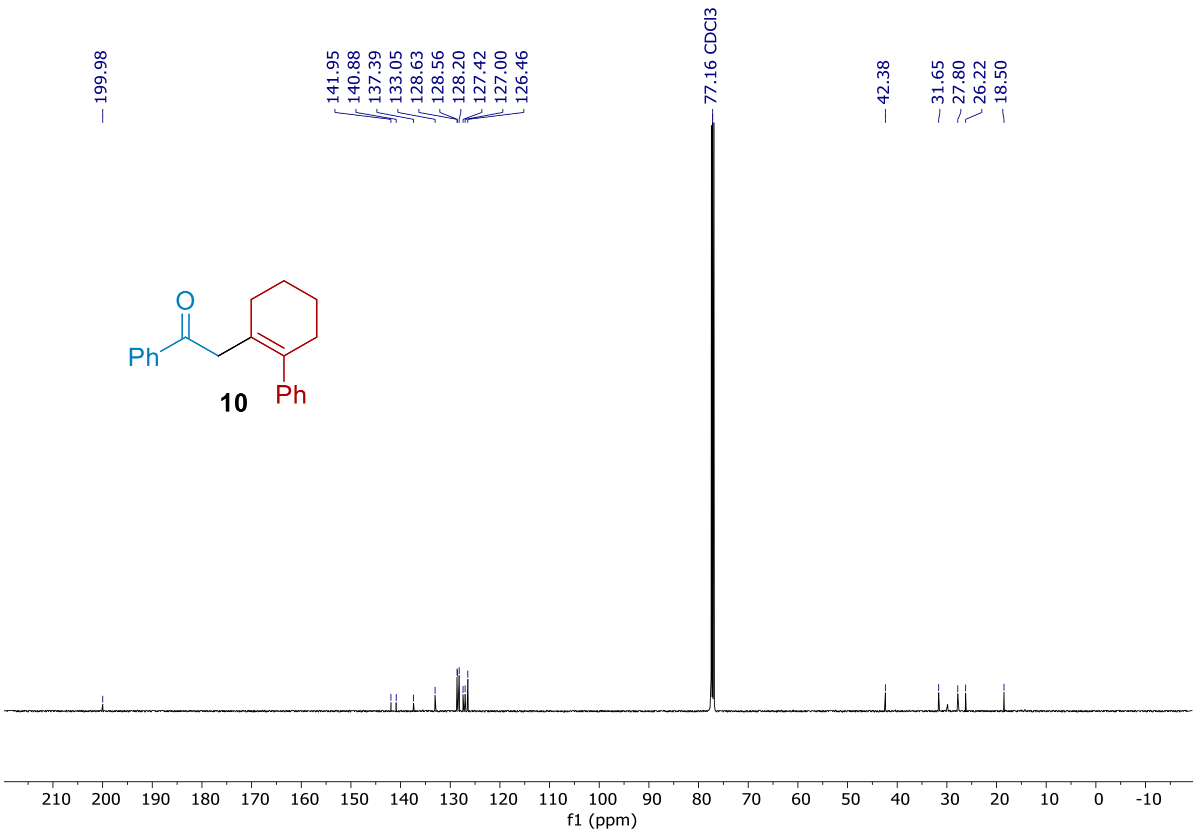

Figure S99. $^{13}C\{^{1}H\}$ NMR spectrum of **10** in $CDCl_3$ (125 MHz)

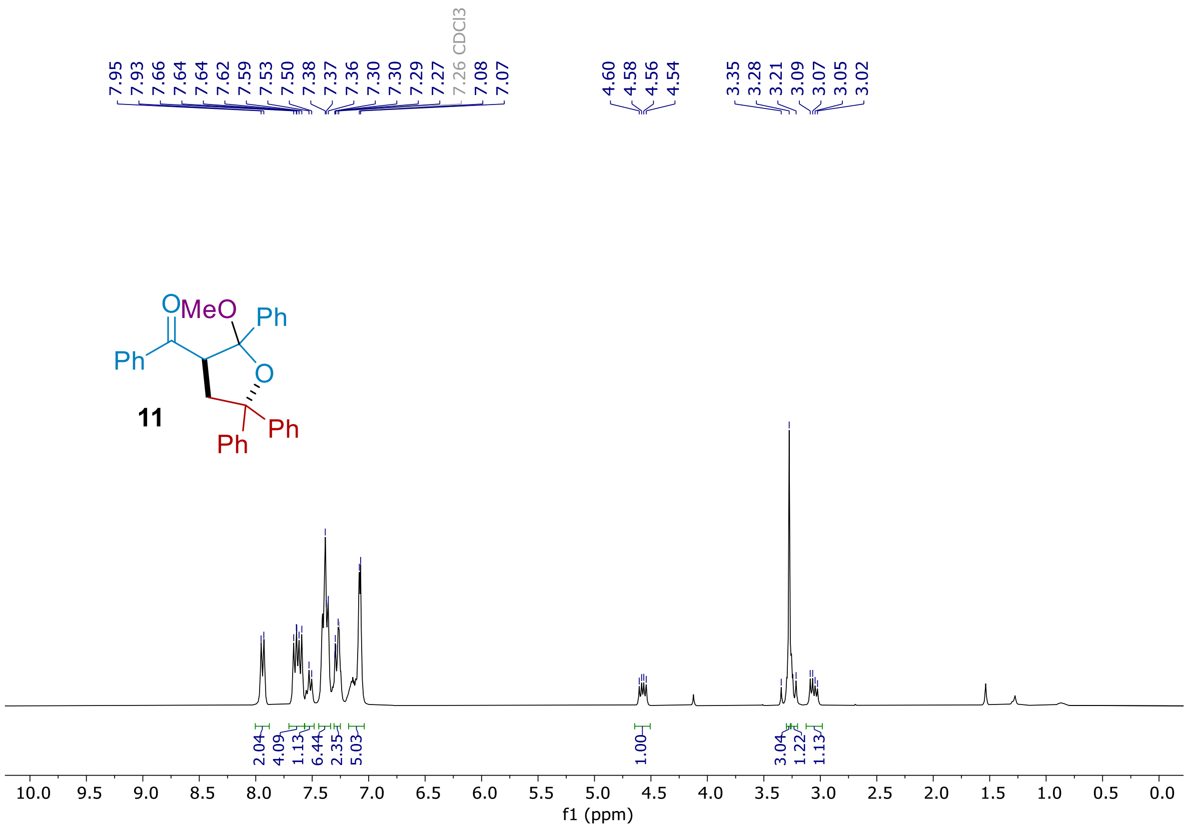

Figure S100. ^{1}H NMR spectrum of **11** in $CDCl_3$ (300 MHz)

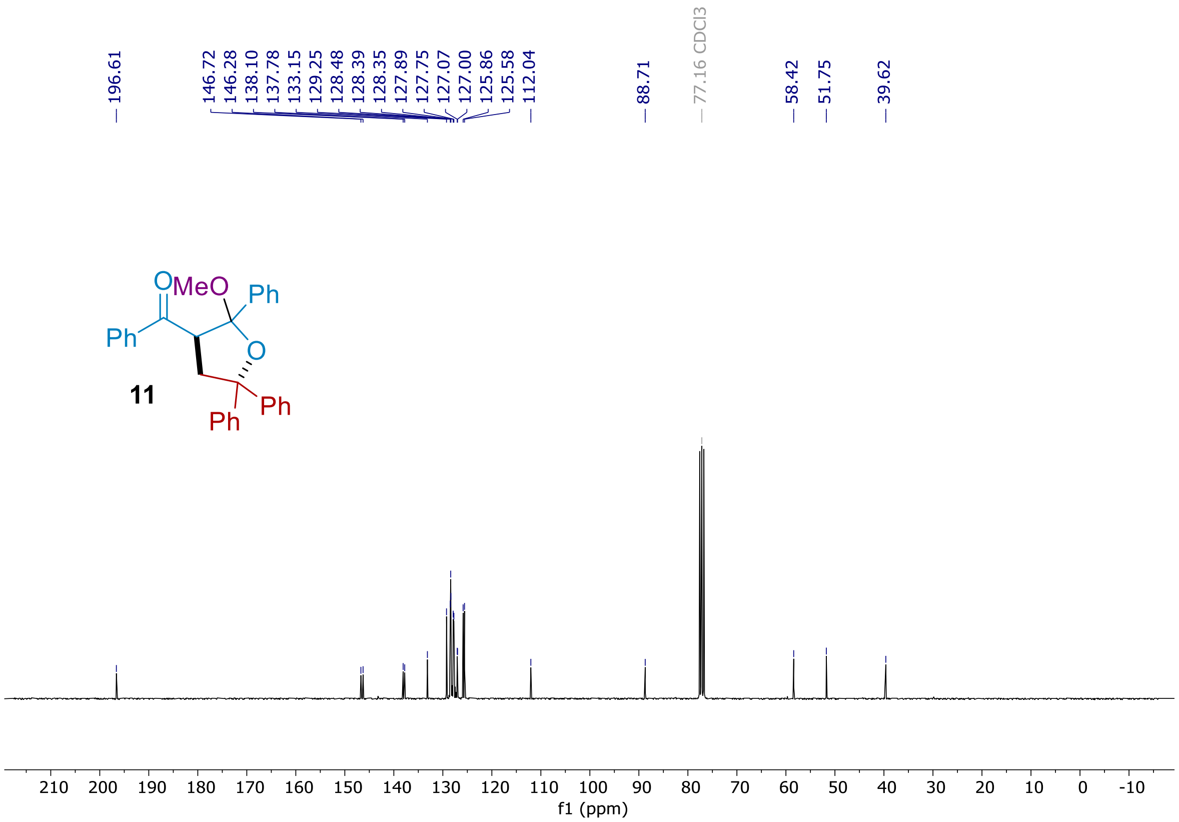

Figure S101. $^{13}C\{^{1}H\}$ NMR spectrum of **11** in $CDCl_3$ (75 MHz)

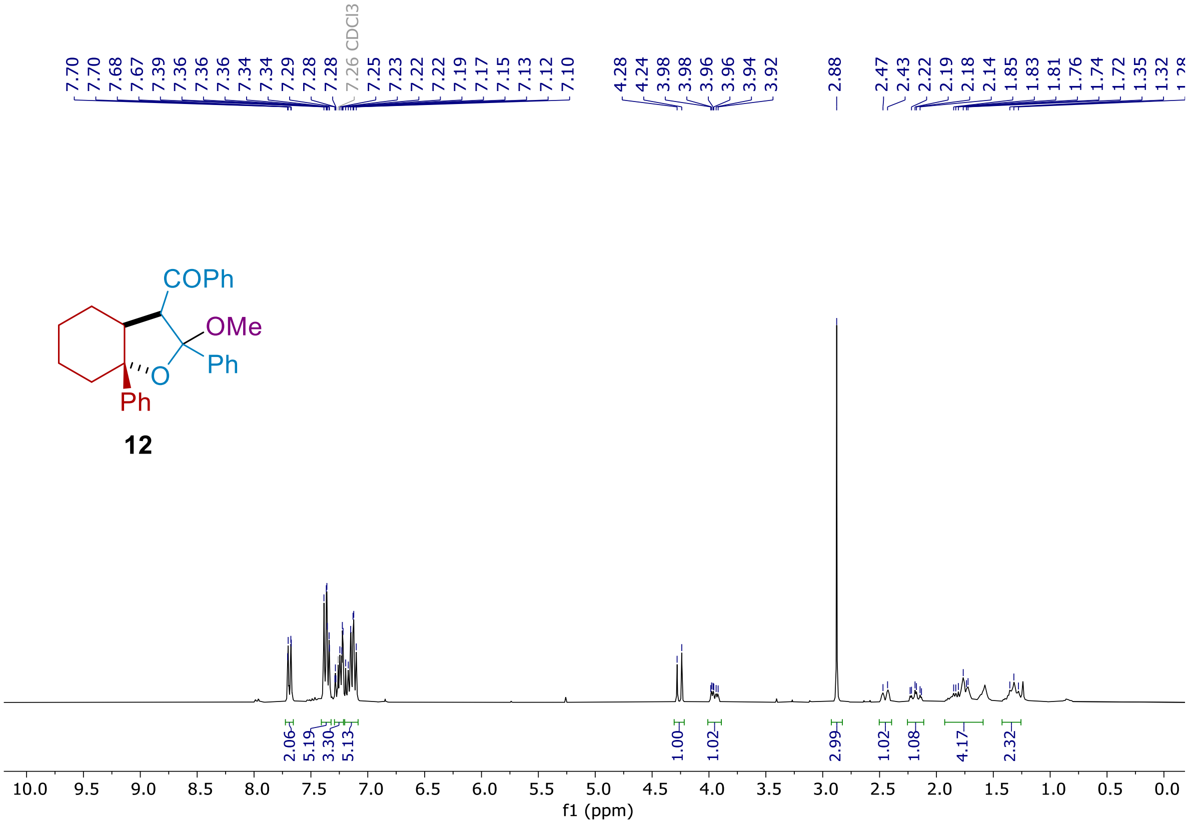

Figure S102. ^{1}H NMR spectrum of **12** in $CDCl_3$ (300 MHz)

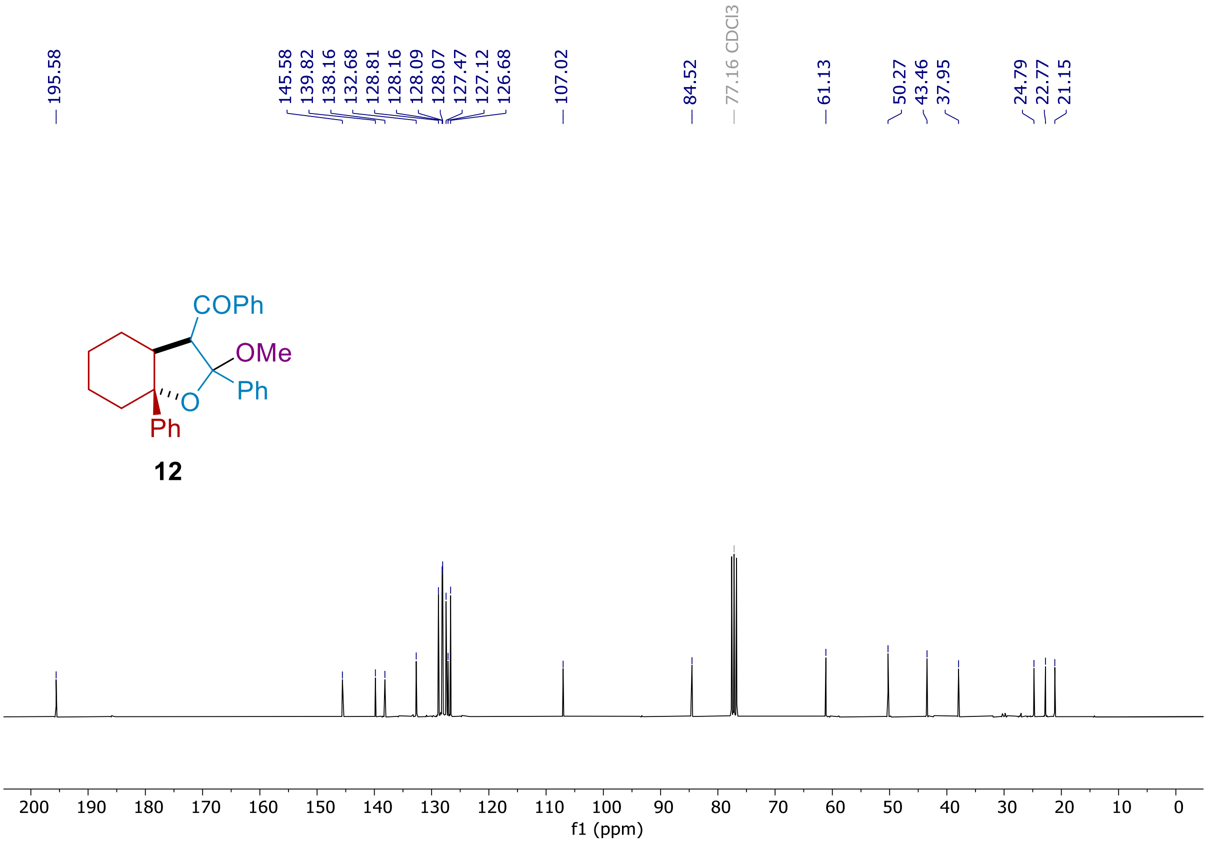

Figure S103. $^{13}C\{^{1}H\}$ NMR spectrum of **12** in $CDCl_3$ (75 MHz)

8. Single Crystal X-Ray Diffraction of 3c, 3aa, 3d and 3e

Table S2. Sample and crystal data for **3c**

**Chemical formula**	$\mathbf{C_{24}H_{22}O_3}$	
**Formula weight**	358.41 g/mol	
**Temperature**	250.00(10) K	
**Wavelength**	1.54184 Å	
**Crystal size**	0.30×0.06×0.05 mm^3	
**Crystal habit**	Clear colourless needle	
**Crystal system**	monoclinic	
**Space group**	$P2_1/n$	
**Unit cell dimensions**	a = 13.77240(10) Å	α = 90°
	b = 5.96940(10) Å	β =102.9420(10)°
	c = 24.0744(2) Å	γ = 90°
**Volume**	1928.95(4) $Å^3$	
**Z**	4	
**Density (calculated)**	1.234 g/cm^3	
**Absorption coefficient**	0.640 mm^{-1}	
**GooF**	1.048	
**wR_2 (all data)**	0.0907	
**R_1**	0.0346	

Table S3. Sample and crystal data for **3aa**

**Chemical formula**	$\mathbf{C_{30}H_{24}O_3}$	
**Formula weight**	358.41 g/mol	
**Temperature**	250.00(10) K	
**Wavelength**	1.54184 Å	
**Crystal size**	0.31×0.20×0.08 mm^3	
**Crystal habit**	Clear colourless prism	
**Crystal system**	monoclinic	
**Space group**	$P2_1/n$	
**Unit cell dimensions**	a = 9.40690(10) Å	α = 90°
	b = 11.23140(10) Å	β = 94.7470(10)°
	c = 21.9930(2) Å	γ = 90°
**Volume**	2315.65(4) $Å^3$	
**Z**	4	
**Density (calculated)**	1.241 g/cm^3	
**Absorption coefficient**	0.626 mm^{-1}	
**GooF**	1.017	
**wR_2 (all data)**	0.0983	
**R_1**	0.0376	

Table S4. Sample and crystal data for **3d**

**Chemical formula**	$\mathbf{C_{29.5}H_{24}ClO_{3.83}}$	
**Formula weight**	475.26 g/mol	
**Temperature**	149.97(10) K	
**Wavelength**	1.54184 Å	
**Crystal size**	0.08×0.07×0.06 mm^3	
**Crystal habit**	Clear colourless prism	
**Crystal system**	orthorhombic	
**Space group**	*Pnn2*	
**Unit cell dimensions**	a = 22.0330(4) Å	α = 90°
	b = 20.0329(4) Å	β = 90°
	c = 5.75720(10) Å	γ = 90°
**Volume**	2541.14(8) $Å^3$	
**Z**	4	
**Density (calculated)**	1.234 g/cm^3	
**Absorption coefficient**	1.586 mm^{-1}	
**GooF**	1.044	
**wR_2 (all data)**	0.2087	
**R_1**	0.0702	

Table S5. Sample and crystal data for **3e**

**Chemical formula**	$\mathbf{C_{30.5}H_{27}ClO_3}$	
**Formula weight**	476.97 g/mol	
**Temperature**	250.00(10) K	
**Wavelength**	1.54184 Å	
**Crystal size**	0.24×0.09×0.06 mm^3	
**Crystal habit**	Clear colourless prism	
**Crystal system**	monoclinic	
**Space group**	$P2_{1/c}$	
**Unit cell dimensions**	a = 11.63760(10) Å	α = 90°
	b = 6.01690(10) Å	β = 99.0360(10) °
	c = 36.5654(4) Å	γ = 90°
**Volume**	2528.62(6) $Å^3$	
**Z**	4	
**Density (calculated)**	1.253 g/cm^3	
**Absorption coefficient**	1.568 mm^{-1}	
**GooF**	1.040	
**wR_2 (all data)**	0.1144	
**R_1**	0.0396	

9. References

1. X. Wu, C. N. Gannett, J. Liu, R. Zeng, L. F. T. Novaes, H. Wang, H. D. Abruña and S. Lin, Intercepting Hydrogen Evolution with Hydrogen-Atom Transfer: Electron-Initiated Hydrofunctionalization of Alkenes, *J. Am. Chem. Soc.*, 2022, **144**, 17783-17791.
2. G. Zhang, R.-X. Bai, C.-H. Li, C.-G. Feng and G.-Q. Lin, Halogenation of 1,1-diarylethylenes by N-halosuccinimides, *Tetrahedron*, 2019, **75**, 1658-1662.
3. K. Xie, S. Kemper and M. Oestreich, Dehydrative Coupling of 1,1-Diarylalkenes and Cyclohexa-2,5-diene-1-carbaldehyde Derivatives Induced by a B(C6F5)3-Initiated [1,2]-Alkyl Migration, *The Journal of Organic Chemistry*, 2023, **88**, 10310-10313.
4. N. Salaverri, R. Mas-Ballesté, L. Marzo and J. Alemán, Visible light mediated photocatalytic [2 + 2] cycloaddition/ring-opening rearomatization cascade of electron-deficient azaarenes and vinylarenes, *Commun Chem.*, 2020, **3**, 132.
5. J. B. Lee, A. Zgair, J. Malec, T. H. Kim, M. G. Kim, J. Ali, C. Qin, W. Feng, M. Chiang, X. Gao, G. Voronin, A. E. Garces, C. L. Lau, T.-H. Chan, A. Hume, T. M. McIntosh, F. Soukarieh, M. Al-Hayali, E. Cipolla, H. M. Collins, D. M. Heery, B. S. Shin, S. D. Yoo, L. Kagan, M. J. Stocks, T. D. Bradshaw, P. M. Fischer and P. Gershkovich, Lipophilic activated ester prodrug approach for drug delivery to the intestinal lymphatic system, *J. Controlled Release*, 2018, **286**, 10-19.
6. R. Martinez-Haya, L. Marzo and B. König, Reinventing the De Mayo reaction: synthesis of 1,5-diketones or 1,5-ketoesters via visible light [2+2] cycloaddition of β-diketones or β-ketoesters with styrenes, *Chem. Commun.*, 2018, **54**, 11602-11605.
7. K. Hao, D. Li, D. Fu, P. Zou, S. Xie, Y. Lan and Y. Chen, Metal-Free 1,3-Boronate Rearrangement to Ketones Driven by Visible Light, *Angew. Chem. Int. Ed.*, 2024, **63**, e202316481.
8. Z. He, X. Qi, S. Li, Y. Zhao, G. Gao, Y. Lan, Y. Wu, J. Lan and J. You, Transition-Metal-Free Formal Decarboxylative Coupling of α-Oxocarboxylates with α-Bromoketones under Neutral Conditions: A Simple Access to 1,3-Diketones, *Angew. Chem. Int. Ed.*, 2015, **54**, 855-859.
9. Y. Liu, Y. Mao, Y. Hu, J. Gui, L. Wang, W. Wang and S. Zhang, The Employment of Sodium Hydride as a Michael Donor in Palladium-catalyzed Reductions of α, β-Unsaturated Carbonyl Compounds, *Adv. Synth. Catal.*, 2019, **361**, 1554-1558.
10. P. C. Too, Y. L. Tnay and S. Chiba, Copper-catalyzed aerobic aliphatic C–H oxygenation with hydroperoxides, *Beilstein J. Org. Chem.*, 2013, **9**, 1217-1225.
